# CDK7/CDK9 mediates transcriptional activation to prime paraptosis in cancer cells

**DOI:** 10.1186/s13578-024-01260-2

**Published:** 2024-06-10

**Authors:** Shih-Kai Chiang, Wei-Chao Chang, Shuen-Ei Chen, Ling-Chu Chang

**Affiliations:** 1 Department of Animal Science, National Chung Hsing University, Taichung, 40227 Taiwan; 2 Center for Molecular Medicine, China Medical University Hospital, Taichung, 406040 Taiwan; 3 Research Center for Cancer Biology, China Medical University, Taichung, 406040 Taiwan; 4 Cancer Biology and Precision Therapeutics Center, China Medical University, Taichung, 406040 Taiwan; 5 The iEGG and Animal Biotechnology Center, National Chung Hsing University, Taichung, 40227 Taiwan; 6 Innovation and Development Center of Sustainable Agriculture (IDCSA), National Chung Hsing University, Taichung, 40227 Taiwan; 7 i-Center for Advanced Science and Technology (iCAST), National Chung Hsing University, Taichung, 40227 Taiwan

**Keywords:** Cancer, Paraptosis, Cyclin-dependent kinase 7/9, Heat shock proteins, Reactive oxygen species, Nuclear stress, Protein kinase R

## Abstract

**Background:**

Paraptosis is a programmed cell death characterized by cytoplasmic vacuolation, which has been explored as an alternative method for cancer treatment and is associated with cancer resistance. However, the mechanisms underlying the progression of paraptosis in cancer cells remain largely unknown.

**Methods:**

Paraptosis-inducing agents, CPYPP, cyclosporin A, and curcumin, were utilized to investigate the underlying mechanism of paraptosis. Next-generation sequencing and liquid chromatography-mass spectrometry analysis revealed significant changes in gene and protein expressions. Pharmacological and genetic approaches were employed to elucidate the transcriptional events related to paraptosis. Xenograft mouse models were employed to evaluate the potential of paraptosis as an anti-cancer strategy.

**Results:**

CPYPP, cyclosporin A, and curcumin induced cytoplasmic vacuolization and triggered paraptosis in cancer cells. The paraptotic program involved reactive oxygen species (ROS) provocation and the activation of proteostatic dynamics, leading to transcriptional activation associated with redox homeostasis and proteostasis. Both pharmacological and genetic approaches suggested that cyclin-dependent kinase (CDK) 7/9 drive paraptotic progression in a mutually-dependent manner with heat shock proteins (HSPs). Proteostatic stress, such as accumulated cysteine-thiols, HSPs, ubiquitin-proteasome system, endoplasmic reticulum stress, and unfolded protein response, as well as ROS provocation primarily within the nucleus, enforced CDK7/CDK9–Rpb1 (RNAPII subunit B1) activation by potentiating its interaction with HSPs and protein kinase R in a forward loop, amplifying transcriptional regulation and thereby exacerbating proteotoxicity leading to initiate paraptosis. The xenograft mouse models of MDA-MB-231 breast cancer and docetaxel-resistant OECM-1 head and neck cancer cells further confirmed the induction of paraptosis against tumor growth.

**Conclusions:**

We propose a novel regulatory paradigm in which the activation of CDK7/CDK9–Rpb1 by nuclear proteostatic stress mediates transcriptional regulation to prime cancer cell paraptosis.

**Graphical Abstract:**

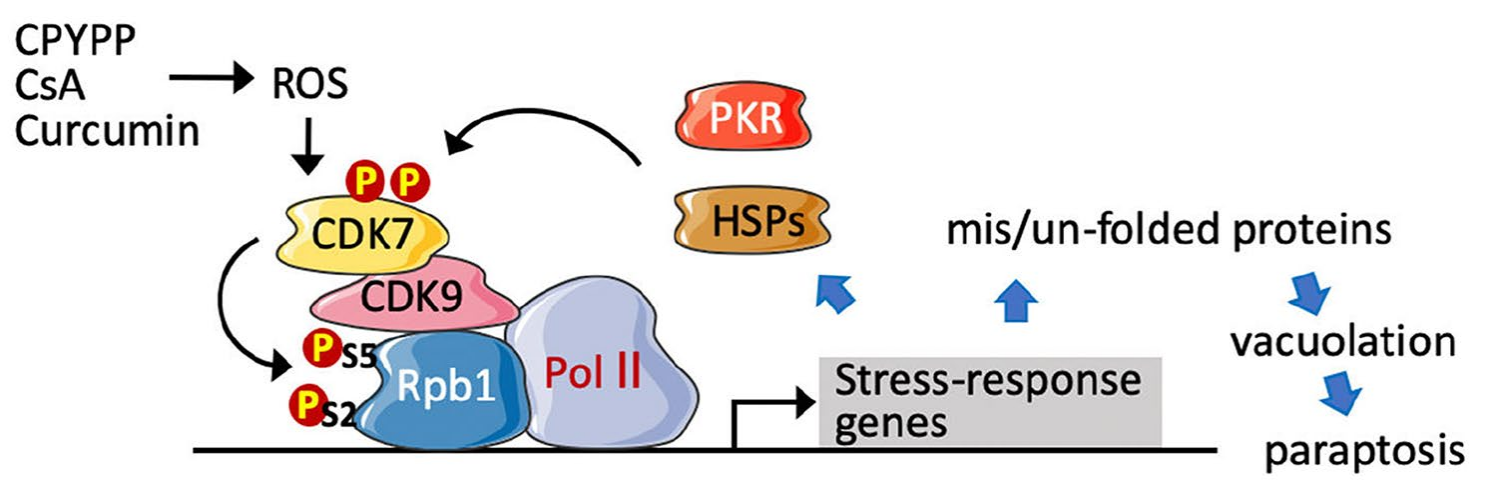

**Supplementary Information:**

The online version contains supplementary material available at 10.1186/s13578-024-01260-2.

## Introduction

Paraptosis, a non-apoptotic cell death characterized by the massive accumulation of cytoplasmic vacuoles [1, 2], has been suggested to be associated with neurodegenerative diseases [3] and viral infections [4]. Various stimuli, such as the activation of IGF1R and TAJ/TROY receptors and hormonal inductions, have been shown to induce paraptosis [1, 2, 5]. Recently, several small-molecule compounds have demonstrated significant anti-cancer effects by activating the paraptotic program [5–10] and may thus represent a new strategy against cancer growth, especially in cases resistant to apoptotic induction [8, 9].

The induction of cell paraptosis largely depends on the activation of MAPK members but is independent of caspase activation and the formation of apoptotic bodies [2, 9–11]. Paraptosis proceeds with the provocation of reactive oxygen species (ROS), prolonged endoplasmic reticulum (ER) stress, and the unfolded protein response (UPR) [6, 7, 10], as well as an imbalance in intracellular Ca^2+^ and Na^+^/K^+^ traffic [10–13] and the disruption of proteostasis [14–16]. Dysfunctional endoplasmic reticulum-associated protein degradation (ERAD) and big conductance calcium-activated potassium channels (BKCa) contribute to the cytoplasmic vacuolization morphology due to severely dilated ER and mitochondrial swelling caused by increased osmotic pressure [12]. Pro-paraptotic compounds have been shown to initiate ER stress-mediated transcriptional upregulation, involving UPR and proteostatic dynamics. Blocking *de novo* protein synthesis has been demonstrated to alleviate proteotoxicity and prevent cell paraptosis [1, 6, 7, 12, 17, 18]. However, the specific effectors and events that mediate the paraptotic program, particularly stress-specific transcriptional and translational activation in cancer cells, remain elusive.

Cyclin-dependent kinase (CDK) 7, the primary regulator of the cell cycle, interacts with cyclin H and MAT1 to form the CDK complex, which activates downstream CDKs and drives cell cycle progression [19, 20]. The CDK activating complex also associates with TFIIH (transcription initiation factor II H) to phosphorylate Rpb1 (RNAPII subunit B1, the largest subunit of RNA polymerase II) to facilitate transcription initiation [20]. Additionally, CDK7 activates the CDK9/cyclin T1 complex (TFEB) to promote the elongation process [21]. While CDK7 and CDK9 pathways have recently been targeted in cancer treatment [22, 23], further elucidation is required regarding their involvement in the paraptotic program.

This study aimed to uncover the underlying mechanisms of paraptotic progression and evaluate paraptotic induction as an effective approach in cancer therapy. The results demonstrate that CPYPP, cyclosporin A (CsA), and curcumin significantly disrupt redox homeostasis and proteostasis to initiate paraptosis in melanoma and breast cancer cells. The paraptotic program involves transcriptional activation associated with redox homeostasis and proteostasis, with the nucleus experiencing more substantial proteostatic stress compared to the cytosol. Importantly, CDK7/CDK9–Rpb1 and its interaction with heat shock proteins (HSPs) and protein kinase R (PKR) were identified as responsive to proteostatic stress and mediating transcriptional activation in a forward regulatory loop, thereby exacerbating paraptotic progression. The effectiveness of paraptotic induction against tumor growth was further confirmed in xenograft mouse models using breast cancer cells and chemotherapy-resistant head and neck carcinoma cells.

## Results

### CPYPP, CsA, and curcumin induce paraptosis in cancer cells

**Fig. 1:**
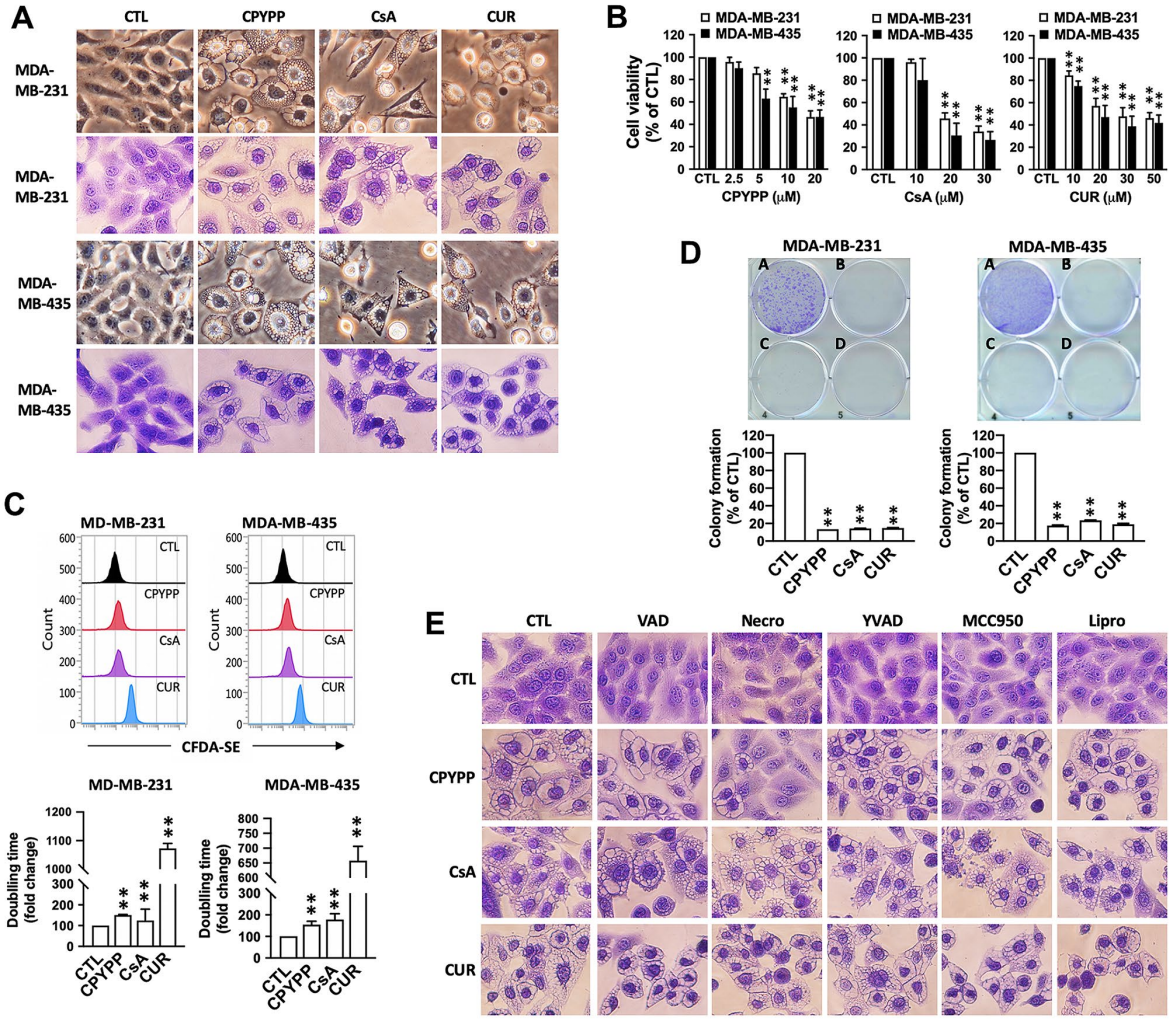
CPYPP, cyclosporin A, and curcumin induce paraptotic death in cancer cells. (**A**–**D**) Human cancer cells MDA-MB-231 and MDA-MB-435 were treated with CPYPP (10 µM), cyclosporin A (CsA, 20 µM), or curcumin (CUR, 30 µM) at the indicated concentrations for 24 h. Cells were then assessed for morphological alterations in cytoplasmic vacuolization (upper panel, phase-contrast; lower panel, crystal violet staining, A), cell viability (MTT assay, B), proliferation (CFDA-SE staining, C), and clonogenic activity (A, CTL; B, CPYPP; C, CsA; D, CUR) (**D**). (**E**) MDA-MB-435 cells pretreated with various inhibitors of programmed cell death, including z-VAD-FMK (VAD, 10 µM, apoptosis), necrostatin-1 (Necro, 100 µM, necroptosis), Ac-YVAD-CHO (YVAD, 10 µM, pyroptosis), MCC950 (10 µM, pyroptosis), or liproxstatin-1 (Lipro, 2 µM, ferroptosis), for 30 min were treated with CPYPP (10 µM), cyclosporin A (CsA, 20 µM), or curcumin (CUR, 30 µM) for 24 h to trigger cell paraptosis. Cells were collected for morphological vacuolization analysis. The results are expressed as the mean ± SD from three independent experiments. **P < 0.01 compared to the control group (CTL)

CPYPP, a DOCK (dedicator of cytokinesis) inhibitor known for its anti-cancer effects by disrupting the interaction between DOCK2 and Rac1 [24–26], as well as CsA and curcumin, induced significant cytoplasmic vacuolization (Fig. 1A). These compounds also suppressed cell viability in a concentration-dependent manner (Fig. 1B). The half-maximal inhibitory concentration (IC_50_) values of cell viability for CPYPP, CsA, and curcumin in MDA-MB-231 and MDA-MB-435 were 19.43 ± 0.91 µM vs. 12.93 ± 2.16 µM, 22.75 ± 1.15 µM vs. 17.72 ± 4.97 µM, and 23.68 ± 1.98 µM vs. 23.33 ± 2.45 µM, respectively. Therefore, 10 µM CPYPP, 20 µM CsA, and 30 µM curcumin were employed to investigate the paraptosis mechanism. CPYPP, CsA, and curcumin all inhibited cell proliferation and clonogenic activity (Fig. 1C and D) and arrested the cell cycle at the G_2_/M phase (Supplemental Fig. S1) in MDA-MB-231 and MDA-MB-435 cancer cells. Genetic knockdown of DOCK1 suppressed cell viability without inducing vacuolization morphology but exacerbated cell paraptosis when treated with CPYPP. Furthermore, knockdown of Rac1 and Rac2 had no effects on cell viability and vacuolization, whether in the presence or absence of CPYPP (Supplemental Fig. S2A–C). These results suggest that paraptotic cell death induced by CPYPP is not dependent on DOCK1 activation.

The paraptotic program was found to be independent of other types of cell death, as inhibitors like z-VAD-FMK (apoptosis), MCC950 (NLRP3, pyroptosis), Ac-YVAD-CHO (caspase-1/-4, pyroptosis), and liproxstatin-1 (ferroptosis) failed to alter paraptotic vacuolization (Fig. 1E and Supplemental Fig. S3A). Interestingly, the necroptosis inhibitor, necrostatin-1, prevented CPYPP-induced vacuolization but had no effect on CsA and curcumin induction. Other necroptosis inhibitors, GSK872 (RIPK3 inhibitor) and necrosulfonamide (MLKL inhibitor), also had no such suppressive effects (Fig. 1E and Supplemental Fig. S3A, B). Furthermore, CPYPP, CsA, and curcumin induced cytoplasmic vacuolization and cell death in glioblastoma cells (DBTRG-05MG and U-87 MG), pancreatic cancer cells (PANC-1), and lung cancer cells (NCI-H1299) (Supplemental Fig. S3C). However, they had much less pronounced effects on the normal breast epithelial cell line MCF 10A (Supplemental Fig. S3D).

### Paraptosis proceeds from transcriptional activation involved in redox homeostasis and proteostasis

**Fig. 2:**
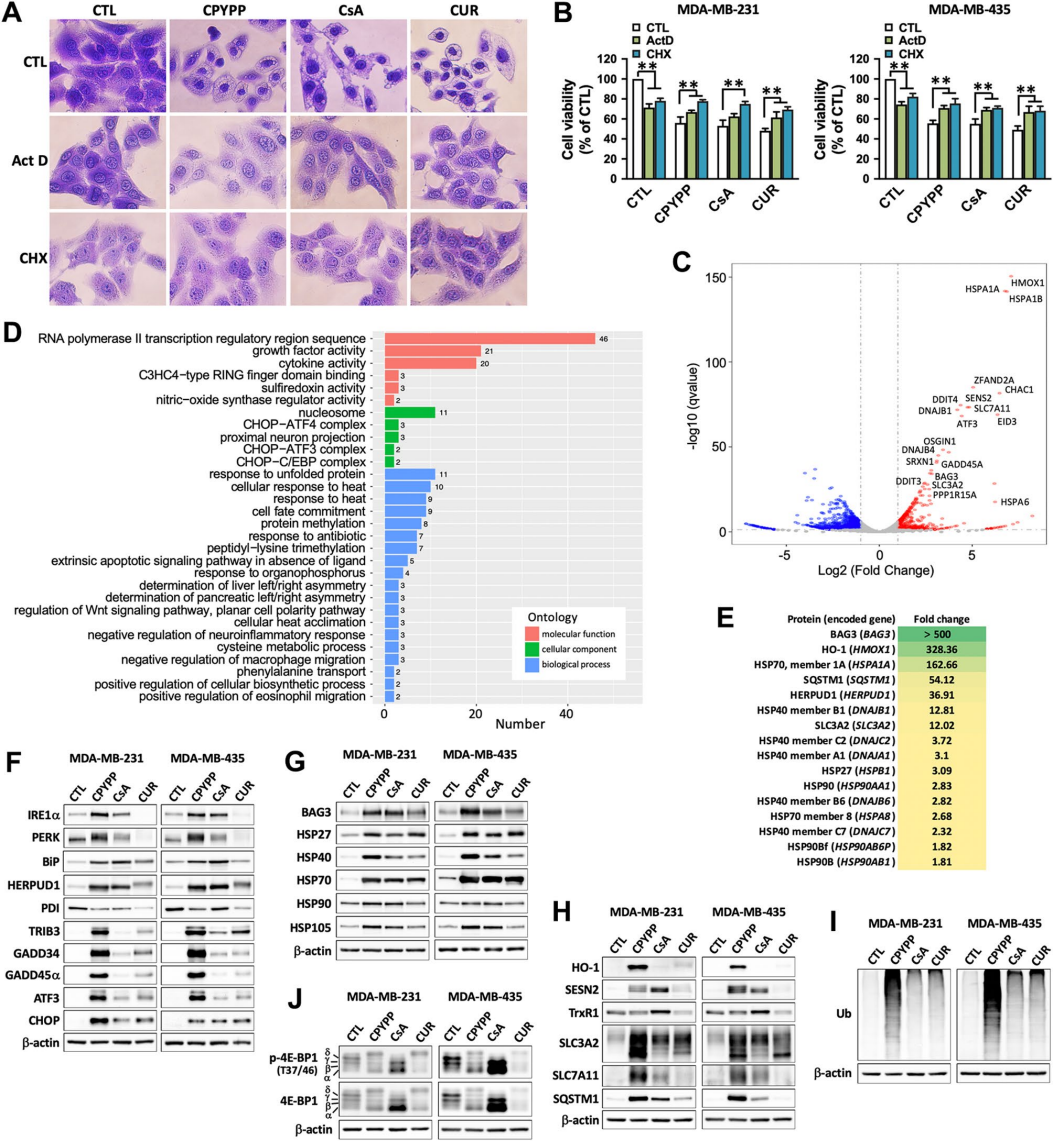
Paraptosis proceeds with activation of proteostatic dynamics and transcriptional regulation involved in redox homeostasis and proteostasis. (**A**, **B**) Cancer cells were pretreated with the transcription inhibitor actinomycin A (ActD, 1 µM) or the translation inhibitor cycloheximide (CHX, 20 µM) for 30 min, followed by treatment with CPYPP (10 µM), cyclosporin A (CsA, 20 µM), or curcumin (CUR, 30 µM) for 24 h. The cells were then assessed for morphological vacuolization alterations using crystal violet staining (A, MDA-MB-435 cells) or for cell viability via MTT assay (**B**). The results are expressed as the mean ± SD from three independent experiments. **P < 0.01, compared to the control group (CTL). (**C**, **D**) CPYPP-treated MDA-MB-435 cells underwent next-generation sequencing (NGS) to analyze differential gene expressions (DGEs) The volcano plot represents the distribution of DGEs based on the FPKM analysis with at least 2-fold changes (**C**). Results from the Gene Ontology (GO) analysis classify molecular function, cellular component, and biological process (**D**), along with the number of related significant changed genes. (**E**) CPYPP-treated MDA-MB-435 cells were subjected to mass spectrometric analysis for proteomic profiling with at least 1.5-fold changes. (**F**–**J**) Western blot analysis confirmed the involvement of differentially expressed genes in ER stress (**F**), HSP chaperones (**G**), redox homeostasis (**H**), protein ubiquitination (**I**), and translational activity (**J**) in cancer cells treated with CPYPP, CsA, or CUR for 24 h

Similar to previous reports [1, 7], the inhibition of transcriptional and translational activity by actinomycin D (ActD) and cycloheximide (CHX), respectively, abrogated paraptotic vacuolization and rescued cell viability (Fig. 2A and B, and Supplemental Fig. S4A), despite ActD having a pro-apoptotic effect and causing ROS provocation [27]. Next-generation sequencing (NGS) analysis of differential gene expressions (DGEs) showed that gene profiling was significantly altered by CPYPP in both MDA-MB-231 and MDA-MB-435 cancer cells (Fig. 2C and Supplemental Table S1). CPYPP significantly upregulated 320 genes in MDA-MB-231 and 572 genes in MDA-MB-435, whereas downregulation affected 212 genes in MDA-MB-231 and 1359 genes in MDA-MB-435. Interestingly, significantly increased genes mainly involved in HSP chaperones, ER stress, UPR, autophagy, and redox homeostasis, including *HMOX1*, *HSPA1A*, *HSPA1B*, *DNAJB1*, *DNAJB4*, *ATF3*, *BAG3*, *GADD45A*, *SESN2*, *SLC7A11*, *SLC3A2*, and *PPP1R15A*, were significantly upregulated by CPYPP (Fig. 2C, Supplemental Fig. S4B, Table S1). Gene Ontology (GO) analysis further suggested active operations by the DGEs in transcriptional activation, stress response, protein chaperoning, and the ubiquitin-proteasome system (UPS) for proteostasis (Fig. 2D). However, the downregulated genes in both cancer cell lines lack similarity in gene ontology analysis (data not shown).

Mass spectrometry analysis confirmed that most DGEs functioned in cellular redox status and proteostasis, such as BAG3, HO-1, HSP70, SQSTM1, HERPUD1, HSP40, and SLC3A2 (Fig. 2E). Accordingly, proteins involved in ER stress, the HSP family, thiol redox homeostasis, and autophagy (Fig. 2F–H), as well as protein polyubiquitination (Fig. 2I), were remarkably upregulated, particularly by CPYPP via transcriptional regulation as affected by ActD and CHX (Supplemental Fig. S4C–E). Paraptotic transcription was supported by an increase in rate-limiting cap-dependent activity in the translation process, specifically the δ isoform of 4E-BP1 by CPYPP and curcumin, and the α isoform by CsA (Fig. 2J). These results suggest that paraptosis involves the activation of proteostatic dynamics, followed by transcriptional activation for a stress-specific transcriptome and translational reprogramming for protein synthesis involved in the integrated stress response (ISR) [28].

### CDK7 and CDK9 mediate transcriptional regulation in paraptosis progression

**Fig. 3:**
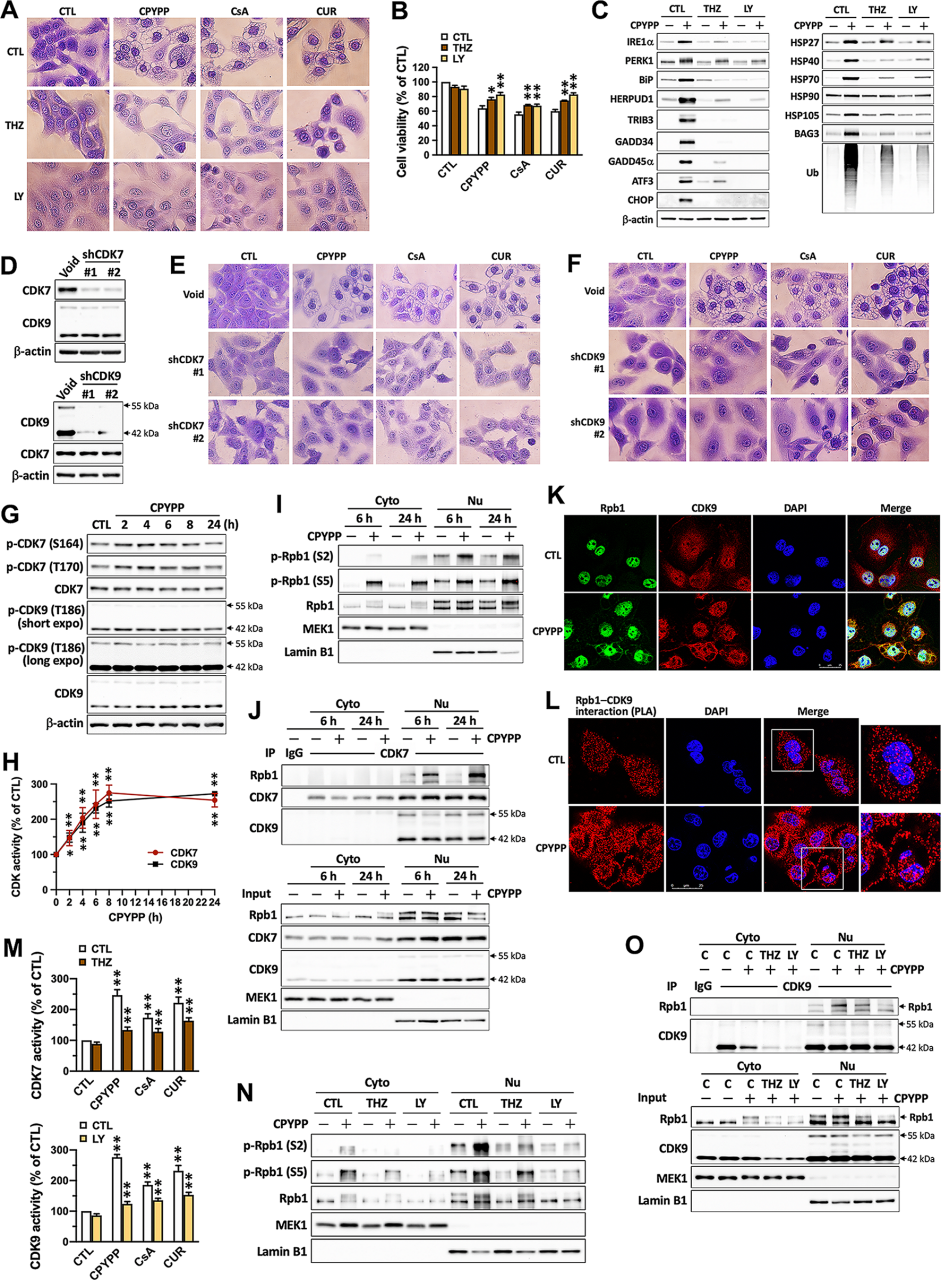
CDK7/CDK9 mediates the transcriptional regulation for paraptotic development. (**A**–**C**) MDA-MB-435 cells pretreated with the CDK7 inhibitor THZ1 (THZ, 0.2 µM) or the CDK9 inhibitor LY2857785 (LY, 1 µM) for 30 min were treated with CPYPP (10 µM), cyclosporin A (CsA, 20 µM), or curcumin (CUR, 30 µM) for 24 h. Cells were then collected for vacuolization (**A**), cell viability (**B**), and protein expression (**C**) analyses. (**D**–**F**) MDA-MB-435 cells were treated with control shRNA (Void), shCDK7, or shCDK9 for 4 days and then with CPYPP (10 µM) for 24 h. Cells were harvested for Western blot analysis (**D**) and vacuolization alterations (**E**, **F**). (**G**, **H)** MDA-MB-435 cells were treated with CPYPP (10 µM) for the indicated time and then collected for Western blot and CDK7/CDK9 enzyme activity analysis. (**I**, **J**) MDA-MB-435 cancer cells were treated with CPYPP for 6–24 h, then harvested to isolate cytosolic and nuclear fractions for Rpb1 phosphorylation (**I**) and CDK7–Rpb1 complex activation by co-immunoprecipitation method (**J**). (**K**) The co-localization of Rpb1 and CDK9 was detected through co-immunofluorescence staining using anti-Rpb1 antibody (shown in green) and anti-CDK9 antibody (shown in red), followed by secondary antibody staining, along with DAPI (shown in blue) for nuclei counter staining. The images were captured using confocal microscopy at 630× magnification. The scale bar represents 25 μm. (**L**) The interaction and distribution between Rpb1 and CDK9 were assessed using the proximity ligation assay. (**M**–**O**) MDA-MB-435 cells pretreated with the CDK7 inhibitor THZ1 (THZ, 0.2 µM) or the CDK9 inhibitor LY2857785 (LY, 1 µM) for 30 min were treated with CPYPP (10 µM), cyclosporin A (CsA, 20 µM), or curcumin (CUR, 30 µM) for 24 h. Cells were collected for CDK7/CDK9 activity analysis (**M**) or to isolate cytosolic and nuclear fractions for Rpb1 phosphorylation (**N**) and CDK9–Rpb1 complex activation by co-immunoprecipitation method (**O**). **P < 0.01, compared to the corresponding control group (CTL)

Transcription is the key event in regulating paraptosis [1]. The cell cycle is significantly arrested by all three paraptosis-inducing agents, suggesting the involvement of cell cycle regulators. CDKs play essential roles in regulating both cell division and transcription [29, 30]. To investigate whether CDKs regulate paraptosis, several CDK pharmacological inhibitors were applied (Fig. 3A). THZ1 (CDK7 inhibitor), PHA-767491 (CDK7/9 inhibitor), and LY2857785 (CDK9 inhibitor) remarkably modulated vacuolization (Fig. 3B, Supplemental Fig. S5A and B), rescued cell viability (Fig. 3C, Supplemental Fig. S5C), and alleviated the upregulation of ER stress and UPR proteins, HSPs, and protein ubiquitination induced by CPYPP, CsA, and curcumin (Fig. 3C). Other CDK inhibitors, such as RO-3306 (CDK1 inhibitor), Roscovitine (CDK2 inhibitor), Abemaciclib, and Palbociclib (CDK4/CDK6 inhibitor), BML-259 (CDK5 inhibitor), BRD6989, and Senexin A (CDK8 inhibitor), as well as SR-4835 and THZ531 (CDK12/13 inhibitor), had no effect on vacuolation and cell viability (Supplemental Fig. S5A–C). The abrogation of cytoplasmic vacuolization by CDK7 and CDK9 knockdown confirmed their critical role in mediating paraptotic vacuolization (Fig. 3D–F). CPYPP promoted CDK7 phosphorylation at S164 and T170 within 2 h and sustained this effect up to 8 h but had no effects on the activated site T186 of CDK9 (Fig. 3G). Both CDK7 and CDK9 activities increased promptly upon CPYPP treatment, reaching their maximum at 8 h and remaining elevated up to 24 h (Fig. 3H). As early as 6 h of treatment, CPYPP significantly increased Rpb1 phosphorylation and its interaction with CDK7 and CDK9 in the nuclear compartment (Fig. 3I and J). The co-immunofluorescence staining and proximity ligation assay demonstrated the co-localization and interaction of CDK9 and Rpb1, further confirming their predominant distribution in the nuclear compartment (Fig. 3K and L). THZ1 and LY2857785 mitigated the paraptotic increase in CDK7 and CDK9 activity (Fig. 3M), attenuated Rpb1 activation, and reduced its interaction with CDK7/CDK9 (Fig. 3N and O), suggesting the obligatory role of CDK7/CDK9 in driving transcriptional activation during paraptotic development.

### CDK7/CDK9–Rpb1 interacts with HSPs to amplify paraptotic transcription in a forward loop and reciprocally-regulated manner

**Fig. 4:**
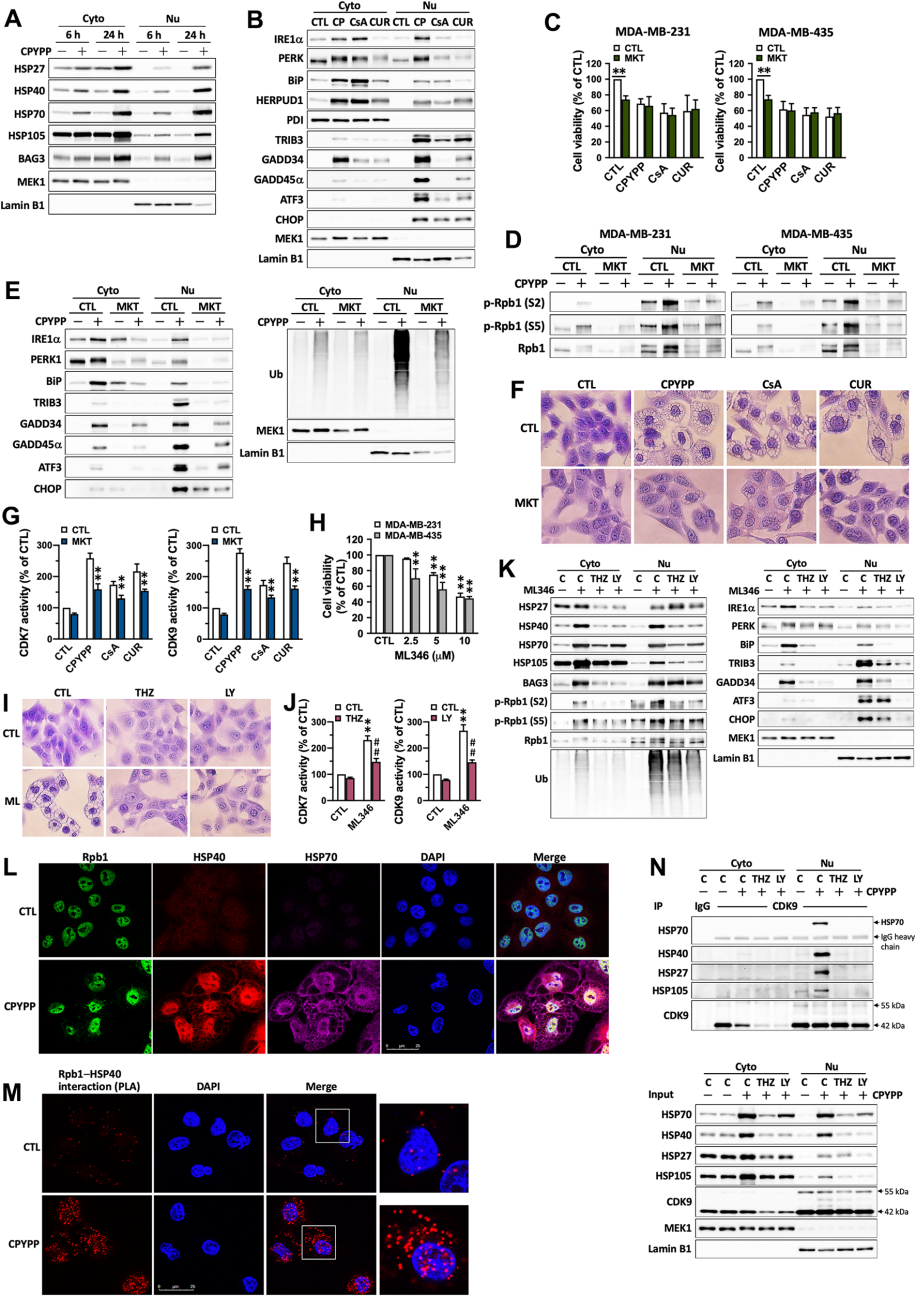
HSPs interact with the CDK7/CDK9–Rpb1 complex to prime stress-specific transcription in a forward loop and reciprocally regulated manner. (**A**, **B**) MDA-MB-435 cells were treated with CPYPP (10 µM) for 6–24 h, or with CPYPP, cyclosporin A (CsA, 20 μM), or curcumin (CUR, 30 μM) for 24 h, and then collected for cytosolic (Cyto) and nuclear (Nu) fraction isolation for Western blot analysis. (**C**–**G**) MDA-MB-435 and/or MDA-MB-231 cells were pretreated with MKT-077 (MKT) for 30 min followed by CPYPP (10 µM), cyclosporin A (CsA, 20 µM), or curcumin (CUR, 30 µM) for 24 h. Cells were then collected for cell viability (**C**), RNA polymerase II activation (**D**), ER stress, UPR, and protein ubiquitination (**E**), paraptotic vacuolization (**F**), and CDK7 and CDK9 activity (**G**) analysis. The results are expressed as the mean ± SD from three independent experiments. **P < 0.01, compared to the corresponding control group. (**H**, **I**) Cancer cells treated with indicated concentrations of ML346 for 24 h promoted cell death (**H**) and paraptotic vacuolization (I, 10 µM). (**J**, **K**, **N)** MDA-MB-435 cells were pretreated with THZ1 (THZ, 0.2 µM) or LY2857785 (LY, 1 µM) for 30 min followed by ML346 treatment for 24 h. Total cell lysates, cytosolic and nuclear fractions were used for CDK7 and CDK9 activity (**J**), protein expression (**K**), and the interaction of HSPs with CDKs by co-immunoprecipitation (**N**) analysis. **P < 0.01, compared to the corresponding control group (CTL). (**L**) The co-localization of Rpb1, HSP40, and HSP70 was examined in CPYPP-treated MDA-MB-435 cells co-stained with anti-Rpb1 (shown in green), anti-HSP40 (shown in red), and anti-HSP70 (shown in magenta) antibodies, followed by individual secondary antibodies. DAPI staining (shown in blue) was used for nuclei counterstaining. Confocal microscopy was utilized to capture the images at 630× magnification. The scale bar represents 25 μm. (**M**) The interaction and distribution between Rpb1 and HSP40 were evaluated using the proximity ligation assay

CPYPP promptly increased HSP protein expressions, including HSP27, HSP40, HSP70, and HSP105 (Supplemental Fig. S6A), with particularly rapid activation of HSP27 within 2 h, while ER stress proteins were upregulated after 4–6 h of treatment (Supplemental Fig. S6B). Time-lapse observations showed visible vacuole formation at 8 h and pronounced vacuolization after 10 h of treatment (Supplemental Fig. S6C), suggesting that transcriptional activation precedes vacuolization alteration (Supplemental Table S1, Fig. S4B). After 6 h of treatment, CPYPP, CsA, and/or curcumin upregulated both cytosolic and nuclear HSPs and the co-chaperone BAG3 (BCL-2-associated athanogene 3) (Fig. 4A), as well as ER stress and UPR proteins. Surprisingly, some cascading effectors were profoundly localized in the nucleus (Fig. 4B and Supplemental Fig. S7A), while PDI (protein disulfide isomerase) remained unchanged in the cytosol and was not detected in the nuclear fractions. The nuclear compartmentalization of HSPs and ER stress proteins occurred within 6 h of treatment, preceding pronounced vacuolization (Fig. 4A and Supplemental Figs. S6C, S7A). Nuclear localization of HSPs and the cascading effectors such as IRE1α, PERK, and GADD45α were visualized not only on the nuclear membranes but also within the nucleus (Supplemental Fig. S7B–F). In the absence of paraptotic compounds, MKT-077, which interferes with ATP hydrolysis to dysfunction HSP70 [31], suppressed cell viability and Rpb1 activation (Fig. 4C and D) with differential effects on UPR protein abundances (Fig. 4E). However, it had no effects on cell morphology, CDK7 and CDK9 activity, and protein ubiquitination (Fig. 4E and G). MKT-077 reversed CPYPP-induced vacuolization, CDK7/CDK9 activity, Rpb1 activation, UPR protein abundances, and protein ubiquitination in both cytosolic and nuclear fractions (Fig. 4D–G), without significant effects on cell viability (Fig. 4C) and HSP abundances (Supplemental Fig. S7G).

In the paraptosis induction system, several HSPs were transcriptionally upregulated, implying that multiple HSPs contribute to paraptosis. ML346, an activator of HSP70 expression and HSF-1 (heat shock factor 1) activity [32], was used to investigate the potential roles of HSPs in paraptosis. Treatment with ML346 induced cell paraptosis (Fig. 4H) in a CDK7 and CDK9-dependent manner, as demonstrated by the attenuation of vacuolization by THZ1 and LY2857785 (Fig. 4I), as well as the reduction in CDK7 and CDK9 activity (Fig. 4J). Additionally, ML346 treatment led to alterations in ER stress and UPR proteins and protein ubiquitination, particularly in the nuclear fractions (Fig. 4K). Interestingly, ML346 not only upregulated HSP70 expression but also other HSPs, including HSP27, HSP40, and HSP105, in a CDK7 and CDK9-dependent manner (Fig. 4K). These results demonstrate that the early activation of HSPs occurs upstream in the sequence of proteostatic dynamics. This, in turn, elicits stress-specific transcriptional activation, resulting in cytoplasmic vacuolization and ultimately driving cells into paraptosis, particularly in cancer cells [33]. CPYPP upregulated HSP27, HSP40, HSP70, and HSP105 expressions, and their interaction with CDK7/CDK9 within the nucleus after 6 h of treatment (Fig. 4L–N, Supplemental Fig. S8A, B) in a CDK7/CDK9-dependent manner (Fig. 4N). Additionally, ML346 exacerbated paraptotic cell death, consistent with the upregulation of ER stress and UPR proteins, and protein ubiquitination in the nucleus (Supplemental Fig. S9A, B). As HSP and CDK7/CDK9 functions were reciprocally dependent (Fig. 4G, J, K and N), and HSPs, as downstream products of CDK7/CDK9-mediated transcription (Figs. 2C–E and 3C, and Supplemental Table S1), responded to cellular proteostatic stress as early as within 6 h. Therefore, the interaction of HSPs with CDK7/CDK9–Rpb1 enforces transcriptional regulation in a forward loop, exacerbating paraptotic progression.

### Activation of HSPs and UPS elicits proteostatic stress primarily in the nucleus

**Fig. 5:**
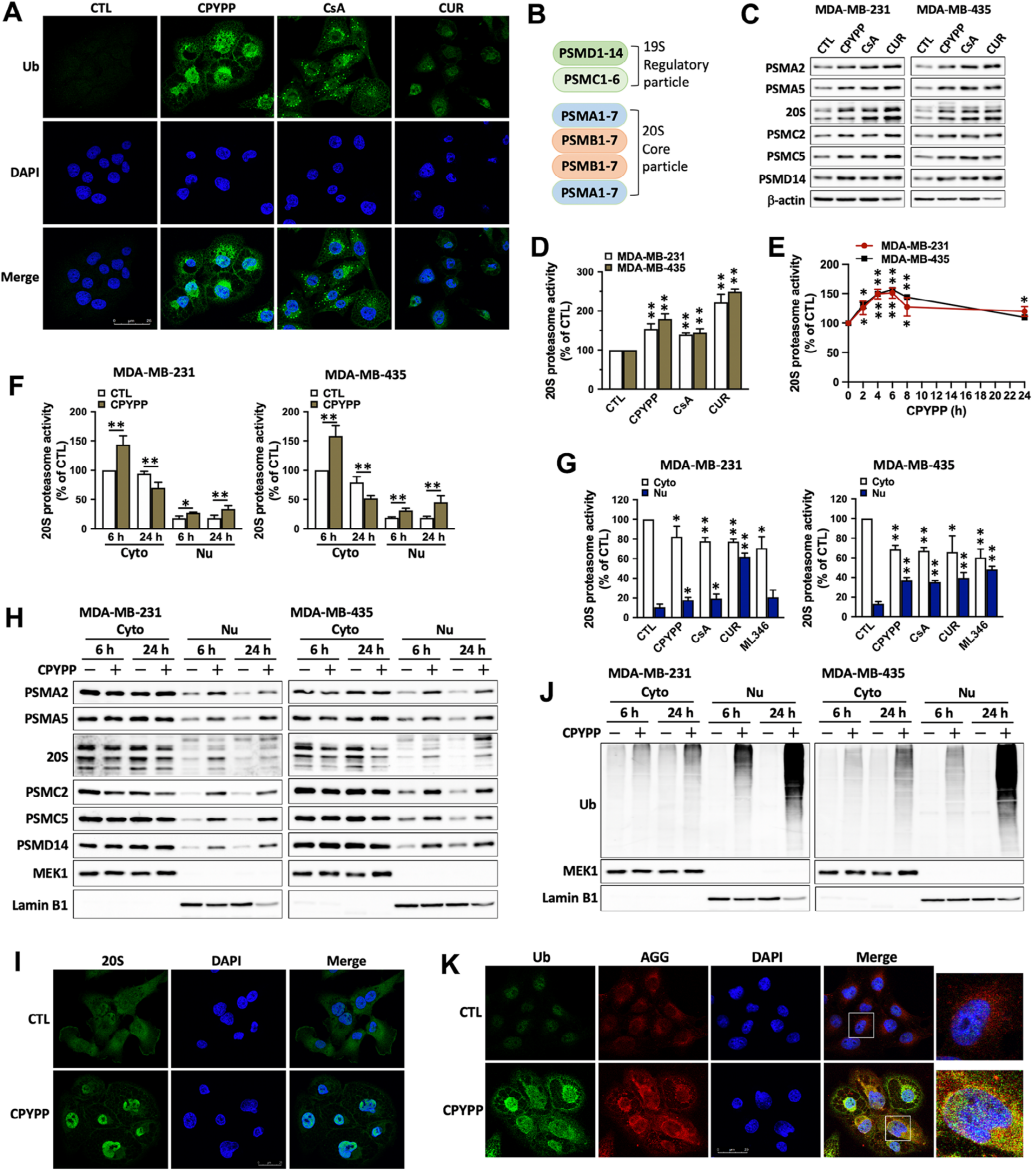
Overload of HSPs and the ubiquitin-proteasome system elicits unclear stress and promotes CDK7/CDK9–Rpb1 activation for transcriptional regulation. (**A**) MDA-MB-435 cells were treated with CPYPP (10 µM), cyclosporin A (CsA, 20 µM), or curcumin (CUR, 30 µM) for 24 h. Protein ubiquitination was visualized by immunofluorescence using an anti-ubiquitin antibody (shown in green), with DAPI (shown in blue) used for nuclei counterstaining. Results were imaged under a confocal microscopy at 630× magnification. Scale bar = 25 μm. (**B**) The subunits of the 19S and 20S proteasome complex. (**C**, **D**, **F**, **G**, **H**, **J)** Cancer cells were treated with CPYPP, CsA, CUR, or ML346 (10 µM) for 6 h (**D**, **G**) or 24 h and then harvested for whole lysate collection or cytosolic and nuclear fractionation for proteasome subunit abundance determination by Western blot (**C**, **H**), 20 S proteasome activity (**D**, **F**, **G**), and protein ubiquitination analysis (**J**). (**E**) Cancer cells were treated with CPYPP for the indicated time period (h). The results are expressed as the mean ± SD from three independent experiments. *P < 0.05, **P < 0.01, compared to the corresponding control group (CTL). (**I**,**K**) CPYPP-treated MDA-MB-435 cells were imaged by immunofluorescence staining for nuclear accumulation of proteasomes using an anti-20S core subunit antibody (green) and DAPI (blue) for nuclei counterstaining (**I**), or for colocalization of ubiquitins and aggresomes using an anti-ubiquitin antibody (green) with aggresome staining by ProteoStat® dye (red), and DAPI (blue) for nuclei counterstaining (**K**)

CPYPP, CsA, and curcumin dramatically promoted protein ubiquitination (Figs. 2I and 5A), consistent with the upregulated proteasome subunit abundances (PSMA2 and PSMA5 for 20S, and PSMC2, PSMC5, PSMD14 for 19S) (Fig. 5B and C), and total cellular 20S proteasome activity (Fig. 5D), where 20S proteasome activity increased after 2 h of stimulation, reached its maximum at 6 h, and then slightly declined until 24 h (Fig. 5E). Surprisingly, cytosolic 20S proteasome activity transiently increased after 6 h of treatment but was suppressed after 24 h, while nuclear 20S proteasome activity was constantly promoted (Fig. 5F and G). The nucleus even accumulated many more proteasome subunits and ubiquitinated proteins within 6 h of CPYPP treatment (Fig. 5H–J and Supplemental Fig. S10A–C), suggesting an early activation of UPS with HSPs in response to nuclear proteostatic stress. The increase in ubiquitinated proteins was primarily co-localized within aggresomes around the perinuclear regions (Fig. 5K).

ActD and CHX had no marked effects on cytosolic proteasome subunit abundances in the presence of CPYPP, but ActD enhanced and CHX suppressed the upregulation in the nucleus (Supplemental Fig. S10D). Inhibition of CDK7 and CDK9 by THZ1 and LY2857785, as well as HSP70 by MKT-077, enhanced CPYPP-induced nuclear proteasome activity and subunit accumulation (Supplemental Fig. S10E–I). Additionally, the E3 ligases SKP2 (S-phase 2 kinase-associated protein 2) and CUL4A (Cullin-4A) were downregulated after 24 h of CPYPP treatment, but MDM2 was enhanced, while the deubiquitinase CYLD, Cyclin D1, and p21 were upregulated (Supplemental Fig. S10J), suggesting an operative mechanism in UPS against cancer cell growth [34, 35].

The importin inhibitor ivermectin and the nuclear export inhibitor leptomycin B failed to alter paraptotic upregulation of cytosolic and nuclear proteasome subunits, despite slight increases in the nucleus (Supplemental Fig. S11A). Lamin B1 staining confirmed no significant alterations in nuclear membrane integrity (Supplemental Fig. S11B). These results demonstrate the critical role of nuclear UPS in clearing unfolded proteins under overactivated proteostatic dynamics, even in unstressed cancer cells with intrinsically higher transcriptional activities.

### ROS primes the paraptotic program by CPYPP

**Fig. 6:**
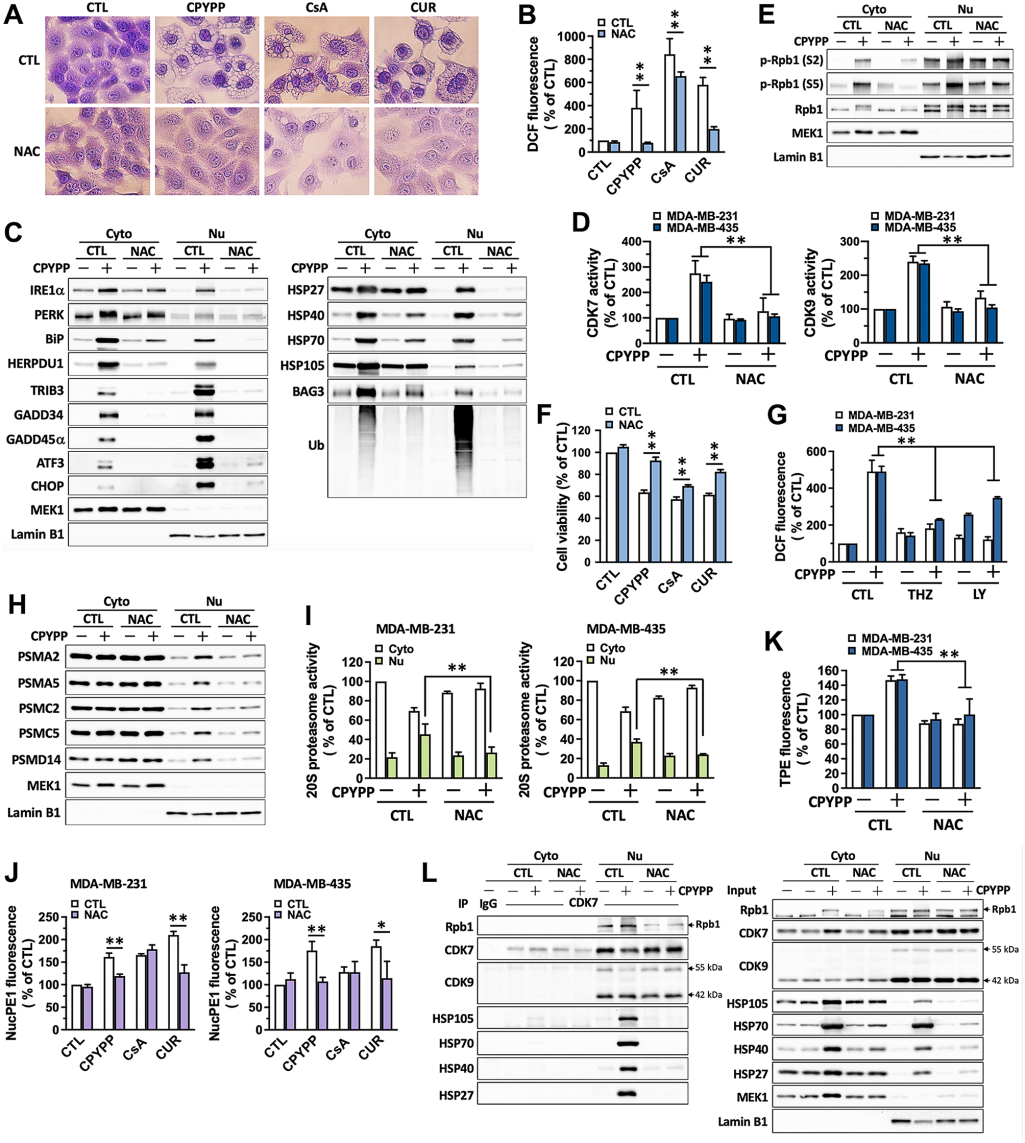
ROS ignites the paraptotic process by CPYPP. (**A**–**F**, **H**–**L**) MDA-MB-435 and/or MDA-MB-231 cells were pretreated with NAC (3 mM) for 30 min followed by CPYPP (10 µM), cyclosporin A (CsA, 20 µM), or curcumin (CUR, 30 µM) treatment for 24 h. Cells were collected for analyses, including morphological vacuolization (**A**), ROS generation (**B**), abundance of ER stress and UPR proteins, HSPs (**C**), CDK7 and CDK9 activity (**D**), RNA polymerase II activation (**E**), and cell viability (**F**), proteasome subunit abundances (**H**), 20S proteasome activity (**I**), nuclear hydrogen peroxide generation (**J**), free cysteine-thiol levels (**K**), and CDK7 interaction with HSPs by co-immunoprecipitation (**L**). (**G**) Cancer cells pretreated with THZ1 (THZ, 0.2 µM) or LY2857785 (LY, 1 µM) for 30 min followed by CPYPP treatment for 24 h were assessed for cellular ROS generation. The results are expressed as the mean ± SD from three independent experiments. *P < 0.05 or **P < 0.01, compared to the corresponding control group (CTL)

Proteolysis is a pivotal system to remove oxidatively damaged proteins [36]. We suspect that paraptosis elicits proteostatic stress, enhancing intracellular oxidative stress, and the increase in ROS may serve as a signal to mediate downstream events, including the transactivation of transcription and translation. We next examined the role of ROS in the paraptosis process. CPYPP, CsA, and curcumin induced cell paraptosis in a ROS-dependent manner, as the ROS scavenger *N*-acetyl-L-cysteine (NAC) abolished vacuolization and attenuated cellular ROS levels, ER stress, UPR proteins, HSPs, CDK7/CDK9 activity, Rpb1 activation, and suppressed cell viability (Fig. 6A–F and Supplemental Fig. S12A–D). THZ1 and LY2857785 relieved CPYPP-induced cellular ROS generation but not free cysteine-thiols, (Fig. 6G and Supplemental Fig. S12E), while NAC treatment attenuated nuclear accumulation of proteasome proteins and activity (Fig. 6H and I), hydrogen peroxide production (Fig. 6J), free cysteine-thiols (Fig. 6K), and abolished the interaction of the nuclear CDK7/CDK9 complex with Rpb1 and HSPs without altering Rpb1 abundance (Fig. 6L). These results suggest a reciprocally-dependent relationship between ROS provocation and CDK7/CDK9–Rpb1 activation. Furthermore, nuclear proteotoxic stress-induced ROS promotes stress-specific transcription via CDK7/CDK9 activation, exacerbating paraptotic progression.

### Protein kinase R (PKR) interacts with CDK7/CDK9–Rpb1 complex to enhance transcriptional regulation and exacerbate paraptotic progression

**Fig. 7:**
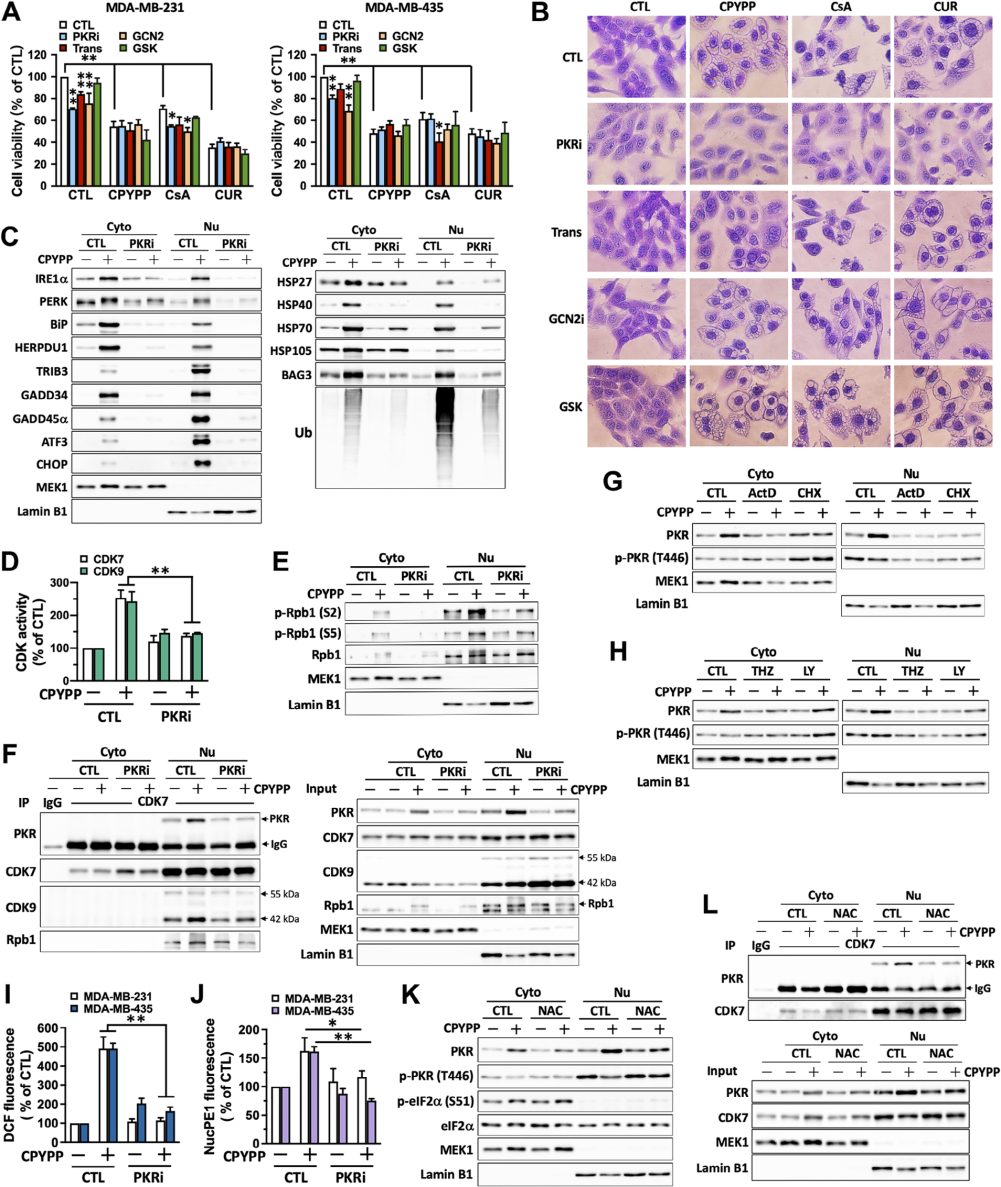
Protein kinase R (PKR) interacts with the CDK7/CDK9–Rpb1 complex to enhance transcription activation and exacerbate paraptotic progression. (**A**–**F**, **I**, **J**) MDA-MB-435 cells were pretreated with PKR inhibitor (PKRi, 2 µM), trans-ISRIB (Trans, 3 µM), GCN2-IN-1 (GCN2i, 5 µM), or GSK2656157 (GSK, 5 µM) for 30 min followed by CPYPP (10 µM), CsA (20 µM), or curcumin (CUR, 30 µM) for 24 h. Cells were harvested and assessed for cell viability (**A**), morphological vacuolization (**B**), protein abundance of ER stress, UPR, and HSPs (**C**), CDK7 and CDK9 activity (**D**), RNA polymerase II activation (**E**), PKR interaction with CDK7/CDK9 by co-immunoprecipitation (**F**), cellular ROS (**I**), and nuclear hydrogen peroxide generation (**J**). The results are expressed as the mean ± SD from three independent experiments. *P < 0.05 or **P < 0.01, compared to the corresponding control group (CTL). (**G**, **H**) MDA-MB-435 cells pretreated with ActD (1 µM), CHX (20 µM), THZ1 (0.2 µM), or LY2857785 (1 µM) for 30 min followed by CPYPP treatment for 24 h. Cytosolic and nuclear fractions were isolated for PKR abundance and activation analysis by Western blot. (**K**, **L**) MDA-MB-435 cells pretreated with NAC (3 mM) for 30 min followed by CPYPP treatment for 24 h were used for PKR abundance and activation (**K**) and interaction with CDK7 by co-immunoprecipitation (**L**)

Integrated stress response (ISR) downregulates the translation process by phosphorylating eIF2α to alleviate proteostatic stress and support cell survival [37]. Interventions with PKRi (PKR inhibitor), GCN2-IN-1 (GCN2 inhibitor), and trans-ISRIB (eIF2α phosphorylation inhibitor), but not GSK2656157 (PERK inhibitor), differentially repressed cell viability and enhanced CsA-induced cell death (Fig. 7A). All of the inhibitors failed to ameliorate cell paraptosis in the presence of CPYPP or curcumin (Fig. 7A). However, PKRi significantly abrogated CPYPP-induced vacuolization (Fig. 7B) and relieved the upregulation of ER stress and UPR proteins, particularly HSPs, as well as protein ubiquitination in nuclear fractions (Fig. 7C). Moreover, the interaction between CDK7 and PKR was also enhanced by CPYPP, as detected as early as 6 h into the treatment (Supplemental Fig. S13). PKRi attenuated CDK7/CDK9 activity and Rpb1 activation, and interrupted the interaction between PKR and CDK7/CDK9 (Fig. 7D–F).

CPYPP upregulated cytosolic and nuclear PKR abundances without a significant effect on PKR phosphorylation at T446 in both the cytosol and nuclear fractions (Fig. 7G and H). ActD and CHX, as well as THZ1 and LY2857785, alleviated the CPYPP-induced upregulation of cytosolic and nuclear PKR abundances (Fig. 7G and H). PKRi had no effects on CPYPP-induced nuclear proteasome activity, subunit accumulation, and cellular free cysteine-thiols, despite a slight increase in some of the subunits (Supplemental Fig. S14A–C), but it remarkably ameliorated cellular ROS levels and nuclear hydrogen peroxide generation (Fig. 7I and J). Furthermore, NAC attenuated the upregulation of PKR abundances induced by CPYPP and its interaction with CDK7 (Fig. 7K and L). Taken together, PKR is activated by CPYPP in a ROS- and CDK7/CDK9-dependent manner, and its interaction with the CDK7/CDK9–Rpb1 complex enhances paraptotic transcription, whereas cytosolic PKR primarily functions in stress-specific translational regulation for proteostasis. Functional PKR, likely in complex with CDK7/CDK9–Rpb1, serves as a source to elicit ROS generation, particularly within the nucleus.

### CPYPP, CsA, and curcumin induce paraptotic cell death and suppress tumor growth in xenograft mouse models

**Fig. 8:**
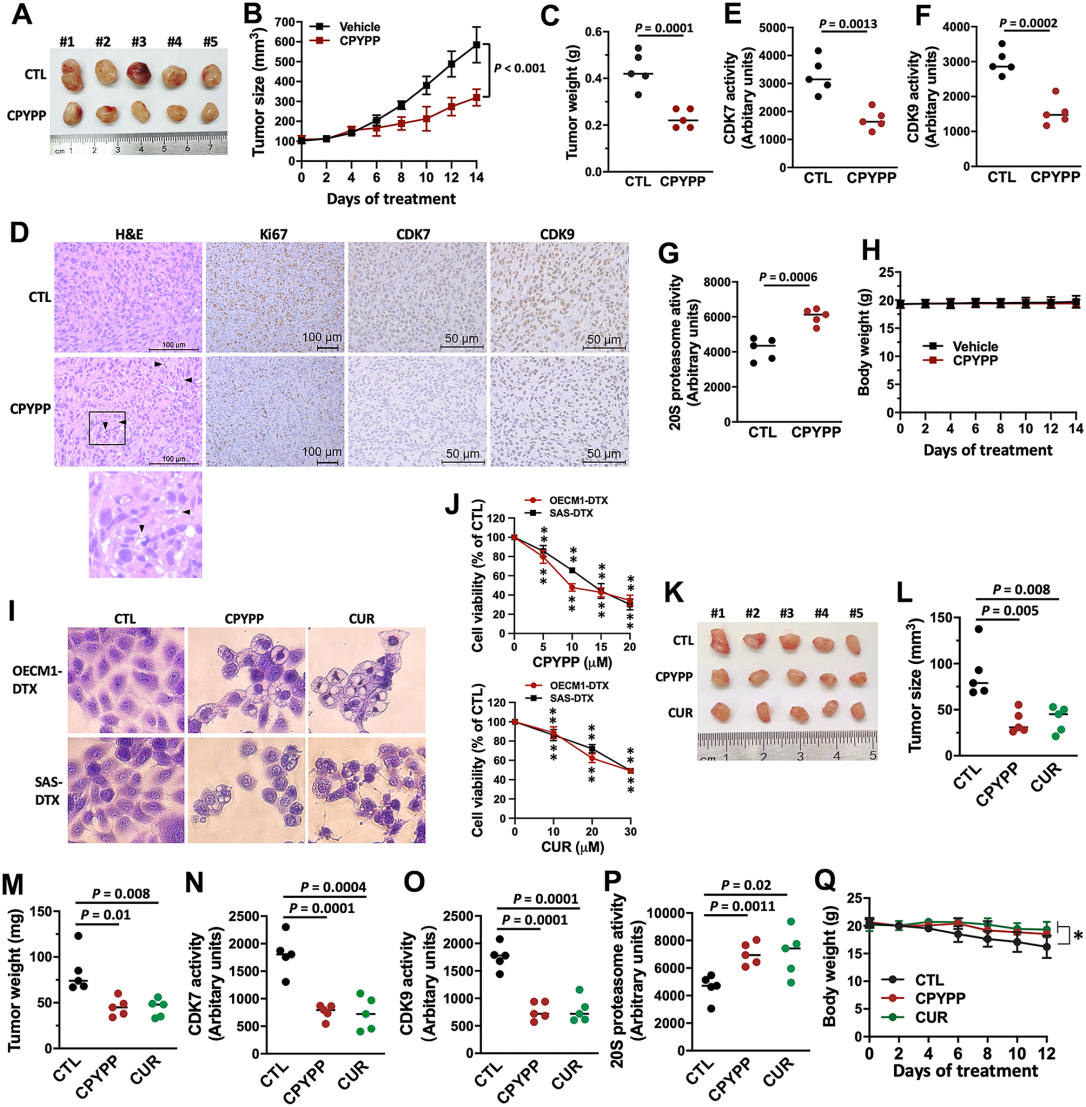
CPYPP and curcumin-induced paraptosis suppress tumor growth in xenograft mice. (**A**–**H)** MDA-MB-231 breast cancer xenograft mouse models treated with vehicle (CTL) or CPYPP. *n* = 5. (**A**) Representative image of the tumor masses in mice treated with different regimens. (**B**, **C**) Tumor size and tumor weight. (**D**) Hematoxylin and eosin (H&E) staining, Ki67, CDK7, and CDK9 expression in tumor samples. (**E–G)** CDK7, CDK9, and 20S proteasome activities in tumor samples. (**H**) Changes in mouse body weight throughout the 14-day experimental period. (**I**, **J**) Docetaxel-resistant head and neck cancer cell lines, OECM1-DTX and SAS-DTX, were treated with indicated concentrations of CPYPP or curcumin (CUR) for 24 h, followed by morphological vacuolization assessment (**I**) and cell viability analysis (**J**). Results are expressed as the mean ± SD from three independent experiments. ** *P* < 0.01, compared to the control group (CTL). (**K**–**Q**) OECM1-DTX tumor xenograft mouse models treated with vehicle (CTL), CPYPP, or curcumin (CUR). (**K**) Representative images of the tumor masses in mice receiving different treatments. (**L**, **M**) Tumor volume and tumor weight at experiment endpoint. *n* = 5. (**N**–**P**) CDK7, CDK9, and 20S proteasome activities in tumor samples. (**Q**) Changes in mouse body weight throughout the 12-day experimental period

We next evaluated the efficacy of paraptosis induction as a potential cancer therapeutic strategy using the MDA-MB-231 breast cancer orthotopic mouse model. The results revealed remarkable tumor growth retardation during the treatment period (Fig. 8A–C). Hematoxylin and eosin (H&E) staining showed vacuolation in tumor samples from CPYPP-treated mice, indicating the occurrence of paraptosis. Ki67 staining of the tumor sections demonstrated strong antiproliferative activity (Fig. 8D). Furthermore, immunohistochemistry staining of tumors revealed lower levels of CDK7 and CDK9 in CPYPP-treated mice (Fig. 8D). Additionally, enzyme activities of CDK7, CDK9 were significantly inhibited in the tumors, whereas 20S proteasome activity was increased. (Fig. 8E–G). There was no significant change in body weight during the test period (Fig. 8H).

Chemotherapy resistance is a central issue in cancer treatment. To address this challenge, we utilized docetaxel-resistant head and neck cancer cells, OECM1-DTX, and SAS-DTX, to assess the induction of paraptosis as a potential strategy to overcome chemo-resistance. CPYPP and curcumin induced massive vacuolization and suppressed cell viability in docetaxel-resistant head and neck cancer cells, OECM1-DTX, and SAS-DTX (Fig. 8I and J). Furthermore, CPYPP and curcumin significantly suppressed tumor growth, tumor weight, CDK7, and CDK9 activities in an OECM1-DTX orthotopic mouse model (Fig. 8K–O), consistently with upregulated proteasome activity and sustained body weight (Fig. 8P and Q). These results suggest paraptosis induction as an effective approach for alternative treatment in chemo-resistant cancers.

## Discussion

Despite distinctive pharmacological mechanisms [10–12, 38, 39], CPYPP, CsA, and curcumin were shown for the first time to induce cancer cell paraptosis in a ROS- and CDK7/CDK9-dependent manner. The finding that CDK7/CDK9 interacts with HSPs to arrest the cell cycle at the G2/M phase in paraptotic development extends the role of CDKs to the upstream step in cell cycle progression [19, 40, 41]. HSPs may typically act as chaperones to escort the complex. Upregulation of HSP70 by ML346 induced cell paraptosis in a CDK7/CDK9-dependent manner, while selective inhibition of HSP70 by 2-phenylethynesulfonamide impaired the function of associated chaperones and resulted in cell paraptosis in several cancer cell lines [42]. HSPs, therefore, may serve as functional associated activators with the CDK7/CDK9–Rpb1 complex, akin to TLR4 activation in tumor-induced muscle wasting [43]. The initial upregulation of HSPs and UPS thus reflects an adaptive response to ER stress for protein quality control. As nuclear stress increases, HSPs are recruited into the nucleus, where they interact with CDK7/CDK9 to amplify paraptotic transcription in a forward loop. However, single HSP70 knockdown failed to alter paraptotic vacuolization and ER stress (data not shown), suggesting that the activity of the CDK7/CDK9–Rpb1 complex is regulated stoichiometrically and synergistically by various members of the HSP family [41].

Under prolonged activation and severe stress, ISR signaling can shift to programmed cell death [37, 44]. The blockade of CDK7/CDK9 activity alleviated paraptotic cell death, and inhibition of PKR and HSP70 suppressed CDK activity, relieved cytoplasmic vacuolization, but failed to rescue cell viability. The interaction of HSPs and PKR with CDK7/CDK9–Rpb1 functions critically in paraptotic transcription, exacerbating proteostatic stress in the ER and leading to vacuolization morphology, while they may independently mediate other pro-death programs [30, 37]. Dramatic remodeling of microtubules contributes to vacuolation morphology and cell shrinkage during paraptotic cell death [45]. ISR operations have been shown to enhance centrosomal protein syntheses to remodel microtubule dynamics and concentrate unfolded proteins for perinuclear degradation [46]. P58IPK (inhibitor of the PKR protein kinase) has been shown to bring HSP40–HSP70/HSC70 (heat-shock cognate 70) to PKR to form a trimeric complex that stimulates the ATPase activity of HSP70/HSC70 [43]. Accordingly, PKR may act on HSP activity to facilitate CDK7/CDK9–Rpb1 functions, while PKR likely interacts with CDK7/CDK9–Rpb1, even provoking ROS generation within the nucleus. Details of the CDK7/CDK9–Rpb1 complex interacting with HSPs and PKR against impeded redox and proteotoxicity in the nucleus and the origins contributing to ROS production, such as nuclear lipoxygenase [47], cytochrome oxidase, and the electron transport system on nuclear membranes [48], remain to be investigated. Despite upregulated activity, the phosphorylation sites for the activation of CDK9 (T186) [49] were not altered in this study. Additional phosphorylation sites and post-translational modifications on CDK7/CDK9 themselves or on their complexed effectors, such as MAT1, cyclin H, TFIIH, and cyclin T1, require further investigation. Interestingly, the in vivo xenograft study revealed that both the expression and enzyme activity of CDK7 and CDK9 were significantly inhibited following long-term treatment with CPYPP or curcumin, suggesting the existence of a negative feedback loop in the regulation of CDK7/CDK9.

Our results showed that 20S proteasome activity was persistently enhanced by the paraptotic stimuli, reflecting an adaptive response to maintain proteostasis. However, it ultimately failed to handle the redundant protein generation due to the dysregulation of E3 in the UPS. Curcumin has been shown to inhibit proteasome activity and induce cancer cell paraptosis [50, 51]. Its modulation of proteasome activity is employed in a biphasic manner [52], with initial enhancement of proteasome activity followed by a decline, as observed in this study. The inconsistency may be attributed to differences in the duration of stimulation time and dosage.

Intracellular proteasome traffic is dynamically regulated; aberrant nuclear proteins can be targeted to cytosolic proteasomes for degradation or by nuclear proteasomes, primarily via nucleus-localized CHIP, a chaperone-dependent E3 ligase [53, 54]. Accumulation of nuclear HSP70 and BAG3 may facilitate CHIP activity for unfolded protein clearance. Rather surprisingly, CPYPP-induced ER stress and UPR, particularly PERK1, IRE1α, BiP, GADD34, and TRIB3 kinase, as well as UPS and HSPs, were mainly localized in the nucleus, suggesting an enhanced transnuclear process of unfolded proteins by nuclear proteasome degradation. Alternatively, this also suggests in situ proteotoxic stress to recruit the effectors into the nucleus. Inhibition of eIF2α phosphorylation by trans-ISRIB and protein synthesis by CHX has been shown to reverse the accumulation of stress granules induced by arsenite but fails in nuclear stress foci formation [55].

The primarily nuclear accumulation of proteasomes in proliferating cells reflects the majority of proteasomal substrates within the nucleus, and their timely and in situ degradation can facilitate the progression of the cell cycle [56]. We have clearly demonstrated that paraptosis progresses with impeded proteostasis primarily in the nucleus, as evidenced by the accumulation of hydrogen peroxide, HSPs, and UPS. Interestingly, the accumulation of nuclear proteasomes was not altered by ivermectin and leptomycin B. Importin/exportin-independent processes for nucleo-translocation, such as AKIRIN2-mediated nuclear import of proteasomes [57], transportin-mediated pathways, or through the dynamics of the nuclear pore complex (NPC) [58], and even by the VPS4 (vacuolar protein sorting 4)-dependent vacuolar clearance of nuclear and cytoplasmic unfolded proteins at the juxtanuclear junctions [59], may mediate the import of proteasomes and subunits to maintain nuclear proteostasis. Nuclear proteasomes have been localized to tether via their Rpn9 subunits with the NPC [60]. Under conditions of amino acid starvation, disassembled ribosomal proteins and proteasome subunits, as well as inhibition of perinuclear proteasomes and their nuclear export, and even under conditions of hyperosmotic stress, heat stress, and oxidative stress, polyubiquitinated proteins are recruited to nuclear foci for degradation [56]. The massive accumulation of perinuclear aggresomes induced by CPYPP may suggest a two-way traffic of cytoplasmic and nuclear proteasomes for the clearance of unfolded proteins.

**Fig. 9:**
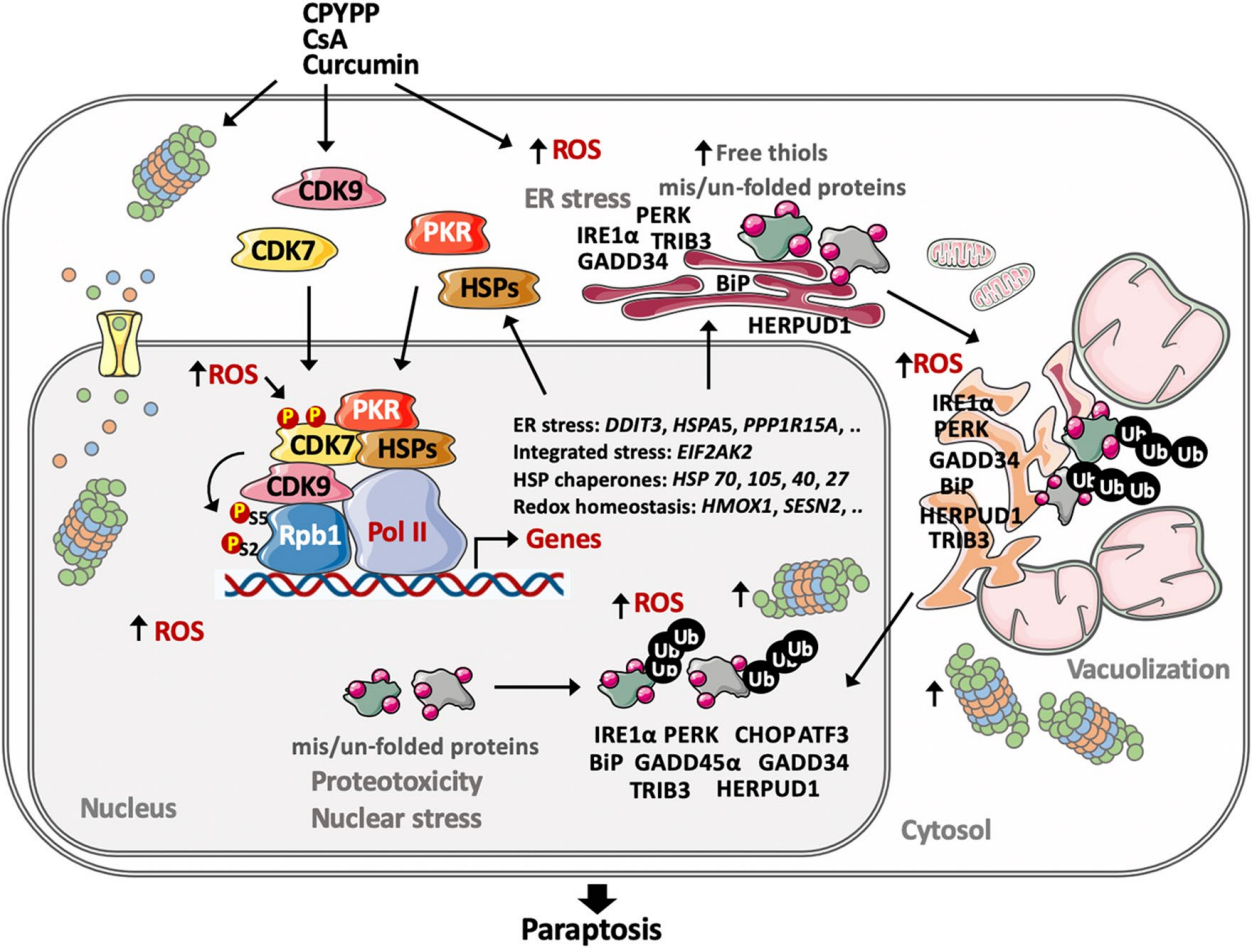
The proposed mechanism of paraptotic progression. Parts of the figure were created using images from Servier Medical Art, which is licensed under a Creative Commons Attribution 4.0 Unported License (https://creativecommons.org/licenses/by/4.0/)

In summary, CPYPP, CsA, and CUR induce paraptotic death in cancer cells, commonly characterized by massive vacuolization, ROS provocation, and transcriptional activation for protein synthesis involved in redox homeostasis and proteostasis, including ER stress, UPR, HSP chaperones, and the ubiquitin-proteasome system (UPS). CDK7/CDK9 activation is required for the transcriptional regulation to drive the paraptotic program. Paraptosis evolves with ROS provocation and activation of proteostatic dynamics in response to ER stress for protein quality control. Impaired redox status and proteotoxicity occur as ROS are constantly provoked, and HSPs and UPS, as well as ER and UPR proteins, accumulate particularly within the nucleus, thus activating CDK7/CDK9 to prime stress-specific transcription. The interaction of CDK7/CDK9–Rpb1 with downstream HSPs, and PKR potentiates transcriptional activation. As functional CDK7/CDK9–Rpb1–HSPs–PKR proceeds, CDK7/CDK9-dependent ROS provocation further impedes nuclear redox status and proteostasis, thereby amplifying transcriptional regulation in a forward regulatory loop and reciprocally-regulated manner. This, in turn, exacerbates cellular redox status and proteotoxicity, leading to the failure of ERAD, cytoplasmic vacuolization, and ultimately inducing cell paraptosis (Fig. 9). For the first time, we report that activation of CDK7/CDK9–Rpb1 by nuclear stress mediates transcriptional regulation to prime paraptosis in cancer cells. The induction of paraptosis is also operative in orthotopic mouse models of breast cancer and chemo-resistant head and neck cancer cells.

The heterogeneity within tumors poses significant challenges in cancer treatment, as it can result in differences in treatment response, disease progression, and the emergence of drug-resistant clones [61, 62]. Inducing different forms of cell death in a heterogeneous cancer cell population may require diverse strategies to activate alternate cell death pathways in different cancer cells. Our study has shown that inducing paraptosis could be a practical strategy for treating cancer, particularly in cases of primary or chemotherapy-resistant cancer. Paraptosis offers a potential alternative cell death pathway that could overcome the resistance mechanisms developed by cancer cells. By targeting paraptosis induction, we may be able to effectively combat the heterogeneity of cancer and improve treatment outcomes.

## Materials and methods

### Reagents

CPYPP (#4568) and ivermectin (#1260) were purchased from Tocris Bioscience (Cat. No. 4568, Ellisville, MO, USA). Actinomycin D (#11421), CPYPP (#17386), cycloheximide (#14126), cyclosporin A (#12088), THZ1 (#9002215), liproxstatin-1 (#17730), LY2857785 (#34880), necrostatin-1 (#11658), z-VAD(OMe)-FMK (#14463), Ac-YVAD-CHO (#10016), MKT-077 (#14395), 3-methyladenine (#13242), bafilomycin A1 (#11038), chloroquine (#14194), curcumin (#81025), ML346 (#23844), PKR inhibitor (#15323), GCN2-IN-1 (# 36027), *trans*-ISRIB (#16258), and GSK2656157 (#17372) were obtained from Cayman Chemicals (Ann Arbor, MI, USA). *N*-acetyl-L-cysteine (sc-202232), MCC950 (sc-505904), and leptomycin B (sc-358688) were obtained from Santa Cruz Biotechnology (CA, USA). DMEM/F12 medium, RPMI-1640 medium, DMEM medium, MEM medium, fetal bovine serum (FBS), and penicillin/streptomycin were purchased from ThermoFisher Scientific Inc. (Pittsburgh, PA, USA). Antibodies used in this study were listed in Supplemental Table S2. Other reagents were purchased from Sigma-Aldrich (St. Louis, MO, USA).

### Cell lines and cell cultures

The human breast cancer cell line MDA-MB-231 and human glioblastoma cell lines DBTRG-05MG and U-87 MG were obtained from the Bioresource Collection Research Center (Hsinchu, Taiwan). The human melanoma cell line MDA-MB-435, breast epithelial cell line MCF 10A, pancreatic cancer cell line PANC-1, and lung adenocarcinoma cell line NCI-H1299 were acquired from the American Type Culture Collection (Manassas, VA, USA). The human oral squamous carcinoma cell line OECM-1 was purchased from MilliporeSigma, and the human tongue squamous cell carcinoma cell line SAS was obtained from the Japanese Collection Research Bioresources.

MDA-MB-231 and MDA-MB-435 cells were cultured in DMEM/F12 medium supplemented with 10% (v/v) FBS, 100 U/ml penicillin, and 100 µg/ml streptomycin. U-87 MG cells were cultured in MEM medium containing 10% FBS, 2 mM L-glutamine, 0.1 mM non-essential amino acids, and 1 mM sodium pyruvate. DBTRG-05MG, NCI-H1299, and OECM-1 were maintained in RPMI-1640 medium supplemented with 10% FBS. PANC-1 and SAS cells were maintained in DMEM medium containing 10% FBS. MCF 10 A cells were cultured in DMEM/F12 medium supplemented with 5% horse serum, 20 ng/ml EGF, 0.5 µg/ml hydrocortisone, 10 µg/ml insulin, 1% NEAA (non-essential amino acids), 100 U/ml penicillin, and 100 µg/ml streptomycin. Docetaxel-resistant OECM-1 and SAS cells were maintained in culture medium supplemented with 0.4 nM docetaxel (#11637, Cayman). All cells were maintained at 37 °C in a humidified incubator containing 5% CO_2_.

### Cell viability, proliferation rate, and morphology observation

Cell viability was assessed using the MTT staining method. Cells were cultured in 96-well plates with the specified compounds for the indicated durations. After treatment, cells were incubated with Thiazolyl blue tetrazolium bromide (MTT, 0.5 mg/ml, Sigma, Cat# M5655) for an additional 3 h. The purple formazan crystals were dissolved in DMSO, and the absorbance was measured at 570 nm and 630 nm.

CFDA-SE (Cayman, Cat# 14456), a cell-permeable dye, was used to trace the daughter cells following the cell division for proliferation rate. Cells were cultured in growth medium containing 0.5 µM CFDA-SE and then subjected to various compound stimulations. After 24 h, cells were harvested for fluorescence determination using flow cytometry (BD FACSVerse, Becton Dickinson Inc., San Jose, CA, USA). Data analysis was performed using CellQuest software (BD Biosciences, Franklin Lakes, NJ, USA).

For morphological observations, cells were stained with a crystal violet solution (0.5%, w/v, in 30% ethanol) for 20 min, followed by a wash with H_2_O. Morphological alterations were visualized and photographed using a phase-contrast microscope (Leica Microsystems, Wetzlar, Germany).

### Colony formation assay

Five hundred cells per well were seeded onto 6-well plates and maintained at 37 °C in an incubator with 5% CO_2_. Cells were treated with different compounds for 24 h and then cultured in normal culture medium for 10 days. Colonies were subsequently fixed and stained with a crystal violet solution (0.5%, w/v, in 30% ethanol). Colony formation was visualized and quantified spectrophotometrically at 595 nm after dissolution in 1% SDS.

### Immunofluorescence staining for confocal microscope

Cells were fixed using 3.7% (w/v) formaldehyde and then permeabilized with 0.3% (v/v) Triton X-100. After blocking with 2% (w/v) bovine serum albumin in phosphate-buffered saline (PBS), cells were stained with specific antibodies followed by Alexa Fluor™ 488- or Alexa Fluor™ 594-conjugated secondary antibodies (ThermoFisher Scientific). Coverslips were mounted using Fluoroshield Mounting Medium with DAPI (Abcam), and the fluorescence images were captured on a Leica Microsystems TCS SP8 Confocal Spectral microscope (Leica Microsystems, Wetzlar, Germany).

Aggresome accumulation was detected using the PROTEOSTAT™ Aggresome Detection Kit (#51035, Enzo Life Sciences, Farmingdale, NY, USA) following the manufacturer’s instructions. Briefly, cells were fixed with 4% paraformaldehyde and permeabilized with 0.5% Triton X-100. Cells were then incubated with the Proteostat Aggresome dye for 1.5 h, and the fluorescence images were captured using a confocal microscope.

### Next-generation sequencing analysis (NGS)

NGS was employed to profile the differentially expressed genes (DEGs) induced by CPYPP in MDA-MB-231 and MDA-MB-435 cells. RNA sequencing cluster generation and high-throughput sequencing were conducted by AZENTA Life Science (Chelmsford, MA, USA) following the manufacturer’s instructions. The acquired raw sequencing data underwent sequence quality control, read trimming, alignment, expression quantification, differential expression analysis, and functional enrichment. Genes were considered differentially expressed if the fold change value was greater than 2 and the false discovery rate (FDR, q-value) was less than 0.05. Pathway annotation analysis was carried out using the Kyoto Encyclopedia of Genes and Genomes (KEGG) database with the DAVID/EASE tool. All pathways obtained in the transcriptome analysis are organism-specific pathways. The NGS data have been deposited in the Gene Expression Omnibus (GEO) with the GEO Accession number GSE262661.

### Proteomic identification

Proteomic alterations in CPYPP-treated MDA-MB-435 cancer cells were assessed by mass spectrometric analysis. Total proteins were separated using SDS-PAGE and subjected to in-gel digestion to produce tryptic peptides. Mass spectrometric analysis was performed using an Orbitrap Fusion mass spectrometer (ThermoFisher Scientific, Waltham, MA, USA) equipped with the Ultimate 3000 RSLC system (Dionex) and a nano-electrospray ion source (New Objective). Protein identification and label-free quantification were analyzed using the computational platform Proteome Discoverer (v2.4). LC-MS data has been deposited into the ProteomeXchange Consortium via the PRIDE partner repository with the jPOST ID: JPST002971 and project accession PXD050304.

### Measurement of protein thiol contents and reactive oxygen species levels

The free cysteine-thiol groups of proteins were determined by labeling with Tetraphenylethene maleimide (TPE-MI, # HY-143218, MedChemExpress, Monmouth Junction, NJ, USA), presenting the unfolded protein load and proteostasis in cells [63]. Cytosolic and nuclear ROS were detected using fluorescent dyes, 2’,7’-dichlorodihydrofluorescein diacetate (DCF-DA, #85155, Cayman, Ann Arbor, MI, USA), and NucPE1 (MedChemExpress, HY-101859), respectively. After treatment, cells were washed with PBS three times and stained with fluorescent dyes (final concentrations: TPE-MI, 20 µM; DCF-DA, 2 µM; NucPE1, 2 µM) for 30 min at 37 °C and then quantified for intracellular fluorescence by flow cytometry.

### CDK7 and CDK9 activity assay

Cells or mouse tumor samples were lysed in assay buffer and quantified for total protein concentrations. Ten micrograms of proteins were used for CDK7 and CDK9 activity determination following the instructions enclosed in the kit (CDK7 Assay Kit, Cat# 79603; CDK9 Assay Kit, Cat# 79628, BPS Bioscience, San Diego, CA, USA).

### Measurement of 20S proteasome activity

Cells were lysed in lysis buffer (50 mM Tris-HCl, pH 7.5, 150 mM NaCl, 1 mM EDTA, 10% Glycerol, 0.5% Nonidet P-40, and 1 mM MgCl_2_) and incubated on ice for 30 min, followed by centrifugation at 15,000 g for 20 min to collect the supernatants for proteasome activity analysis. Ten micrograms of supernatant proteins were incubated with Suc-Leu-Leu-Val-Tyr-AMC (final concentration 50 µM, Cayman, Cat#10008119) in the assay buffer (50 mM Tris-HCl, pH 7.5, 150 mM NaCl, 5 mM MgCl_2_, 1 mM ATP) at 37 °C for 1 h, and thereafter the proteasomal activity was measured at excitation/emission wavelengths of 360/460 nm. For cytosolic and nuclear 20 S proteasome activity, cells were fractionated and suspended in the lysis buffer for cytosolic and nuclear 20S proteasome activity assays.

### Quantitative real-time reverse transcription PCR (qRT-PCR)

Total RNA was isolated using TRIzol™ Reagent (ThermoFisher). Five micrograms of total RNA were used for cDNA strand synthesis using the M-MLV reverse transcriptase kit (ThermoFisher). qRT-PCR analysis was performed with iQ™ SYBR® Green Supermix (Bio-Rad Laboratories) and a LightCycler® 480 II RT-PCR system (Roche Applied Sciences, Mannheim, Germany). Primer information is provided in the Supplemental Table S3. The expression levels of mRNA were normalized to β-actin mRNA (ACTB) in the same samples.

### Gene knockdown by shRNA

The specific short hairpin PLKO.1, pCMV-ΔR8.91, and pMD.G plasmids were purchased from the National RNAi Core Facility, Academia Sinica (Taiwan). The shRNA clones used in this study are described in the Supplemental Table S4. Specific shRNA and packaging vectors (pCMV-ΔR8.91 and pMD.G) were transiently transfected into the host 293T cells using Lipofectamine 2000 transfection reagent (Invitrogen). Two days after transduction, lentiviral particles in the culture medium were used to transduce the target cells. Twenty-four hours after infection, the medium was replaced with normal medium supplemented with 2 µg/ml of puromycin. Three days after knockdown, the cells were used for further studies.

#### Western blot analysis

Harvested cells were sonicated in PBS containing protease inhibitors (1 mM PMSF, 10 µg/ml each of leupeptin, aprotinin, and pepstatin A) and phosphatase inhibitors (1 mM Na_3_VO_4_, 1 mM NaF). Equal amounts of lysates were subjected to SDS-PAGE gels and transferred onto FluoroTrans® PVDF Transfer Membranes (Pall Corporation). After blocking with 5% skim milk (in Tris-buffered saline with 0.1% Tween 20), membranes were further incubated with the primary antibodies overnight and then detected with horseradish peroxidase-conjugated secondary antibodies (EMD Millipore). The signal was captured by the Clarity™ Western ECL Substrate (Bio-Rad) and quantified using the ChemiDoc™ Touch Imaging System and Image Lab Software (Bio-Rad).

### Isolation of cytosol and nuclear proteins

After trypsinization, cells were gently suspended in the hypotonic buffer (10 mM HEPES, pH 7.9, 1.5 mM MgCl_2_, 0.1 mM EDTA, 0.1 mM EGTA, proteinase inhibitors, and phosphatase inhibitors) and kept on ice for 15 min. After the addition of 0.5% NP-40, cell suspensions were vortexed vigorously for 10 s to lyse cells. The cell lysate mixtures were further centrifuged at 10,000 × *g* for 2 min at 4 °C to separate the cytoplasmic (supernatants) and nuclei fractions (pellets). The nuclear pellets were washed with hypotonic buffer three times to purify the nuclei and then suspended in PBS containing proteinase inhibitors and phosphatase inhibitors. The suspensions were further sonicated and quantified for protein concentration for Western blot and 20 S proteasome activity analysis.

#### Co-immunoprecipitation of proteins

Whole cell lysates or cytosol and nuclear fractions were suspended in RIPA buffer (5 mM Tris-HCl, pH 7.4, 150 mM NaCl, 1% NP-40, 1 mM EDTA, 1% sodium deoxycholate, proteinase inhibitors, and phosphatase inhibitors). One milligram of proteins was incubated with specific primary antibodies and pulled down by SureBeads™ Protein G Magnetic Bead (Bio-Rad). After washing, the immunoprecipitated proteins were subjected to SDS-PAGE and Western blot analysis.

#### Proximity ligation assay

The protein-protein interaction was assessed using the Duolink® Proximity Ligation Assay (PLA) kit (Cat# DUO92101, Sigma-Aldrich) following the manufacturer’s instructions. Briefly, MDA-MB-435 cells were cultured on coverslips. After treatment, cells were fixed with 4% formaldehyde-PBS and then permeabilized using 0.3% Triton-X 100-PBS. Cells were incubated with Duolink blocking solution at 37 °C for 60 min, followed by incubation with the two antibodies used for the interaction study at 4 °C overnight. Then, the slides were further processed with Duolink probe incubation, ligation, and amplification. The resulting signals were captured in photomicrographs using a Confocal Spectral microscope.

#### Mouse models of orthotopic breast cancer and head and neck cancer

The animal procedures (CMUIACUC-2020-096 and CMUIACUC-2021-150) of this study were approved by the Institutional Animal Care and Use Committee at China Medical University. For the orthotopic breast cancer study, five-week-old female SCID (C.B17/lcr-Prkdc/CrlNarl) mice (BioLASCO Taiwan Co., Ltd) were inoculated with MDA-MB-231 breast cancer cells (2 × 10^5^ cells in 100 µl of 50% Matrigel (CORNING Life Science, #354,230)-PBS) at the mammary gland. After 10 days, mice were randomly assigned to two groups for treatment: (1) control and (2) CPYPP groups, each consisting of five mice. For the head and neck cancer study, five-week-old male SCID mice (BioLASCO Taiwan Co., Ltd) were inoculated with docetaxel-resistant OECM-1 cells (2 × 10^5^ cells in 20 µl PBS) at the lateral border of the tongue. After 7 days, mice were randomly assigned to three groups for treatment: (1) control group; (2) CPYPP group; and (3) curcumin group, each consisting of five mice. Mice received either the vehicle (10% DMSO, 40% PEG300, 5% Tween-80, 45% saline), 30 mg/kg CPYPP, or 50 mg/kg curcumin by intraperitoneal injection daily. Mice were sacrificed, and tumors were excised for tumor size detection, hematoxylin and eosin (H&E) staining, immunohistochemistry staining, and enzyme activity analysis, including CDK7, CDK9, and 20S proteasome activity. Tumor volume was calculated using the formula: V = 1/2 × (length × width^2^). Mouse body weight was measured every 2 days.

### Statistical analysis

All data are expressed as the means ± SD of three to five independent experiments. The significance of differences was analyzed by two-tailed Student’s *t*-tests or two-way ANOVA followed by Tukey’s post hoc test. *P* values < 0.05 were considered statistically significant.

### Electronic supplementary material

Below is the link to the electronic supplementary material.

Supplementary Material 1

Supplementary Material 2

## Data Availability

The datasets used and/or analyzed during the current study are available from the corresponding author upon reasonable request.

## Abbreviations

ActD: Actinomycin D
CDK: Cyclin-dependent kinase
CHX: Cycloheximide
CPYPP: 4-[3-(2-Chlorophenyl)-2-propen-1-ylidene]-1-phenyl-3,5-pyrazolidinedione
CsA: Cyclosporin A
DGEs: Differential gene expressions
ER: Endoplasmic reticulum
ERAD: Endoplasmic reticulum-associated protein degradation
FBS: Fetal bovine serum
HSP: Heat shock protein
ISR: Integrated stress response
NAC: *N*-acetyl-L-cysteine
PBS: Phosphate-buffered saline
PDI: Protein disulfide isomerase
PKR: Protein kinase R
ROS: Reactive oxygenase species
Rpb1: RNAPII subunit B1
UPR: Unfolded protein response
UPS: Ubiquitin-proteasome system

## References

[1] Sperandio S, de Belle I, Bredesen DE (2000). An alternative, nonapoptotic form of programmed cell death. Proc Natl Acad Sci USA.

[2] Shubin AV, Demidyuk IV, Komissarov AA, Rafieva LM, Kostrov SV (2016). Cytoplasmic vacuolization in cell death and survival. Oncotarget.

[3] Fricker M, Tolkovsky AM, Borutaite V, Coleman M, Brown GC (2018). Neuronal cell death. Physiol Rev.

[4] Monel B, Compton AA, Bruel T, Amraoui S, Burlaud-Gaillard J, Roy N (2017). Zika virus induces massive cytoplasmic vacuolization and paraptosis-like death in infected cells. EMBO J.

[5] Wang Y, Li X, Wang L, Ding P, Zhang Y, Han W (2004). An alternative form of paraptosis-like cell death, triggered by TAJ/TROY and enhanced by PDCD5 overexpression. J Cell Sci.

[6] Yoon MJ, Kang YJ, Lee JA, Kim IY, Kim MA, Lee YS (2014). Stronger proteasomal inhibition and higher CHOP induction are responsible for more effective induction of paraptosis by dimethoxycurcumin than curcumin. Cell Death Dis.

[7] Ghosh K, De S, Das S, Mukherjee S, Sengupta Bandyopadhyay S (2016). Withaferin A induces ROS-mediated paraptosis in human breast cancer cell-lines MCF-7 and MDA-MB-231. PLoS ONE.

[8] Fontana F, Raimondi M, Marzagalli M, Di Domizio A, Limonta P (2020). The emerging role of paraptosis in tumor cell biology: perspectives for cancer prevention and therapy with natural compounds. Biochim Biophys Acta Rev Cancer.

[9] Wang Y, Wen X, Zhang N, Wang L, Hao D, Jiang X (2019). Small-molecule compounds target paraptosis to improve cancer therapy. Biomed Pharmacother.

[10] Lee D, Kim IY, Saha S, Choi KS (2016). Paraptosis in the anti-cancer arsenal of natural products. Pharmacol Ther.

[11] Sperandio S, Poksay K, de Belle I, Lafuente MJ, Liu B, Nasir J (2004). Paraptosis: mediation by MAP kinases and inhibition by AIP-1/Alix. Cell Death Differ.

[12] Bury M, Girault A, Mégalizzi V, Spiegl-Kreinecker S, Mathieu V, Berger W (2013). Ophiobolin A induces paraptosis-like cell death in human glioblastoma cells by decreasing BKCa channel activity. Cell Death Dis.

[13] Yoon MJ, Lee AR, Jeong SA, Kim YS, Kim JY, Kwon YJ (2014). Release of Ca^2+^ from the endoplasmic reticulum and its subsequent influx into mitochondria trigger celastrol-induced paraptosis in cancer cells. Oncotarget.

[14] Binoy A, Nedungadi D, Katiyar N, Bose C, Shankarappa SA, Nair BG (2019). Plumbagin induces paraptosis in cancer cells by disrupting the sulfhydryl homeostasis and proteasomal function. Chem Biol Interact.

[15] Hager S, Korbula K, Bielec B, Grusch M, Pirker C, Schosserer M (2018). The thiosemicarbazone Me2NNMe2 induces paraptosis by disrupting the ER thiol redox homeostasis based on protein disulfide isomerase inhibition. Cell Death Dis.

[16] Nedungadi D, Binoy A, Pandurangan N, Nair BG, Mishra N (2021). Proteasomal dysfunction and ER stress triggers 2’-hydroxy-retrochalcone-induced paraptosis in cancer cells. Cell Biol Int.

[17] Han J, Back SH, Hur J, Lin YH, Gildersleeve R, Shan J (2013). ER-stress-induced transcriptional regulation increases protein synthesis leading to cell death. Nat Cell Biol.

[18] Ram BM, Ramakrishna G (2014). Endoplasmic reticulum vacuolation and unfolded protein response leading to paraptosis like cell death in cyclosporine A treated cancer cervix cells is mediated by cyclophilin B inhibition. Biochim Biophys Acta.

[19] Ganuza M, Sáiz-Ladera C, Cañamero M, Gómez G, Schneider R, Blasco MA (2012). Genetic inactivation of Cdk7 leads to cell cycle arrest and induces premature aging due to adult stem cell exhaustion. EMBO J.

[20] Fisher RP (2019). Cdk7: a kinase at the core of transcription and in the crosshairs of cancer drug discovery. Transcription.

[21] Kim M, Suh H, Cho EJ, Buratowski S (2009). Phosphorylation of the yeast Rpb1 C-terminal domain at serines 2, 5, and 7. J Biol Chem.

[22] Shapiro GI (2006). Cyclin-dependent kinase pathways as targets for cancer treatment. J Clin Oncol.

[23] Sava GP, Fan H, Coombes RC, Buluwela L, Ali S (2020). CDK7 inhibitors as anticancer drugs. Cancer Metastasis Rev.

[24] Nishikimi A, Uruno T, Duan X, Cao Q, Okamura Y, Saitoh T (2012). Blockade of inflammatory responses by a small-molecule inhibitor of the rac activator DOCK2. Chem Biol.

[25] Laurin M, Huber J, Pelletier A, Houalla T, Park M, Fukui Y (2013). Rac-specific guanine nucleotide exchange factor DOCK1 is a critical regulator of HER2-mediated breast cancer metastasis. Proc Natl Acad Sci USA.

[26] Ferrari MG, Ganaie AA, Shabenah A, Mansini AP, Wang L, Murugan P (2020). Identifying and treating ROBO1^-ve^ /DOCK1^+ ve^ prostate cancer: an aggressive cancer subtype prevalent in African American patients. Prostate.

[27] Liu XF, Xiang L, Zhou Q, Carralot JP, Prunotto M, Niederfellner G (2016). Actinomycin D enhances killing of cancer cells by immunotoxin RG7787 through activation of the extrinsic pathway of apoptosis. Proc Natl Acad Sci USA.

[28] Guan BJ, van Hoef V, Jobava R, Elroy-Stein O, Valasek LS, Cargnello M (2017). A unique ISR program determines cellular responses to chronic stress. Mol Cell.

[29] Chou J, Quigley DA, Robinson TM, Feng FY, Ashworth A (2020). Transcription-associated cyclin-dependent kinases as targets and biomarkers for cancer therapy. Cancer Discov.

[30] Parua PK, Fisher RP (2020). Dissecting the PolII transcription cycle and derailing cancer with CDK inhibitors. Nat Chem Biol.

[31] Rousaki A, Miyata Y, Jinwal UK, Dickey CA, Gestwicki JE, Zuiderweg ER (2011). Allosteric drugs: the interaction of antitumor compound MKT-077 with human Hsp70 chaperones. J Mol Biol.

[32] Calamini B, Silva MC, Madoux F, Hutt DM, Khanna S, Chalfant MA et al. ML346: a novel modulator of proteostasis for protein conformational diseases. 2012 Dec 17 [updated 2013 Apr 5]. In: Probe reports from the NIH molecular libraries program [Internet]. Bethesda (MD): National Center for Biotechnology Information (US). 23833797

[33] Chatterjee S, Burns TF (2017). Targeting heat shock proteins in cancer: a promising therapeutic approach. Int J Mol Sci.

[34] Wang Z, Fukushima H, Inuzuka H, Wan L, Liu P, Gao D (2012). Skp2 is a promising therapeutic target in breast cancer. Front Oncol.

[35] Sharma P, Nag A (2014). CUL4A ubiquitin ligase: a promising drug target for cancer and other human diseases. Open Biol.

[36] Korovila I, Hugo M, Castro JP, Weber D, Höhn A, Grune T, Jung T (2017). Proteostasis, oxidative stress and aging. Redox Biol.

[37] Tian X, Zhang S, Zhou L, Seyhan AA, Hernandez Borrero L, Zhang Y (2021). Targeting the integrated stress response in cancer therapy. Front Pharmacol.

[38] Larasati YA, Yoneda-Kato N, Nakamae I, Yokoyama T, Meiyanto E, Kato JY (2018). Curcumin targets multiple enzymes involved in the ROS metabolic pathway to suppress tumor cell growth. Sci Rep.

[39] Ibrahim SRM, Abdallah HM, El-Halawany AM, Mohamed GA, Alhaddad AA, Samman WA (2022). Natural reno-protective agents against cyclosporine A-induced nephrotoxicity: an overview. Molecules.

[40] Genest O, Wickner S, Doyle SM (2019). Hsp90 and Hsp70 chaperones: collaborators in protein remodeling. J Biol Chem.

[41] Truman AW, Kristjansdottir K, Wolfgeher D, Hasin N, Polier S, Zhang H (2012). CDK-dependent Hsp70 phosphorylation controls G1 cyclin abundance and cell-cycle progression. Cell.

[42] Leu JI, Pimkina J, Frank A, Murphy ME, George DL (2009). A small molecule inhibitor of inducible heat shock protein 70. Mol Cell.

[43] Zhang G, Liu Z, Ding H, Zhou Y, Doan HA, Sin KWT (2017). Tumor induces muscle wasting in mice through releasing extracellular Hsp70 and Hsp90. Nat Commun.

[44] Hurwitz B, Guzzi N, Gola A, Fiore VF, Sendoel A, Nikolova M (2022). The integrated stress response remodels the microtubule-organizing center to clear unfolded proteins following proteotoxic stress. Elife.

[45] Sperandio S, Poksay KS, Schilling B, Crippen D, Gibson BW, Bredesen DE (2020). Identification of new modulators and protein alterations in non-apoptotic programmed cell death. J Cell Biochem.

[46] Melville MW, Tan SL, Wambach M, Song J, Morimoto RI, Katze MG (1999). The cellular inhibitor of the PKR protein kinase, P58(IPK), is an influenza virus-activated co-chaperone that modulates heat shock protein 70 activity. J Biol Chem.

[47] Thannickal VJ, Fanburg BL (2000). Reactive oxygen species in cell signaling. Am J Physiol Lung Cell Mol Physiol.

[48] Li JM, Shah AM (2004). Endothelial cell superoxide generation: regulation and relevance for cardiovascular pathophysiology. Am J Physiol Regul Integr Comp Physiol.

[49] Larochelle S, Amat R, Glover-Cutter K, Sansó M, Zhang C, Allen JJ (2012). Cyclin-dependent kinase control of the initiation-to-elongation switch of RNA polymerase II. Nat Struct Mol Biol.

[50] Yoon MJ, Kim EH, Lim JH, Kwon TK, Choi KS (2010). Superoxide anion and proteasomal dysfunction contribute to curcumin-induced paraptosis of malignant breast cancer cells. Free Radic Biol Med.

[51] Milacic V, Banerjee S, Landis-Piwowar KR, Sarkar FH, Majumdar AP, Dou QP (2008). Curcumin inhibits the proteasome activity in human colon cancer cells in vitro and in vivo. Cancer Res.

[52] Khan TK, You Y, Nelson TJ, Kundu S, Pramanik SK, Das J (2020). Modulation of proteasome activity by curcumin and didemethylcurcumin. J Biomol Struct Dyn.

[53] Muller P, Ruckova E, Halada P, Coates PJ, Hrstka R, Lane DP (2013). C-terminal phosphorylation of Hsp70 and Hsp90 regulates alternate binding to co-chaperones CHIP and HOP to determine cellular protein folding/degradation balances. Oncogene.

[54] Franic D, Zubcic K, Boban M (2021). Nuclear ubiquitin-proteasome pathways in proteostasis maintenance. Biomolecules.

[55] Do TQ, Gaudreau-Lapierre A, Palii CG, Resende VMF, Campuzano D, Aeschimann CS (2020). A nuclear stress pathway that parallels cytoplasmic stress granule formation. iScience.

[56] Enenkel C, Kang RW, Wilfling F, Ernst OP (2022). Intracellular localization of the proteasome in response to stress conditions. J Biol Chem.

[57] de Almeida M, Hinterndorfer M, Brunner H, Grishkovskaya I, Singh K, Schleiffer A (2021). AKIRIN2 controls the nuclear import of proteasomes in vertebrates. Nature.

[58] Tessier TM, Dodge MJ, Prusinkiewicz MA, Mymryk JS (2019). Viral appropriation: laying claim to host nuclear transport machinery. Cells.

[59] Sontag EM, Morales-Polanco F, Chen JH, McDermott G, Dolan PT, Gestaut D (2023). Nuclear and cytoplasmic spatial protein quality control is coordinated by nuclear-vacuolar junctions and perinuclear ESCRT. Nat Cell Biol.

[60] Albert S, Schaffer M, Beck F, Mosalaganti S, Asano S, Thomas HF (2017). Proteasomes tether to two distinct sites at the nuclear pore complex. Proc Natl Acad Sci USA.

[61] Sun XX, Yu Q (2015). Intra-tumor heterogeneity of cancer cells and its implications for cancer treatment. Acta Pharmacol Sin.

[62] Ramón Y, Cajal S, Sesé M, Capdevila C, Aasen T, De Mattos-Arruda L, Diaz-Cano SJ (2020). Clinical implications of intratumor heterogeneity: challenges and opportunities. J Mol Med (Berl).

[63] Chen MZ, Moily NS, Bridgford JL, Wood RJ, Radwan M, Smith TA (2017). A thiol probe for measuring unfolded protein load and proteostasis in cells. Nat Commun.

