# Supplementary material for: CDK7/CDK9 mediates transcriptional activation to prime paraptosis in cancer cells
Source: Cell Biosci. 2024 Jun 10;14:78. doi: 10.1186/s13578-024-01260-2 (PMC11163730; doi:10.1186/s13578-024-01260-2)
Supplement: Supplementary file 1 — Supplementary Material 1 [file 13578_2024_1260_MOESM1_ESM.pdf]

Supplemental information

CDK7/CDK9 mediates transcriptional activation to prime paraptosis in cancer cells

Shih-Kai Chiang [1], Wei-Chao Chang [2,3,4], Shuen-Ei Chen [1,5,6,7,*], and Ling-Chu Chang [2,3,4,*]

[1]Department of Animal Science, National Chung Hsing University, Taichung 40227, Taiwan

[2]Center for Molecular Medicine, China Medical University Hospital, Taichung 406040, Taiwan

[3]Research Center for Cancer Biology, China Medical University, Taichung 406040, Taiwan

[4]Cancer Biology and Precision Therapeutics Center, China Medical University, Taichung 406040, Taiwan

[5]The iEGG and Animal Biotechnology Center, National Chung Hsing University, Taichung 40227, Taiwan

[6]Innovation and Development Center of Sustainable Agriculture (IDCSA), National Chung Hsing University, Taichung 40227, Taiwan

[7]i-Center for Advanced Science and Technology (iCAST), National Chung Hsing University, Taichung 40227, Taiwan

*Correspondence: shueneic@dragon.nchu.edu.tw; Tel.: +886-4-2287-0613

027602@tool.caaumed.org.tw; Tel.: +886-4-2205-3366 ext. 7919

Inventory of supplementary data

I. Supplementary tables

Table S1. List of next-generation sequencing analyses for significantly upregulated genes related to protein chaperoning, endoplasmic reticulum stress, and redox homeostasis in CPYPP-treated MDA-MB-231 and MDA-MB-435 cancer cells.

Table S2. Antibodies used in this study.

Table S3. Primers of q-RT-PCR used in this study.

Table S4. shRNA information in gene knockdown analysis.

II. Supplementary figures

Fig. S1. Pro-paraptotic agents arrest the cell cycle at the G_2/M phase.

Fig. S2. DOCK1 and RACs are not involved in CPYPP-induced paraptosis.

Fig. S3. Paraptosis is not linked to other types of cell death.

Fig. S4. The progression of paraptosis is associated with transcriptional and translational regulation involved in redox homeostasis and proteostasis.

Fig. S5. Effect of CDK inhibitors on CPYPP-induced vacuolation and cell viability.

Fig. S6. CPYPP time-dependently upregulates HSPs and ER stress proteins before vacuole formation.

Fig. S7. CPYPP enhances nuclear localization of UPR proteins.

Fig. S8. CPYPP promotes the interaction of CDK7 with HSPs.

Fig. S9. Activation of HSP enhances paraptosis induction.

Fig. S10. Paraptotic induction activates ubiquitin-proteasome system and its compartmentalization within the nucleus independent of importin/exportin mediation.

Fig. S11. Importin and exportin inhibitors have no effect on proteasome nuclear translocation.

Fig. S12. Paraptotic cell death is primed by ROS provocation.

Fig. S13. CPYPP promotes the interaction of CDK7 with PKR.

Fig. S14. PKR activation is not required for UPS activation and impairment of thiol redox in CPYPP-induced paraptosis.

Supplementary Tables

Table S1. List of next-generation sequencing analyses for significantly upregulated genes related to protein chaperoning, endoplasmic reticulum stress, and redox homeostasis in CPYPP-treated MDA-MB-231 and MDA-MB-435 cancer cells.

Increased gene name	Description	Encoded protein	Fold of change	
			**MDA-MB-231**	**MDA-MB-435**
HMOX1	heme oxygenase 1	HO-1	138.80	136.52
HSPA1B	heat shock protein family, membrane 1B	HSP70	43.12	116.20
HSPA1A	heat shock protein family, membrane 1A	HSP70	42.28	108.41
EID3	EP300 interacting inhibitor of differentiation 3	EID3	15.94	82.15
DNAJB1	DnaJ heat shock protein family (Hsp40) member B1	HSP40	13.01	18.35
ZFAND2A	zinc finger AN1-type containing 2A	ZFAND2A	12.78	33.12
OSGIN1	oxidative stress induced growth inhibitor 1	OSGIN1	12.31	10.78
CHAC1	ChaC glutathione specific gamma-glutamylcyclotransferase 1	CHAC1	7.66	88.67
DNAJB4	DnaJ heat shock protein family (Hsp40) member B4	HSP40	7.38	8.46
SRXN1	sulfiredoxin 1	SRXN1	6.99	7.10
FBXO30	F-box protein 30	FBW7/CDC4	5.73	8.57
SESN2	sestrin 2	SESN2	5.57	21.62
GCLM	glutamate-cysteine ligase modifier subunit	GCLM	5.26	9.04
ATF3	activating transcription factor 3	ATF3	4.75	26.85
SLC7A11	solute carrier family 7 member 11	SLC7A11	4.64	20.75
BAG3	BCL2 associated athanogene 3	BAG3	4.48	5.44
HSPH1	heat shock protein family H (Hsp110) member 1	HSP110	3.65	3.95
GCLC	glutamate-cysteine ligase catalytic subunit	GCLC	3.53	2.28
PPP1R15A	protein phosphatase 1 regulatory subunit 15A	GADD34	3.52	4.47
MXD1	MAX dimerization protein 1	MXD1	3.49	5.44
TXNRD1	thioredoxin reductase 1	TXNRD1	3.30	5.31
SLC3A2	solute carrier family 3 member 2	SLA3A2	3.24	5.06
SQSTM1	sequestosome 1	SQSTM1	3.01	2.62
HERPUD1	homocysteine inducible ER protein with ubiquitin like domain 1	HERPUD1	2.93	3.47
DUSP1	dual specificity phosphatase 1	DUSP1	2.88	4.05
HSP90AA1	heat shock protein 90 alpha family, class A member 1	HSP90	2.84	3.05

DNAJB9	DnaJ heat shock protein family (Hsp40) member B9	HSP40	2.81	3.48
TRIB3	tribbles pseudokinase 3	TRIB3	2.79	3.21
GADD45A	growth arrest and DNA damage inducible alpha	GADD45α	2.47	6.63
GSR	glutathione-disulfide reductase	GSR	2.47	2.62
ATF4	activating transcription factor 4	ATF4	2.35	2.77
DDIT3	DNA damage inducible transcript 3	CHOP	2.01	5.02
DDIT4	DNA damage inducible transcript 4	REDD1	ND	28.56
DUSP2	dual specificity phosphatase 2	DUSP2	ND	5.05
XBP1	X-box binding protein 1	XBP-1	ND	3.85

ND, not detected.

Table S2. Antibodies used in this study.

Antibodies	Source	Identifier	Application
Anti-CDK7	Cell Signaling Technology	Cat# 2916	WB
Anti-CHOP	Cell Signaling Technology	Cat# 2895	WB, IF
Anti-BiP	Cell Signaling Technology	Cat# 3177	WB
Anti-DOCK1/DOCK180	Cell Signaling Technology	Cat# 4846	WB
Anti-phospho-CDK9 (Thr186)	Cell Signaling Technology	Cat# 2549	WB
Anti-CDK9	Cell Signaling Technology	Cat# 2316	WB, IHC
Anti-phospho-4E-BP1 (T37/46)	Cell Signaling Technology	Cat# 9455	WB
Anti-4E-BP1	Cell Signaling Technology	Cat# 9644	WB
Anti-Lamin B1	Cell Signaling Technology	Cat# 13435	WB
Anti-GADD34	Cell Signaling Technology	Cat# 41222	WB
Anti-HSP27 (Ser 82)	Cell Signaling Technology	Cat# 9709	WB,
Anti-phospho-Rpb1 CTD (Ser2)	Cell Signaling Technology	Cat# 13499	WB
Anti-phospho-Rpb1 CTD (Ser5)	Cell Signaling Technology	Cat# 13523	WB
Anti-Rpb1	Cell Signaling Technology	Cat#14958	WB, IF, PLA
Anti-phospho-eIF2α (Ser51)	Cell Signaling Technology	Cat# 9721	WB
Anti-HERPUD1	Cell Signaling Technology	Cat# 26730	WB
Anti-TRIB3	Cell Signaling Technology	Cat# 43043	WB
Anti-IRE1α	Cell Signaling Technology	Cat# 3294	WB, IF
Anti-HSP27 (Ser 82)	Cell Signaling Technology	Cat# 9709	WB,
Anti-HSP27	Cell Signaling Technology	Cat# 95357	WB
Anti-HSP40	Cell Signaling Technology	Cat# 4868	WB, IF, PLA
Anti-HSP70	Cell Signaling Technology	Cat# 4873	WB, IF
Anti-HSP90	Cell Signaling Technology	Cat# 4877	WB
Anti-PSMA2	Cell Signaling Technology	Cat# 2455	WB
Anti-PSMA5	Cell Signaling Technology	Cat# 2457	WB
Anti-PSMD14	Cell Signaling Technology	Cat# 4197	WB
Anti-CUL4A	Cell Signaling Technology	Cat#2699	WB
Anti-CYLD	Cell Signaling Technology	Cat#12797	WB
Anti-SKP1	Cell Signaling Technology	Cat#12248	WB
Anti-SKP2	Cell Signaling Technology	Cat#2652	WB
Anti-MDM2	Cell Signaling Technology	Cat#86935	WB
Anti-p21	Cell Signaling Technology	Cat#2947	WB
Anti-DDB1	Cell Signaling Technology	Cat#6998	WB
Anti-DDB2	Cell Signaling Technology	Cat#5416	WB
Anti-Rac1	EMD Millipore	Cat# 05-839	WB
Anti-Ubiquitin	EMD Millipore	Cat# 05-994	WB, IF
Anti-β-actin	EMD Millipore	Cat# MAB1501	WB
Anti-CDK7	Santa Cruz Biotechnology	Cat# sc-365075	WB, IP, IHC

Anti-CDK9	Santa Cruz Biotechnology	Cat# sc-28321	WB, IP
Anti-ATF3	Santa Cruz Biotechnology	Cat# sc81189	WB, IF
Anti-ATF4	Santa Cruz Biotechnology	Cat# sc-390063	WB
Anti-GADD45α	Santa Cruz Biotechnology	Cat# sc-6850	WB, IF
Anti-TrxR1	Santa Cruz Biotechnology	Cat# sc-28321	WB
Anti-Rac2	Santa Cruz Biotechnology	Cat# sc-517424	WB
Anti-Pol II/Rpb1	Santa Cruz Biotechnology	Cat# sc-55492	WB, IF, PLA
Anti-SQSTM1	Santa Cruz Biotechnology	Cat# sc-28359	WB
Anti-SESN2	Santa Cruz Biotechnology	Cat# sc-393195	WB
Anti-Lamin B1	Santa Cruz Biotechnology	Cat# sc-56144	WB, IF
Anti-HO-1	Santa Cruz Biotechnology	Cat# sc-390991	WB
Anti-PKR	Santa Cruz Biotechnology	Cat# sc-6282	WB
Anti-HSP70	Santa Cruz Biotechnology	Cat# sc-24	WB
Anti-HSP105	Santa Cruz Biotechnology	Cat# sc-74550	WB
Anti-eIF2α	Santa Cruz Biotechnology	Cat# sc-133142	WB
Anti-USP10	Santa Cruz Biotechnology	Cat# sc365828	WB
Anti-Cyclin D1	Santa Cruz Biotechnology	Cat# sc-450	WB
Anti-phospho-CDK7 (Ser164)	ThermoFisher Scientific	Cat# PA5105583	WB
Anti-phospho-CDK7 (Ser 170)	MyBioSource	Cat# MBS9201280	WB
Anti-phospho-PKR (Thr446)	Abcam	Cat# AP1134	WB
Anti-PERK	Proteintech	Cat# 205821-1-AP	WB, IF
Anti-BAG3	Proteintech	Cat# 10599-1-AP	WB
Anti-CDK7	Proteintech	Cat# 67889-1-Ig	IF
Anti-CDK9	Proteintech	Cat# 67256-1-lg	IF, PLA
Anti-20S proteasome core subunit	Enzo	Cat# BML-PW8155	WB, IF

IF, immunofluorescence; IHC, immunohistochemistry; IP, immunoprecipitation; PLA, proximity ligation assay; WB, Western blot.

Table S3. Primers of q-RT-PCR used in this study.

Gene (encoded protein)	Gene Bank acc. no.	Primer sequence
ACTB (β-actin)	AY582799.1	F: 5'-CCAACCGCGAGAAGATGA-3'<br>R: 5'-CCAGAGGCGTACAGGGATAG-3'
ATF3 (ATF3, activating transcription factor 3)	NM_001674.4	F: 5'-CACAGCTCTCTTCTCTCGCC-3'<br>R: 5'-CAAACACCAGTGACCCAGGA-3'
ATF4 (ATF4, activating transcription factor 4)	NM_001675.4	F: 5'-CCAACAACAGCAAGGAGGAT-3'<br>R: 5'-GTGTCATCCAACGTGGTCAG-3'
BAG3 (BAG3, BCL-2-associated athanogene 3)	NM_004281.4	F: 5'-TTGGGTGGAGGCAAAACACT-3'<br>R: 5'-CAGAACAACAAGCAACGGGG-3'
DDIT3 (DDIT3, DNA damage inducible transcripts 3; CHOP, CCAAT/enhancer-binding protein (C/EBP) homologous protein)	S40706.1	F: 5'-CAGAGCTGGAACCTGAGGAG-3'<br>R: 5'-TGGATCAGTCTGGAAAAGCA-3'
DNAJB1 (HSP40, Heat shock protein family member B1)	NM_006145.3	F: 5'-CGTCGGACGAGGAGATCAAG-3'<br>R: 5'-ACTCCCCTTTAGGCCTTCCT-3'
GADD45A (GADD45α, growth arrest and DNA damage inducible alpha)	NM_001924.3	F: 5'-AGTCAGCGCACGATCACTGT-3'<br>R: 5'-TGTTGATGTCGTTCTCGCAG-3'
HMOX1 (HO-1, heme oxygenase 1)	NM_002133.2	F: 5'-TTGCCAGTGCCACCAAGTTC-3'<br>R: 5'-TCAGCAGCTCCTGCAACTCC-3'
HSP90AA1 (HSP90, heat shock protein 90 alpha family class A member 1)	NM_001017963.3	F: 5'-TCTAGTTGACCGTTCCGCAG-3'<br>R: 5'-GCACCCTCAAGTTCACCTCA-3'
HSPA1A (HSPA1, heat shock protein family A member 1A)	NM_005345.6	F: 5'-AGCTGGAGCAGGTGTGTAAC-3'<br>R: 5'-CAGCAATCTTGGAAAGGCCC-3'
HSPA5 (HSPA5, heat shock protein family A (HSP 70) member 5; BiP, binding immunoglobulin protein)	NM_005347.4	F: 5'-CCAACTGTTACAATCAAGGTC-3'<br>R: 5'-ACGAGGAGCAGGAGGAAT-3'
HSPB1 (HSP27, heat shock protein family B member 1)	NM_001540.5	F: 5'-GGCCCAGAAGCTGCAAAATC-3'<br>R: 5'-AAAGAACACACAGGTGGCGG-3'
HSPH1 (HSP105, heat shock protein family member 1)	NM_006644.4	F: 5'-TTTCTTATCAGCCAGCCGCC-3'<br>R: 5'-ATTGGCGATGGTCTCGATGC-3'
MAP1LC3B (LC3B, microtubule associated protein 1 light chain 3 beta)	NM_022818.4	F: 5'-AGCAGCATCCAACCAAAATC-3'<br>R: 5'-CTGTGTCCGTTCACCAACAG-3'
NFE2L2 (Nrf2, nuclear factor-E2-related factor 2)	NM_006164.4	F: 5'-TTCAGCAGCATCCTCTCCACAG-3'<br>R: 5'-GCATGCTGTTGCTGATACTGG-3'
PPP1R15A (GADD34, protein phosphatase 1 regulatory subunit 15A)	NM_014330.5	F: 5'-CTCTGGCAATCCCCCATACC-3'<br>R: 5'-TCTCGCTCACCATACATGCC-3'
SESN2 (SESN2, sestrin 2)	NM_031459.4	F: 5'-GCGAGATCAACAAGTTGCTGG-3'<br>R: 5'-ACAGCCAAACACGAAGGAGG-3'
SLC3A2 (SLC3A2, solute carrier family 3 member 2; 4F2HC)	NMM002394	F: 5'-GTGCTGGGTCCAATTCACAAG-3'<br>R: 5'-CACCCCGGTAGTTGGGAGTA-3'
SLC7A11 (SLC7A11, solute carrier family 7 membrane 11)	XM_011531802.2	F: 5'-TGCTGGGCTGATTTATCTTCG-3'<br>R: 5'-GAAAGGGCAACCATGAAGAGG-3'
SQSTM1 (SQSTM1, sequestosome 1)	NM_003900.4	F: 5'-AAGCCGGGTGGGAATGTTG-3'<br>R: 5'-GCTTGGCCCTTCGGATTCT-3'

Table S4. shRNA information in gene knockdown analysis.

shRNA	Target gene	Gene ID	shRNA clone
shCDK7 #1	*CDK7*	1778	TRCN0000000592
shCDK7 #2	*CDK7*	1778	TRCN0000000595
shCDK9 #1	*CDK9*	1780	TRCN0000000495
shCDK9 #2	*CDK9*	1780	TRCN0000000496
shDOCK1 #1	*DOCK1*	1793	TRCN0000029074
shDOCK1 #2	*DOCK1*	1793	TRCN0000029076
shRAC1 #1	*RAC1*	5879	TRCN0000008430
shRAC1 #2	*RAC1*	5879	TRCN0000008431
shRAC2 #1	*RAC2*	5880	TRCN0000047274
shRAC2 #2	*RAC2*	5880	TRCN0000047276
TRC1.Void			ASN0000000002

Supplementary Figures

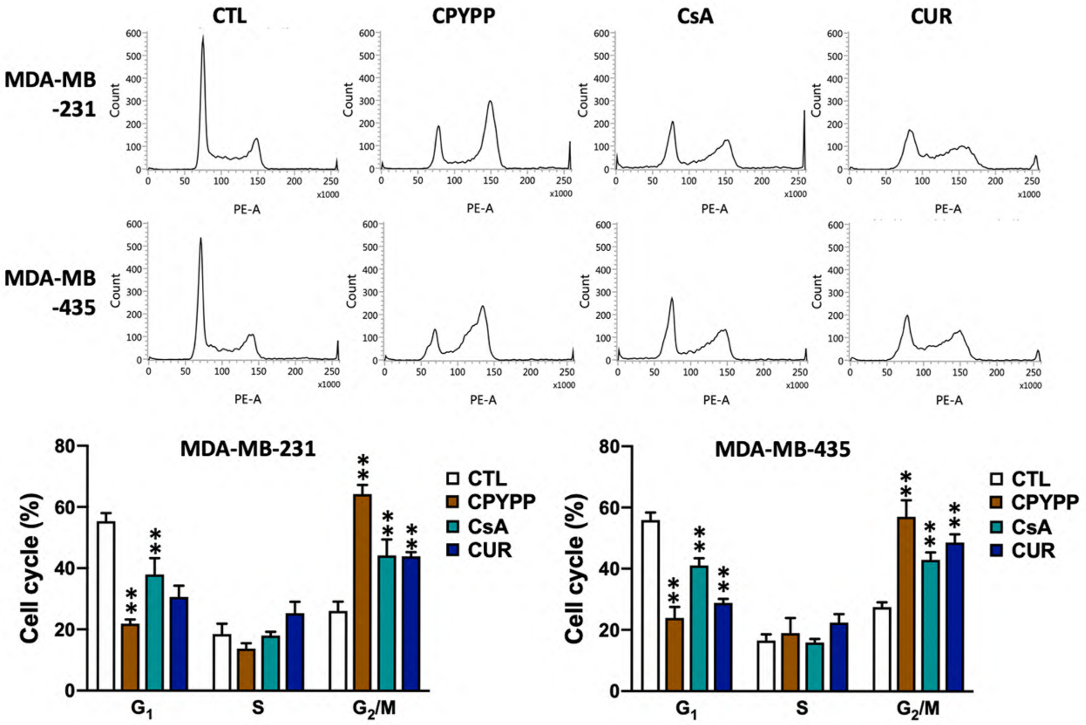

Fig. S1. Pro-paraptotic agents arrest the cell cycle at the G2/M phase. MDA-MB-231 and MDA-MB-435 cancer cells were treated with CPYPP (10 μM), Cyclosporin A (CsA, 20 μM), or curcumin (CUR, 30 μM) for 24 hours. Subsequently, cells were collected for cell cycle progression analysis using propidium iodide staining and flow cytometry analysis. The results are presented as the mean ± SD from three independent experiments. $^{**}P < 0.01$, compared to the corresponding control group (CTL).

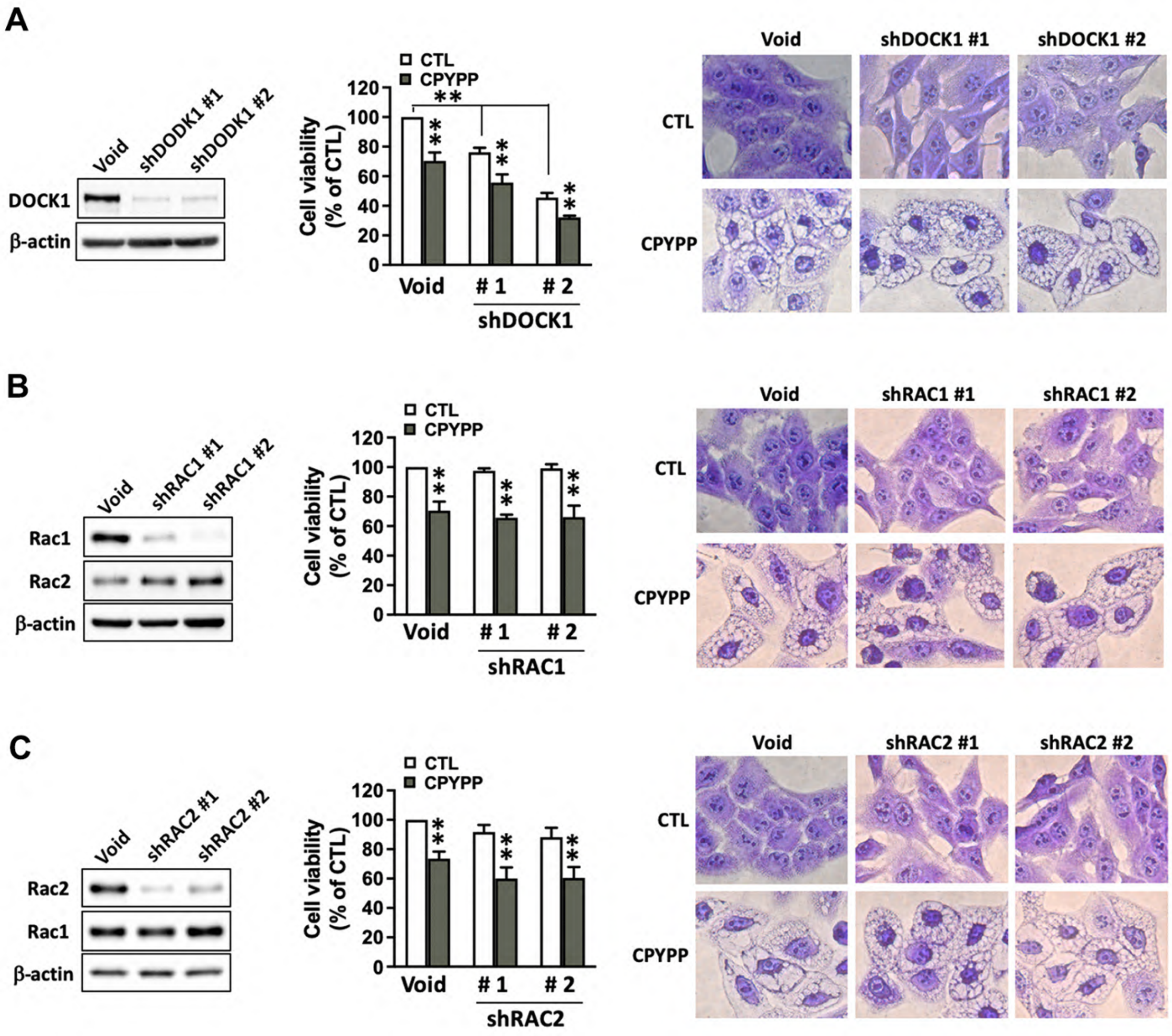

Fig. S2. DOCK1 and RACs are not involved in CPYPP-induced paraptosis. MDA-MB-435 melanoma cells with shRNA intervention for DOCK1 knockdown (Panel **A**), Rac1 knockdown (Panel **B**), or Rac2 knockdown (Panel **C**) were treated with CPYPP (10 μM) for 24 hours to induce paraptotic cell death. Subsequently, cells were collected for Western blot analysis, cell viability assessment using the MTT assay, and cytoplasmic vacuolation analysis through crystal violet staining and imaging under a phase-contrast microscope to monitor paraptosis development. The results are presented as the mean ± SD from three independent experiments. $^{**}P < 0.01$, compared to the corresponding control group (Void).

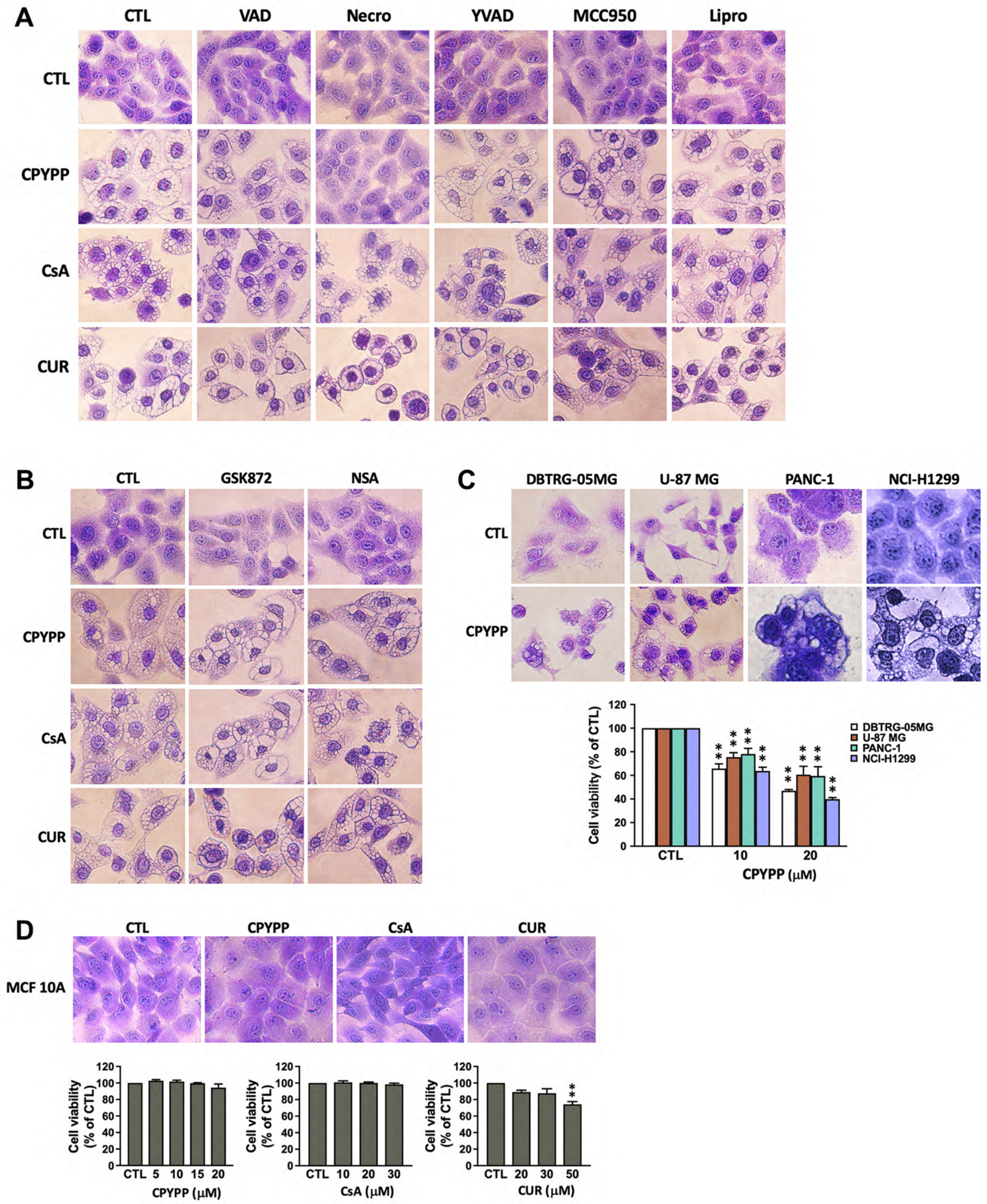

Fig. S3. Paraptosis is not linked to other types of cell death. (**A**) MDA-MB-231 breast cancer cells pretreated with various inhibitors of programmed cell death, including z-VAD-FMK (VAD, 10 μM, apoptosis), necrostatin-1 (Necro, 100 μM, necroptosis), Ac-YVAD-CHO (YVAD, 10 μM, pyroptosis), MCC950 (10 μM, pyroptosis), and liproxstatin-1 (Lipro, 2 μM, ferroptosis), for 30 minutes, were subsequently treated with CPYPP (10 μM), cyclosporin A (CsA, 20 μM), or curcumin (CUR, 30 μM) for 24 hours to induce cell paraptosis. (**B**) MDA-MB-453 melanoma cells were

pretreated with the necroptotic inhibitor, GSK872 (10 μM), or necrosulfonamide (NSA, 5 μM) for 30 minutes before exposure to CPYPP, CsA, or CUR for 24 hours. (**C**, **D**) Human glioblastoma multiforme cells (DBTRG-05MG, U87-MG), pancreatic cancer cells (PANC-1), lung adenocarcinoma cells (NCI-H1299), and the breast epithelial cell line MCF-10A were treated with the indicated concentrations of CPYPP, CsA, or CUR for 24 hours. Subsequently, cells were collected for cytoplasmic vacuolation assessment through crystal violet staining and viability analysis using the MTT method. The results are presented as the mean ± SD from three independent experiments. **$P < 0.01$, compared to the corresponding control group (CTL).

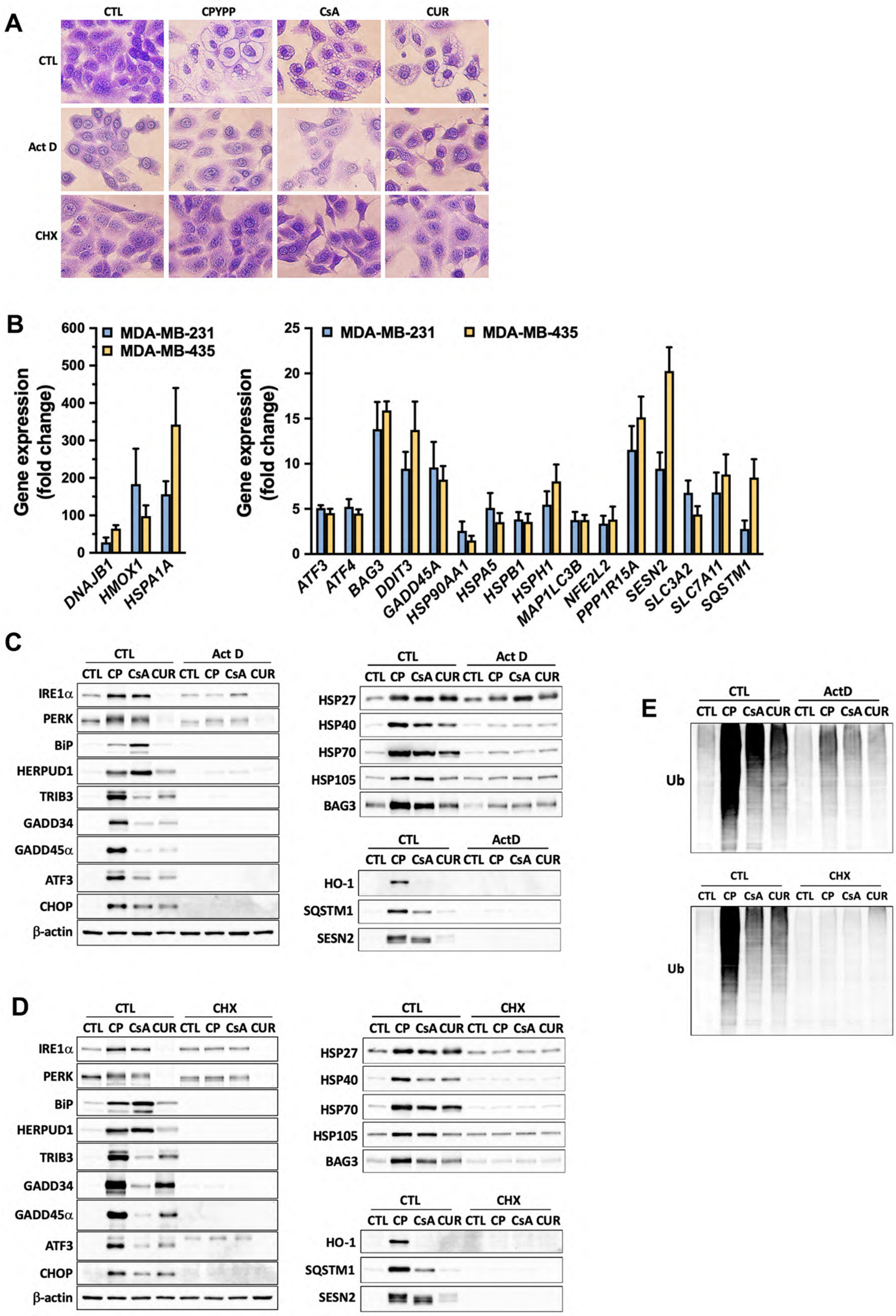

Fig. S4. The progression of paraptosis is associated with transcriptional and translational regulation involved in redox homeostasis and proteostasis. (**A**) Breast cancer cells, MDA-MB-231, were

pretreated with the transcription inhibitor actinomycin A (ActD, 1 μM) or the translation inhibitor cycloheximide (CHX, 20 μM) for 30 minutes, followed by treatment with CPYPP (10 μM), cyclosporin A (CsA, 20 μM), or curcumin (CUR, 30 μM) for 24 hours. Cells were then assessed for morphological vacuolization alterations using crystal violet staining. (**B**) MDA-MB-231 and MDA-MB-435 cancer cells were induced for paraptosis development in the presence of CPYPP (10 μM) for 6 hours and then harvested for total RNA extraction and qRT-PCR analysis. The transcript levels were normalized to β-actin (ACTB) in the same samples. Increased RNA levels were presented as fold change compared to the control. The results are presented as the mean ± SD from three independent experiments. (**C**–**E**) Paraptotic agents promoted protein expressions related to ER stress, UPR (unfolded protein response), heat shock protein (HSP) chaperones, and redox homeostasis through transcriptional and translational regulation. MDA-MB-435 melanoma cells were pretreated with actinomycin D (Act D, 1 μM) or cycloheximide (CHX, 20 μM) for 30 minutes to inhibit transcription and translation activity, and then treated with CPYPP (10 μM), cyclosporin A (CsA, 20 μM), or curcumin (CUR, 30 μM) for 24 hours. Cells were subsequently collected for protein expression (C, D) and ubiquitination analysis (E) using Western blot analysis.

A

CDK inhibitor	Targeted CDKs	Brand	Used concentration
Ro-3306	CDK1	ALX-270-463, ENZO	10 μM
(R)-Roscovitine	CDK2	10009569, Cayman	10 μM
Abemaciclib	CDK4/6	HY-16297A, MCE	0.2 μM
Palbociclib	CDK4/6	HY-50767, MCE	1 μM
BML-259	CDK2/5	HY-108439, MCE	20 μM
THZ1	CDK7	9002215, Cayman	0.2 μM
PHA-767491	CDK7/9	18218, Cayman	5 μM
Senexin A	CDK8	30291, Cayman	50 μM
BRD6989	CDK8/19	HY-122586, MCE	5 μM
LY2857785	CDK9	34880, Cayman	1 μM
SR-4835	CDK12/13	39158, Cayman	5 μM
THZ531	CDK12/13	26386, Cayman	1 μM

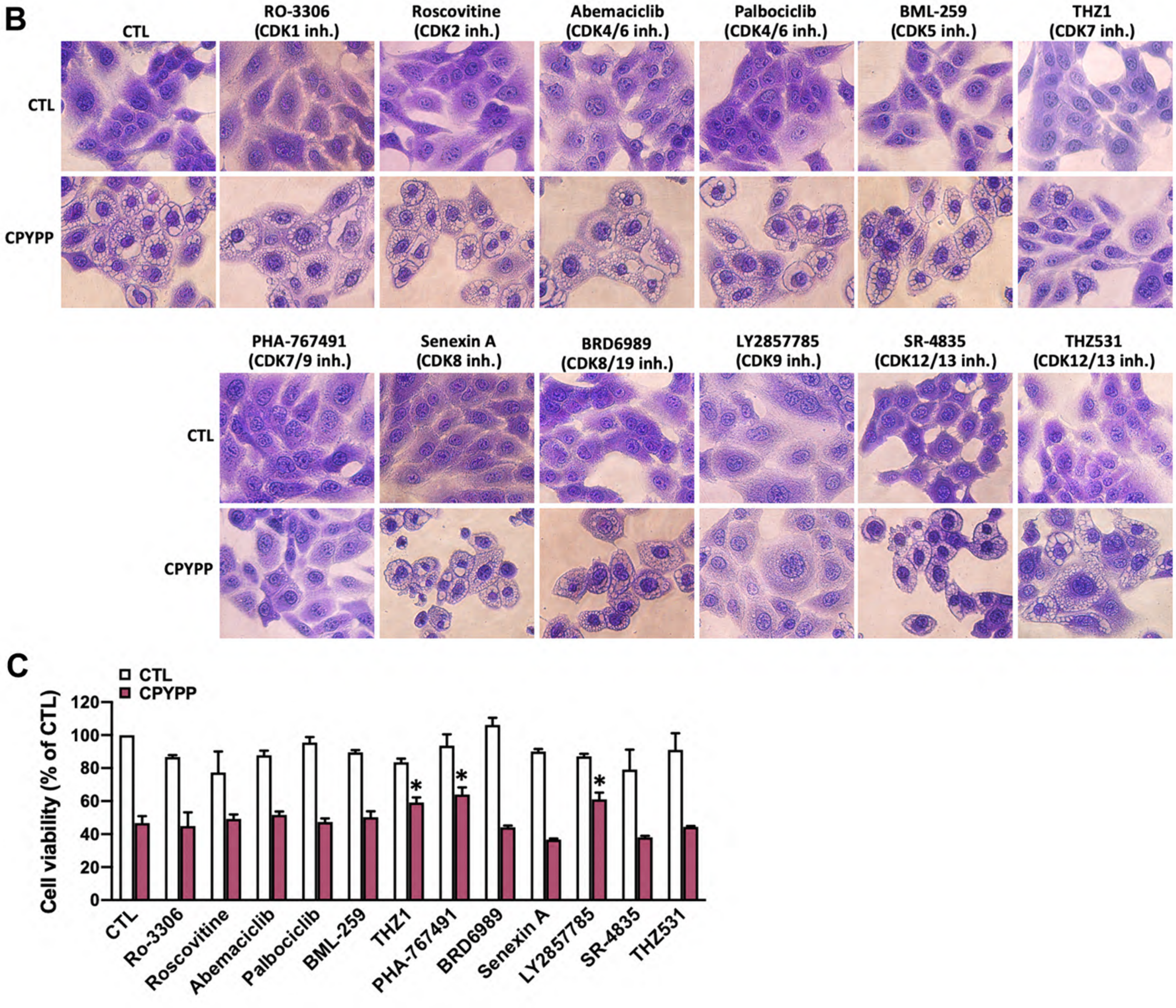

Fig. S5. Effect of CDK inhibitors on CPYPP-induced vacuolation and cell viability. MDA-MB-435 melanoma cells were pretreated with various CDK inhibitors for 30 minutes followed by CPYPP (10 μM) stimulation. After 24 hours of treatment, cell morphology alterations and cell viability were assessed. (**A**) The list of CDK inhibitors, corresponding inhibition of CDKs, and used concentrations. (**B**) Vacuolation induced by treatment. (**C**) Results of cell viability analysis. The results are presented as the mean ± SD from three independent experiments. **P* < 0.05, compared to the corresponding control group (CTL).

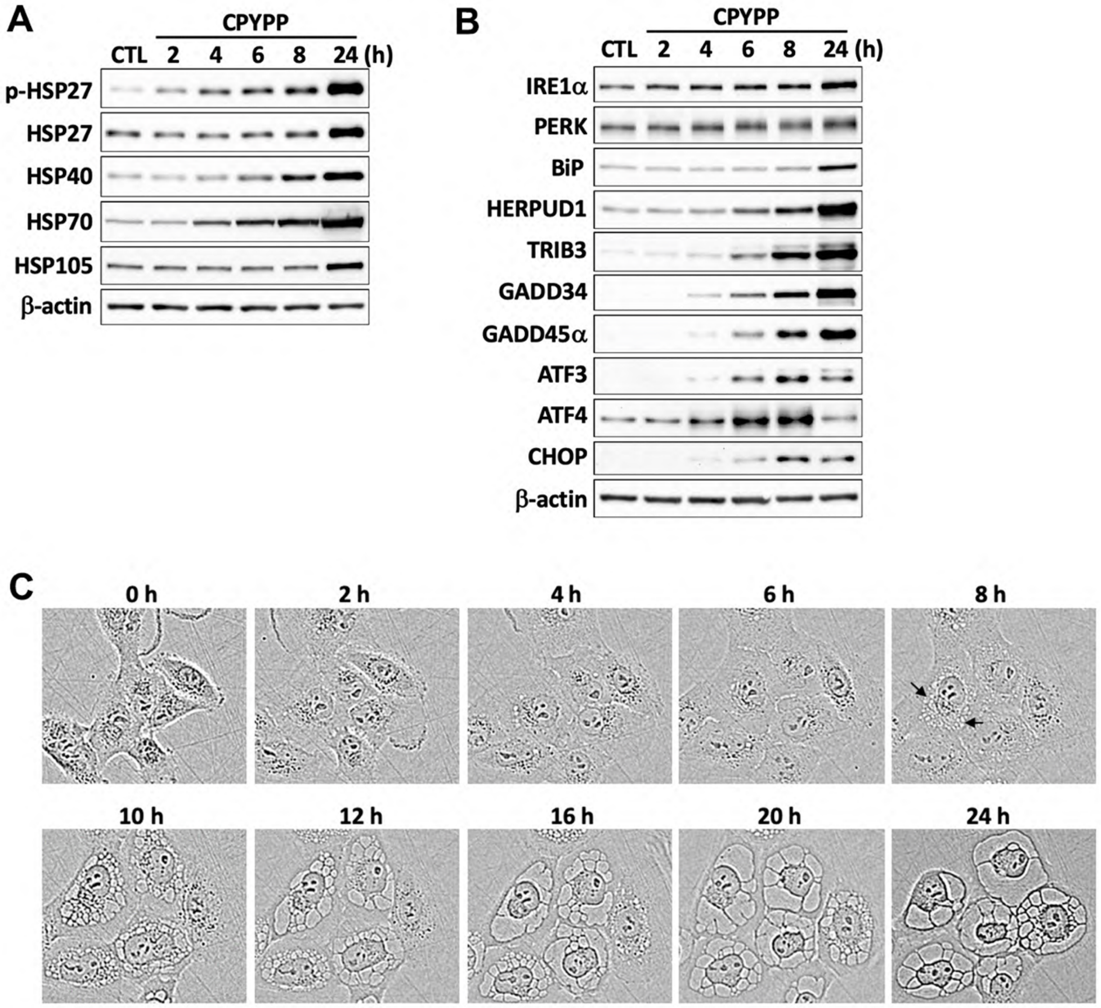

Fig. S6. CPYPP time-dependently upregulates HSPs and ER stress proteins before vacuole formation. MDA-MB-435 melanoma cells were treated with CPYPP (10 μM) for the indicated time. Protein expressions of HSPs and ER stress proteins were analyzed using Western blot analysis (panels **A** and **B**). Morphological vacuolization alterations were visualized through time-lapse images captured using an Incucyte® SX5 Live-Cell Analysis System (Sartorius Stedim Biotech) (panel **C**).

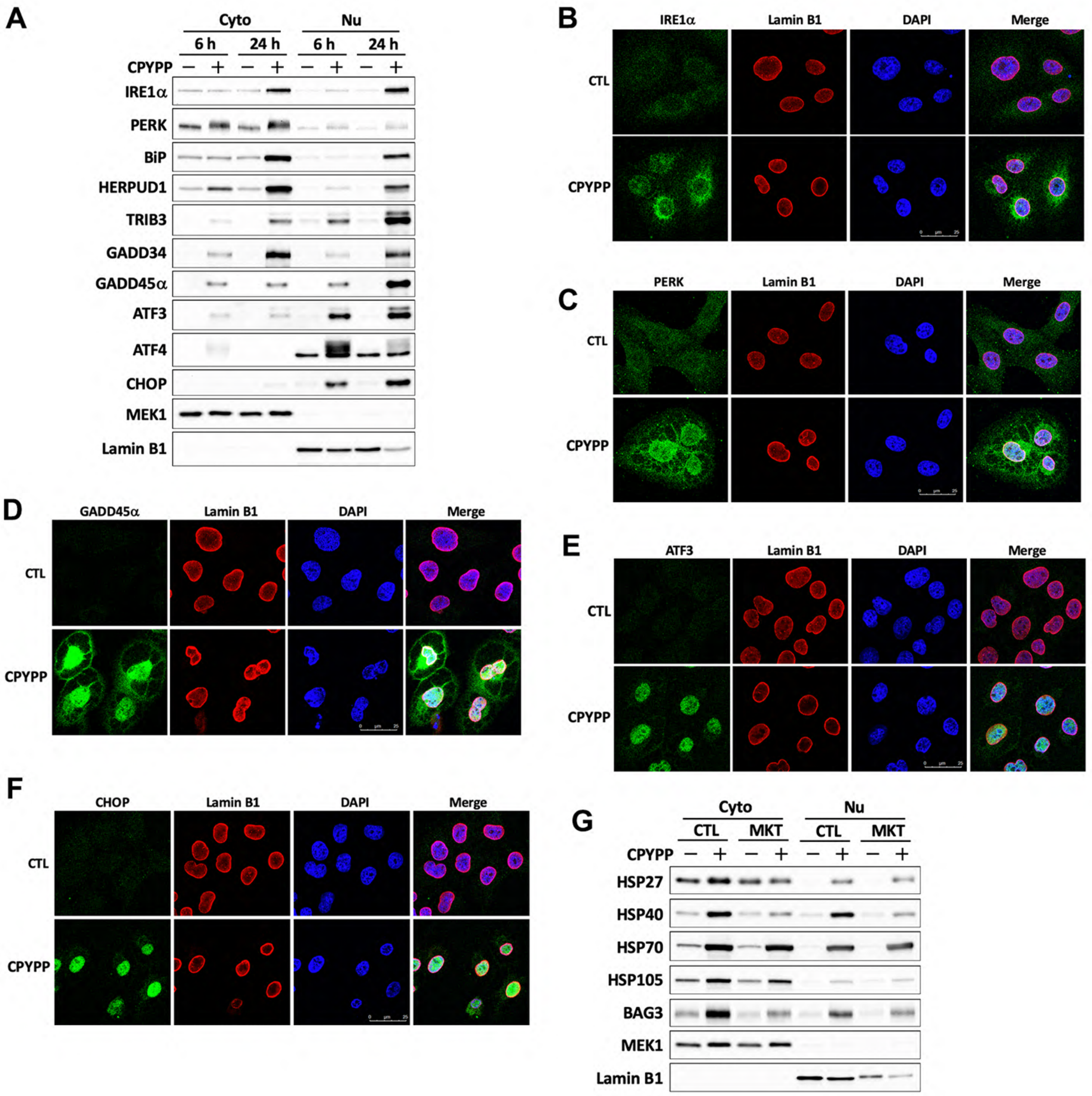

Fig. S7. CPYPP enhances nuclear localization of UPR proteins. (**A**–**F**) MDA-MB-435 cells were treated with CPYPP (10 μM) for 6 or 24 hours to induce cell paraptosis. The nuclear localization of UPR proteins (IRE1α, PERK, GADD45α, ATF3, or CHOP, shown in green) was analyzed using Western blot studies with cytosolic and nuclear fractions (**A**) or by Lamin B1 staining (a nuclear membrane marker, shown in red) along with DAPI counterstaining of nuclei (shown in blue) (24 hours, **B**–**F**). The images were captured using confocal microscopy at 630× magnification. The scale bar represents 25 μm. (**G**) MDA-MB-435 breast cancer cells, pretreated with MKT-077 (20 μM) for 30 minutes to interfere with HSP functions, were subsequently treated with CPYPP (10 μM) for 24 hours. Cells were then collected for cytosolic (Cyto) and nuclear (Nu) fraction isolation, followed by Western blot analysis.

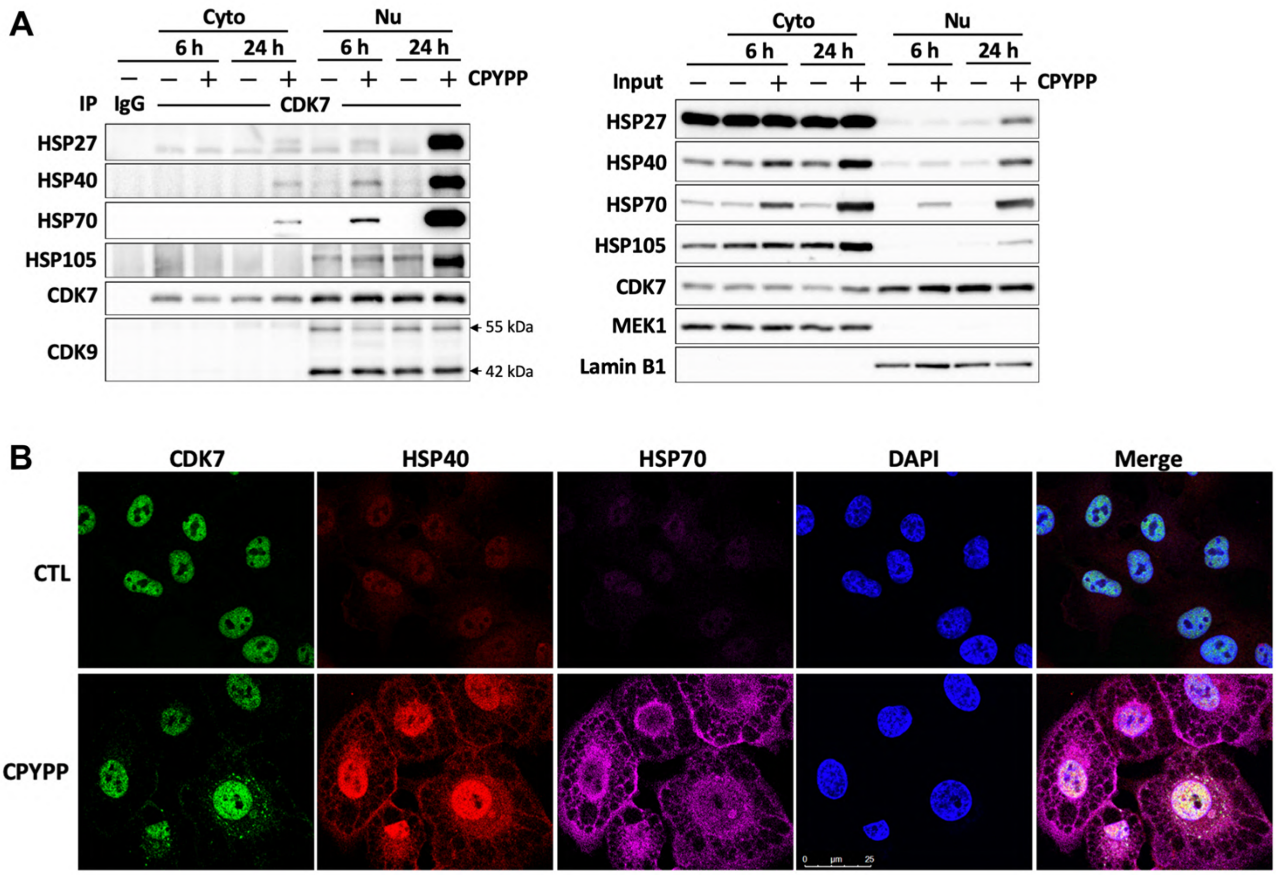

Fig. S8. CPYPP promotes the interaction of CDK7 with HSPs. MDA-MB-435 melanoma cells were treated with CPYPP (10 µM) for 6 or 24 hours. Subsequently, cells were collected for cytosolic (Cyto) and nuclear (Nu) fraction isolation, followed by co-immunoprecipitation and Western blot analysis to assess CDK7 interactions (**A**). (**B**) The co-localization of CDK7 and HSPs was detected by co-immunofluorescence staining with anti-CDK7 (shown in green), anti-HSP40 (shown in red), and anti-HSP70 (shown in magenta) antibodies, followed by individual secondary antibodies, along with DAPI counterstaining of nuclei (shown in blue). The images were captured using confocal microscopy at 630× magnification. The scale bar represents 25 µm.

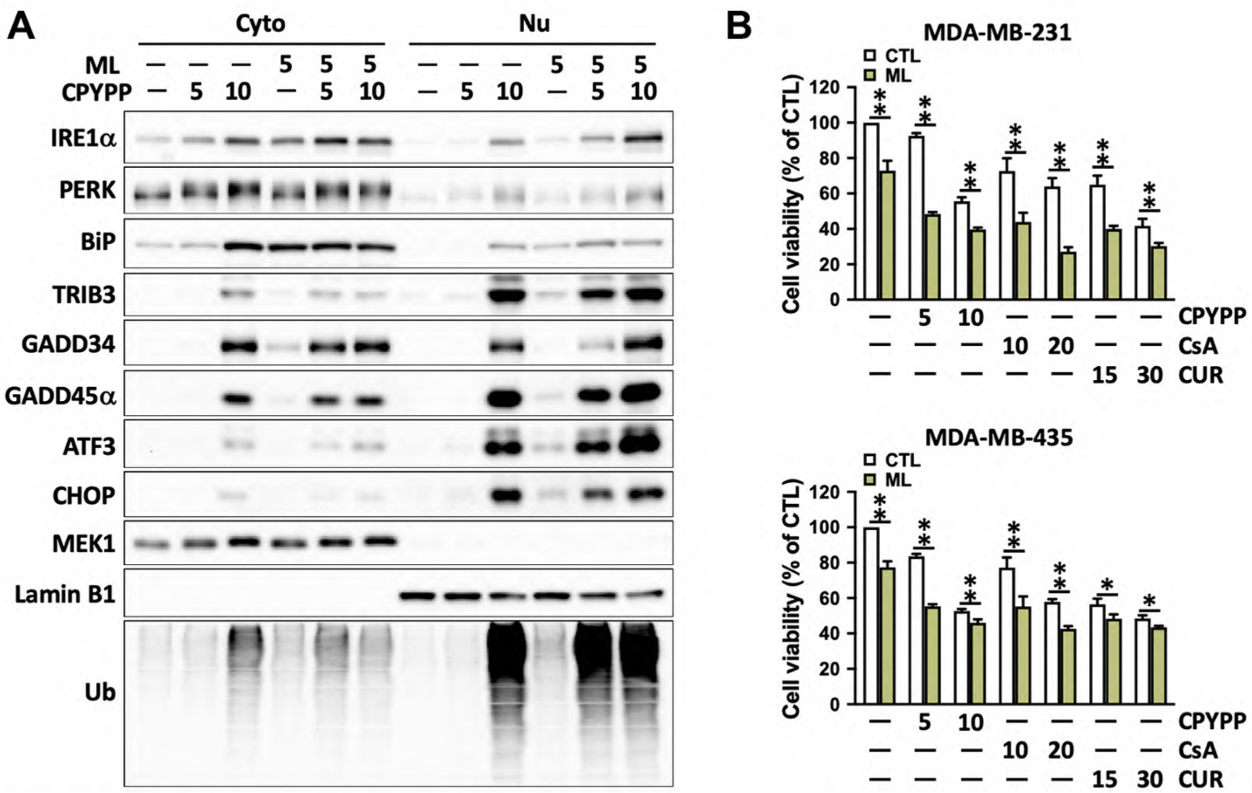

Fig. S9. Activation of HSP enhances paraptosis induction. MDA-MB-435 cells were pretreated with ML346 (5 μM) for 30 minutes followed by indicated concentrations of CPYPP, cyclosporin A (CsA), or curcumin (CUR) for 24 hours. Cells were then collected for protein expression (**A**) and cell viability (**B**) analyses. **P* < 0.05 or ***P* < 0.01, compared to the corresponding control group (CTL).

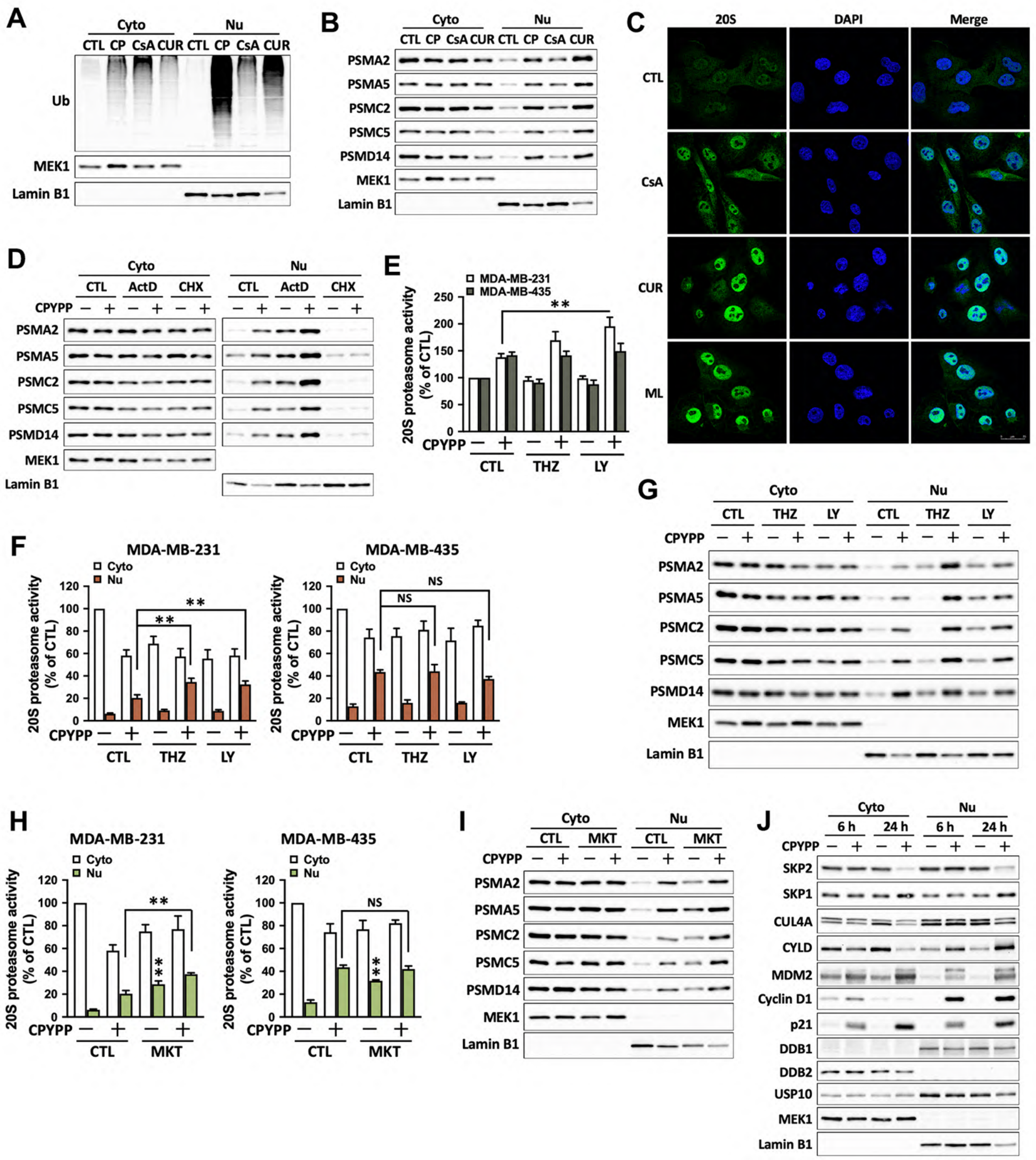

Fig. S10. Paraptotic induction activates the ubiquitin-proteasome system and its compartmentalization within the nucleus independent of importin/exportin mediation. (**A**–**C**) MDA-MB-435 melanoma cells were treated with CPYPP (10 µM), cyclosporin A (CsA, 20 µM), or curcumin (CUR, 30 µM) for 24 hours to induce cell paraptosis. Harvested cells were used for cytosolic (Cyto) and nuclear (Nu) protein ubiquitination or proteasome subunit abundance analysis by Western blot (**A**, **B**). The nuclear localization of 20S proteasomes was visualized using an anti-20S core proteasome subunit antibody, followed by secondary anti-rabbit-Alexa 488 antibody (shown in green), along with DAPI counterstaining of nuclei (shown in blue) (**C**). The images were captured

using confocal microscopy at 630× magnification. The scale bar represents 25 μm. (**D**–**I**) Cancer cells pretreated with the transcription inhibitor actinomycin D (ActD, 1 μM), translation inhibitor cycloheximide (CHX, 20 μM), CDK7 inhibitor THZ1 (THZ, 0.2 μM), CDK9 inhibitor LY2858895 (LY, 1 μM), or HSP inhibitor MKT-077 (MKT, 20 μM) for 30 minutes were subsequently treated with CPYPP (10 μM) for 24 hours. Cells were then collected for whole-cell lysates (**E**) and cytosolic and nuclear fraction isolation (**F**, **H**) for 20S proteasome activity and proteasome subunit abundance analysis (D, G, I, MDA-MB-435 cells). The results are presented as the mean ± SD from three independent experiments. $^{**}P < 0.01$, compared to the corresponding control group (CTL). NS indicates no significant differences. (**J**) MDA-MB-435 cancer cells treated with CPYPP for 6 or 24 hours were used to isolate cytosolic and nuclear fractions for Western blot analysis.

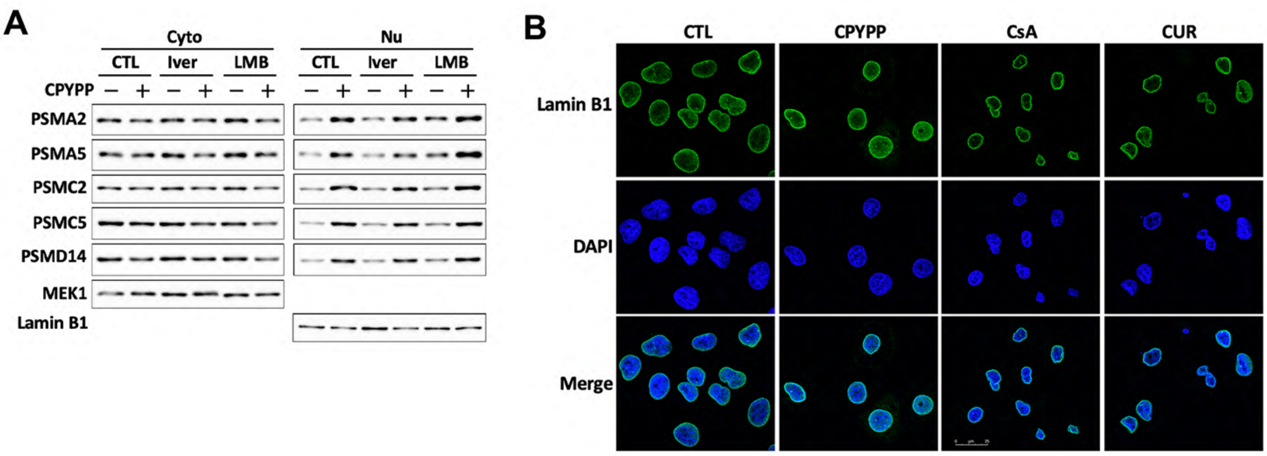

Fig. S11. Importin and exportin inhibitors have no effect on proteasome nuclear translocation. (**A**) MDA-MB-435 cells were pretreated with ivermectin (Iver, 5 μM, an importin inhibitor) or leptomycin B (LMB, 200 ng/ml, an exportin inhibitor) for 30 minutes, followed by treatment with 10 μM CPYPP for 6 hours. Subsequently, cytosolic and nuclear proteasome subunit abundance were detected using Western blot analysis. (**B**) MDA-MB-435 cells were treated with CPYPP (10 μM), cyclosporin A (CsA, 20 μM), or curcumin (CUR, 30 μM) for 24 hours to induce cell paraptosis. Nuclear membrane integrity was visualized using an anti-Lamin B1 antibody, followed by secondary anti-rabbit-Alexa 488 antibody (shown in green), along with DAPI counterstaining of nuclei (shown in blue) (C), respectively. Images were captured using confocal microscopy at 630× magnification. The scale bar represents 25 μm.

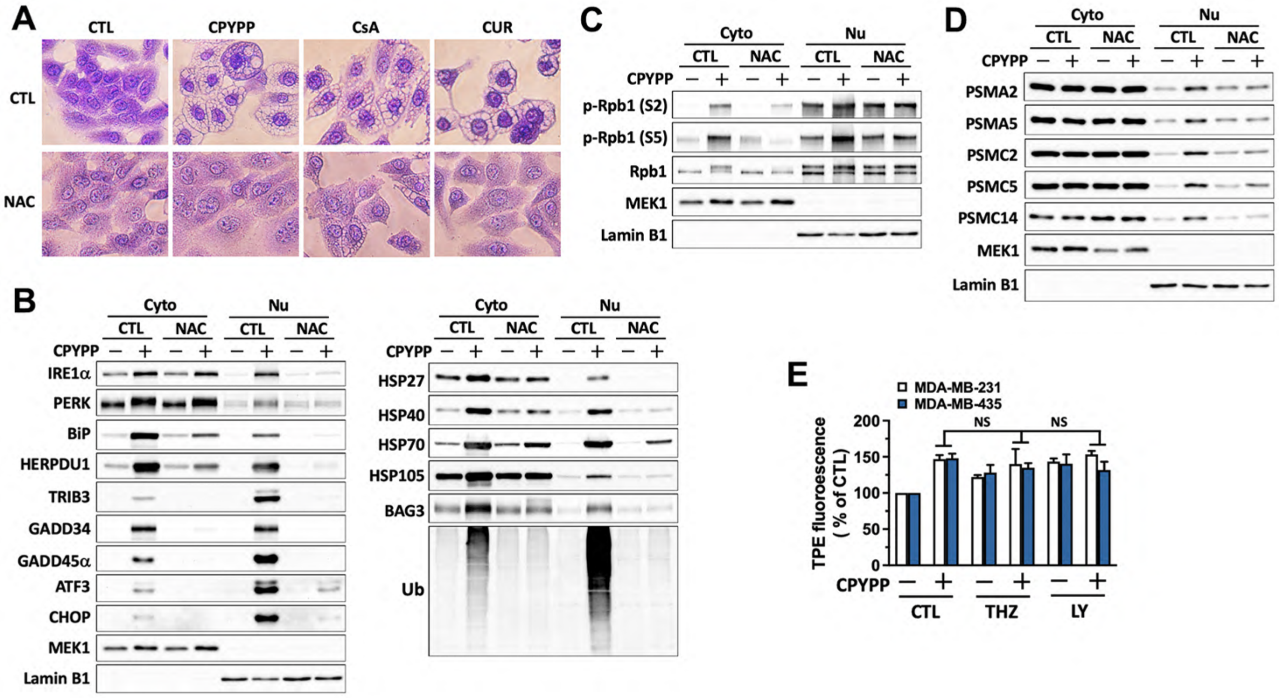

Fig. S12. Paraptotic cell death is primed by ROS provocation. (**A**–**D**) MDA-MB-231 cells, pretreated with NAC (3 mM) for 30 minutes, were subsequently treated with CPYPP (10 μM), cyclosporin A (CsA, 20 μM), or curcumin (CUR, 30 μM) for 24 hours. Cells were then collected for cytoplasmic vacuolation assessment (**A**) or for cytosolic and nuclear fractionation to analyze protein abundance using Western blot (**B**–**D**). (**E**) MDA-MB-231 and MDA-MB-435 cells, pretreated with the CDK7 inhibitor THZ1 (0.2 μM) or the CDK9 inhibitor LY2858895 (LY, 1 μM) for 30 minutes, were subsequently treated with CPYPP (10 μM) for 24 hours. Cells were then collected to determine free cysteine thiols using TPE-MI staining. NS indicates no significant difference.

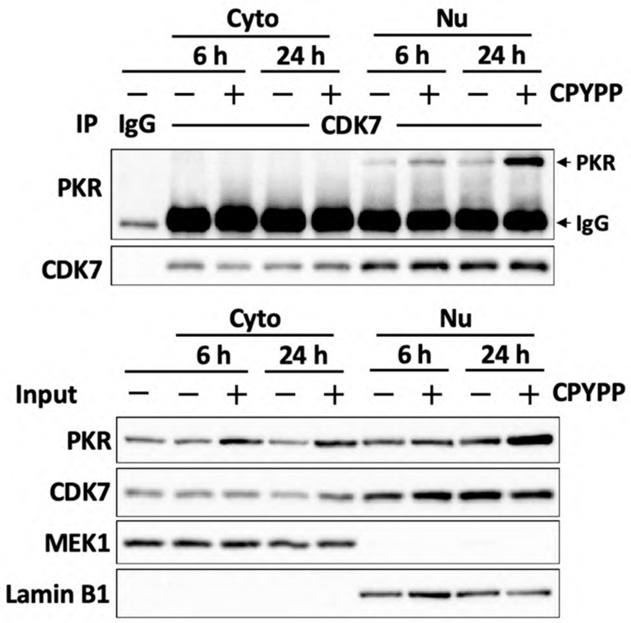

Fig. S13. CPYPP promotes the interaction of CDK7 with PKR. MDA-MB-435 melanoma cells were treated with CPYPP (10 μM) for 6 or 24 hours. Subsequently, cells were collected for cytosolic (Cyto) and nuclear (Nu) fraction isolation, followed by co-immunoprecipitation and Western blot analysis to assess CDK7 interactions.

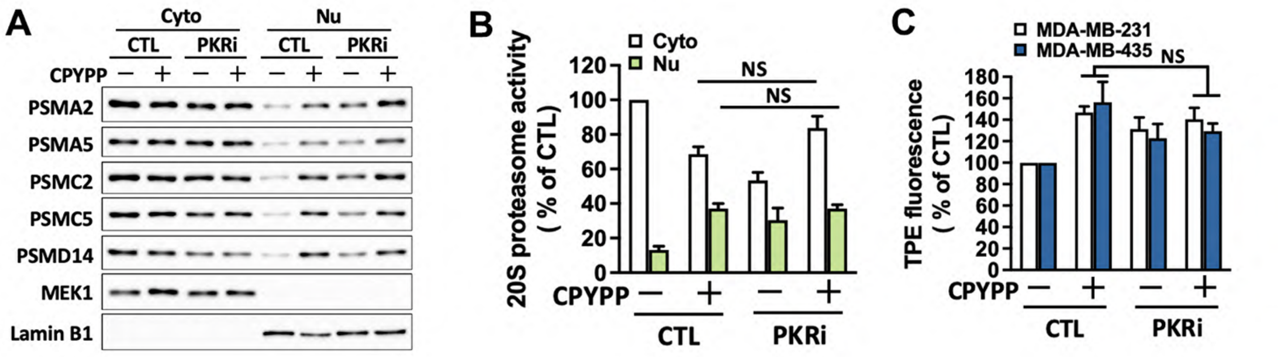

Fig. S14. PKR activation is not required for UPS activation and impairment of thiol redox in CPYPP-induced paraptosis. MDA-MB-435 melanoma cells, pretreated with a PKR inhibitor (PKRi, 2 μM) for 30 minutes, were subsequently treated with CPYPP (10 μM) for 24 hours. Cells were then collected for cytosolic and nuclear fractionation to analyze proteasome subunit abundance by Western blot (**A**), assess 20S proteasome activity (**B**), or determine free cysteine thiols using TPE-MI staining (**C**). NS indicates no significant difference.
