# Supplementary material for: CDK7/CDK9 mediates transcriptional activation to prime paraptosis in cancer cells
Source: Cell Biosci. 2024 Jun 10;14:78. doi: 10.1186/s13578-024-01260-2 (PMC11163730; doi:10.1186/s13578-024-01260-2)
Supplement: Supplementary file 2 — Supplementary Material 2 [file 13578_2024_1260_MOESM2_ESM.pdf]

Additional file: uncropped western blots

CDK7/CDK9 mediates transcriptional activation to prime paraptosis in cancer cells

Shih-Kai Chiang [1], Wei-Chao Chang [2,3,4], Shuen-Ei Chen [1,5,6,7,*], and Ling-Chu Chang [2,3,4,*]

[1]Department of Animal Science, National Chung Hsing University, Taichung 40227, Taiwan

[2]Center for Molecular Medicine, China Medical University Hospital, Taichung 404327, Taiwan

[3]Research Center for Cancer Biology, China Medical University, Taichung 40402, Taiwan

[4]Cancer Biology and Precision Therapeutics Center, China Medical University, Taichung 40402, Taiwan

[5]The iEGG and Animal Biotechnology Center, National Chung Hsing University, Taichung 40227, Taiwan

[6]Innovation and Development Center of Sustainable Agriculture (IDCSA), National Chung Hsing University, Taichung 40227, Taiwan

[7]i-Center for Advanced Science and Technology (iCAST), National Chung Hsing University, Taichung 40227, Taiwan

Fig. 2F (left panel)

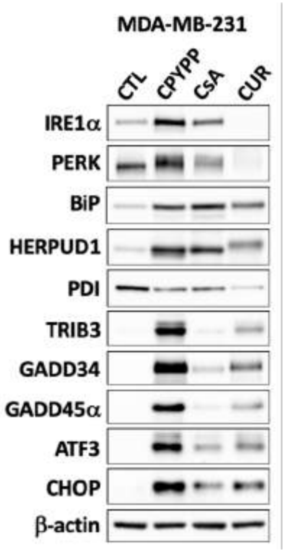

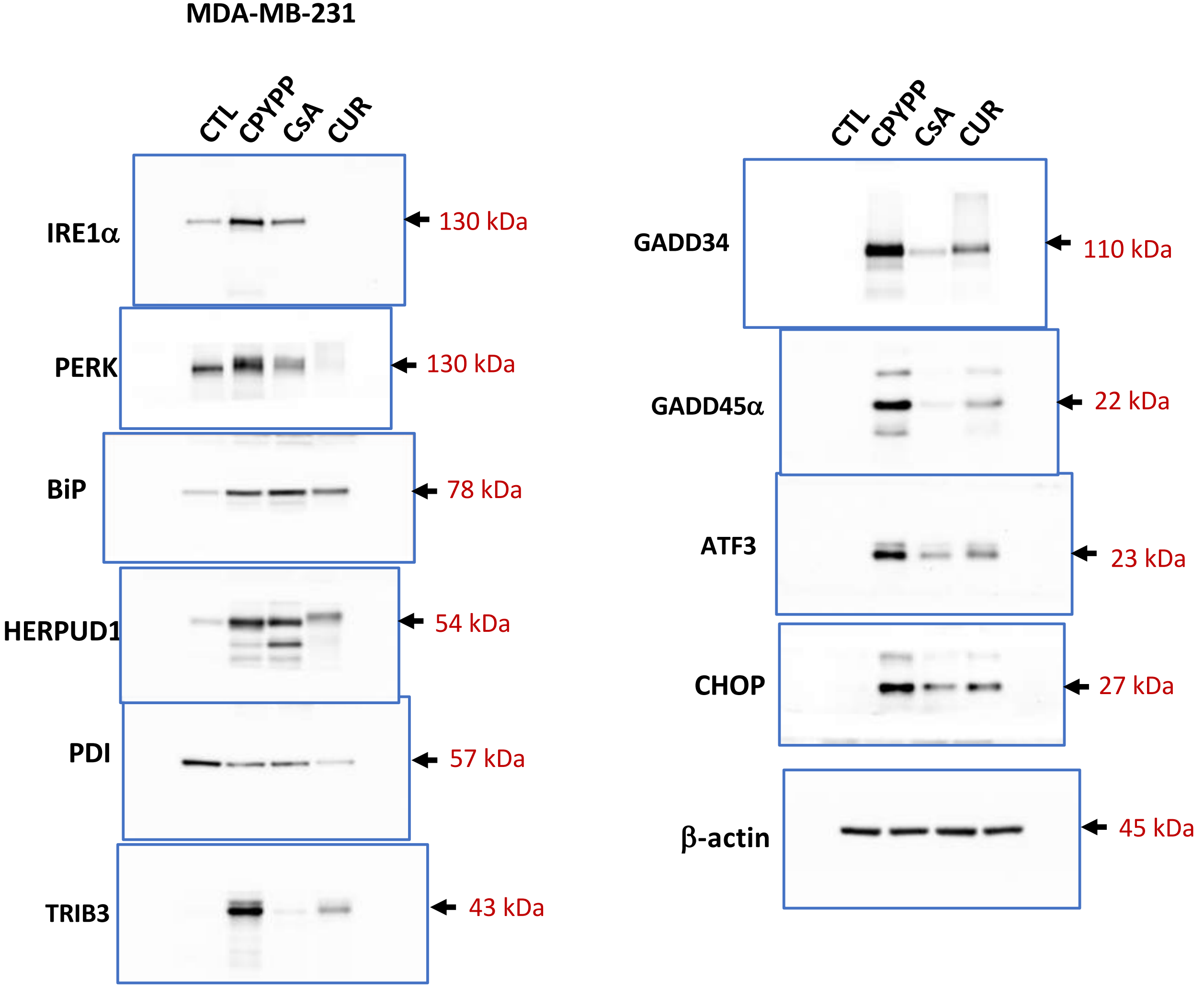

Fig. 2F (right panel)

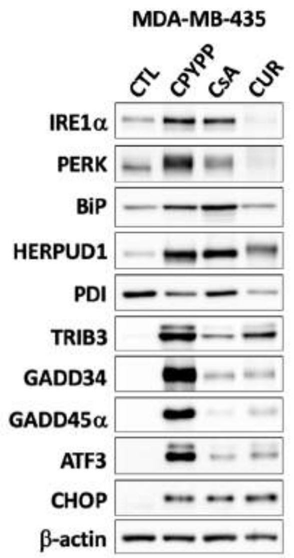

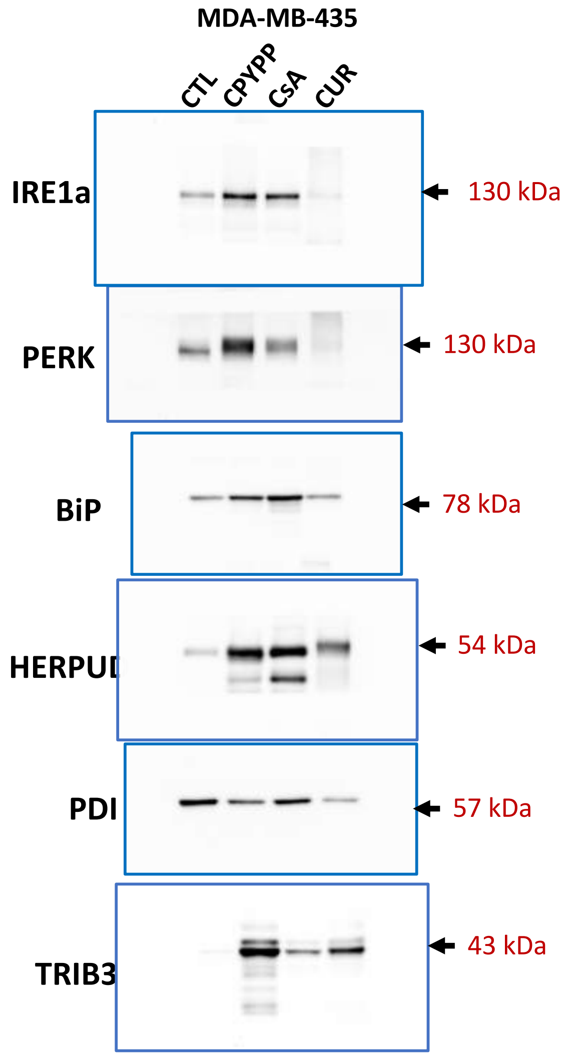

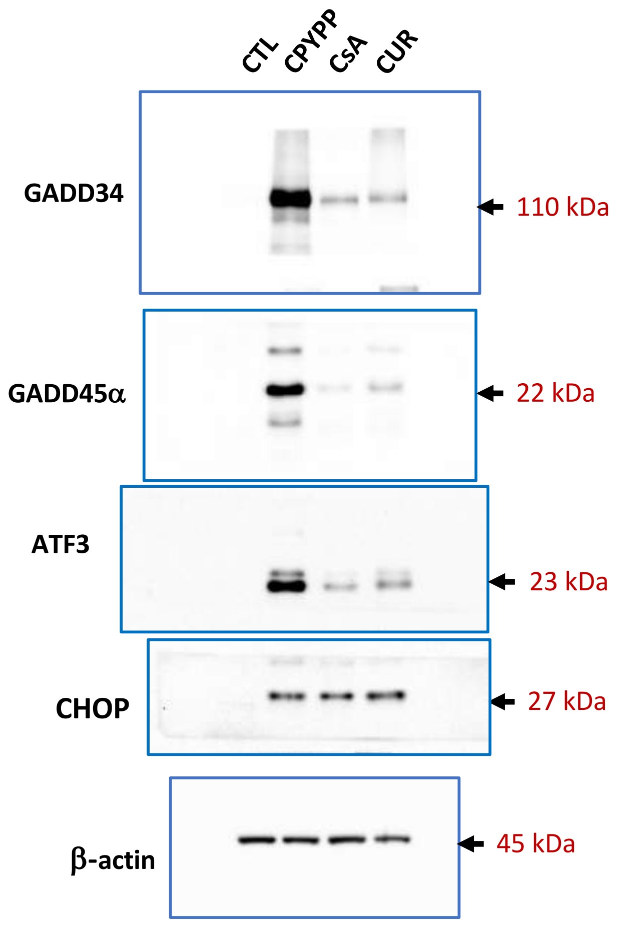

Fig. 2G

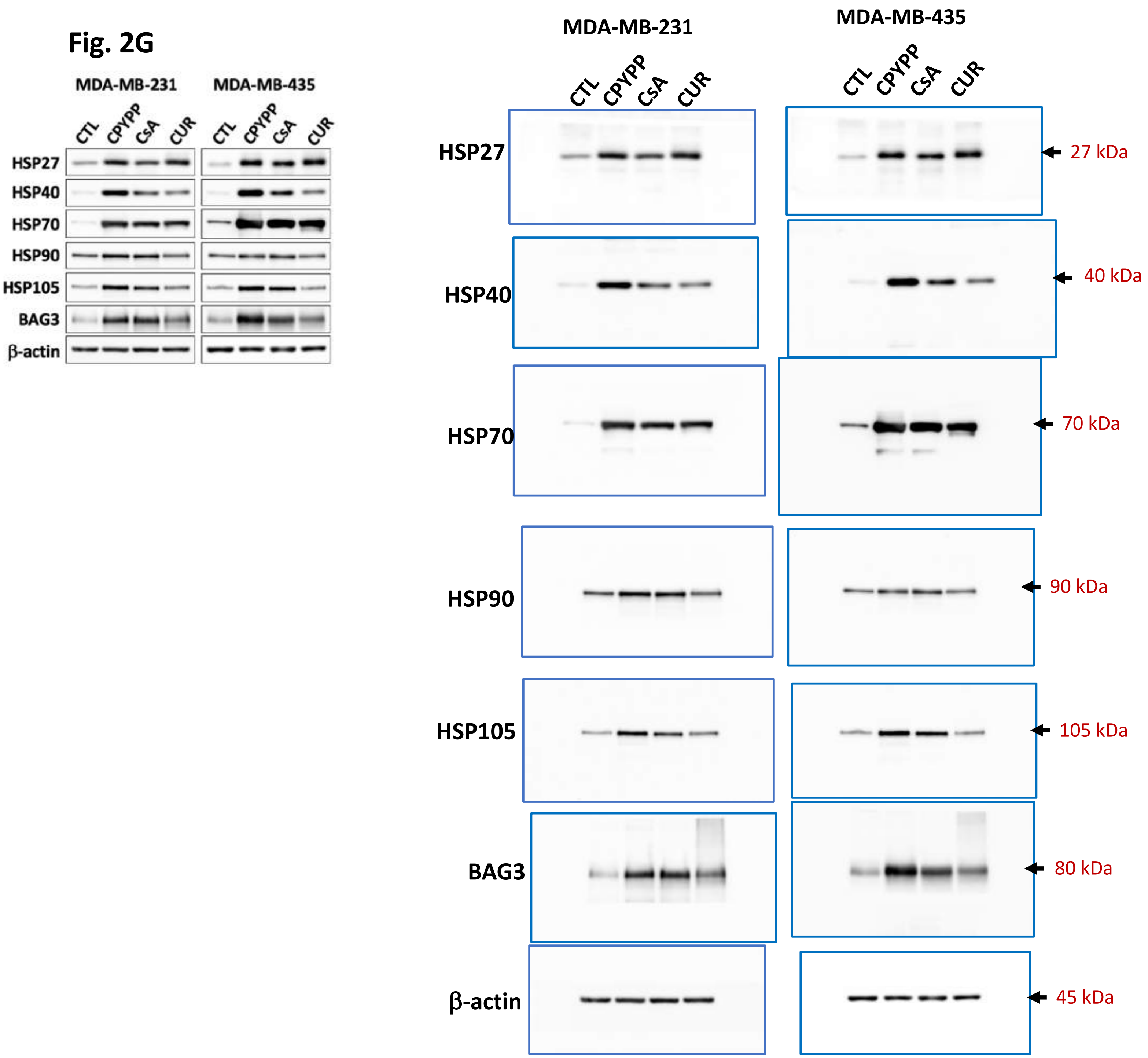
MDA-MB-231
MDA-MB-435
CTL
CPYPP
CsA
CUR
HSP27
HSP40
HSP70
HSP90
HSP105
BAG3
β-actin
27 kDa
40 kDa
70 kDa
90 kDa
105 kDa
80 kDa
45 kDa

Fig. 2H

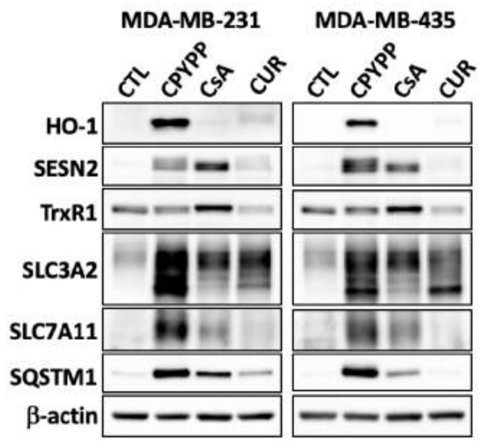

MDA-MB-231
MDA-MB-435
CTL
CPYPP
CsA
CUR
HO-1
SESN2
TrxR1
SLC3A2
SLC7A11
SQSTM1
β-actin

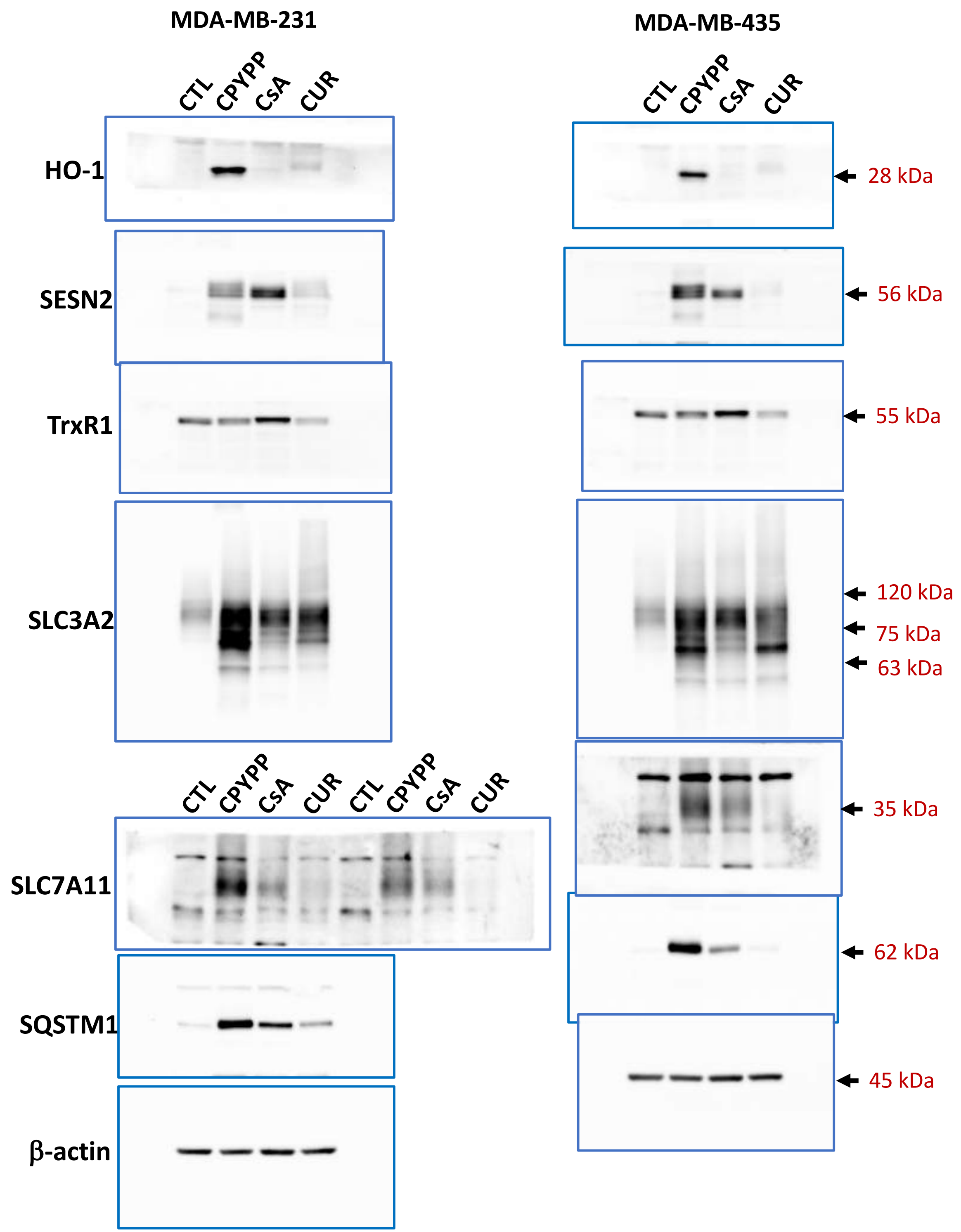

MDA-MB-231
MDA-MB-435
CTL
CPYPP
CsA
CUR
HO-1
SESN2
TrxR1
SLC3A2
SLC7A11
SQSTM1
β-actin
28 kDa
56 kDa
55 kDa
120 kDa
75 kDa
63 kDa
35 kDa
62 kDa
45 kDa

Fig. 2I

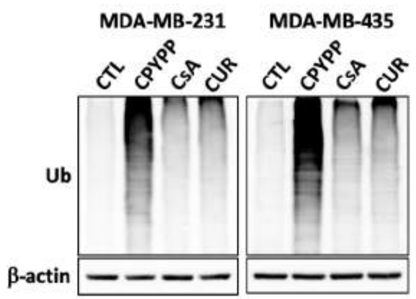
MDA-MB-231
MDA-MB-435
CTL
CPYPP
CsA
CUR
Ub
β-actin

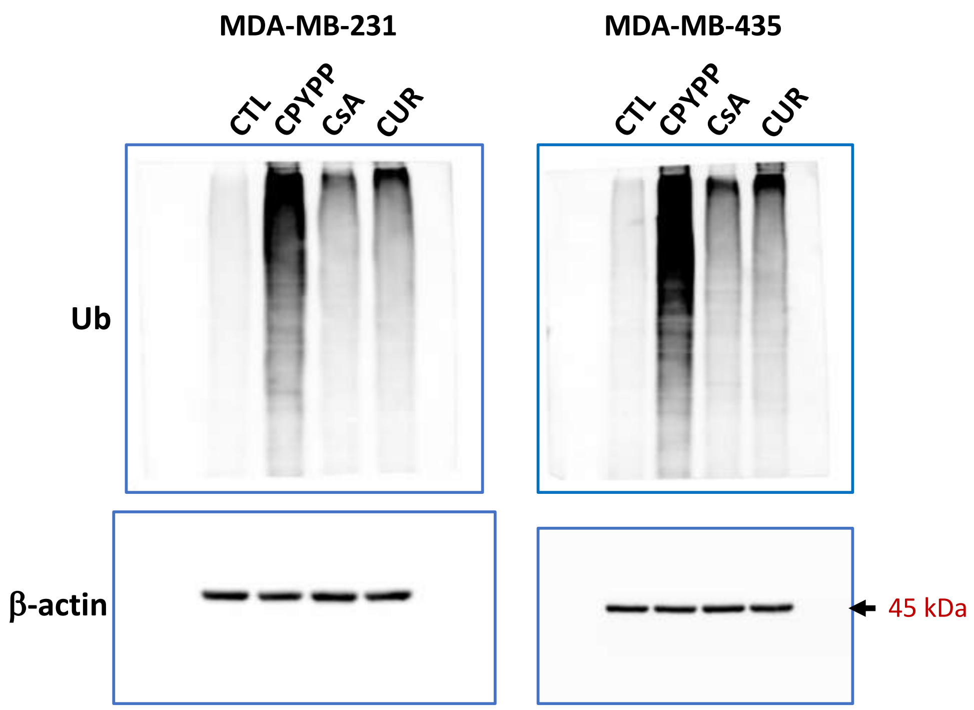
MDA-MB-231
MDA-MB-435
CTL
CPYPP
CsA
CUR
Ub
β-actin
45 kDa

Fig. 2J

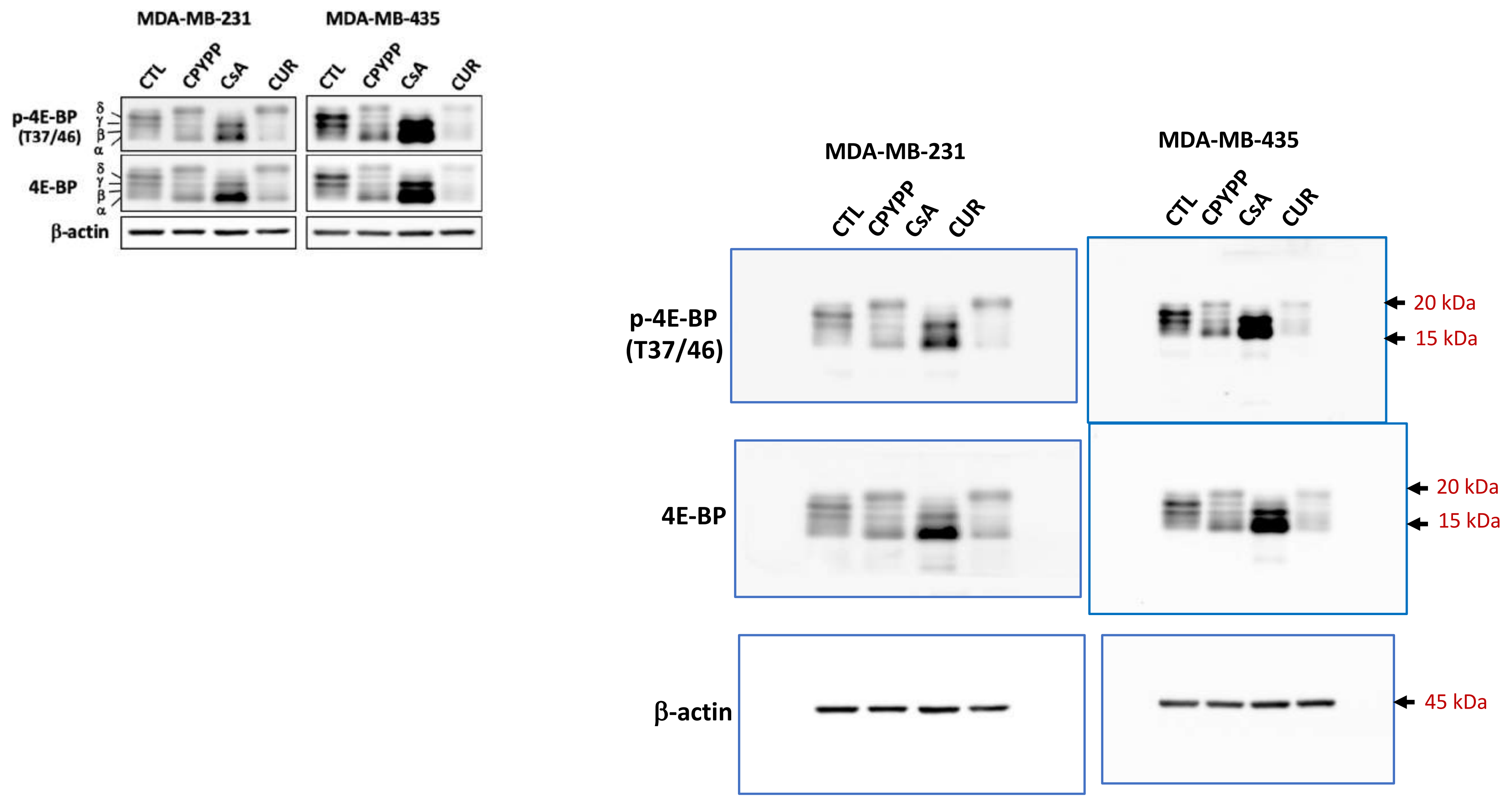

MDA-MB-231
MDA-MB-435
CTL
CPYPP
CsA
CUR
p-4E-BP (T37/46)
4E-BP
β-actin
δ
γ
β
α
20 kDa
15 kDa
45 kDa

Fig. 3C (left panel)

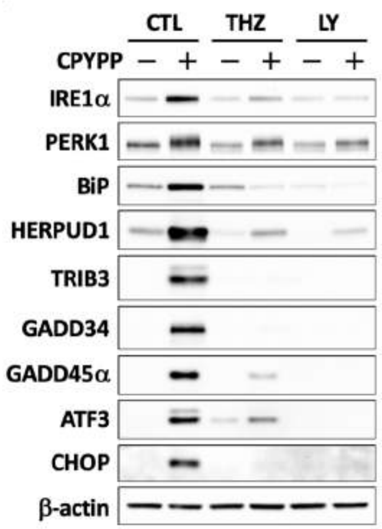

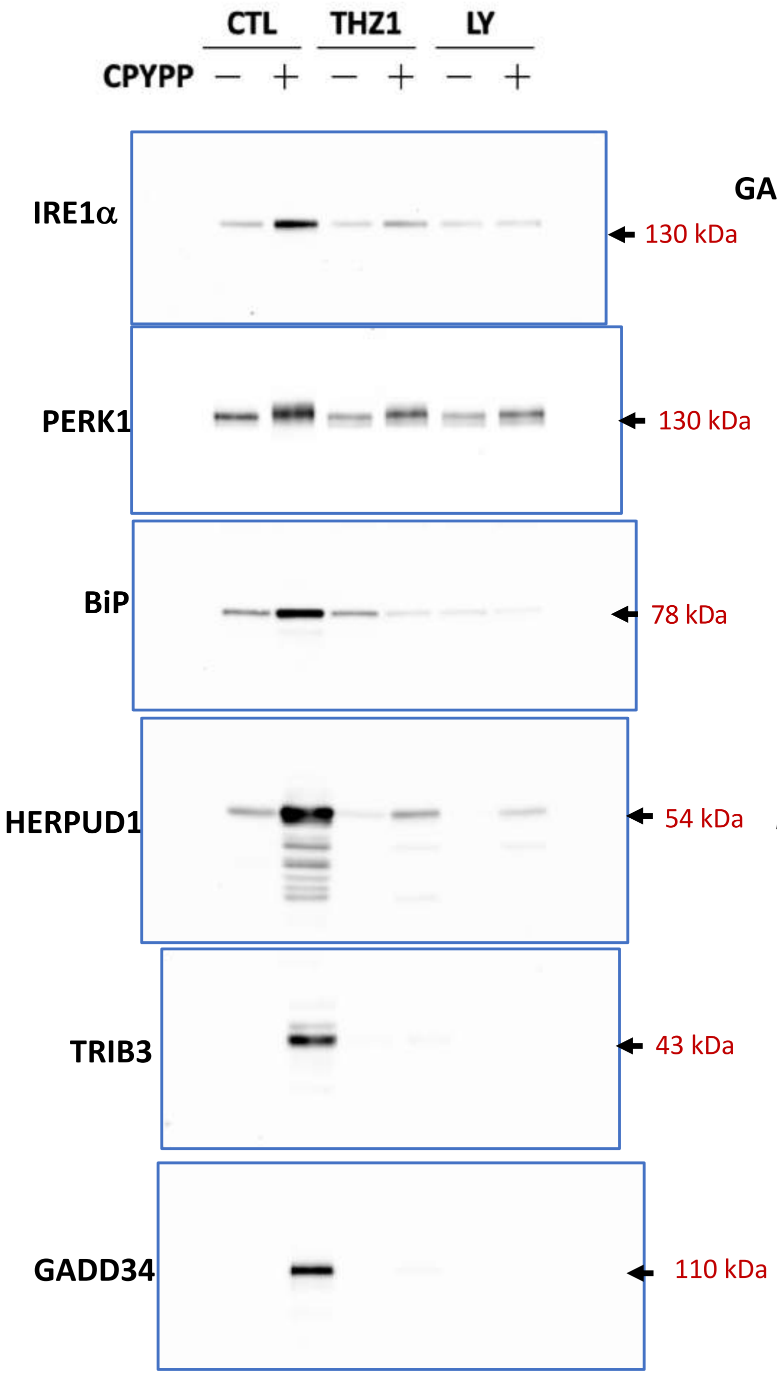

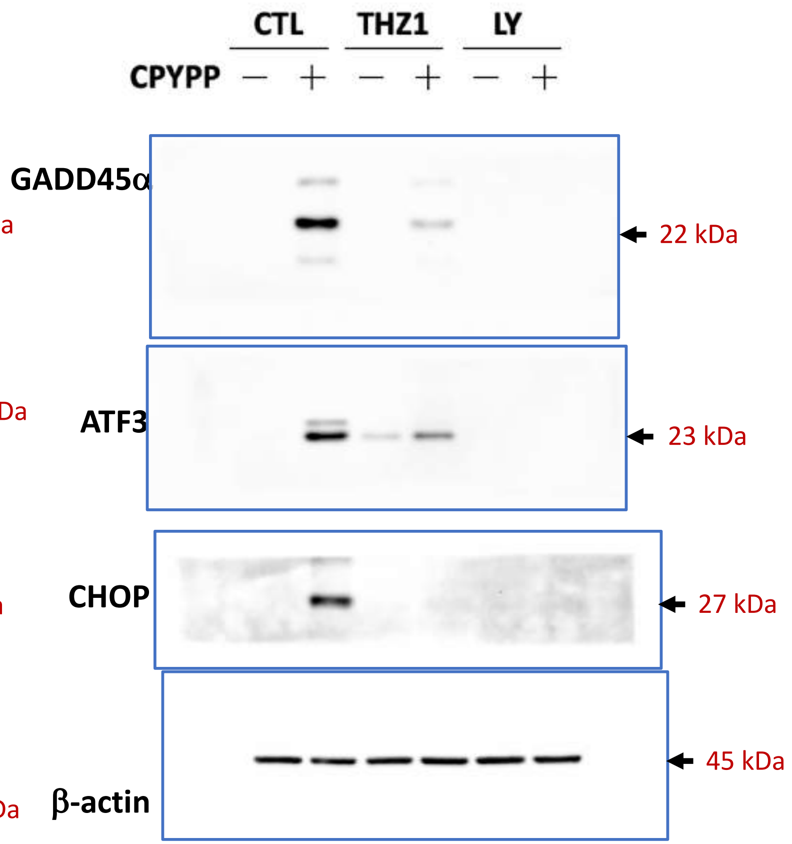

Fig. 3C (right panel)

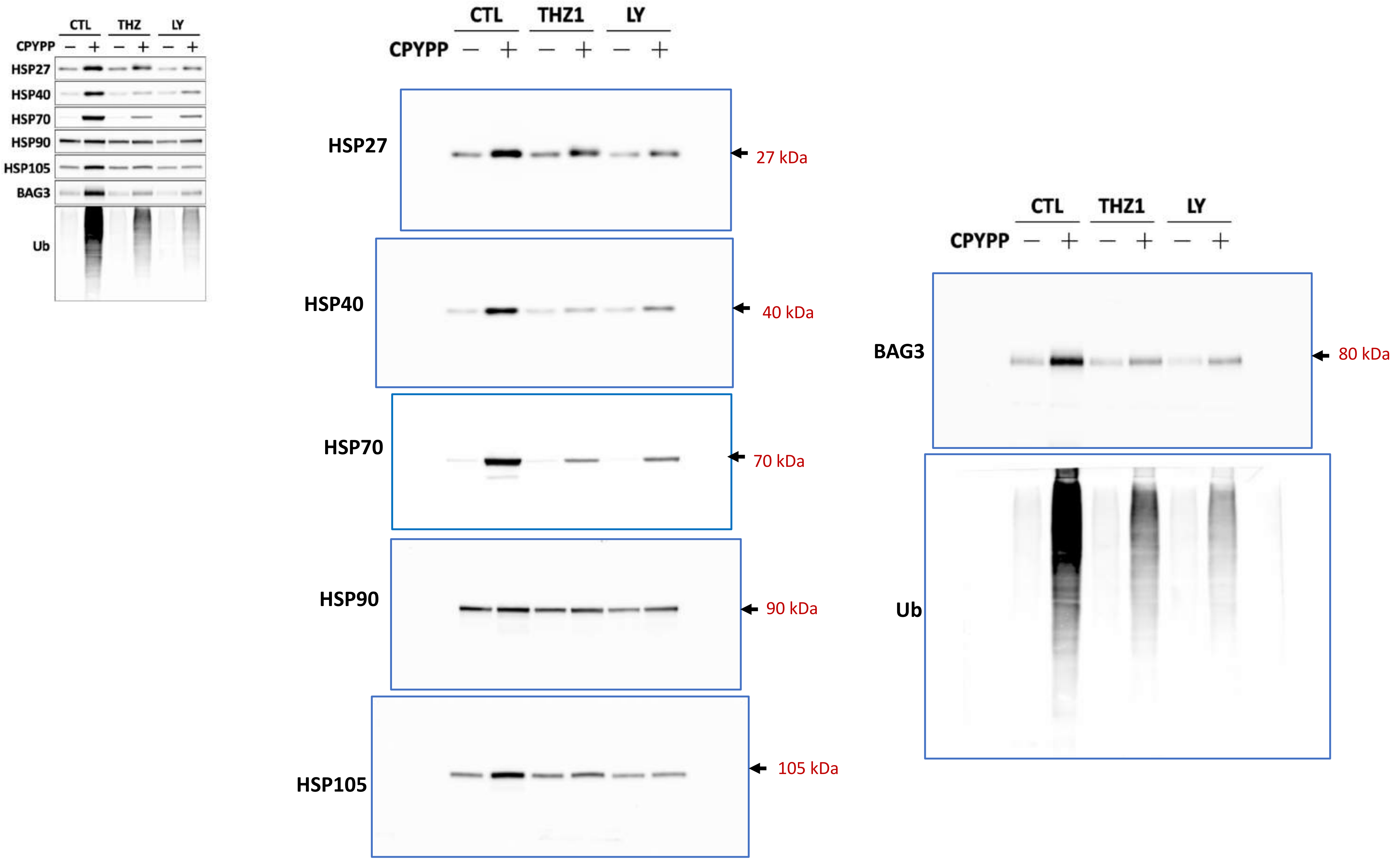

Fig. 3D

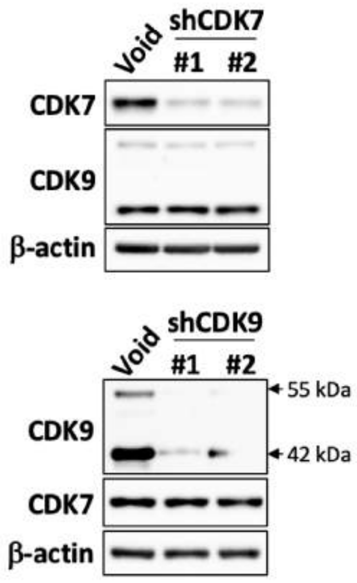

Void
shCDK7
#1
#2
CDK7
CDK9
β-actin
Void
shCDK9
#1
#2
55 kDa
42 kDa
CDK9
CDK7
β-actin

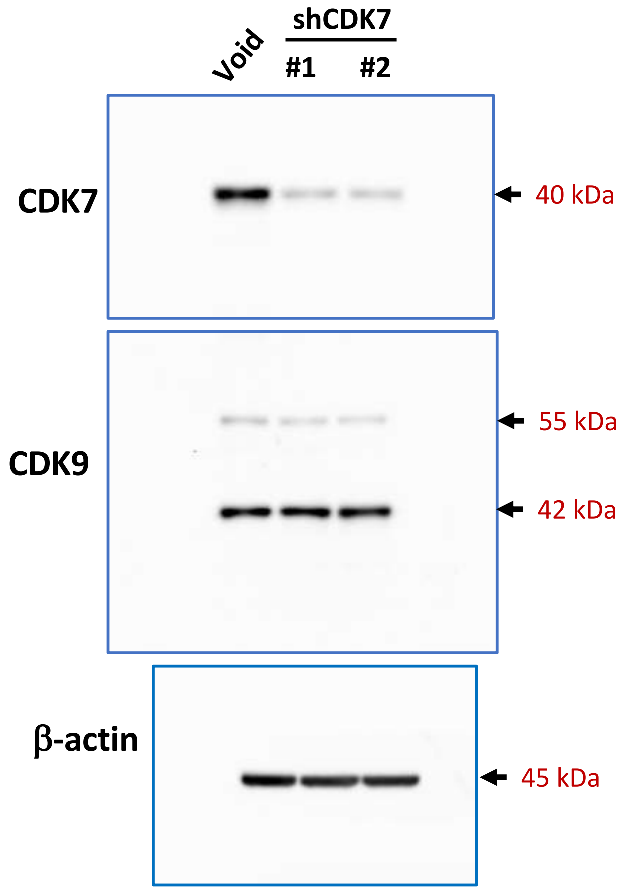

Void
shCDK7
#1
#2
CDK7
40 kDa
CDK9
55 kDa
42 kDa
β-actin
45 kDa

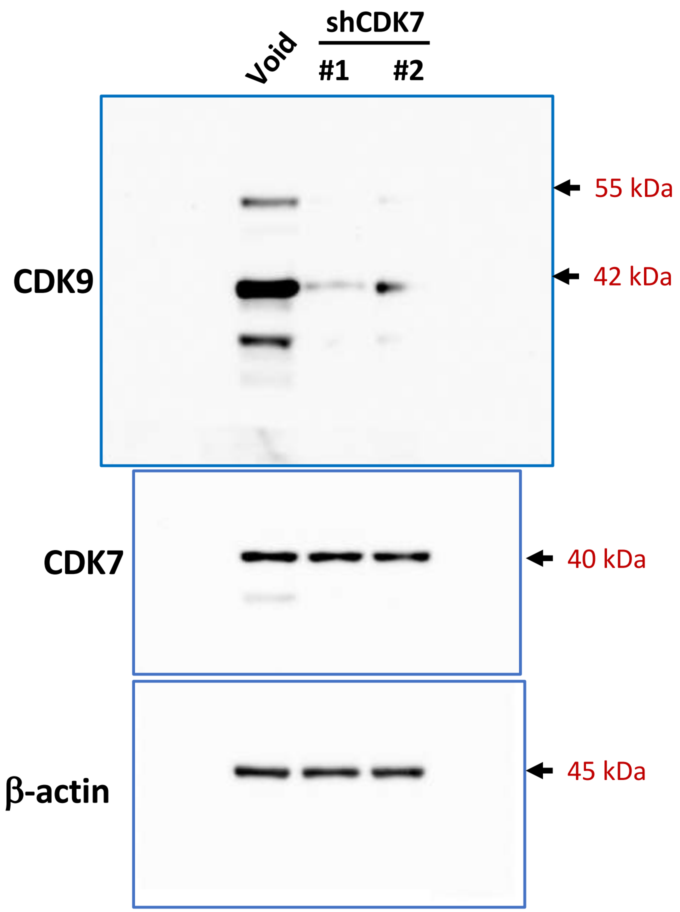

Void
shCDK7
#1
#2
CDK9
55 kDa
42 kDa
CDK7
40 kDa
β-actin
45 kDa

Fig. 3G

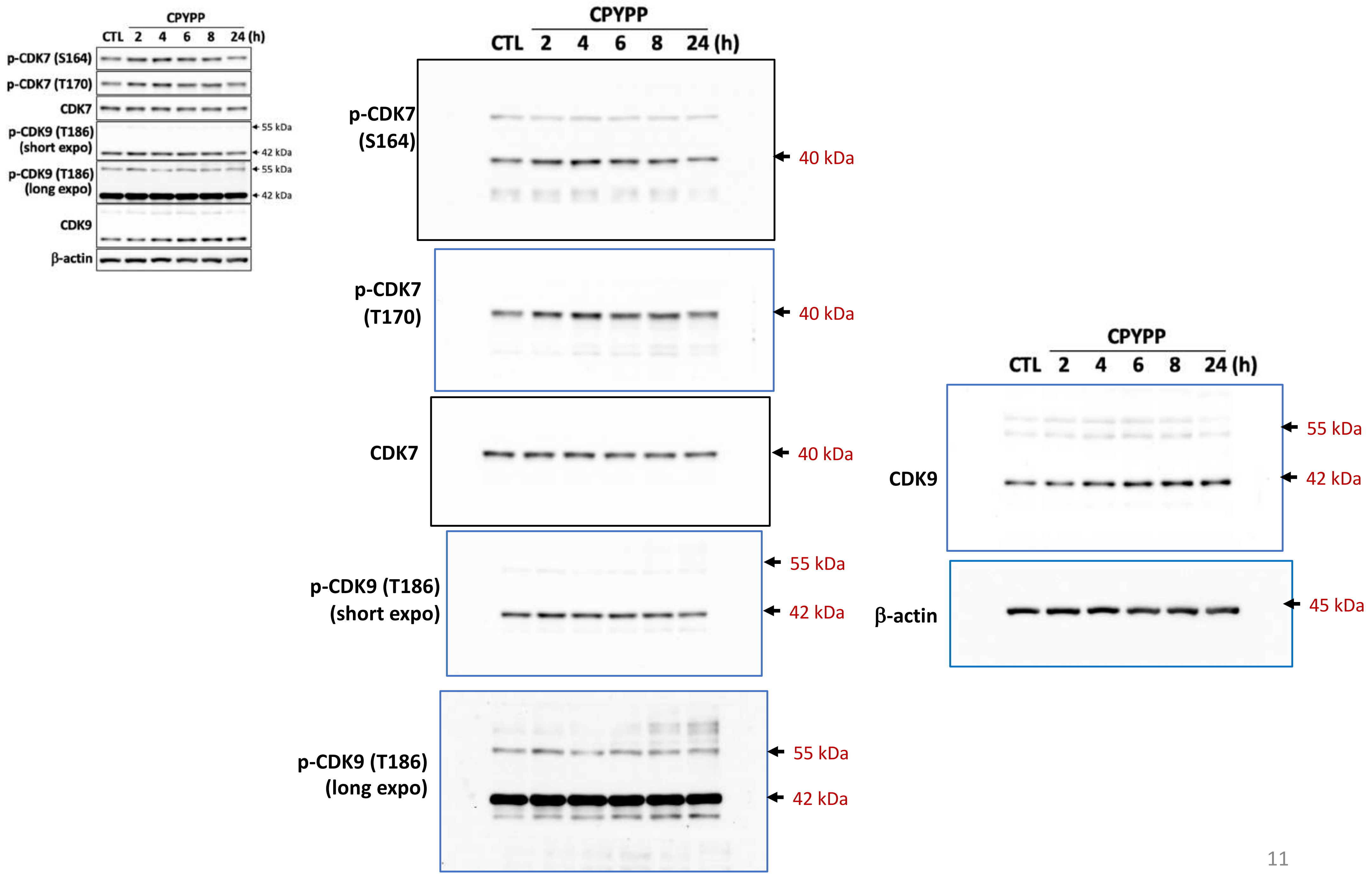
CPYPP
CTL 2 4 6 8 24 (h)
p-CDK7 (S164)
p-CDK7 (T170)
CDK7
p-CDK9 (T186) (short expo)
p-CDK9 (T186) (long expo)
CDK9
β-actin
55 kDa
42 kDa
55 kDa
42 kDa
CPYPP
CTL 2 4 6 8 24 (h)
p-CDK7 (S164)
40 kDa
p-CDK7 (T170)
40 kDa
CDK7
40 kDa
p-CDK9 (T186) (short expo)
55 kDa
42 kDa
p-CDK9 (T186) (long expo)
55 kDa
42 kDa
CPYPP
CTL 2 4 6 8 24 (h)
CDK9
55 kDa
42 kDa
β-actin
45 kDa

Fig. 3I

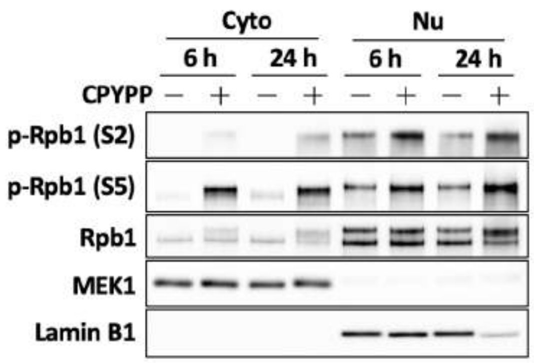

Cyto
Nu
6 h
24 h
6 h
24 h
CPYPP – + – + – + – +
p-Rpb1 (S2)
p-Rpb1 (S5)
Rpb1
MEK1
Lamin B1

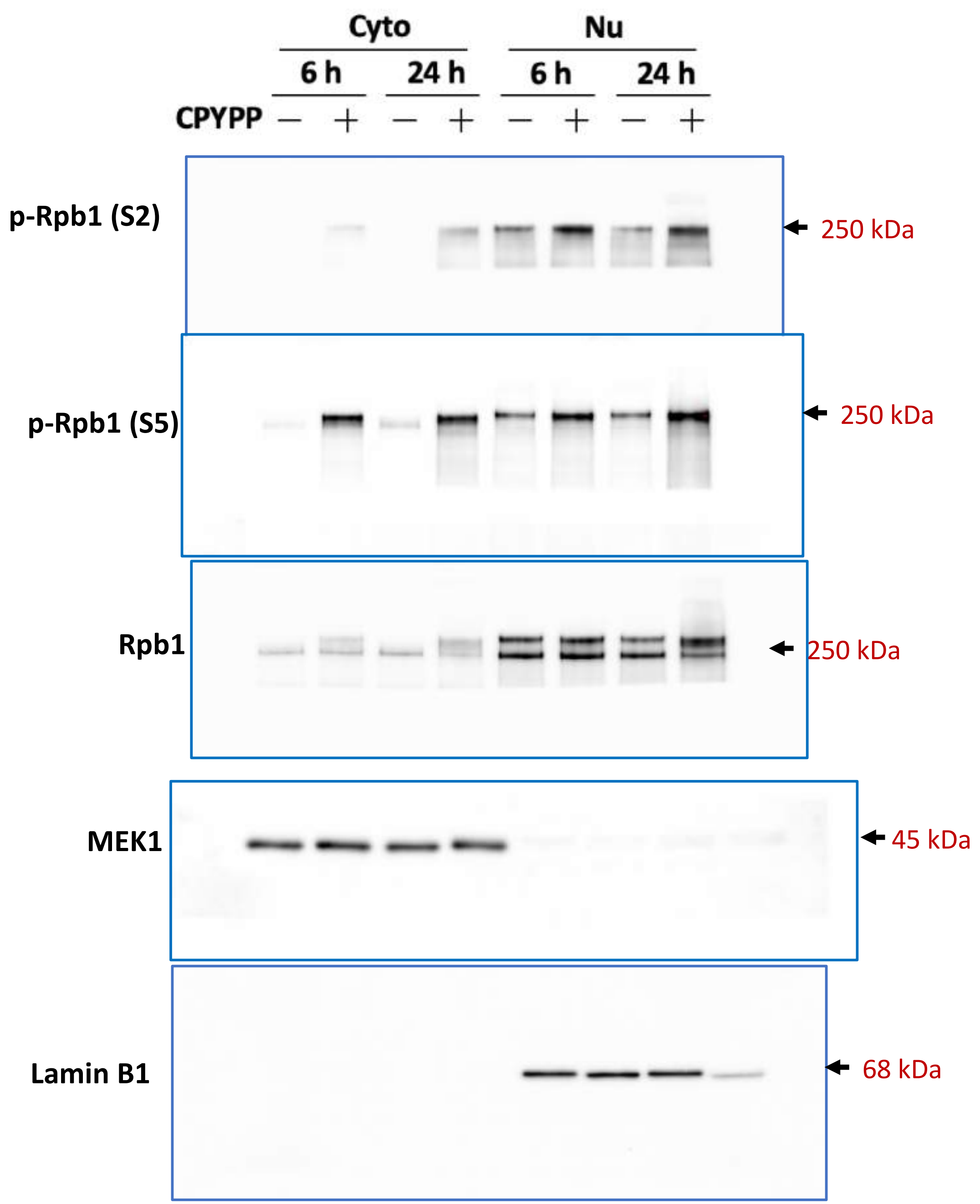

Cyto
Nu
6 h
24 h
6 h
24 h
CPYPP – + – + – + – +
p-Rpb1 (S2)
250 kDa
p-Rpb1 (S5)
250 kDa
Rpb1
250 kDa
MEK1
45 kDa
Lamin B1
68 kDa

Fig. 3J

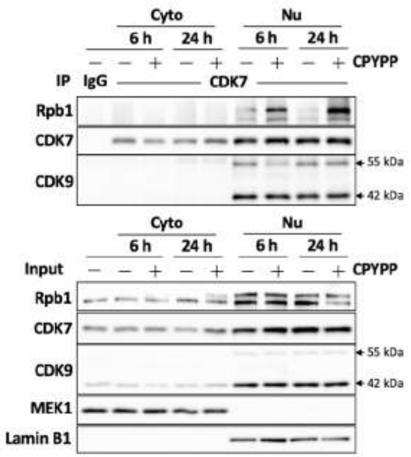
Cyto
Nu
6 h
24 h
6 h
24 h
CPYPP
IP
IgG
CDK7
Rpb1
CDK7
CDK9
55 kDa
42 kDa
Input
MEK1
Lamin B1

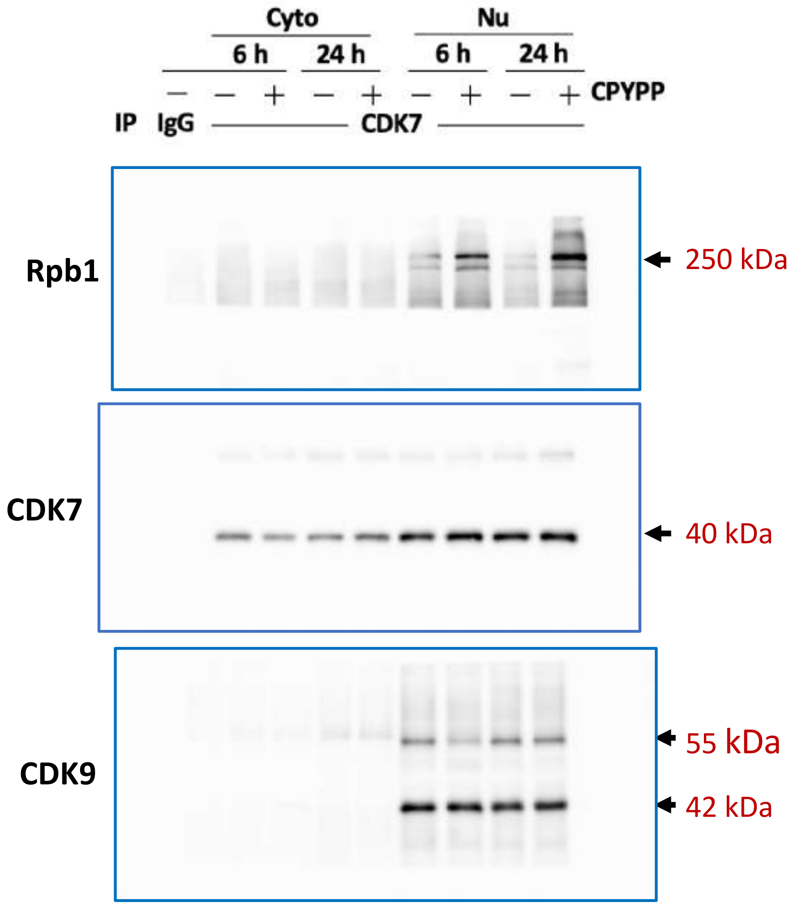
Cyto
Nu
6 h
24 h
6 h
24 h
CPYPP
IP
IgG
CDK7
Rpb1
250 kDa
CDK7
40 kDa
CDK9
55 kDa
42 kDa

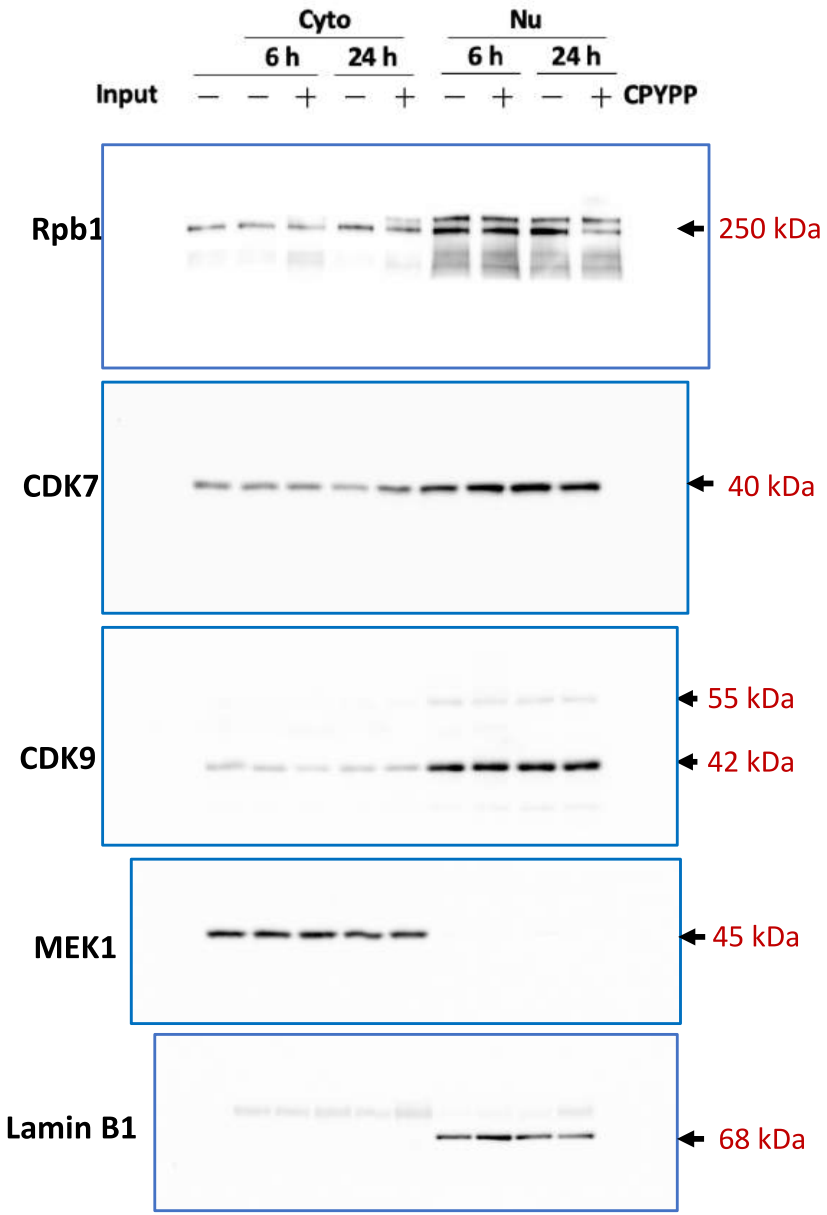
Cyto
Nu
6 h
24 h
6 h
24 h
Input
CPYPP
Rpb1
250 kDa
CDK7
40 kDa
CDK9
55 kDa
42 kDa
MEK1
45 kDa
Lamin B1
68 kDa

Fig. 3N

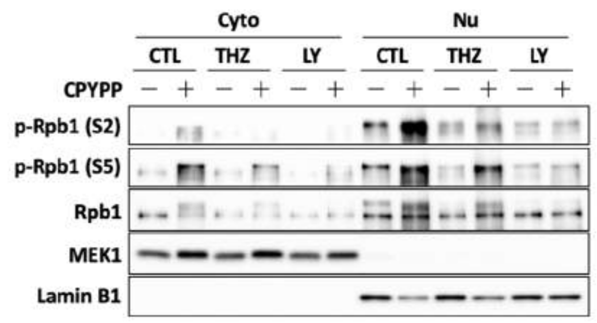
Cyto
Nu
CTL
THZ
LY
CTL
THZ
LY
CPYPP
p-Rpb1 (S2)
p-Rpb1 (S5)
Rpb1
MEK1
Lamin B1

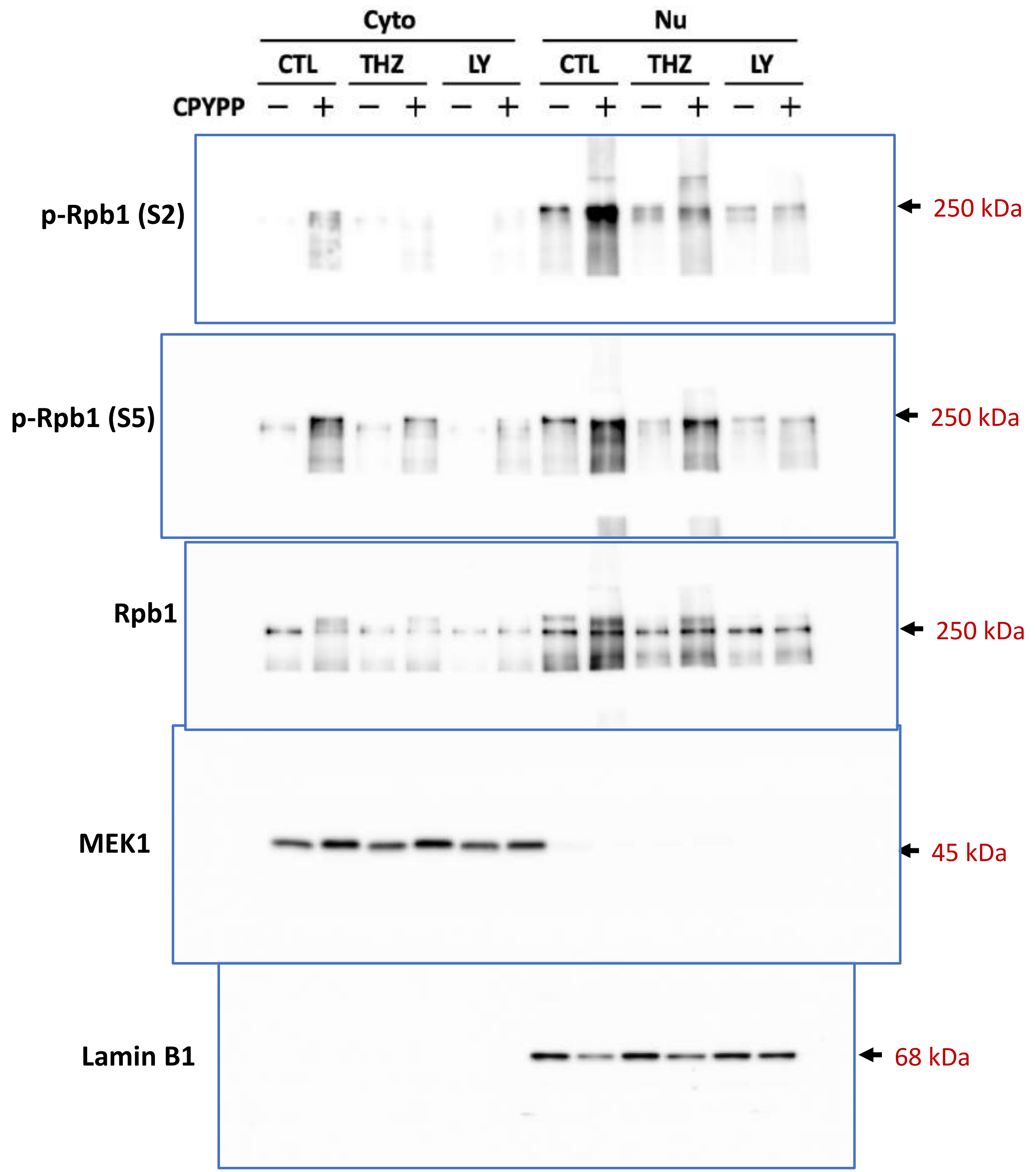
Cyto
Nu
CTL
THZ
LY
CTL
THZ
LY
CPYPP
p-Rpb1 (S2)
250 kDa
p-Rpb1 (S5)
250 kDa
Rpb1
250 kDa
MEK1
45 kDa
Lamin B1
68 kDa

Fig. 3O

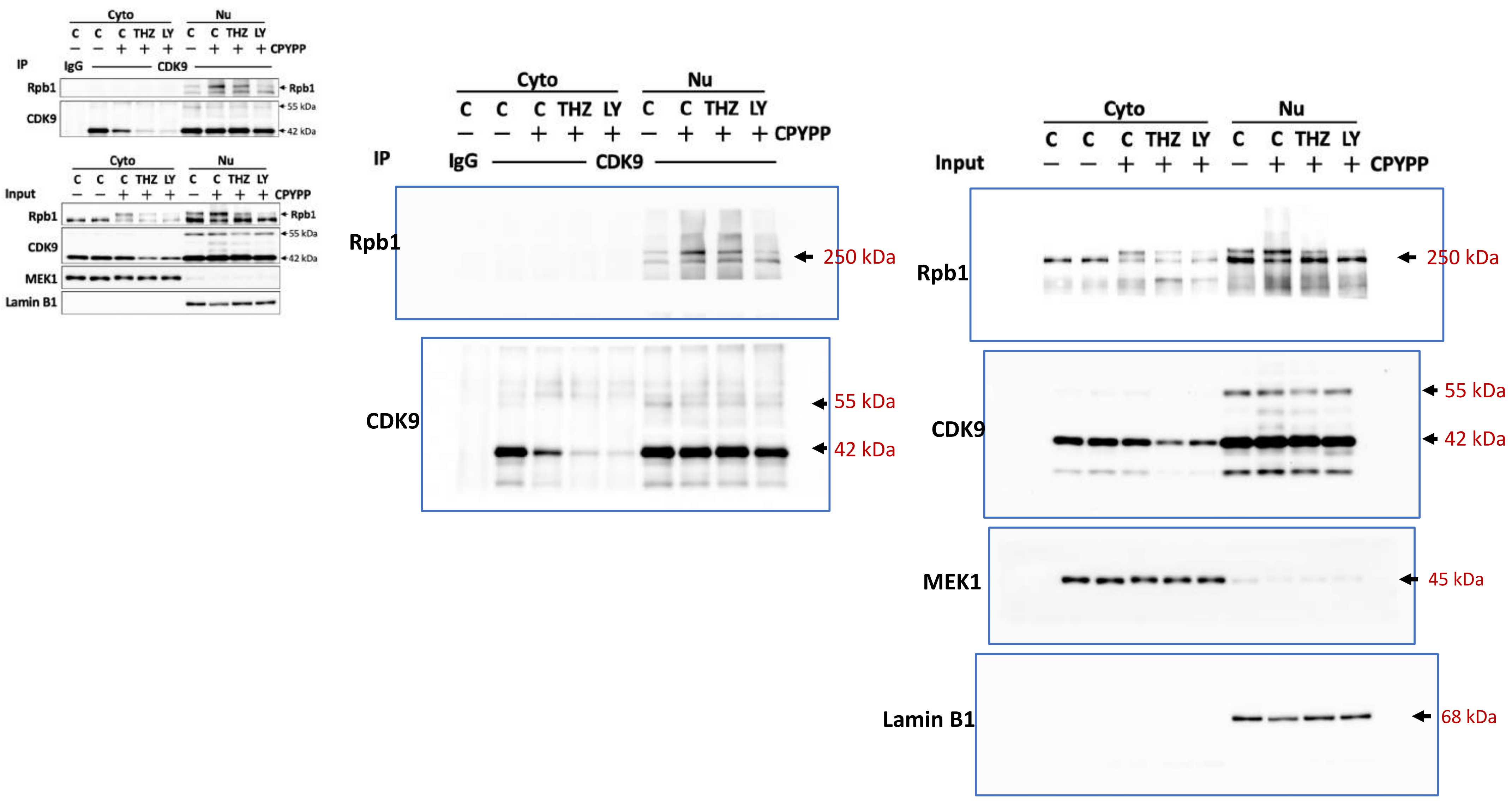
Cyto
Nu
C C C THZ LY C C THZ LY
– – + + + – + + + CPYPP
IP
IgG
CDK9
Rpb1
250 kDa
55 kDa
42 kDa
Input
MEK1
45 kDa
Lamin B1
68 kDa

Fig. 4A

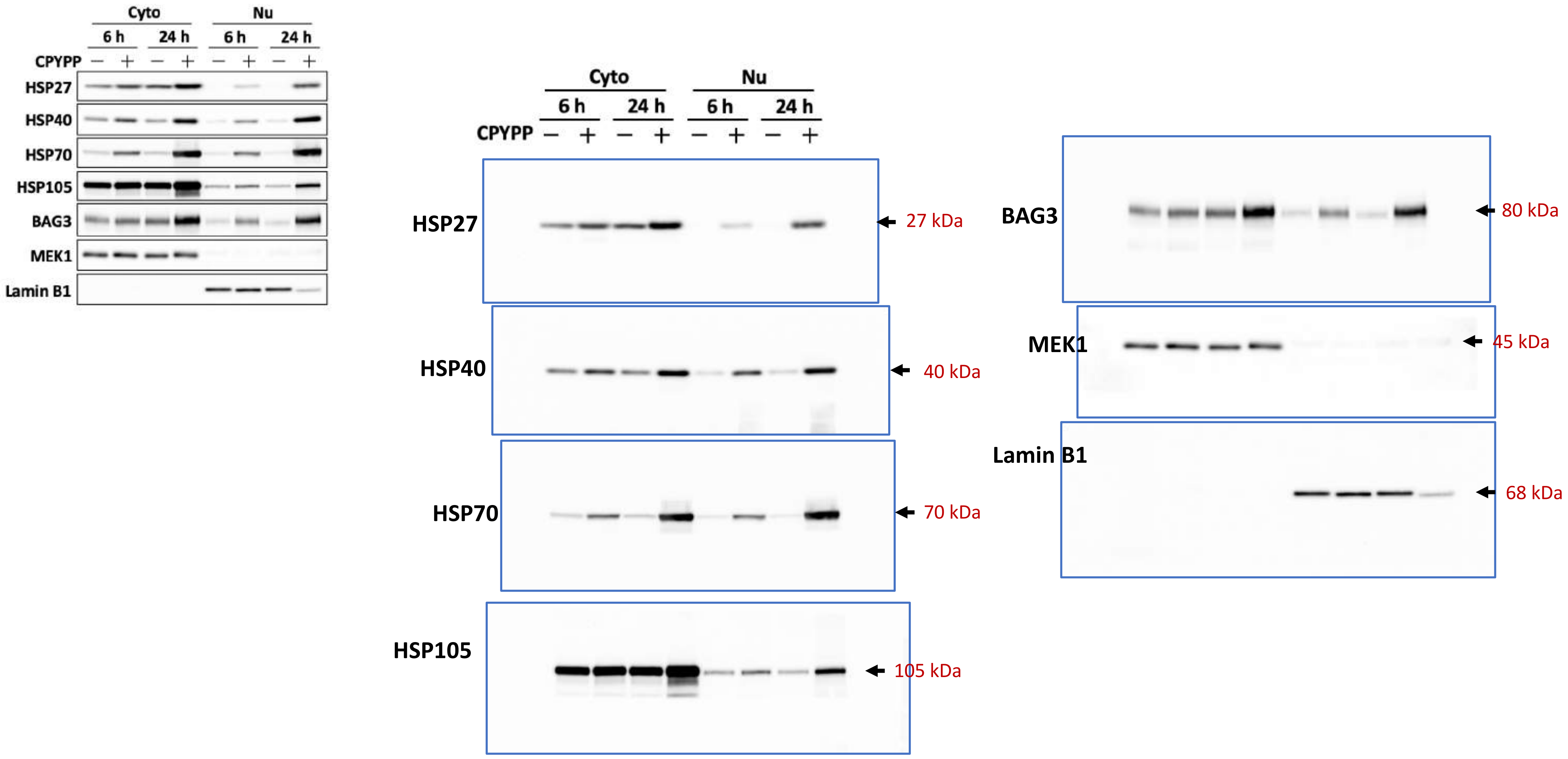
Cyto
Nu
6 h
24 h
6 h
24 h
CPYPP − + − + − + − +
HSP27
HSP40
HSP70
HSP105
BAG3
MEK1
Lamin B1
27 kDa
40 kDa
70 kDa
105 kDa
80 kDa
45 kDa
68 kDa

Fig. 4B

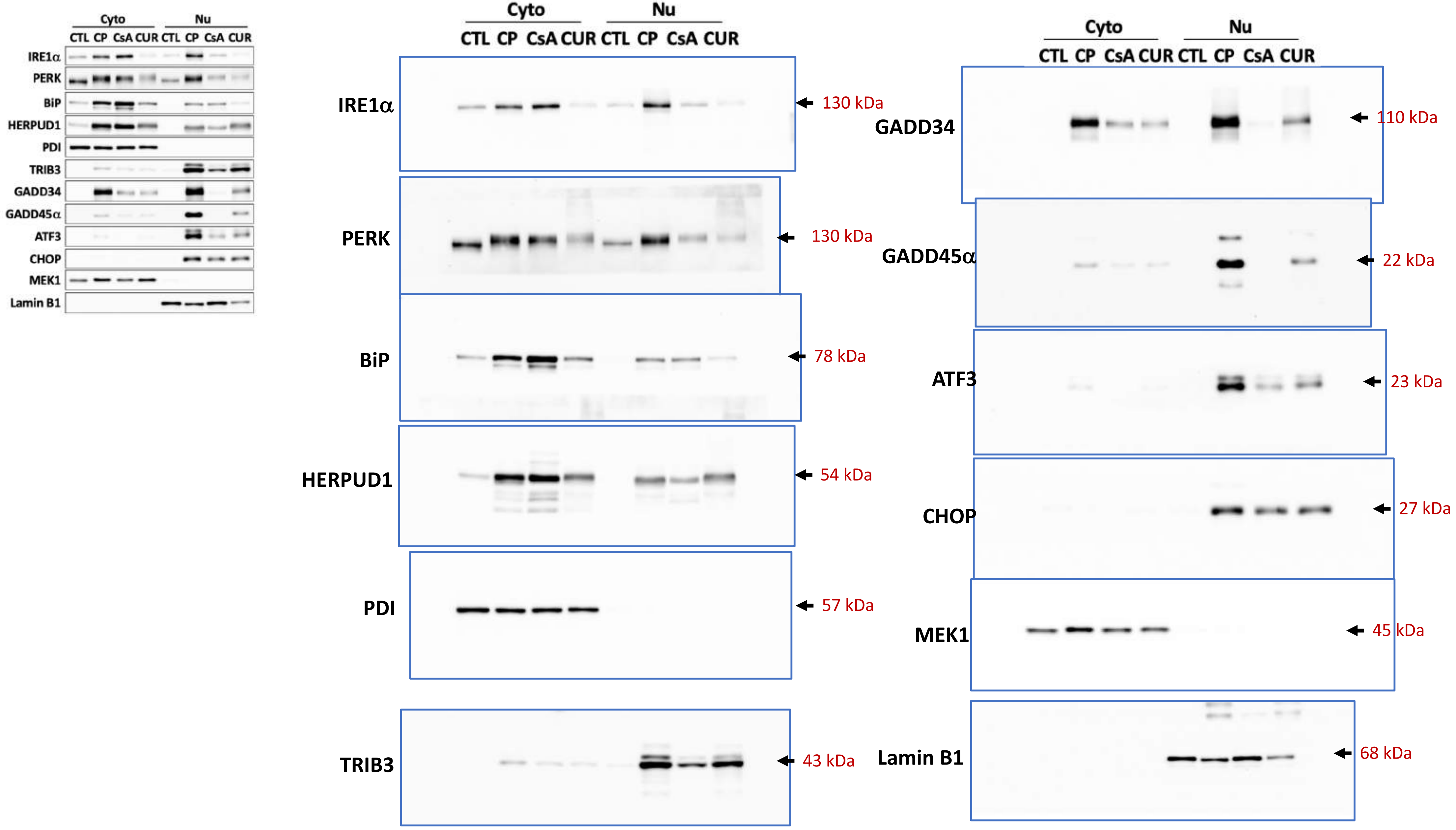

Cyto
Nu
CTL CP CsA CUR CTL CP CsA CUR
IRE1α
130 kDa
PERK
130 kDa
BiP
78 kDa
HERPUD1
54 kDa
PDI
57 kDa
TRIB3
43 kDa
GADD34
110 kDa
GADD45α
22 kDa
ATF3
23 kDa
CHOP
27 kDa
MEK1
45 kDa
Lamin B1
68 kDa

Fig. 4D

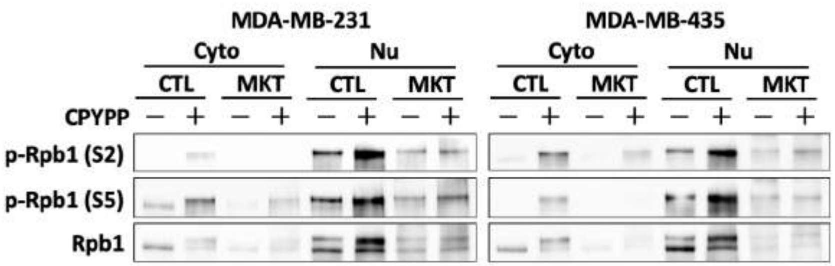
MDA-MB-231
MDA-MB-435
Cyto
Nu
CTL
MKT
CPYPP
p-Rpb1 (S2)
p-Rpb1 (S5)
Rpb1

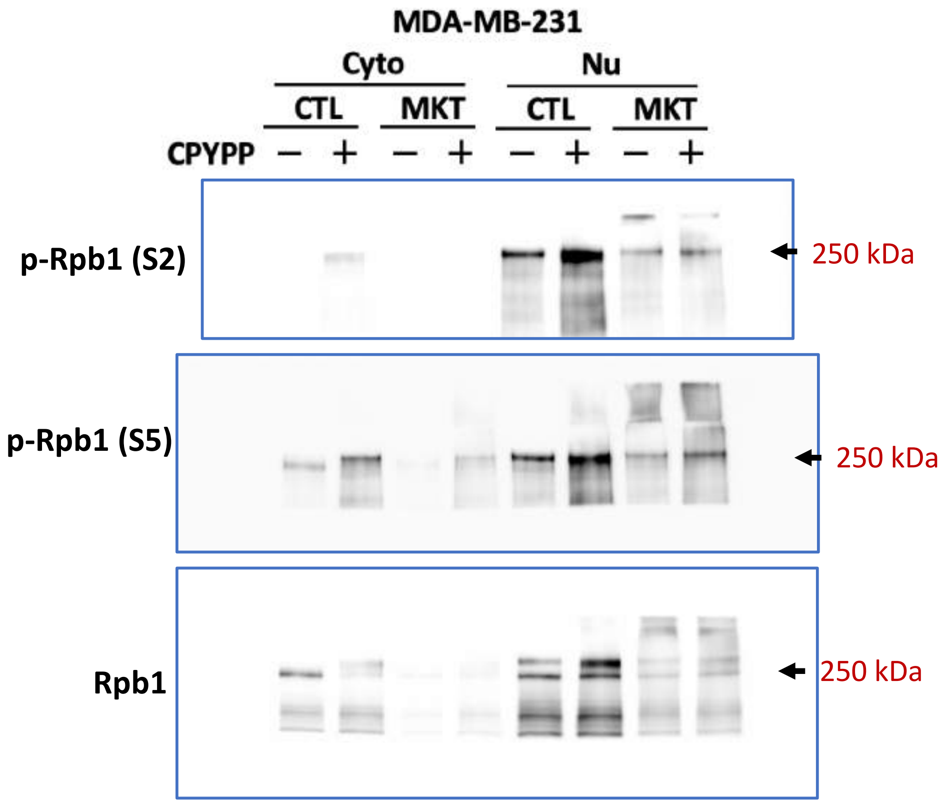
MDA-MB-231
Cyto
Nu
CTL
MKT
CPYPP
p-Rpb1 (S2)
p-Rpb1 (S5)
Rpb1
250 kDa

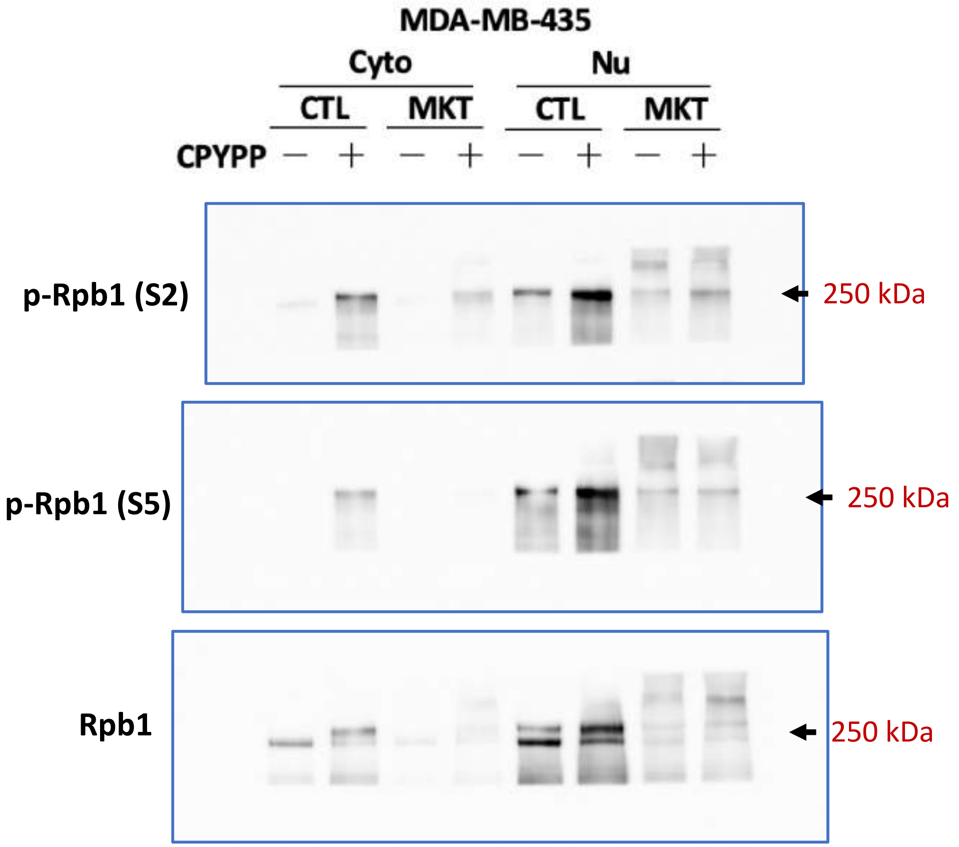
MDA-MB-435
Cyto
Nu
CTL
MKT
CPYPP
p-Rpb1 (S2)
p-Rpb1 (S5)
Rpb1
250 kDa

Fig. 4E

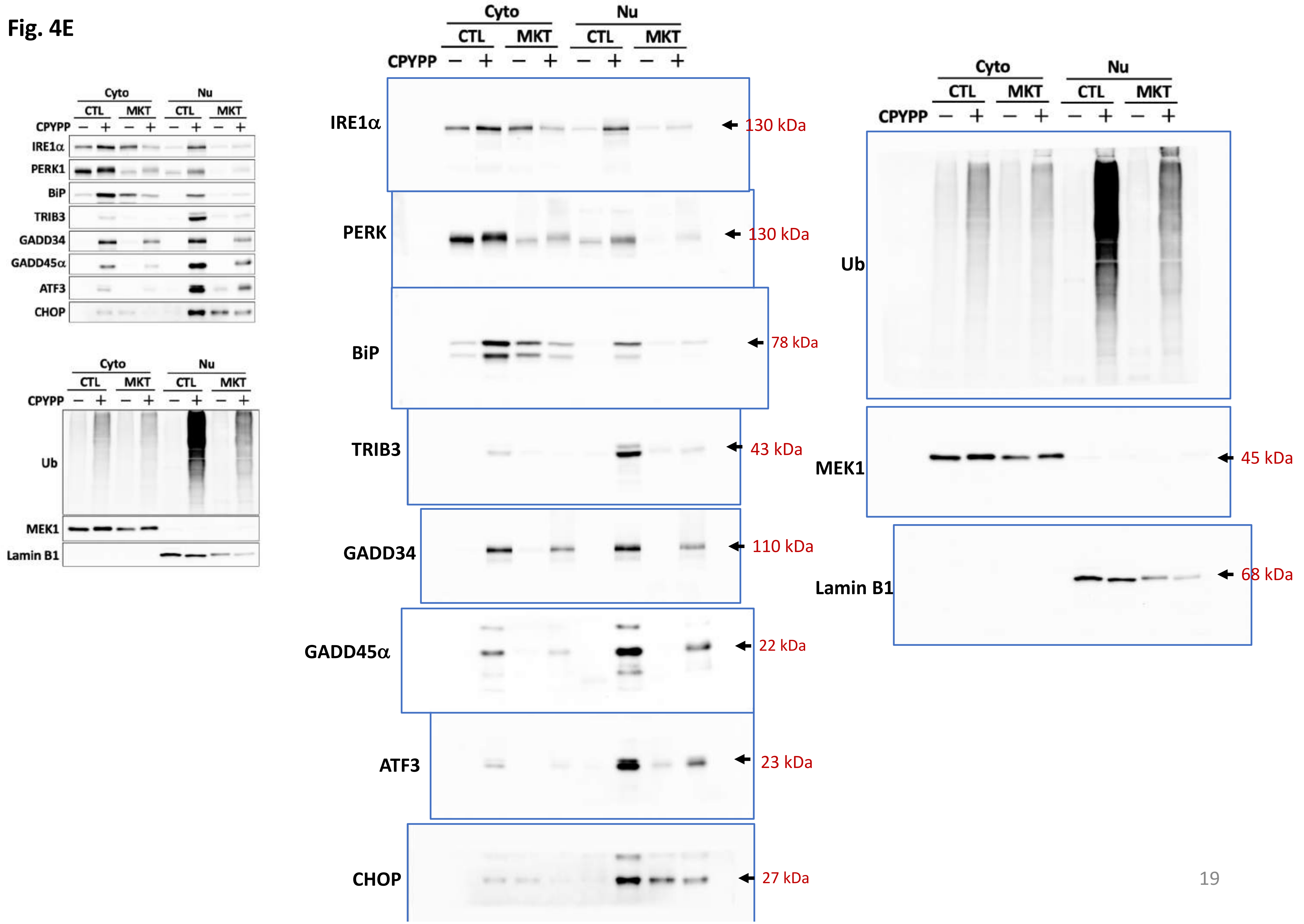
Cyto
Nu
CTL
MKT
CPYPP
IRE1α
PERK1
BiP
TRIB3
GADD34
GADD45α
ATF3
CHOP
Ub
MEK1
Lamin B1
PERK
130 kDa
130 kDa
78 kDa
43 kDa
110 kDa
22 kDa
23 kDa
27 kDa
45 kDa
68 kDa

Fig. 4K (left panel)

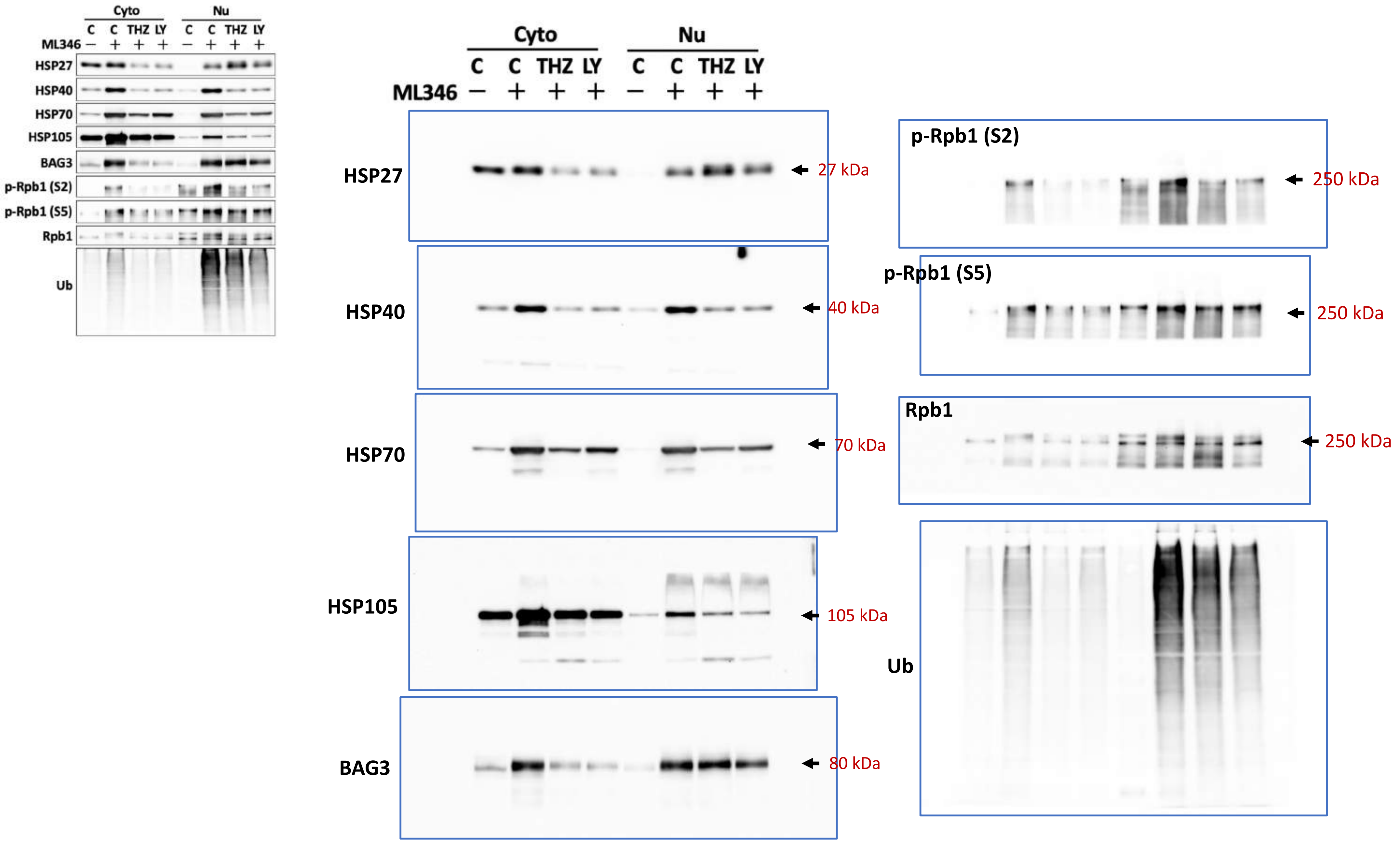

Fig. 4K (right panel)

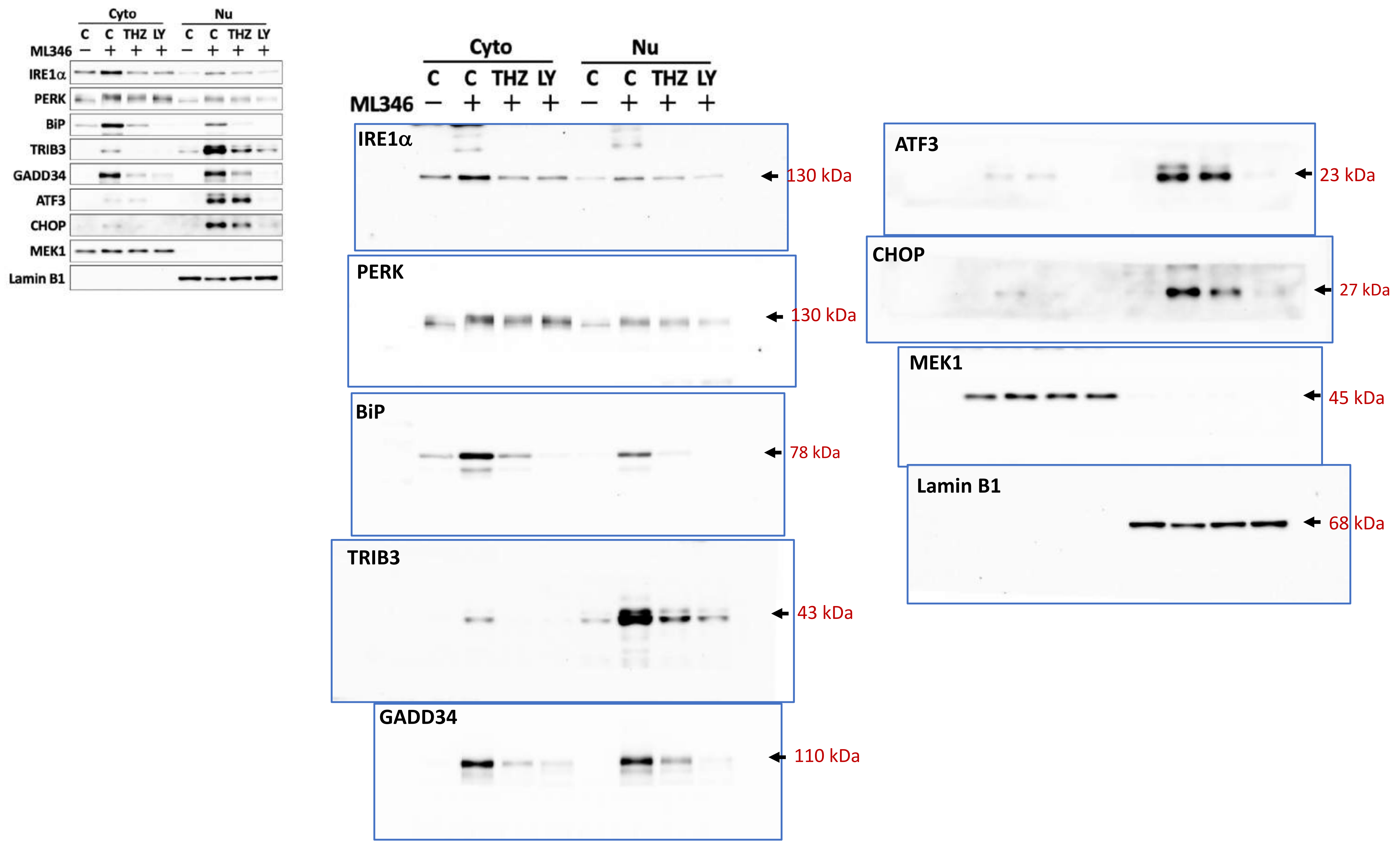

Fig. 4N (upper panel)

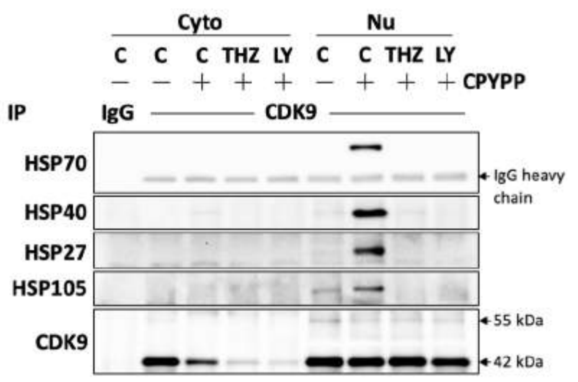

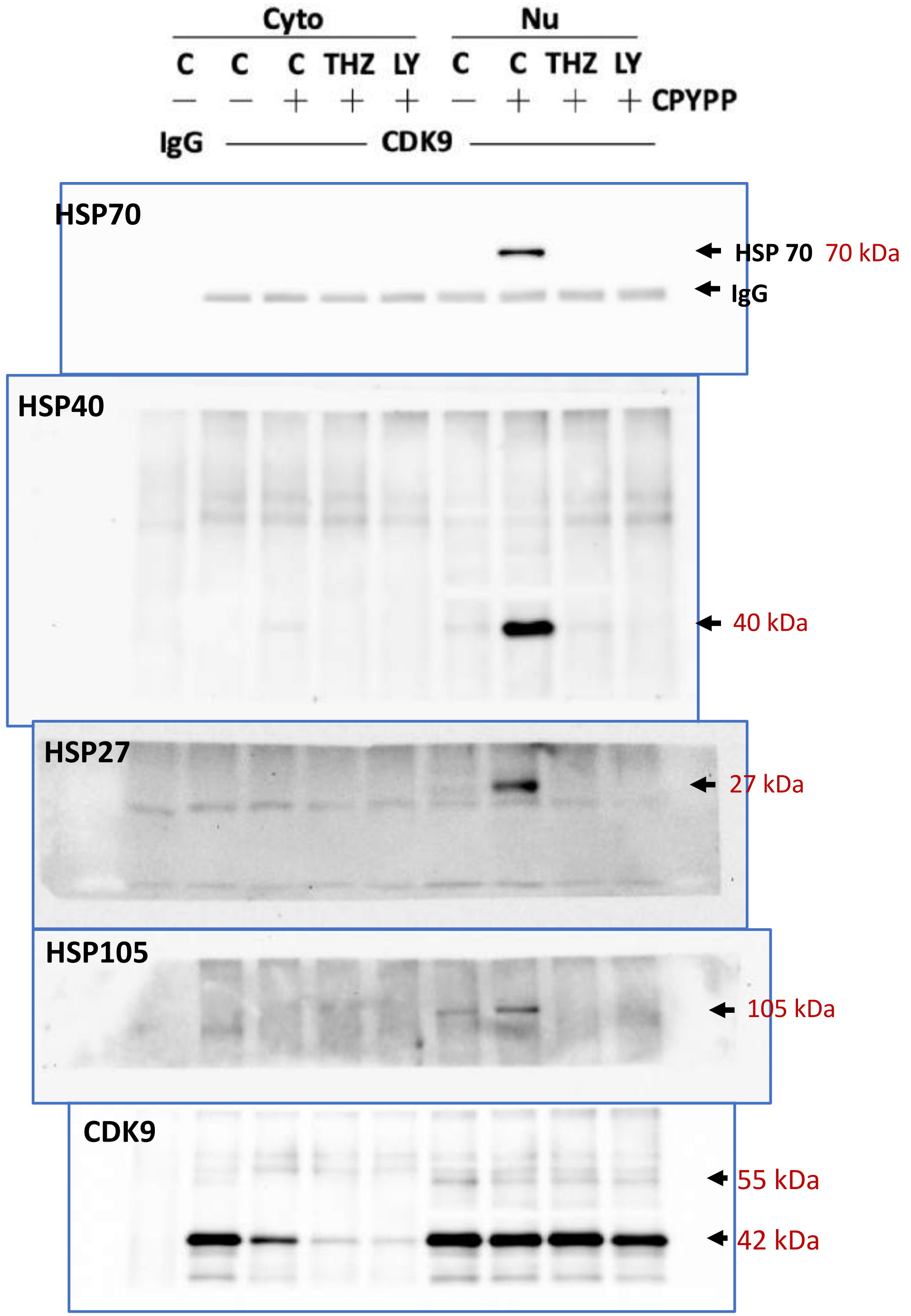

Fig. 4N (lower panel)

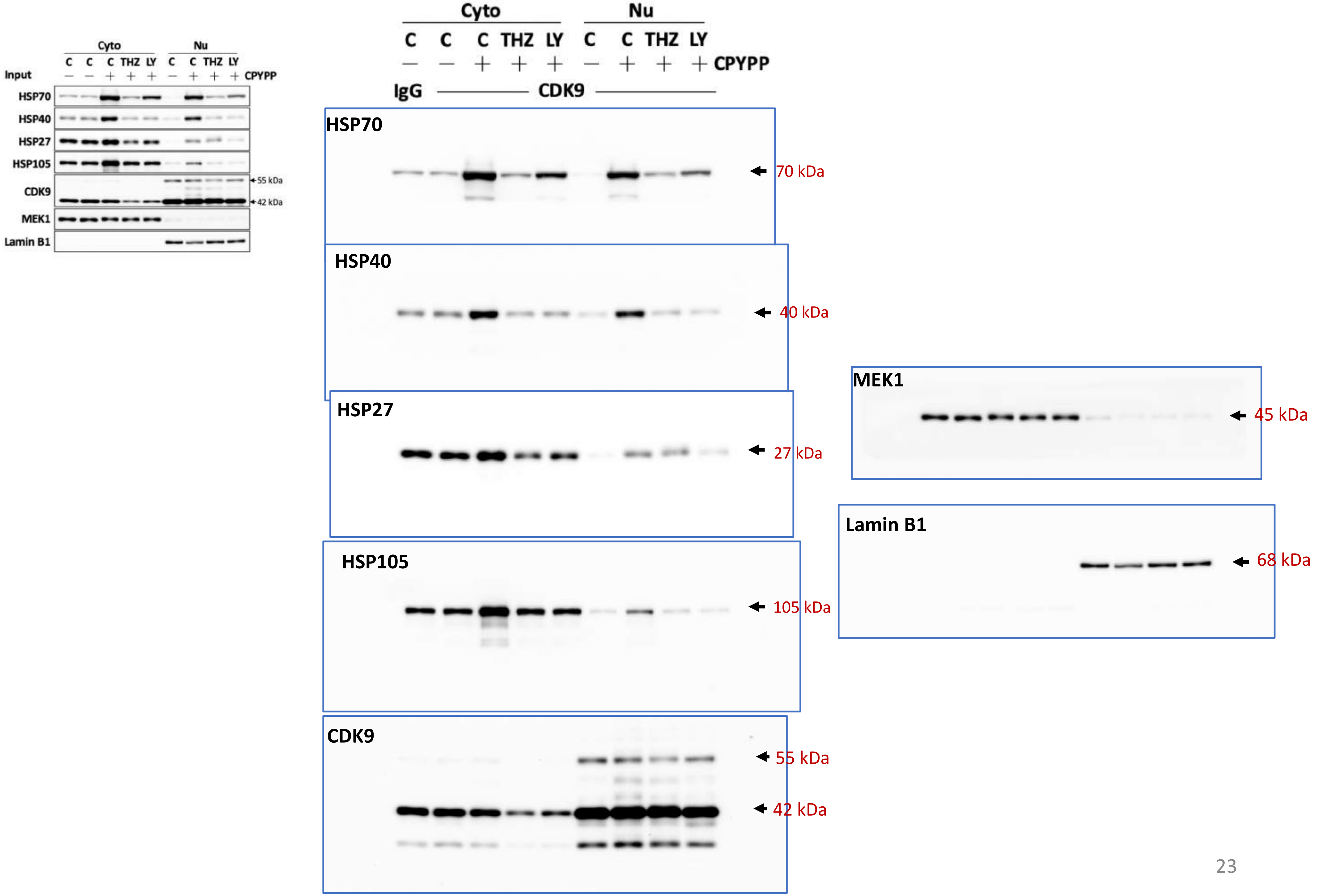

Fig. 5C

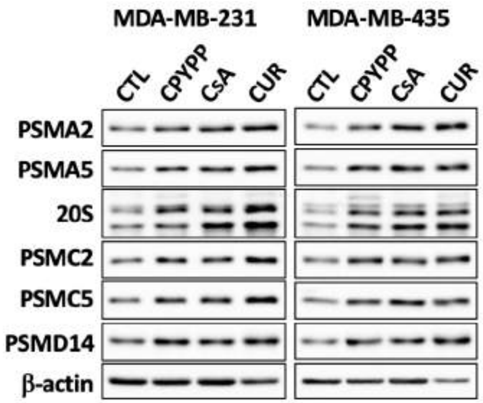
MDA-MB-231
MDA-MB-435
CTL
CPYPP
CsA
CUR
CTL
CPYPP
CsA
CUR
PSMA2
PSMA5
20S
PSMC2
PSMC5
PSMD14
β-actin

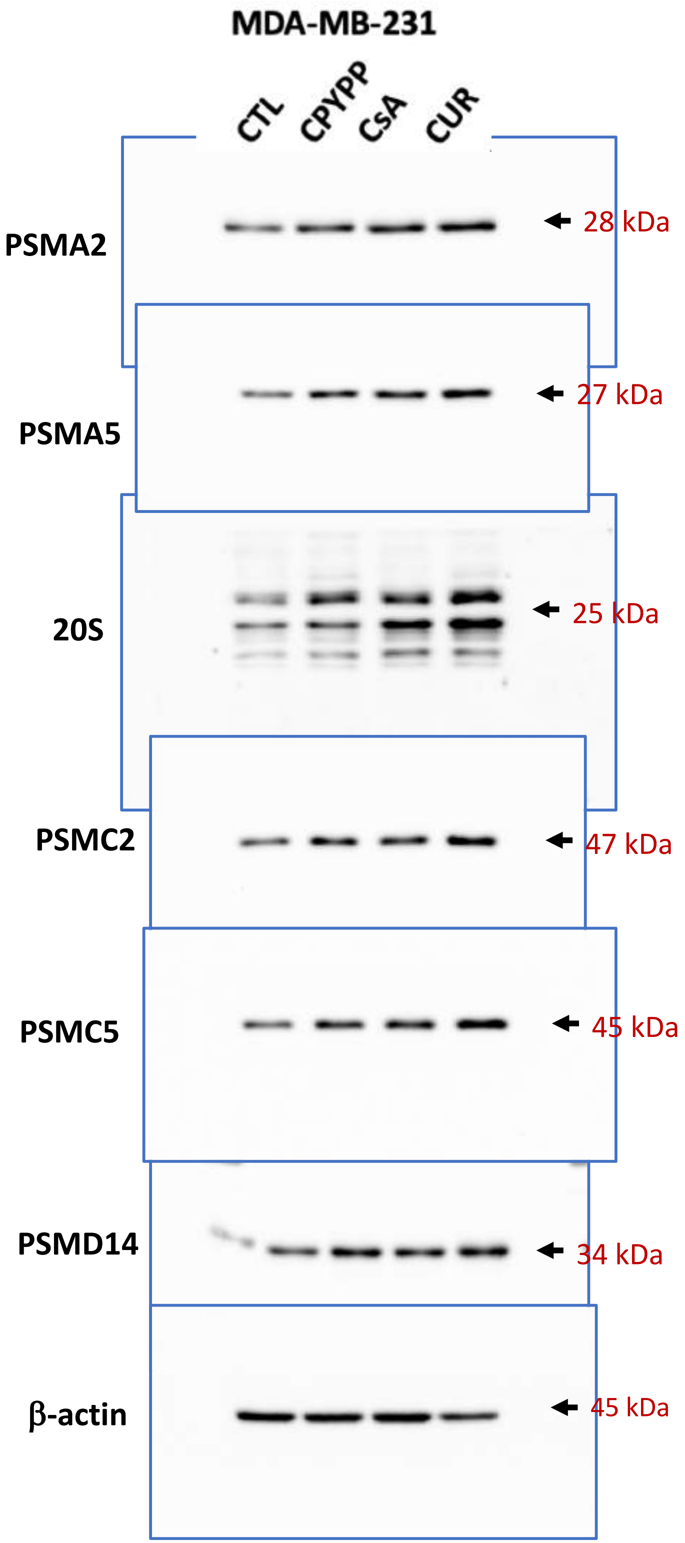
MDA-MB-231
CTL
CPYPP
CsA
CUR
PSMA2
28 kDa
PSMA5
27 kDa
20S
25 kDa
PSMC2
47 kDa
PSMC5
45 kDa
PSMD14
34 kDa
β-actin
45 kDa

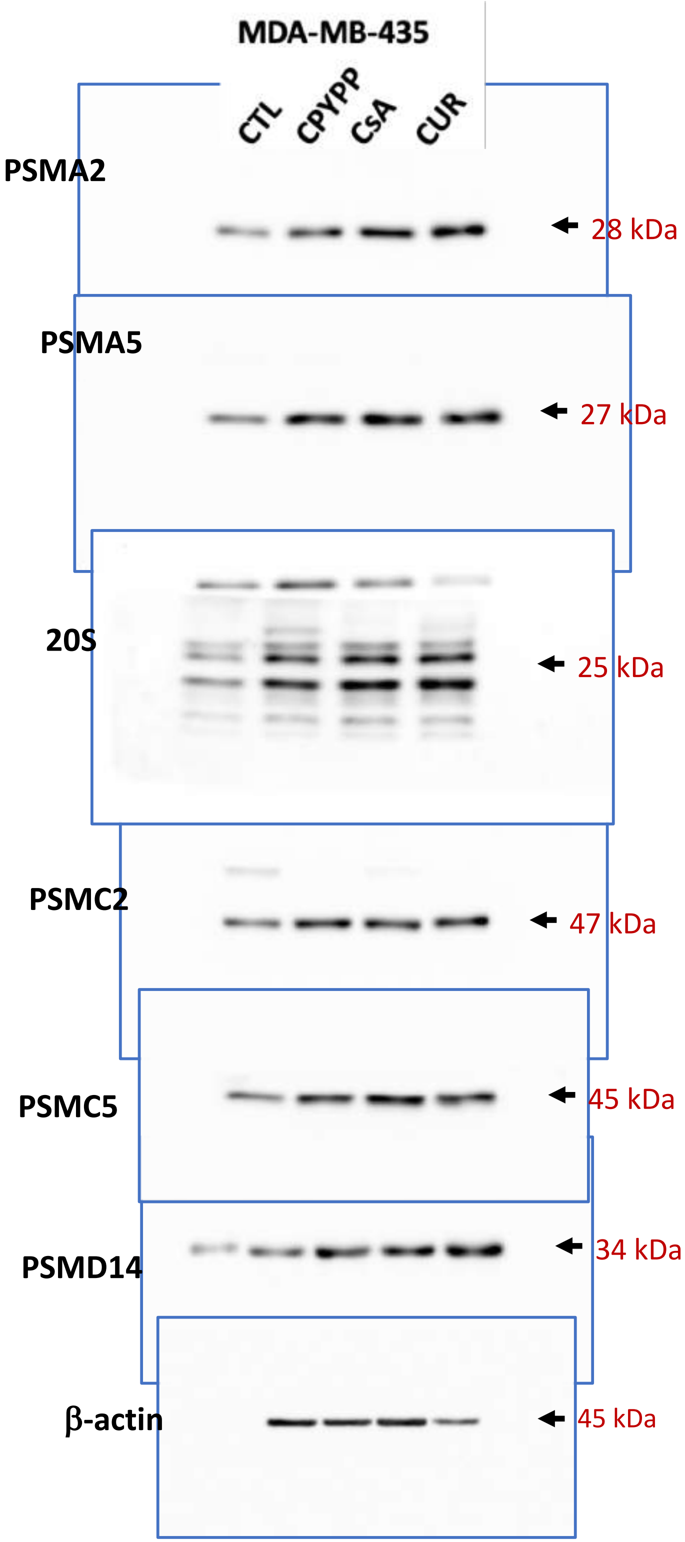
MDA-MB-435
CTL
CPYPP
CsA
CUR
PSMA2
28 kDa
PSMA5
27 kDa
20S
25 kDa
PSMC2
47 kDa
PSMC5
45 kDa
PSMD14
34 kDa
β-actin
45 kDa

Fig. 5H (left panel)

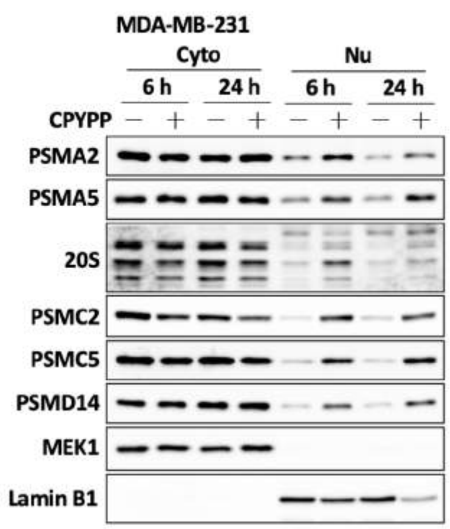

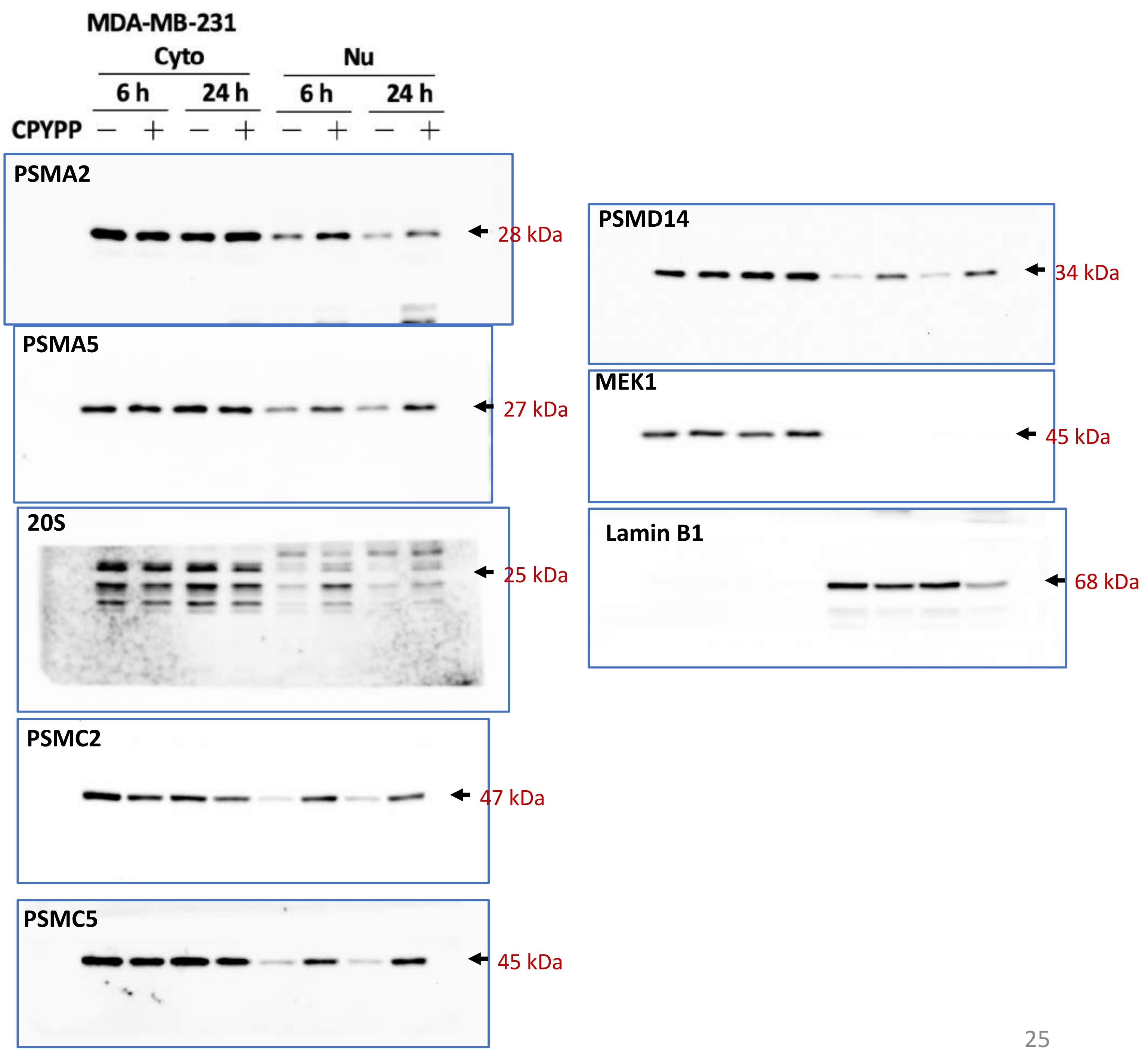

Fig. 5H (right panel)

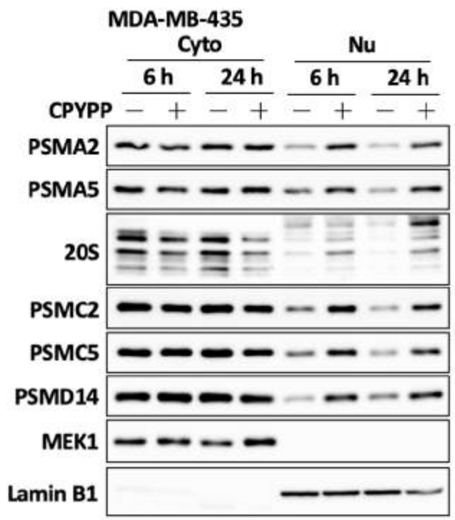

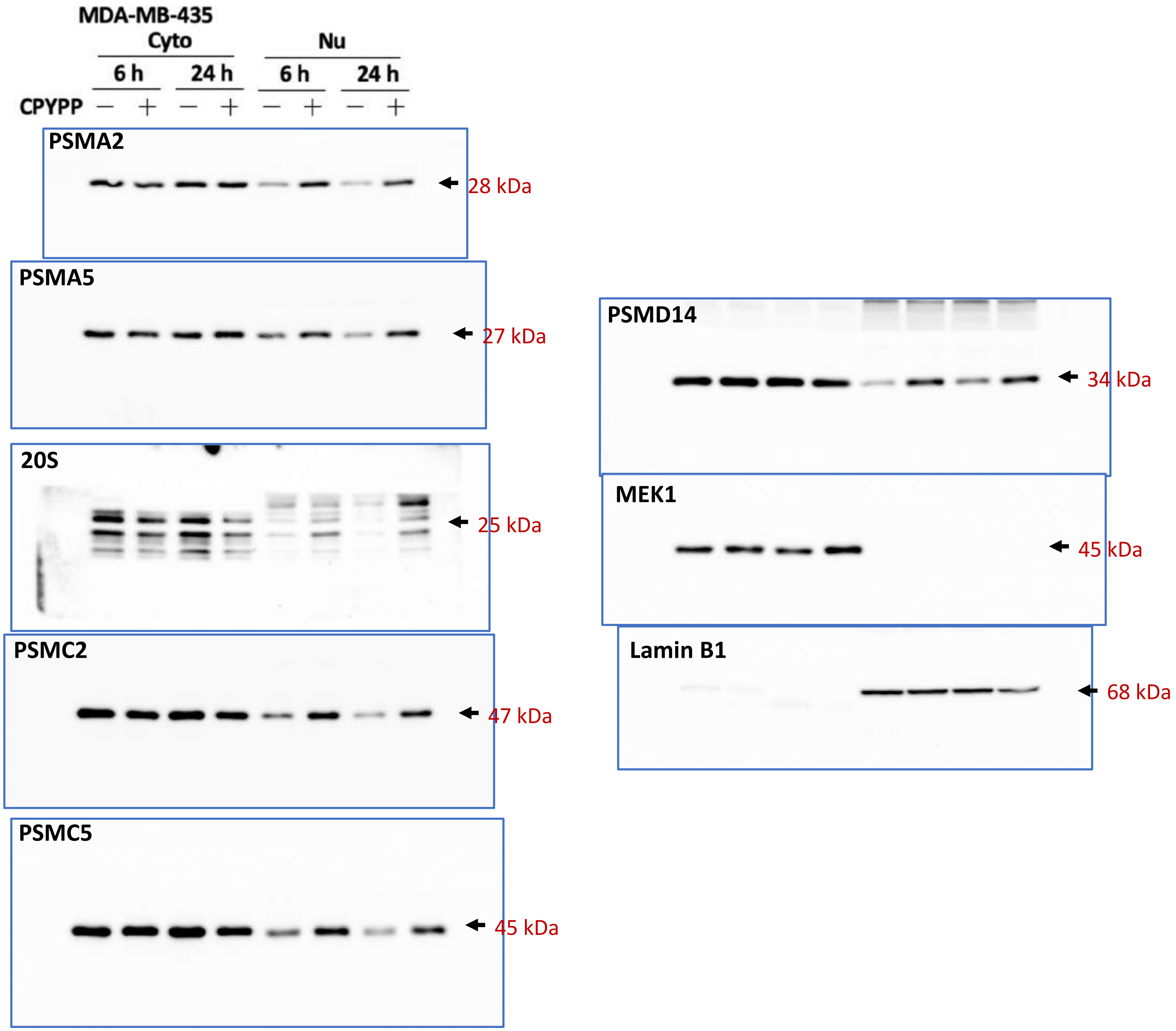

Fig. 5J

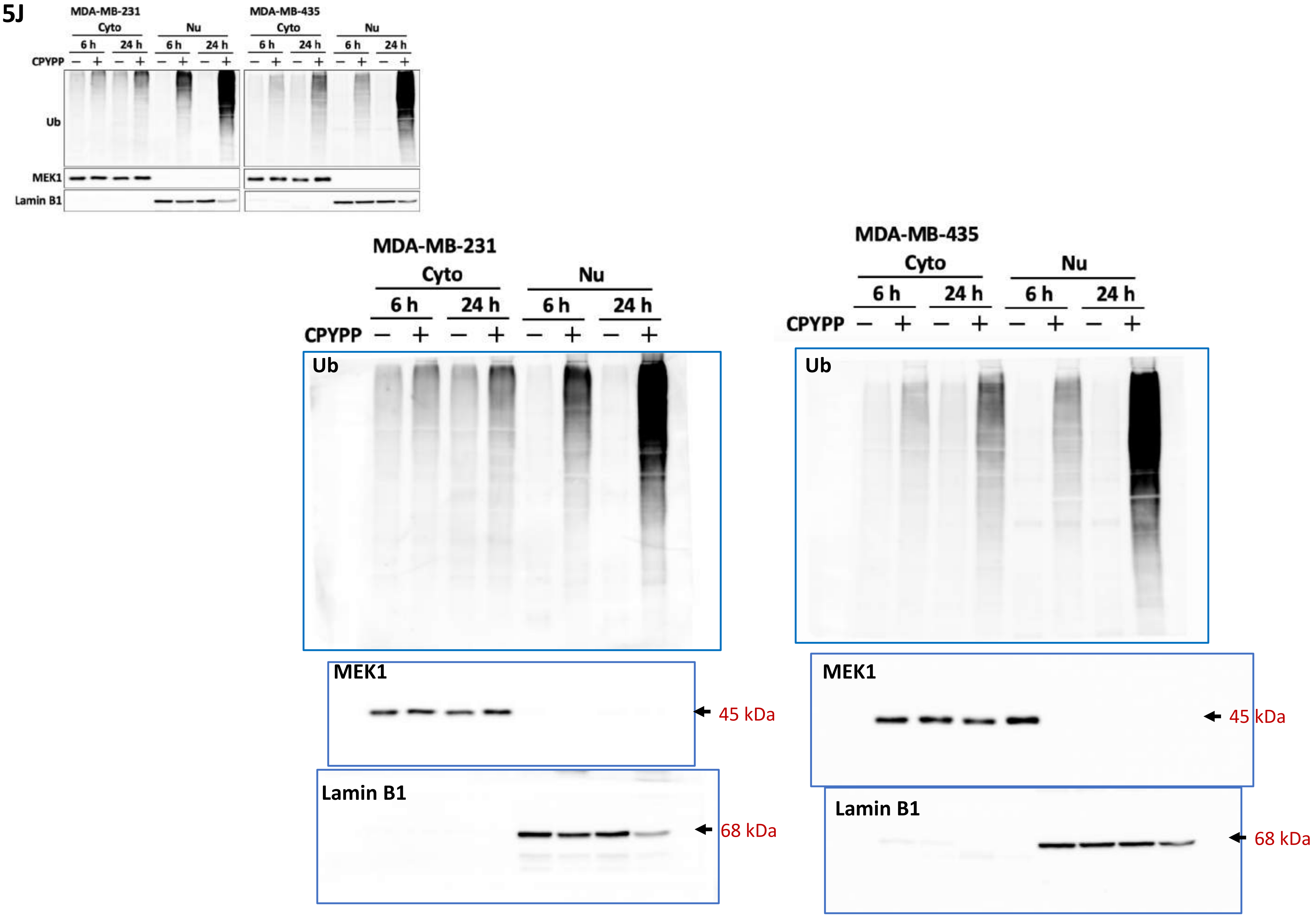

MDA-MB-231
Cyto
Nu
6 h
24 h
CPYPP
Ub
MEK1
Lamin B1
MDA-MB-435
45 kDa
68 kDa

Fig. 6C (left panel)

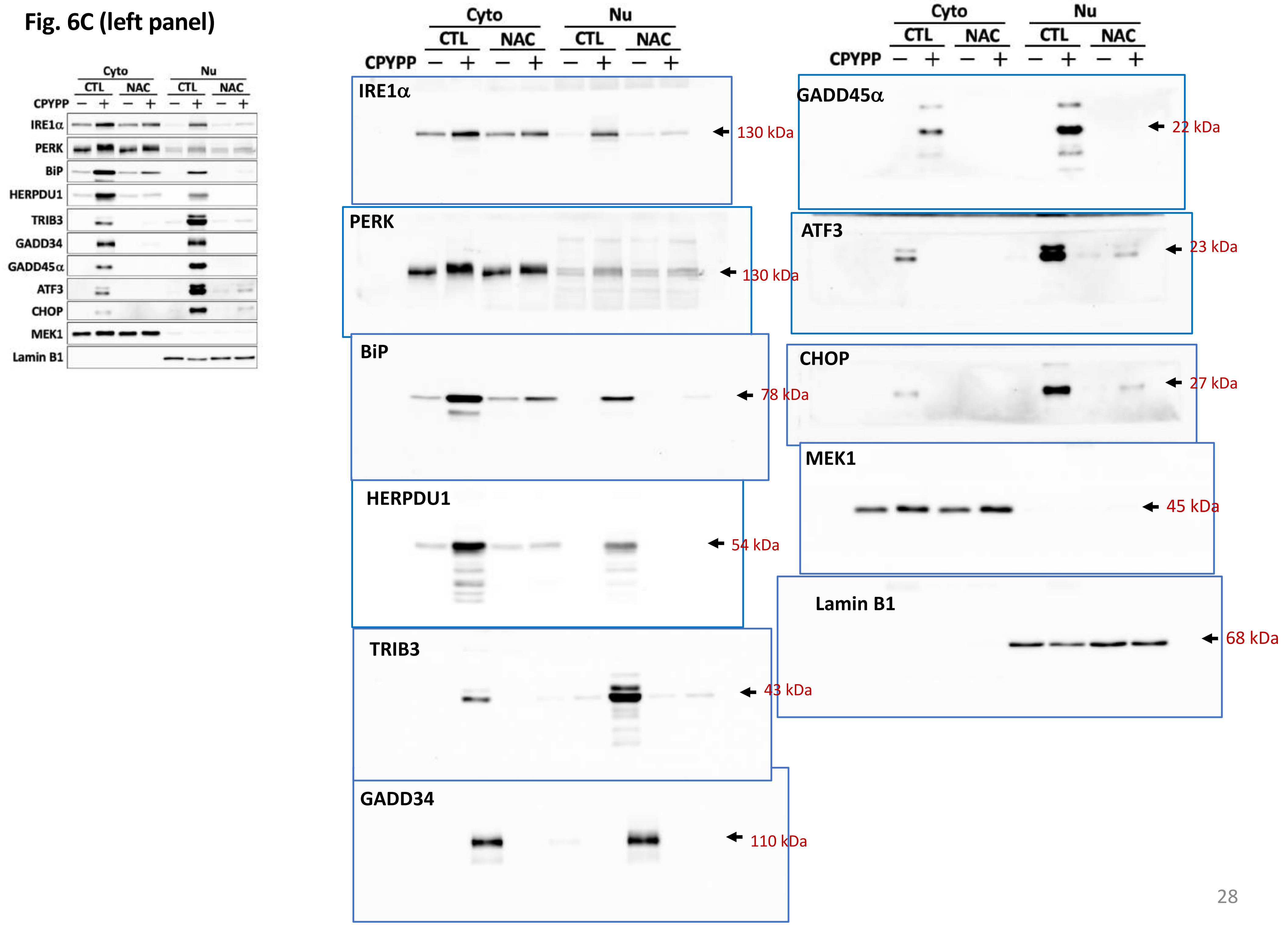

Fig. 6C (right panel)

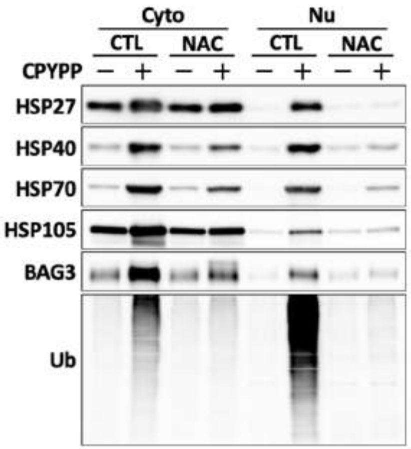

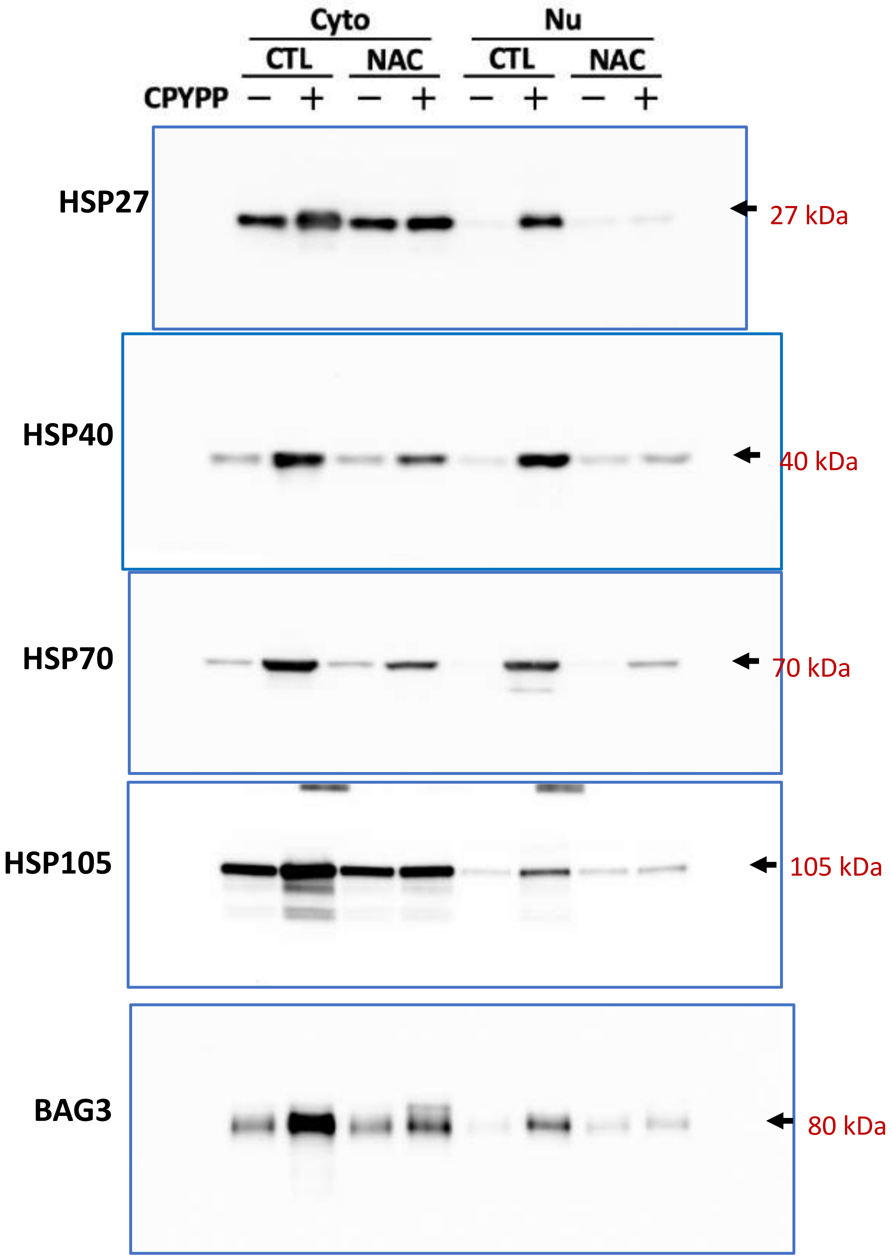

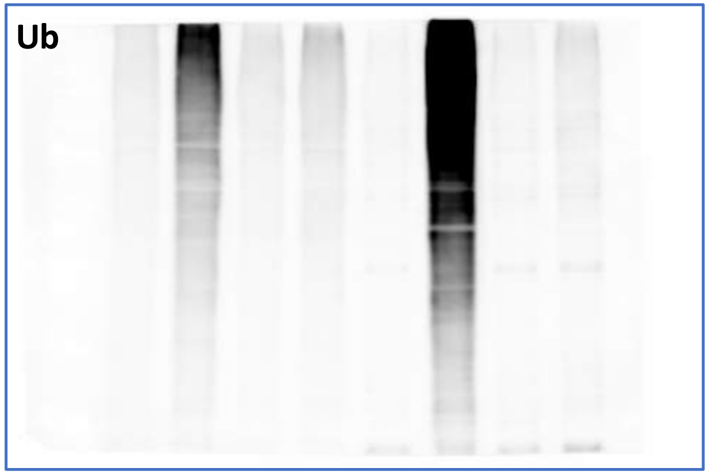

Fig. 6E

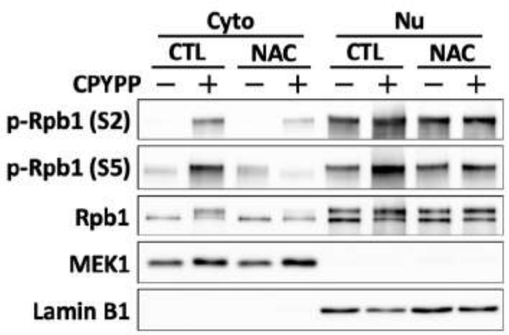
Cyto
Nu
CTL
NAC
CTL
NAC
CPYPP
p-Rpb1 (S2)
p-Rpb1 (S5)
Rpb1
MEK1
Lamin B1

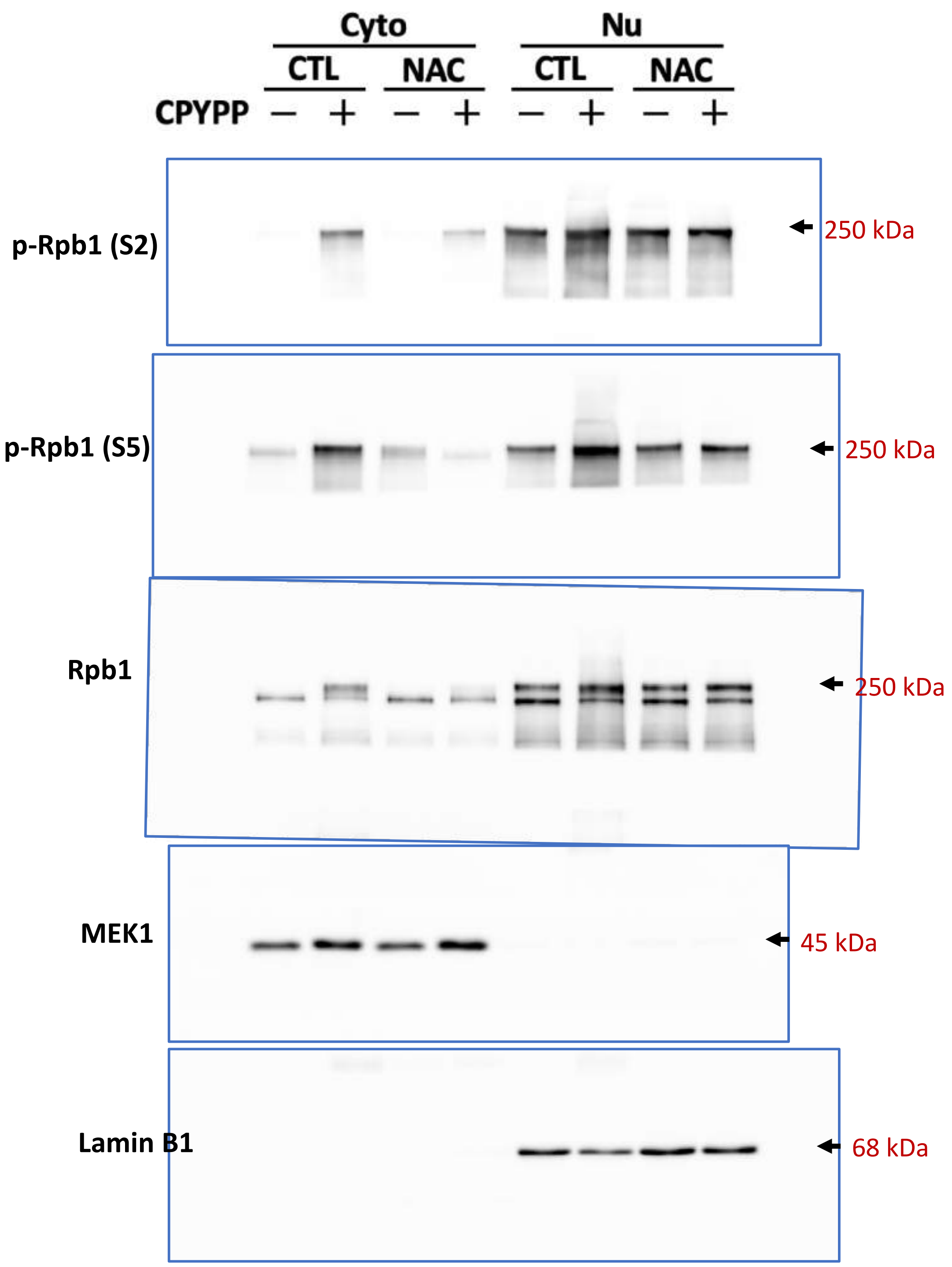
Cyto
Nu
CTL
NAC
CTL
NAC
CPYPP
p-Rpb1 (S2)
250 kDa
p-Rpb1 (S5)
250 kDa
Rpb1
250 kDa
MEK1
45 kDa
Lamin B1
68 kDa

Fig. 6H

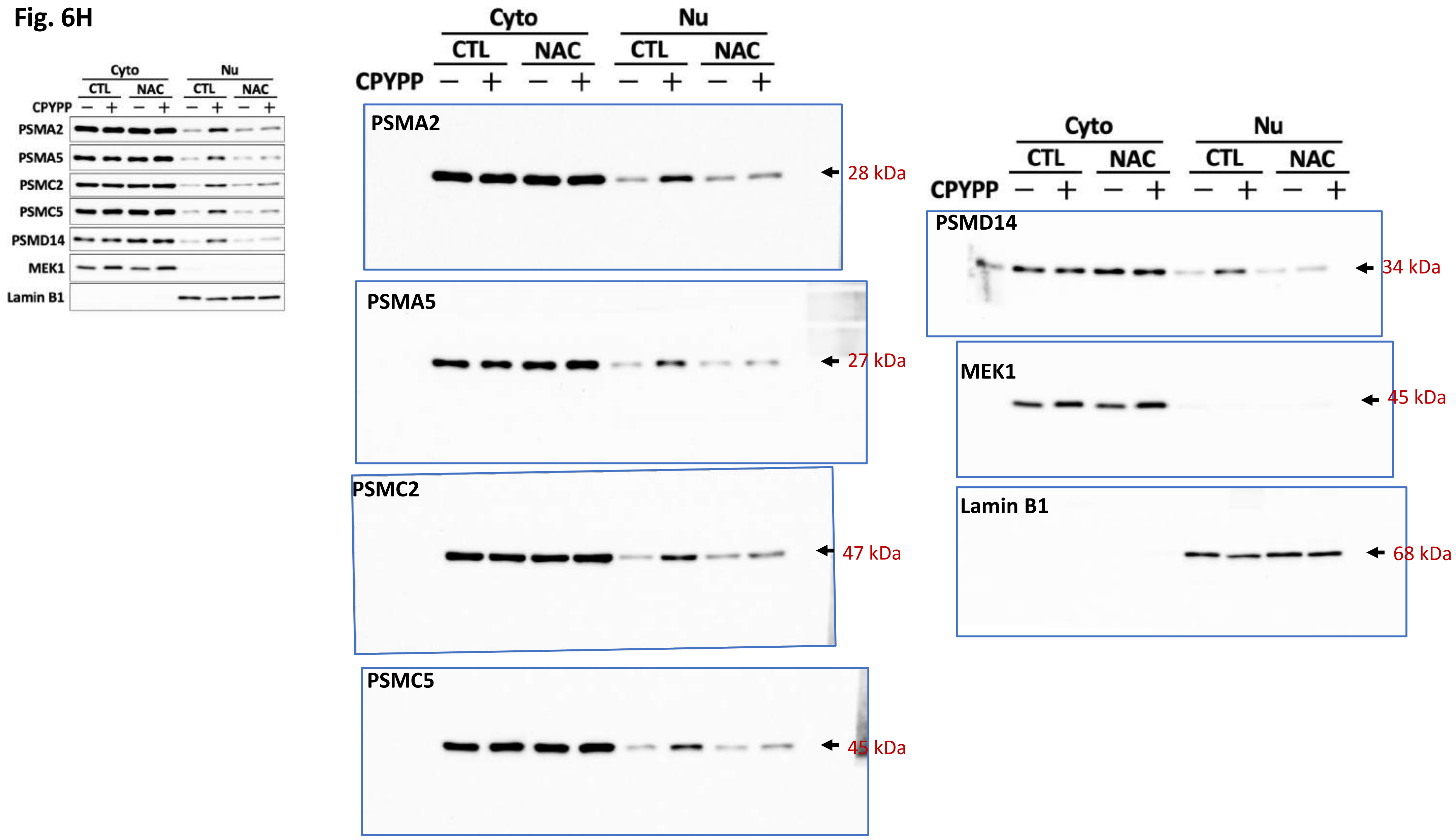
Cyto
Nu
CTL
NAC
CPYPP
PSMA2
PSMA5
PSMC2
PSMC5
PSMD14
MEK1
Lamin B1
28 kDa
27 kDa
47 kDa
45 kDa
34 kDa
45 kDa
68 kDa

Fig. 6L (left panel)

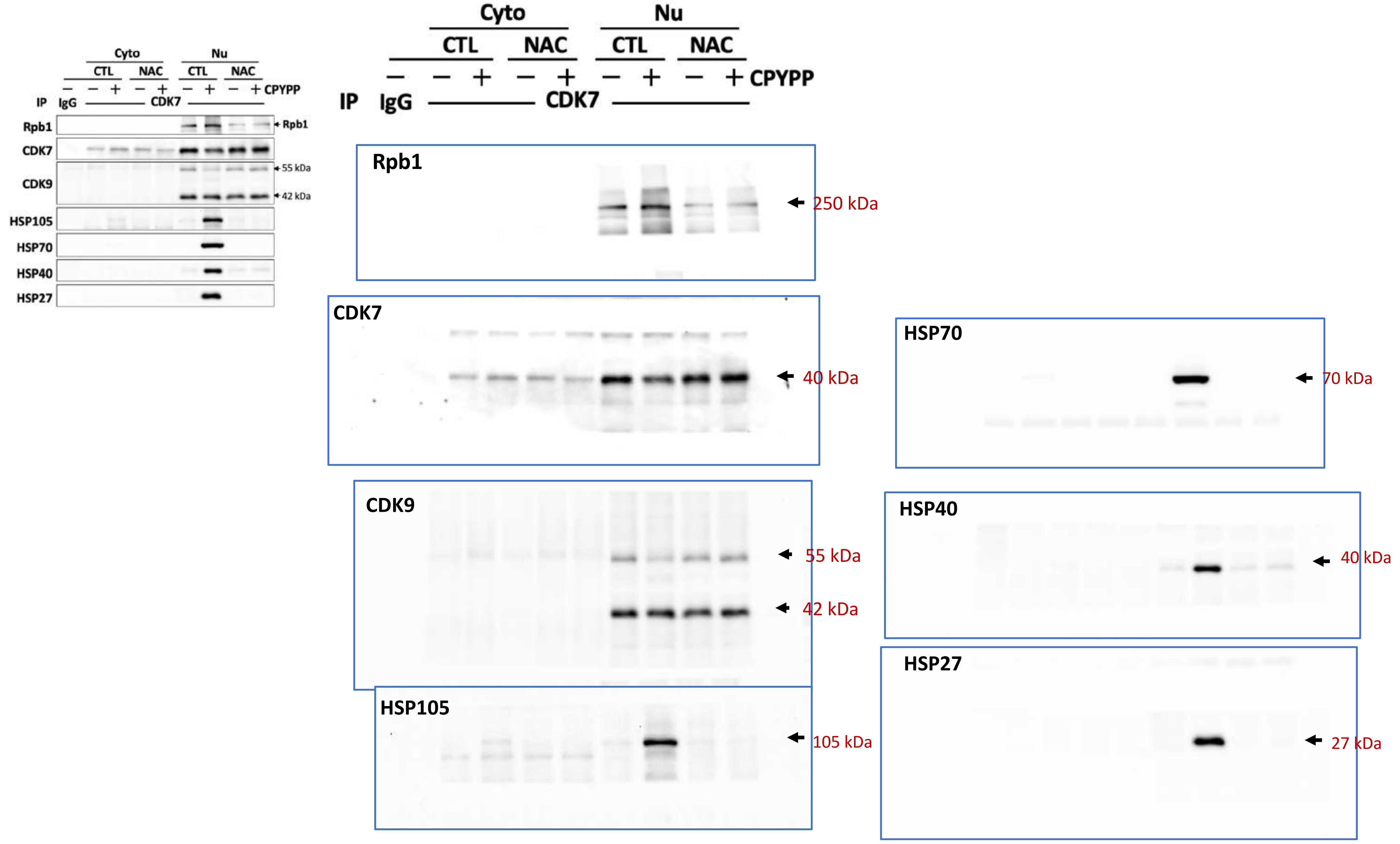

Fig. 6L (right panel)

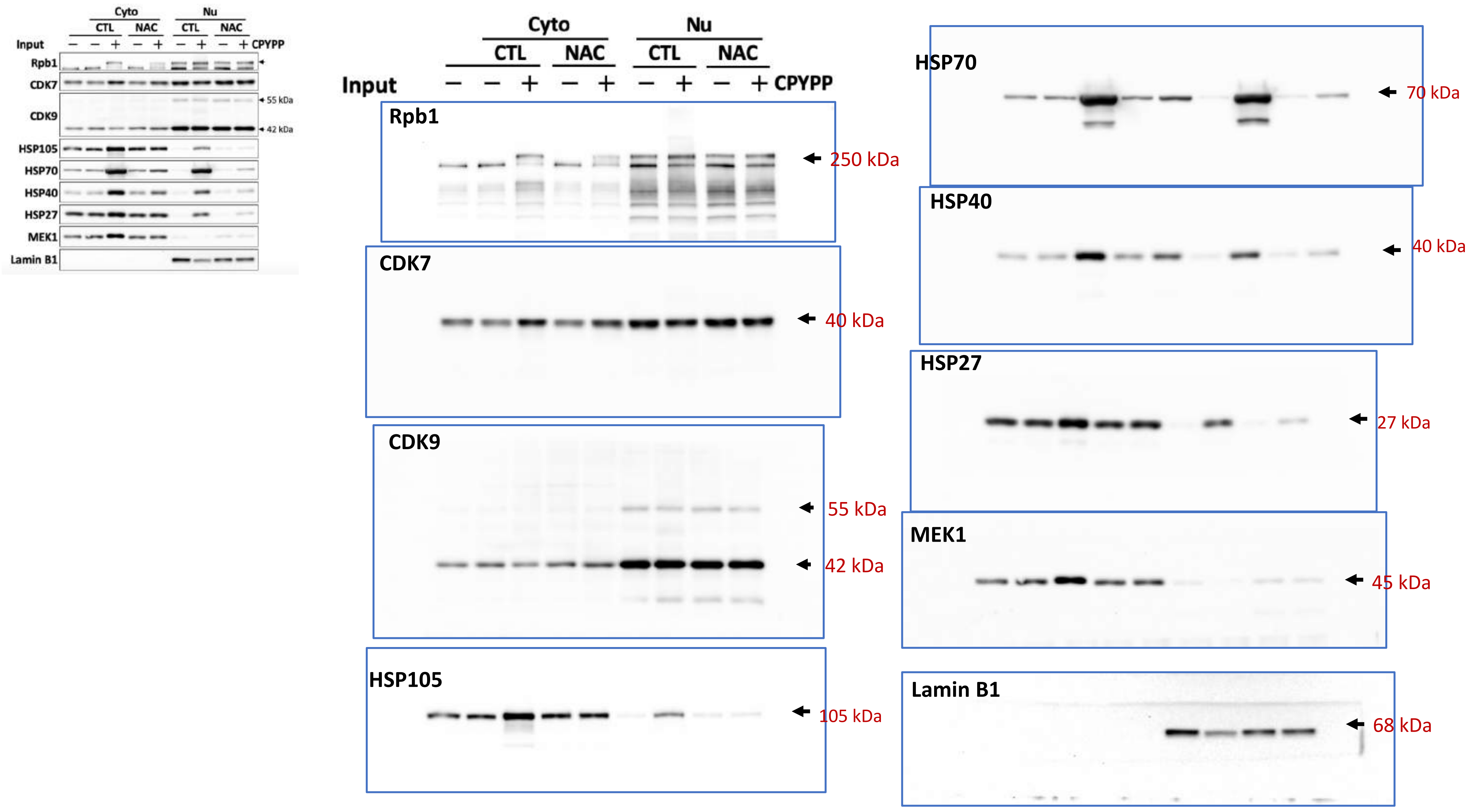

Fig. 7C (left panel)

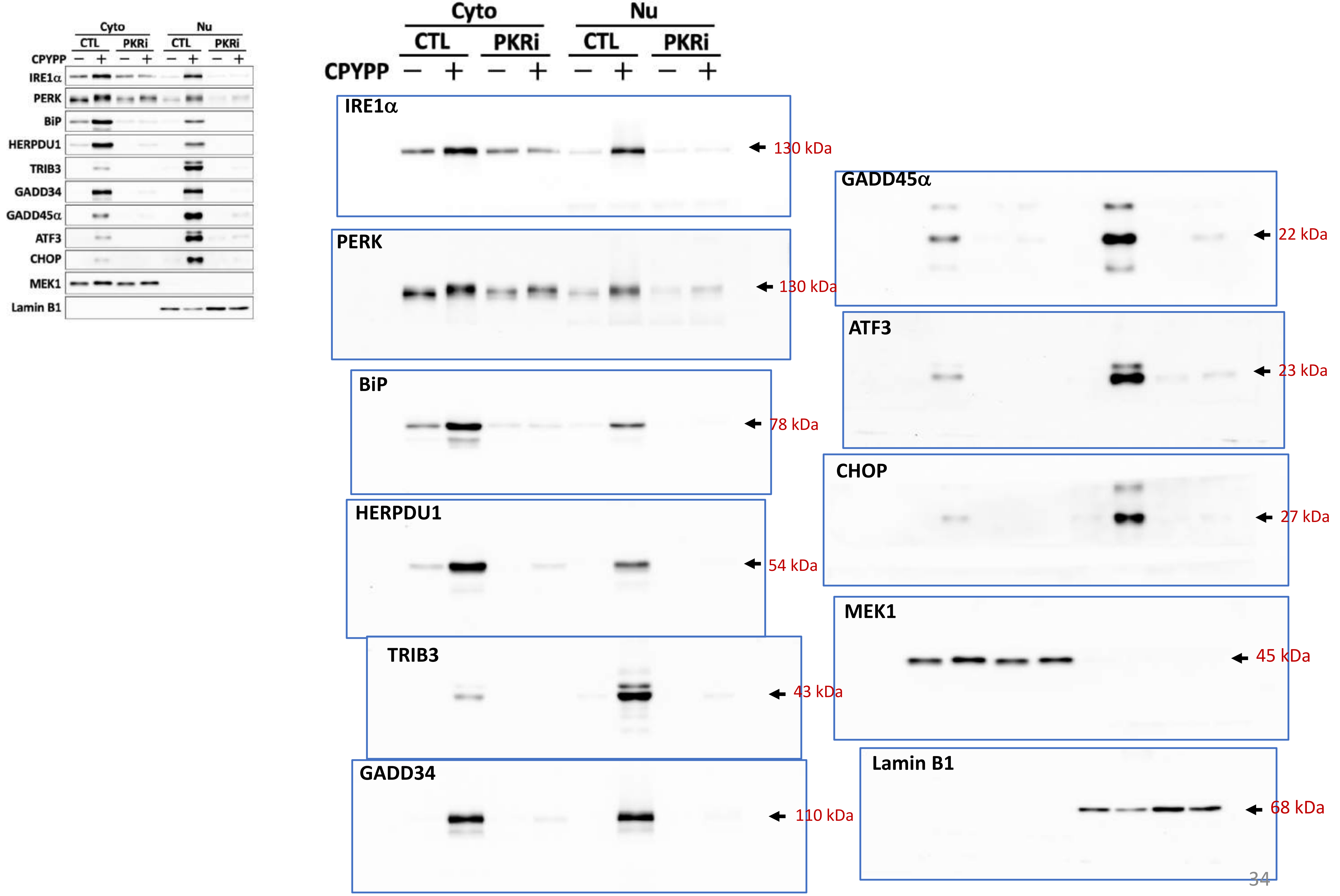

Fig. 7C (right panel)

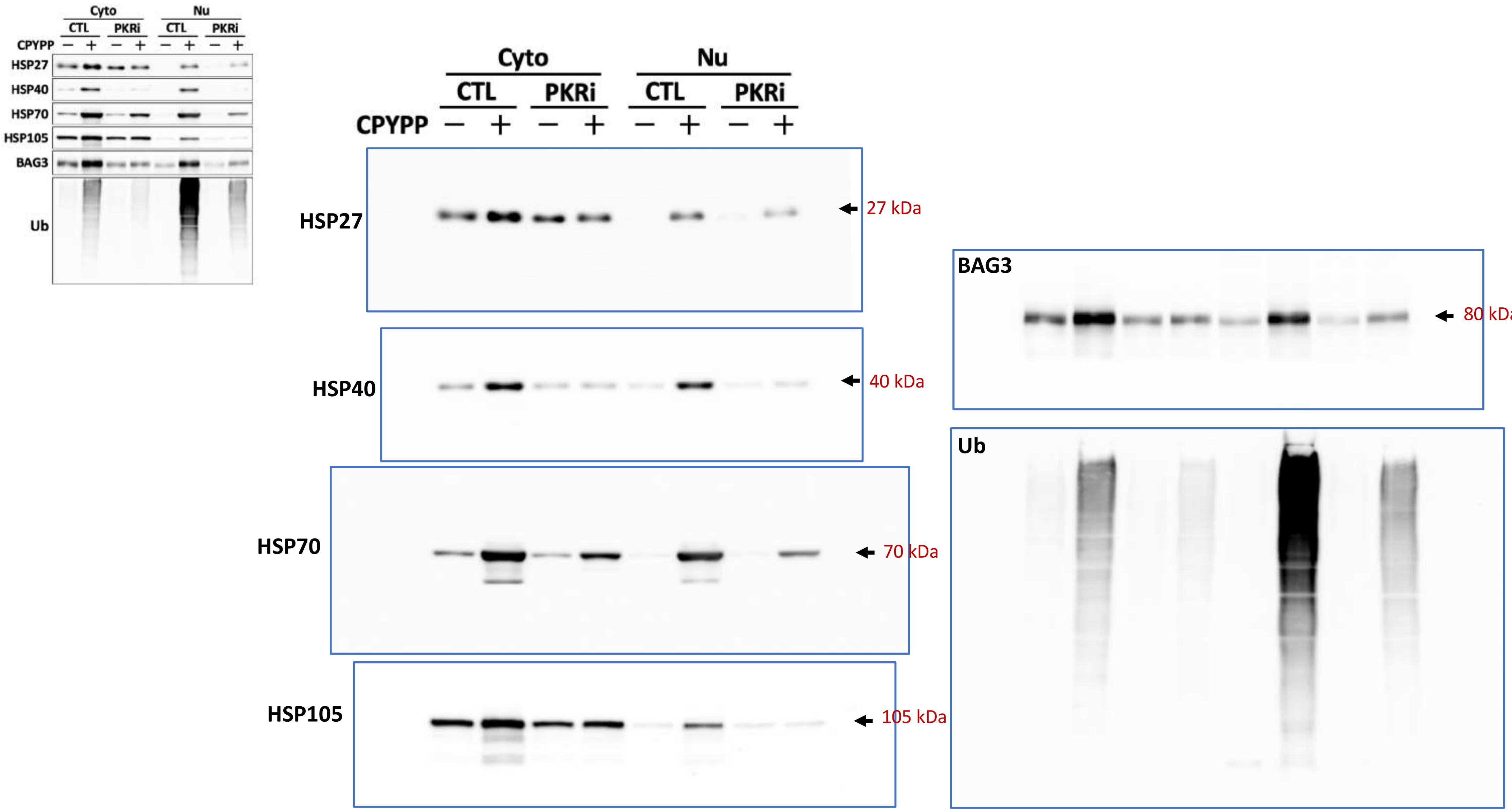

Fig. 7E

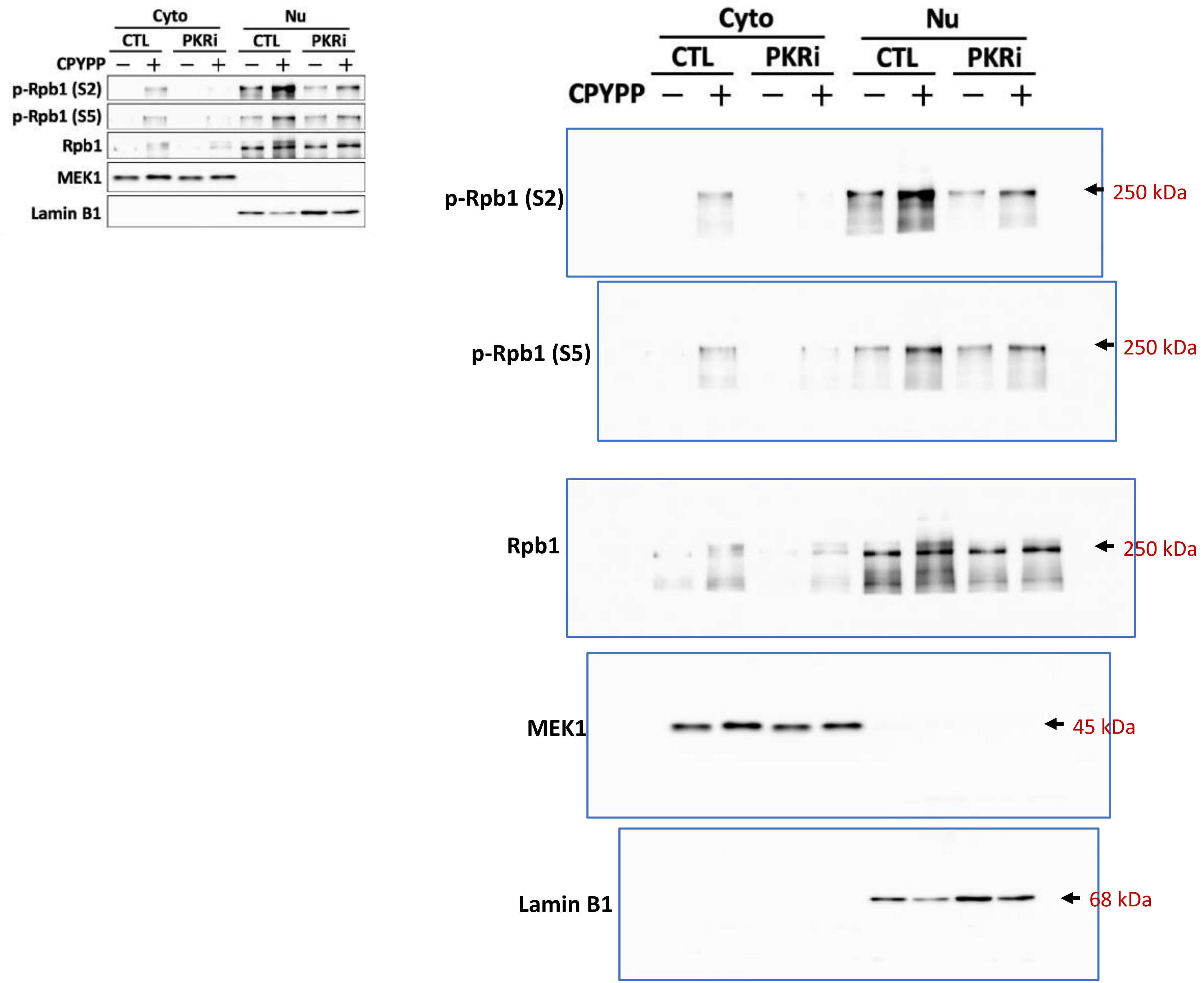
Cyto
Nu
CTL
PKRi
CTL
PKRi
CPYPP − + − + − + − +
p-Rpb1 (S2)
p-Rpb1 (S5)
Rpb1
MEK1
Lamin B1
Cyto
Nu
CTL
PKRi
CTL
PKRi
CPYPP − + − + − + − +
p-Rpb1 (S2)
250 kDa
p-Rpb1 (S5)
250 kDa
Rpb1
250 kDa
MEK1
45 kDa
Lamin B1
68 kDa

Fig. 7F

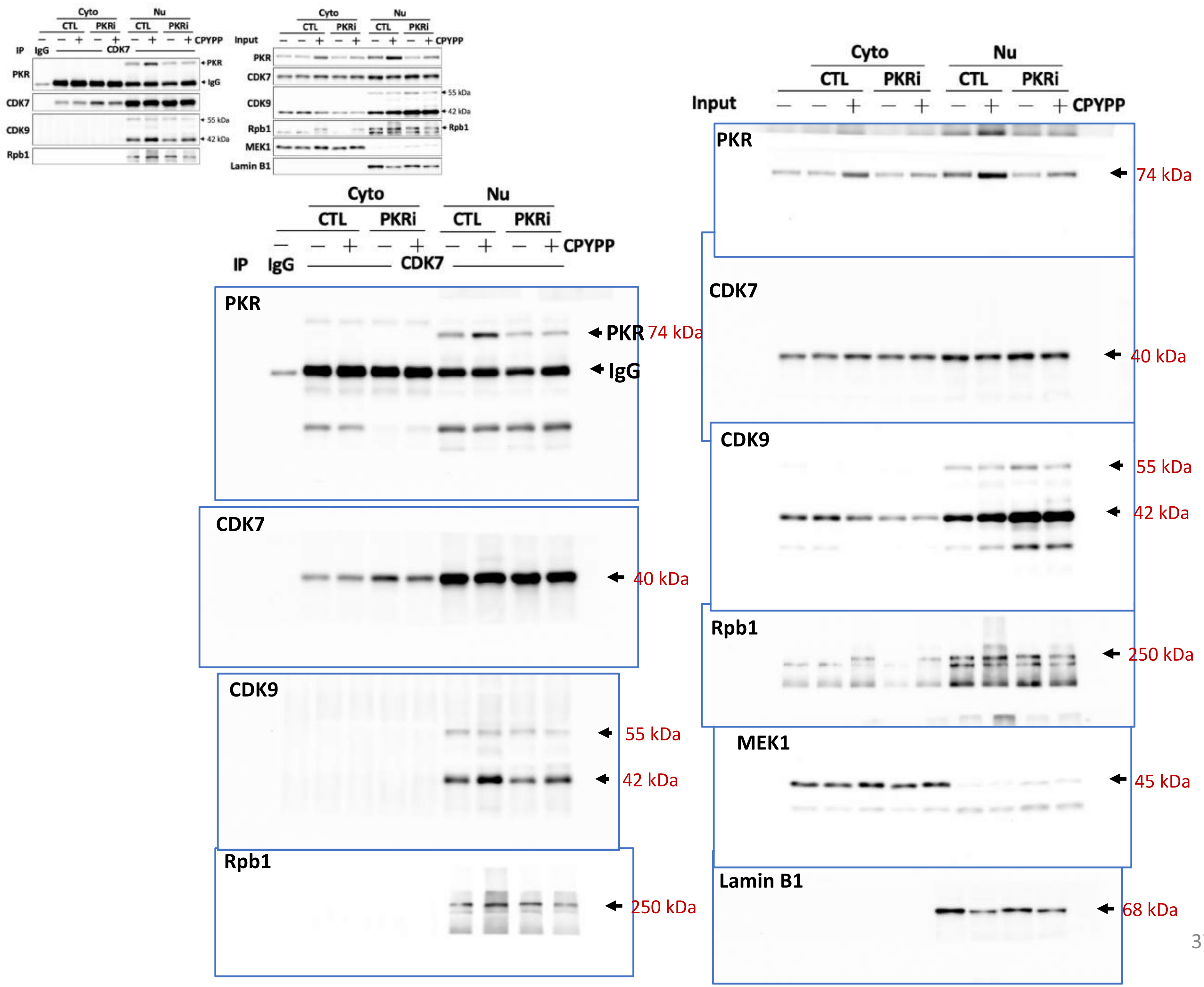
Cyto
Nu
CTL
PKRi
CTL
PKRi
IP
IgG
CDK7
CPYPP
PKR
CDK7
CDK9
Rpb1
55 kDa
42 kDa
Input
MEK1
Lamin B1
PKR 74 kDa
IgG
40 kDa
55 kDa
42 kDa
250 kDa
74 kDa
45 kDa
68 kDa

Fig. 7G

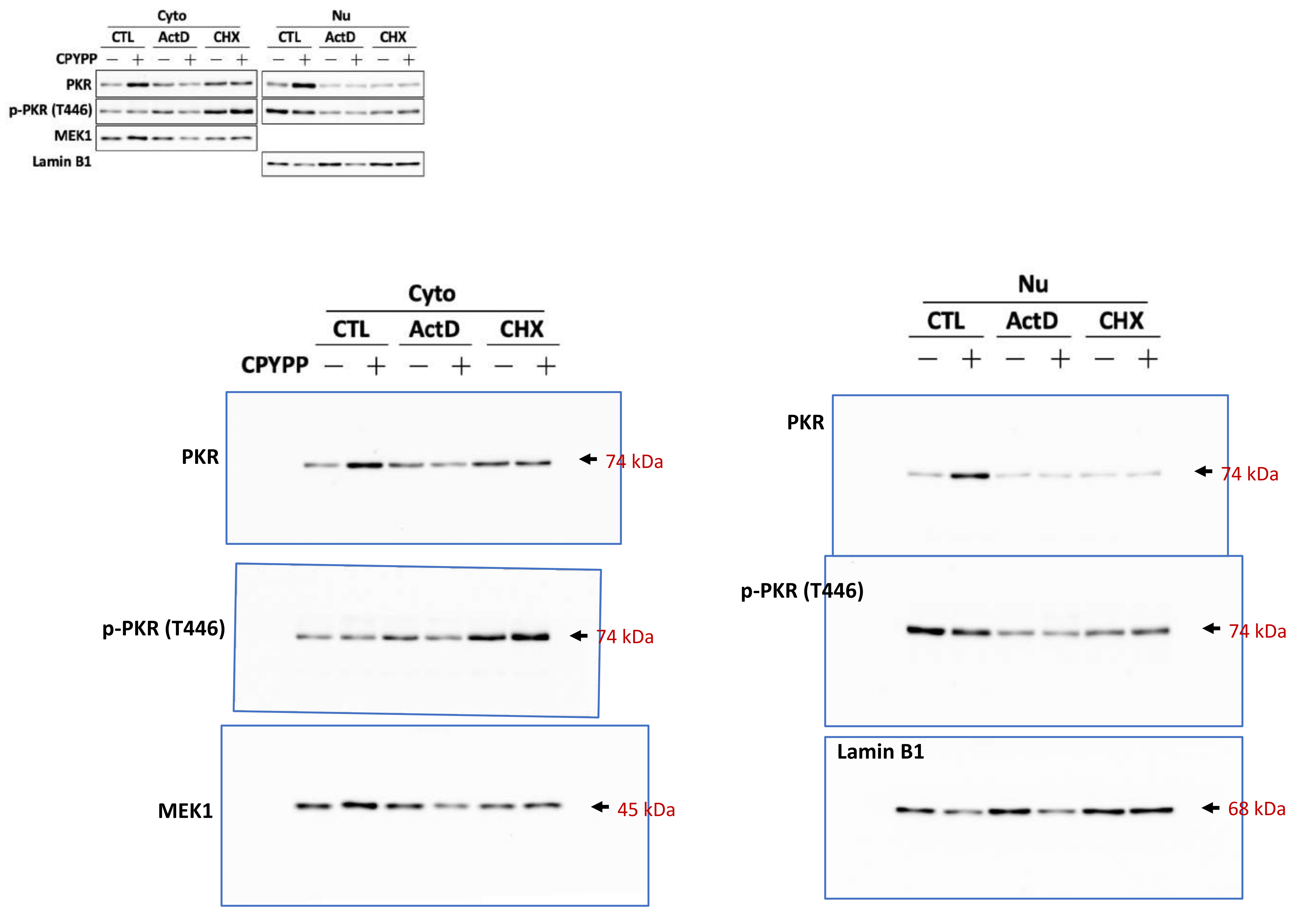
Cyto
Nu
CTL
ActD
CHX
CPYPP
PKR
p-PKR (T446)
MEK1
Lamin B1
74 kDa
45 kDa
68 kDa

Fig. 7H

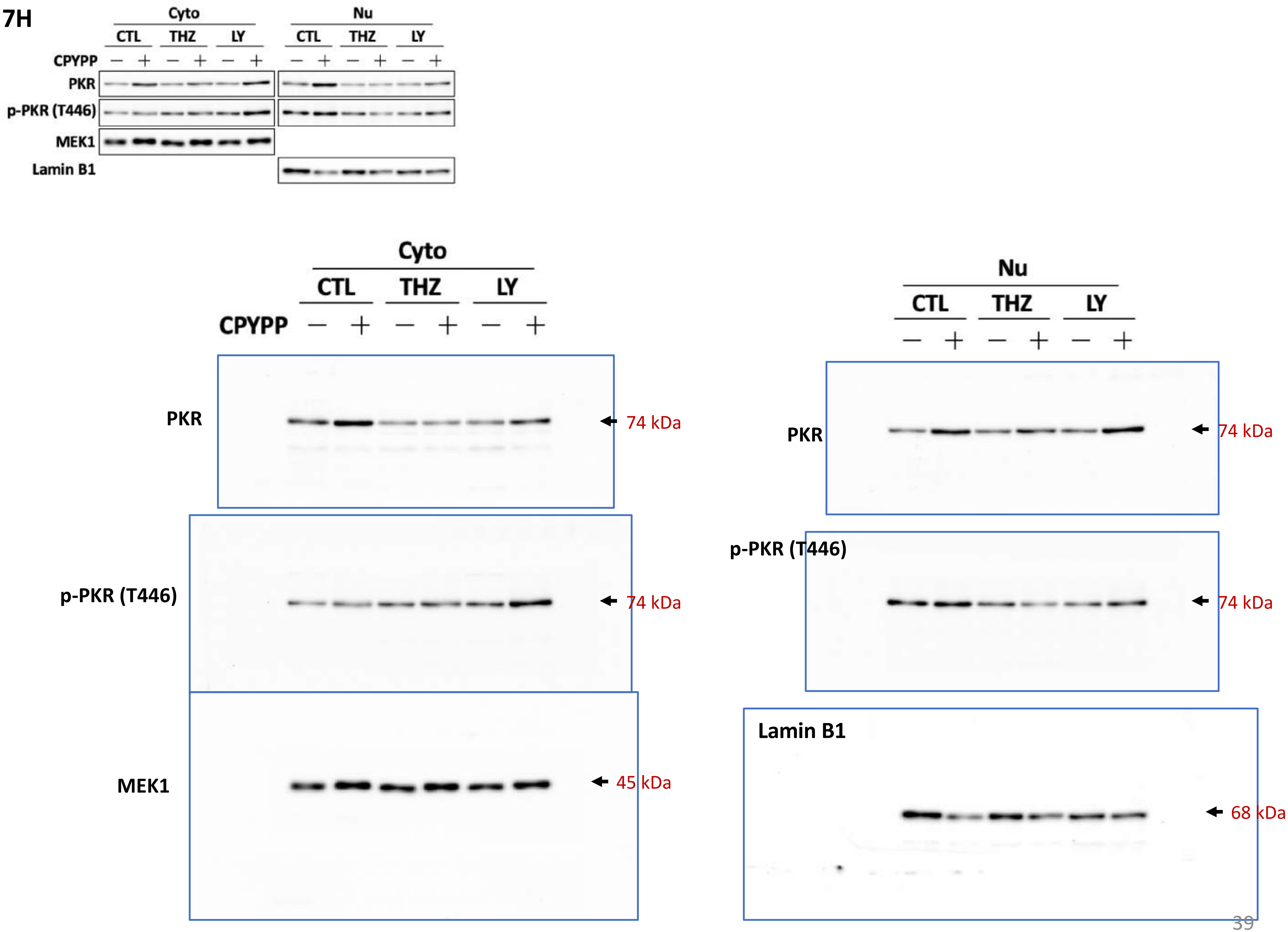

Cyto
Nu
CTL
THZ
LY
CPYPP
− + − + − +
PKR
p-PKR (T446)
MEK1
Lamin B1
74 kDa
74 kDa
45 kDa
74 kDa
74 kDa
68 kDa

Fig. 7K

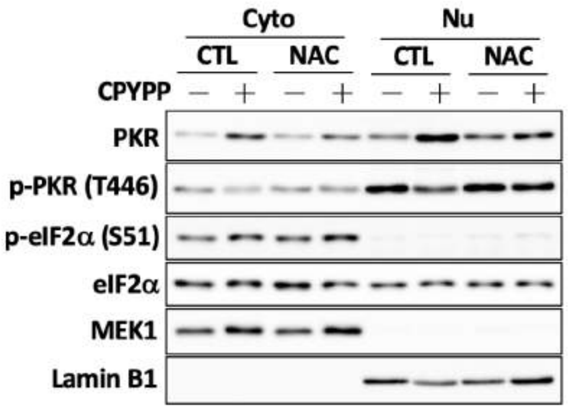
Cyto
Nu
CTL
NAC
CTL
NAC
CPYPP – + – + – + – +
PKR
p-PKR (T446)
p-eIF2α (S51)
eIF2α
MEK1
Lamin B1

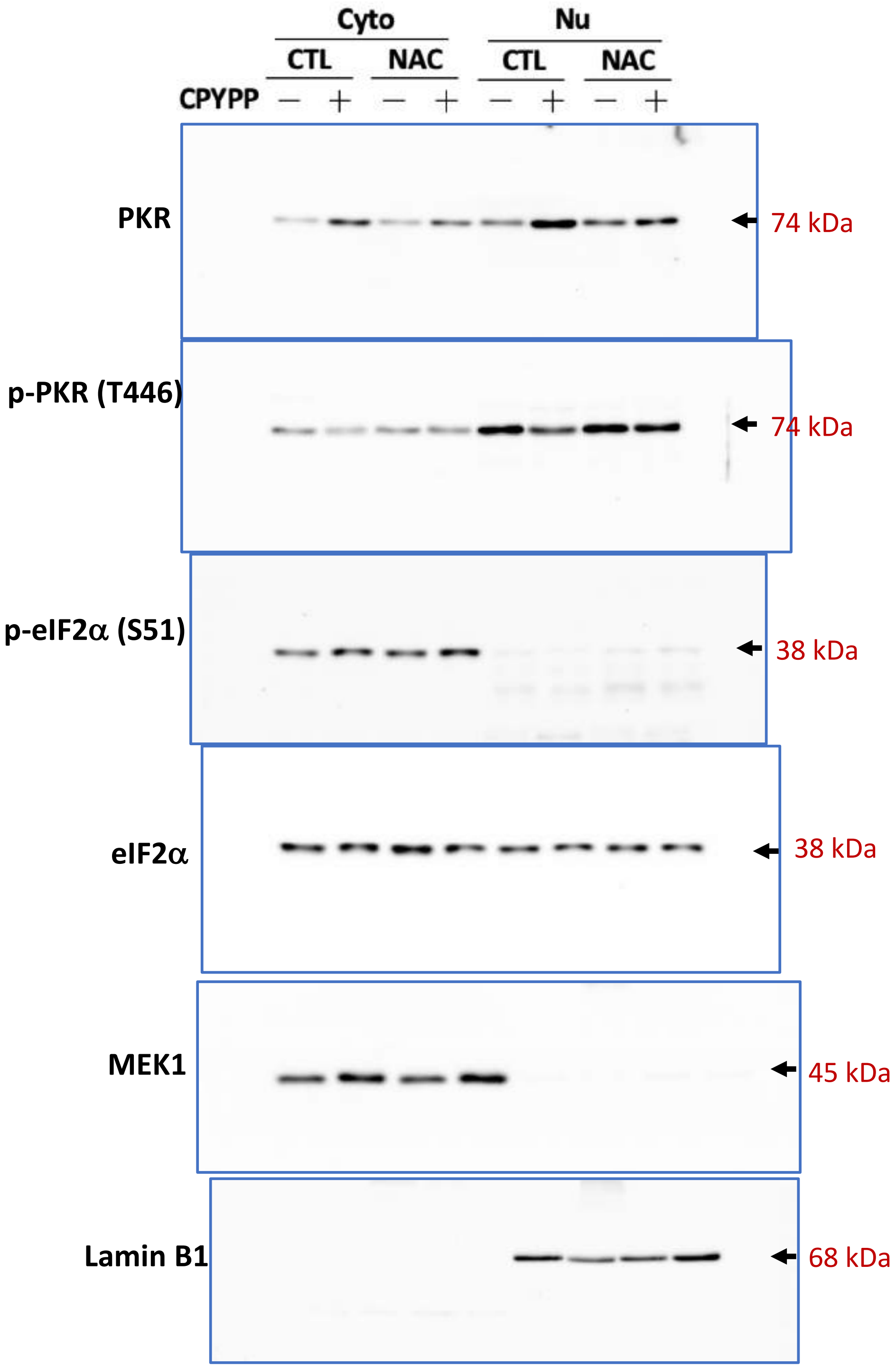
Cyto
Nu
CTL
NAC
CTL
NAC
CPYPP – + – + – + – +
PKR
74 kDa
p-PKR (T446)
74 kDa
p-eIF2α (S51)
38 kDa
eIF2α
38 kDa
MEK1
45 kDa
Lamin B1
68 kDa

Fig. 7L

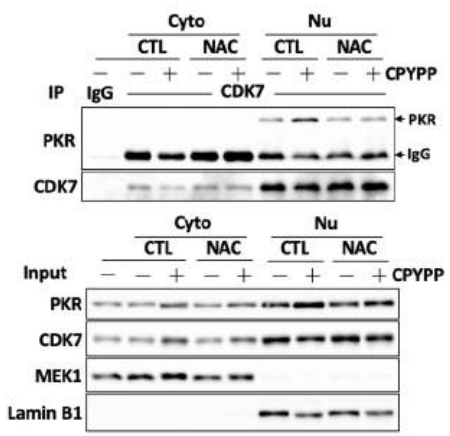
Cyto
Nu
CTL
NAC
CTL
NAC
CPYPP
IP
IgG
CDK7
PKR
CDK7
PKR
IgG
Input
PKR
CDK7
MEK1
Lamin B1

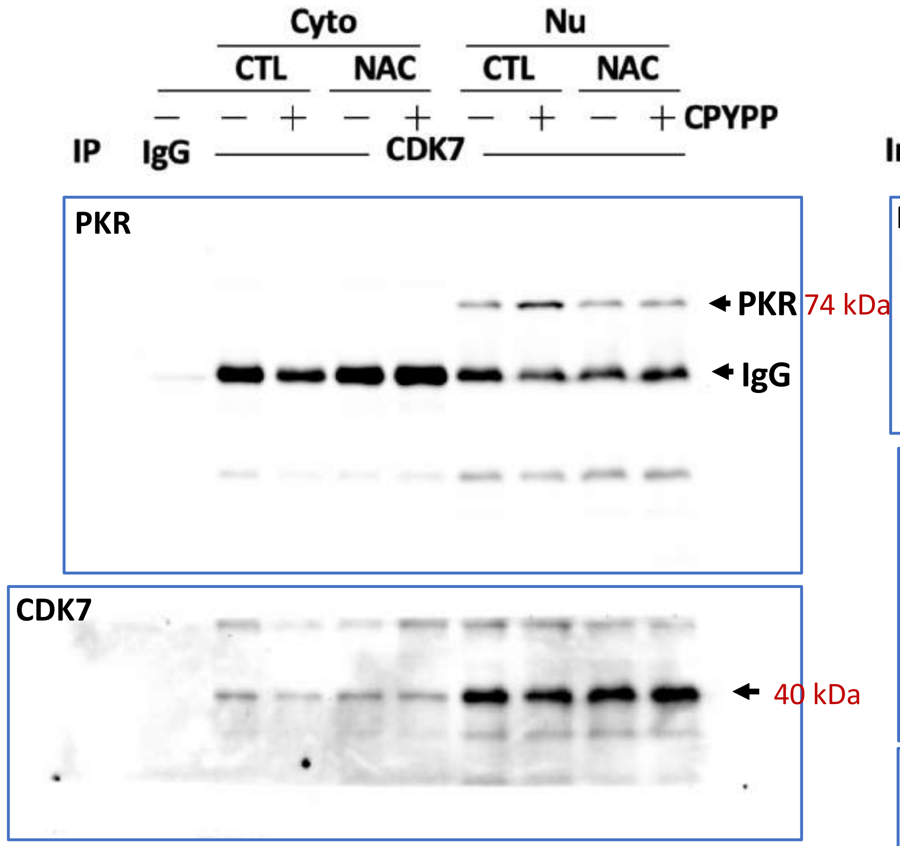
Cyto
Nu
CTL
NAC
CTL
NAC
— — + — + — + — + CPYPP
IP
IgG
CDK7
PKR
PKR
74 kDa
IgG
CDK7
40 kDa

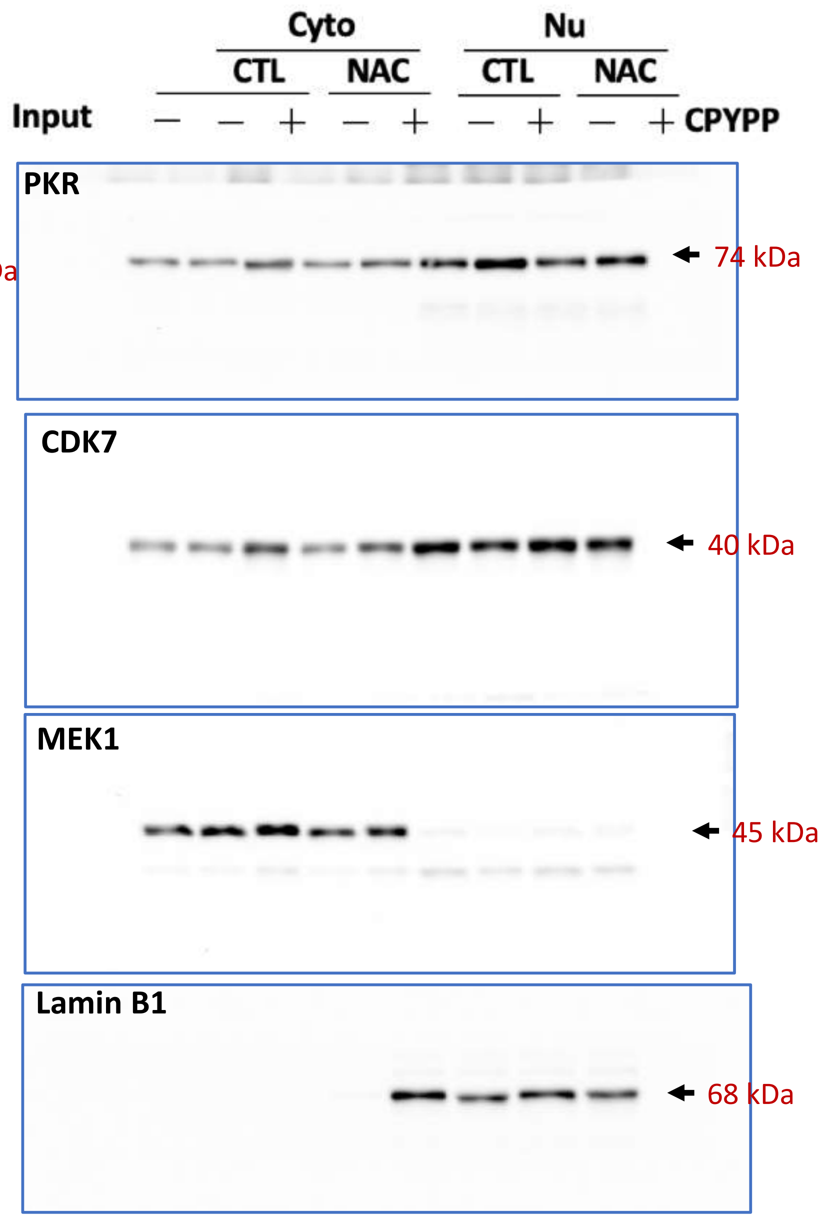
Cyto
Nu
CTL
NAC
CTL
NAC
Input
— — + — + — + — + CPYPP
PKR
74 kDa
CDK7
40 kDa
MEK1
45 kDa
Lamin B1
68 kDa

Fig. S2

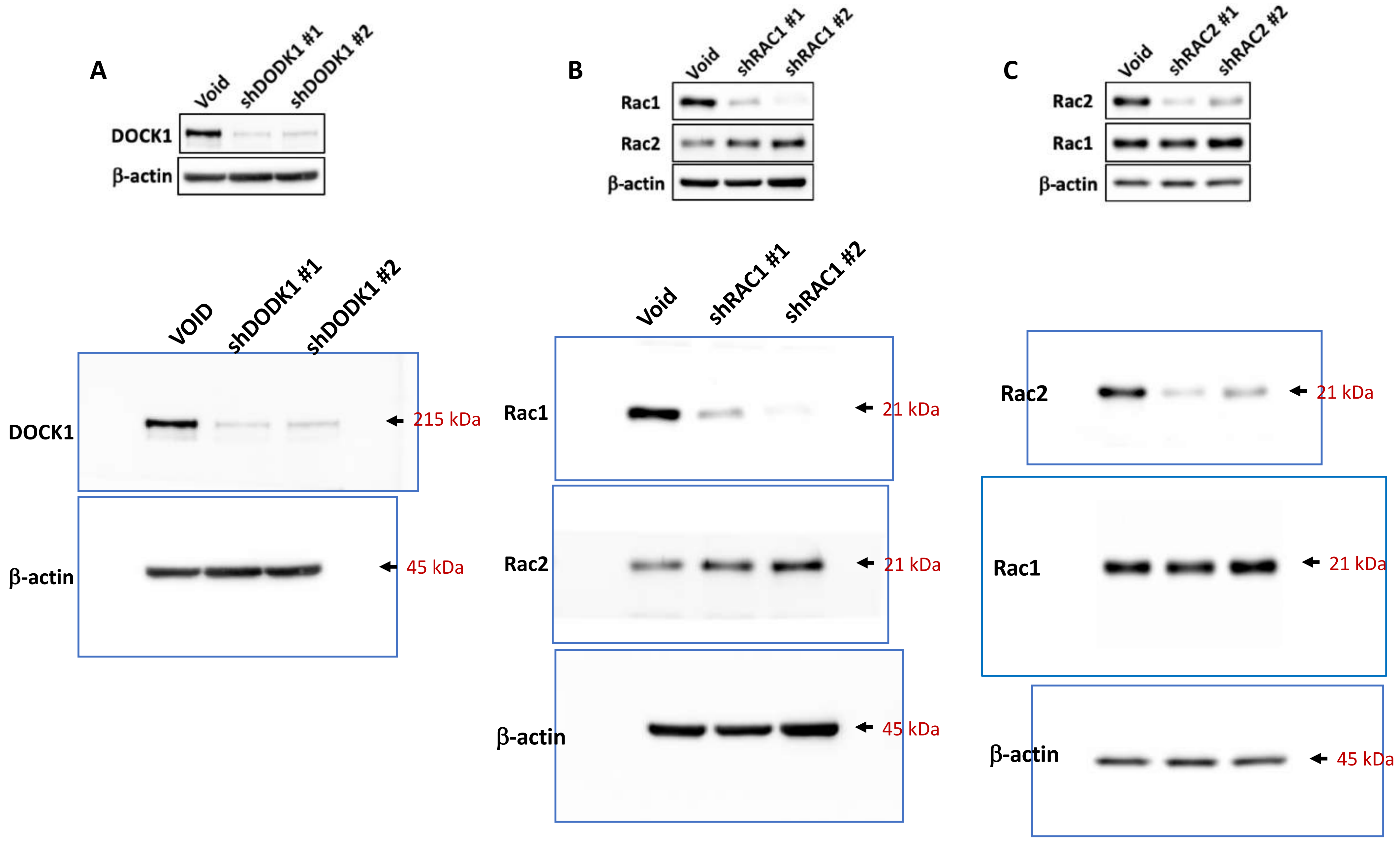

A
Void
shDODK1 #1
shDODK1 #2
DOCK1
β-actin
B
Void
shRAC1 #1
shRAC1 #2
Rac1
Rac2
β-actin
C
Void
shRAC2 #1
shRAC2 #2
Rac2
Rac1
β-actin
VOID
shDODK1 #1
shDODK1 #2
DOCK1
215 kDa
β-actin
45 kDa
Void
shRAC1 #1
shRAC1 #2
Rac1
21 kDa
Rac2
21 kDa
β-actin
45 kDa
Rac2
21 kDa
Rac1
21 kDa
β-actin
45 kDa

Fig. S4C

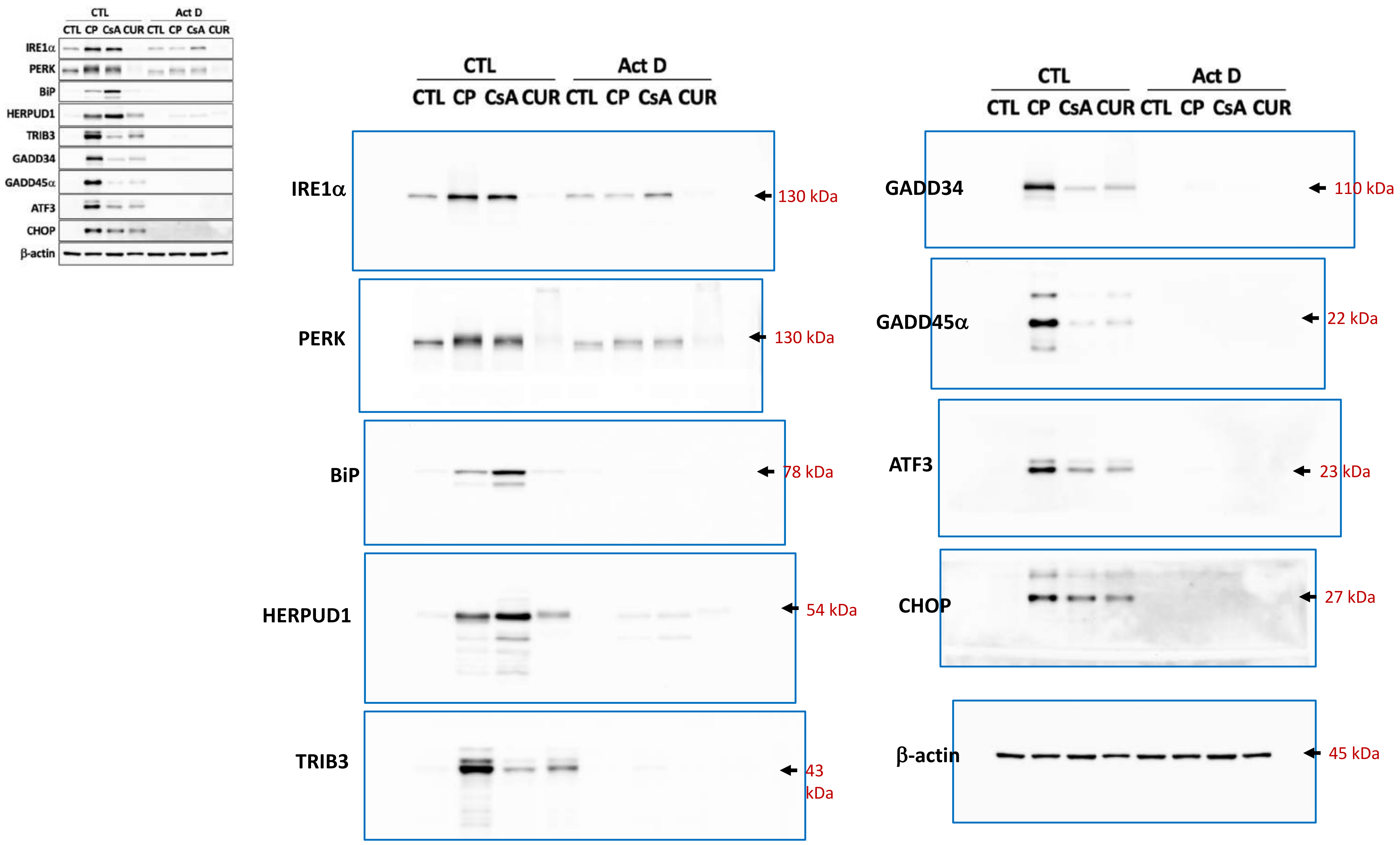

CTL
Act D
CTL CP CsA CUR CTL CP CsA CUR
IRE1α
PERK
BiP
HERPUD1
TRIB3
GADD34
GADD45α
ATF3
CHOP
β-actin
130 kDa
130 kDa
78 kDa
54 kDa
43 kDa
110 kDa
22 kDa
23 kDa
27 kDa
45 kDa

Fig. S4C

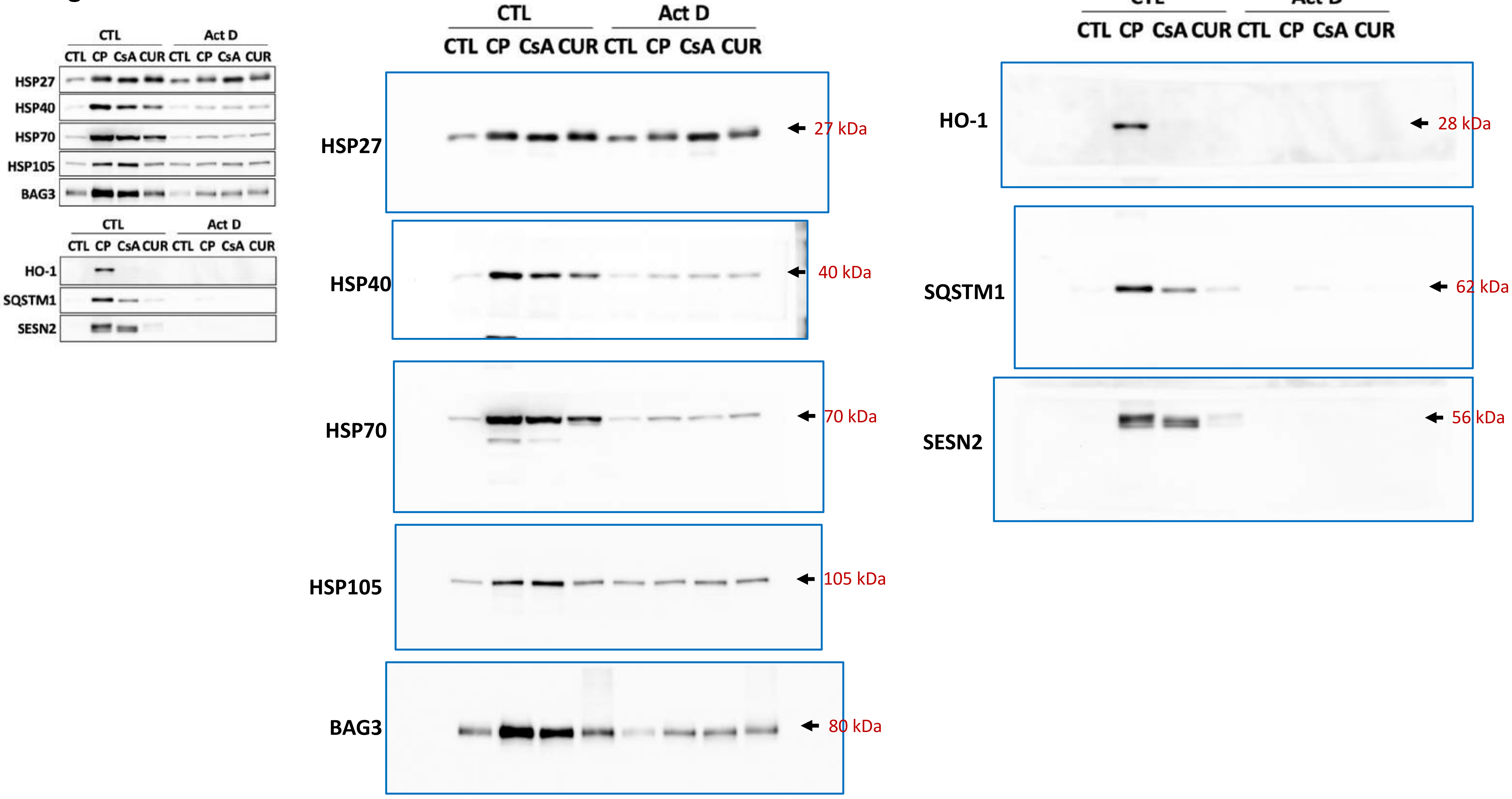
CTL
Act D
CTL CP CsA CUR CTL CP CsA CUR
HSP27
HSP40
HSP70
HSP105
BAG3
HO-1
SQSTM1
SESN2
27 kDa
40 kDa
70 kDa
105 kDa
80 kDa
28 kDa
62 kDa
56 kDa

Fig. S4D

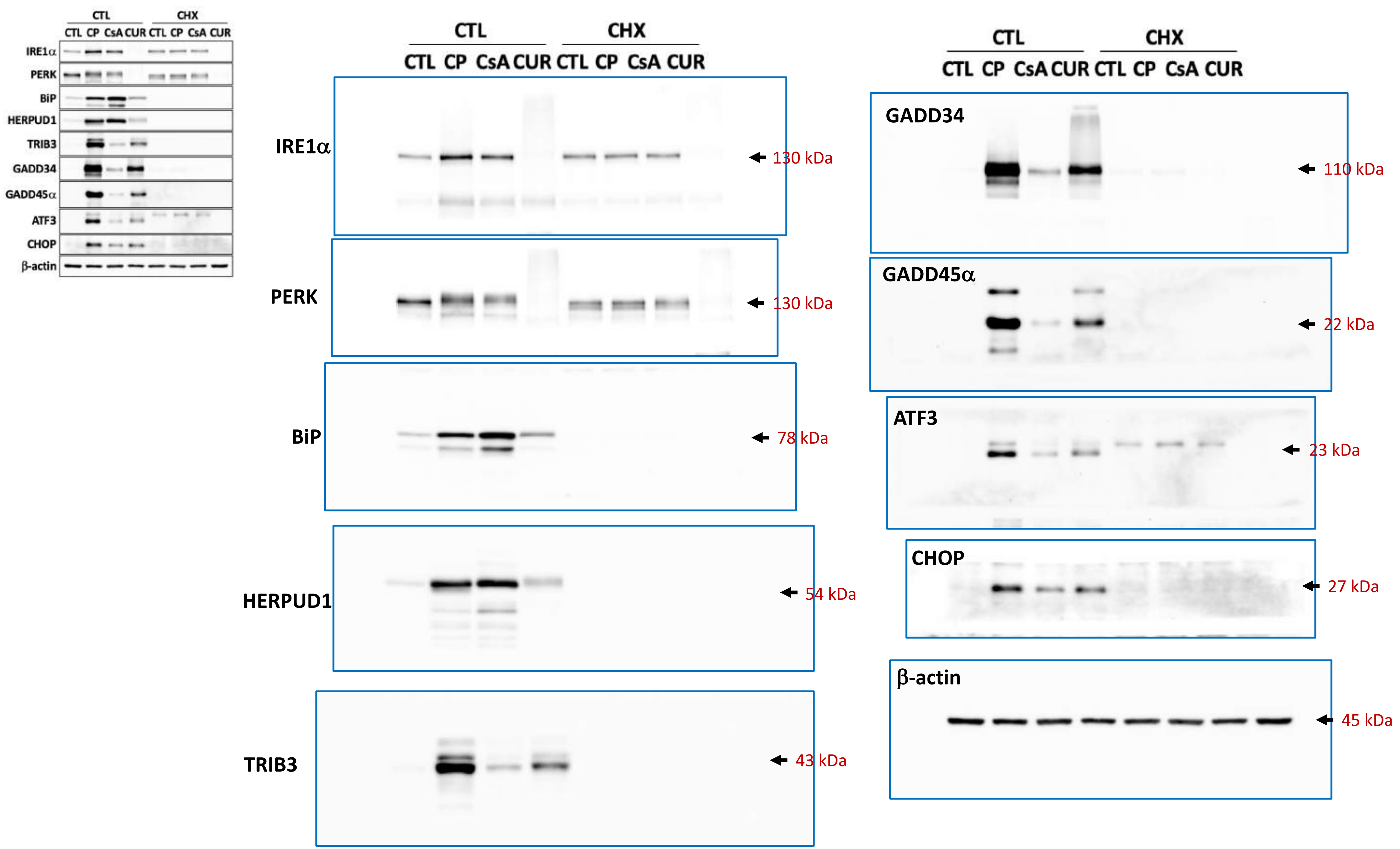
CTL
CHX
CTL CP CsA CUR CTL CP CsA CUR
IRE1α
PERK
BiP
HERPUD1
TRIB3
GADD34
GADD45α
ATF3
CHOP
β-actin
130 kDa
130 kDa
78 kDa
54 kDa
43 kDa
110 kDa
22 kDa
23 kDa
27 kDa
45 kDa

Fig. S4D

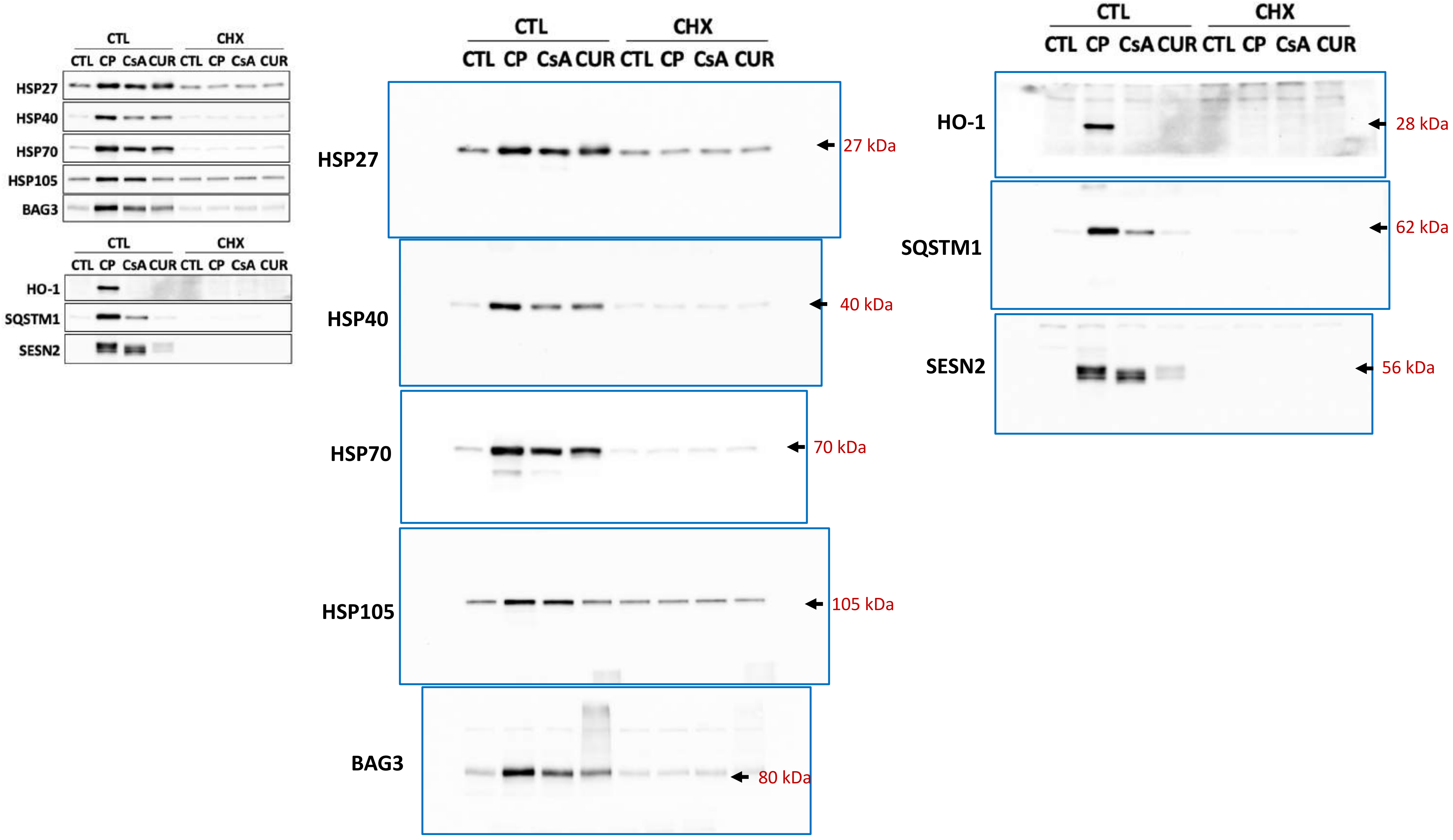
CTL
CHX
CTL CP CsA CUR CTL CP CsA CUR
HSP27
HSP40
HSP70
HSP105
BAG3
HO-1
SQSTM1
SESN2
27 kDa
40 kDa
70 kDa
105 kDa
80 kDa
28 kDa
62 kDa
56 kDa

Fig. S4E

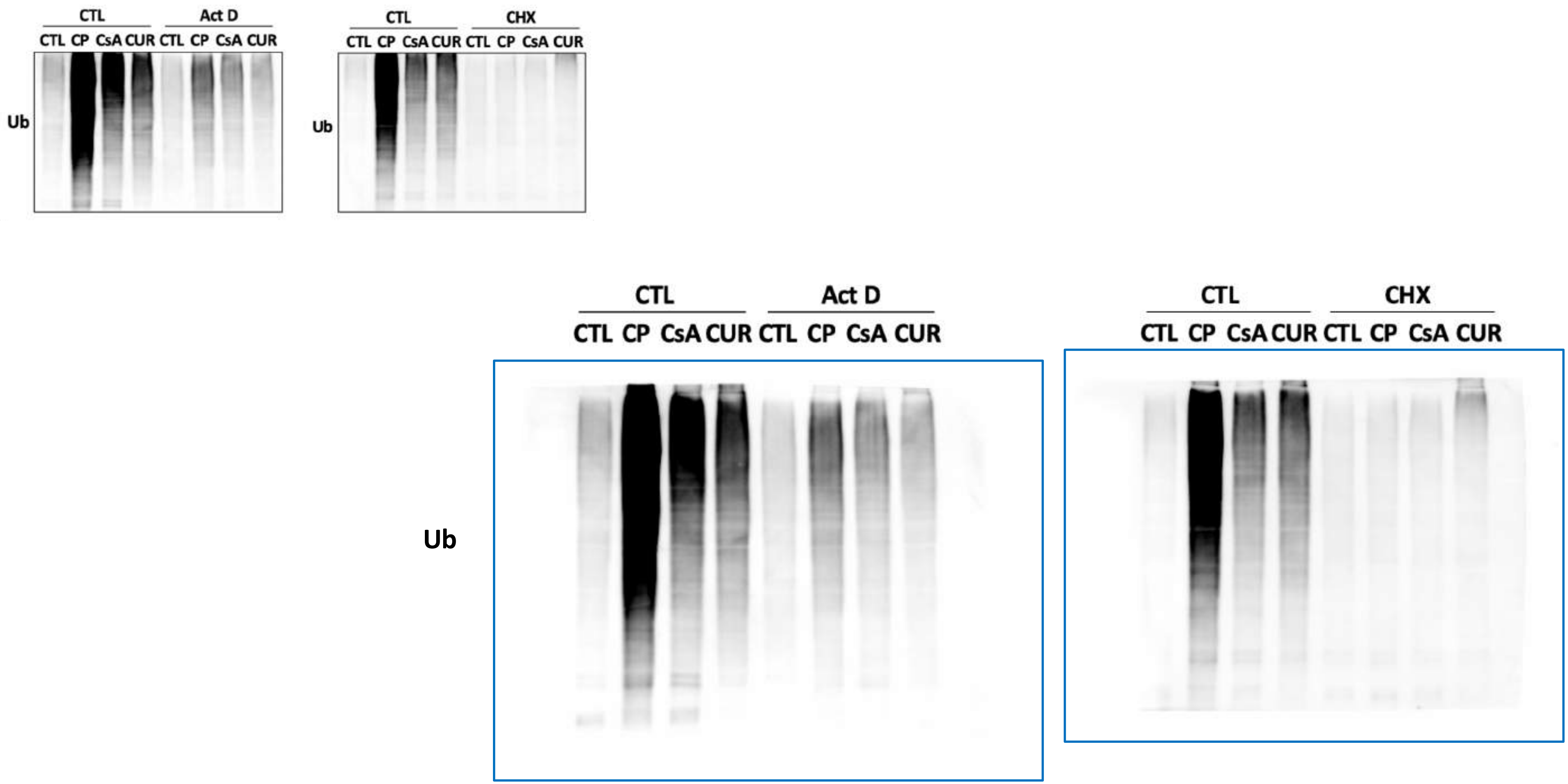
CTL
Act D
CTL CP CsA CUR CTL CP CsA CUR
Ub
CTL
CHX
CTL CP CsA CUR CTL CP CsA CUR
Ub
CTL
Act D
CTL CP CsA CUR CTL CP CsA CUR
Ub
CTL
CHX
CTL CP CsA CUR CTL CP CsA CUR

Fig. S6A

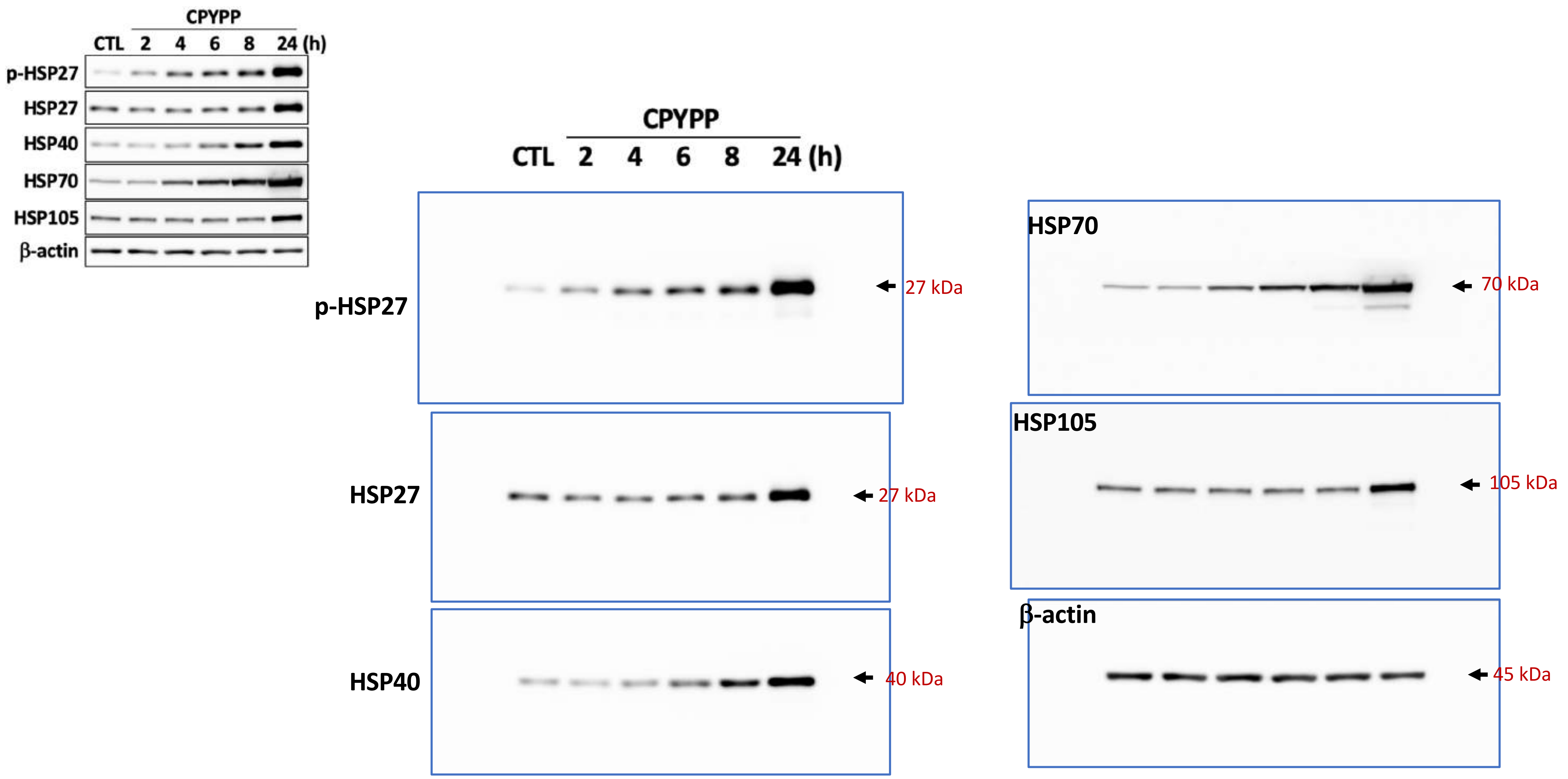
CPYPP
CTL 2 4 6 8 24 (h)
p-HSP27
HSP27
HSP40
HSP70
HSP105
β-actin
CPYPP
CTL 2 4 6 8 24 (h)
p-HSP27
27 kDa
HSP27
27 kDa
HSP40
40 kDa
HSP70
70 kDa
HSP105
105 kDa
β-actin
45 kDa

Fig. S6B

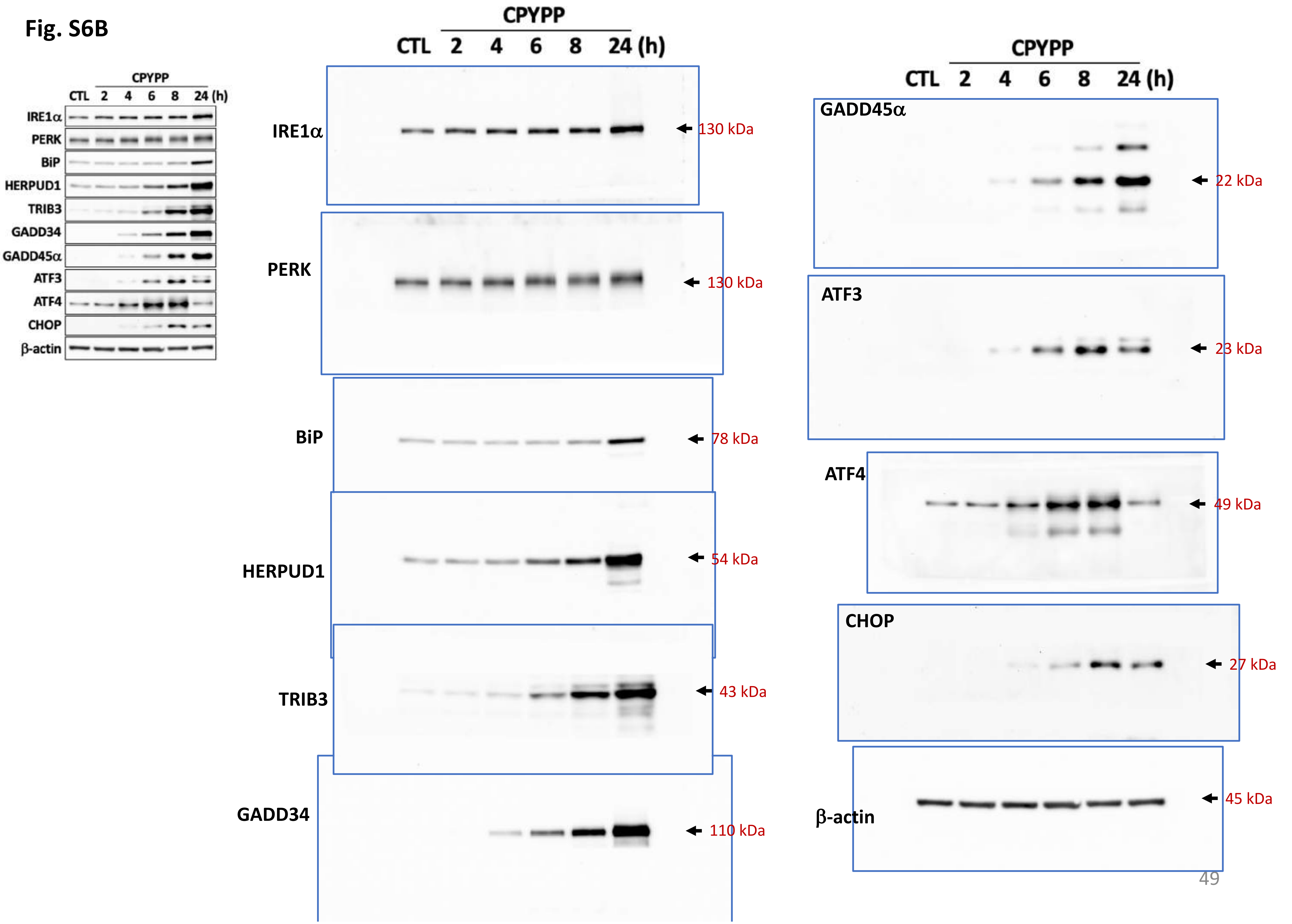

CPYPP
CTL 2 4 6 8 24 (h)
IRE1α
PERK
BiP
HERPUD1
TRIB3
GADD34
GADD45α
ATF3
ATF4
CHOP
β-actin
130 kDa
130 kDa
78 kDa
54 kDa
43 kDa
110 kDa
22 kDa
23 kDa
49 kDa
27 kDa
45 kDa

Fig. S7A

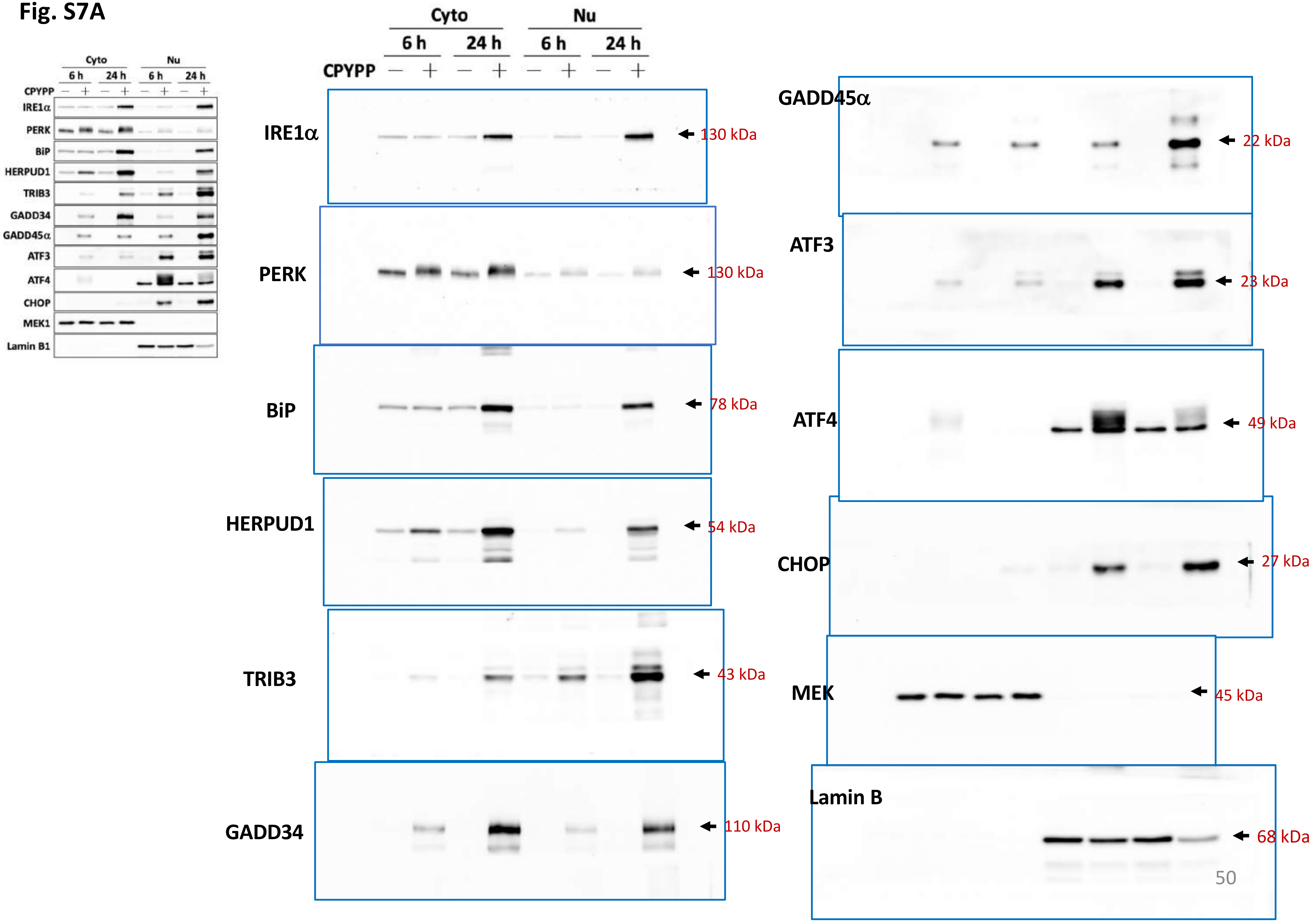

Cyto
Nu
6 h
24 h
CPYPP
IRE1α
PERK
BiP
HERPUD1
TRIB3
GADD34
GADD45α
ATF3
ATF4
CHOP
MEK1
Lamin B1
130 kDa
78 kDa
54 kDa
43 kDa
110 kDa
22 kDa
23 kDa
49 kDa
27 kDa
MEK
45 kDa
Lamin B
68 kDa

Fig. S7G

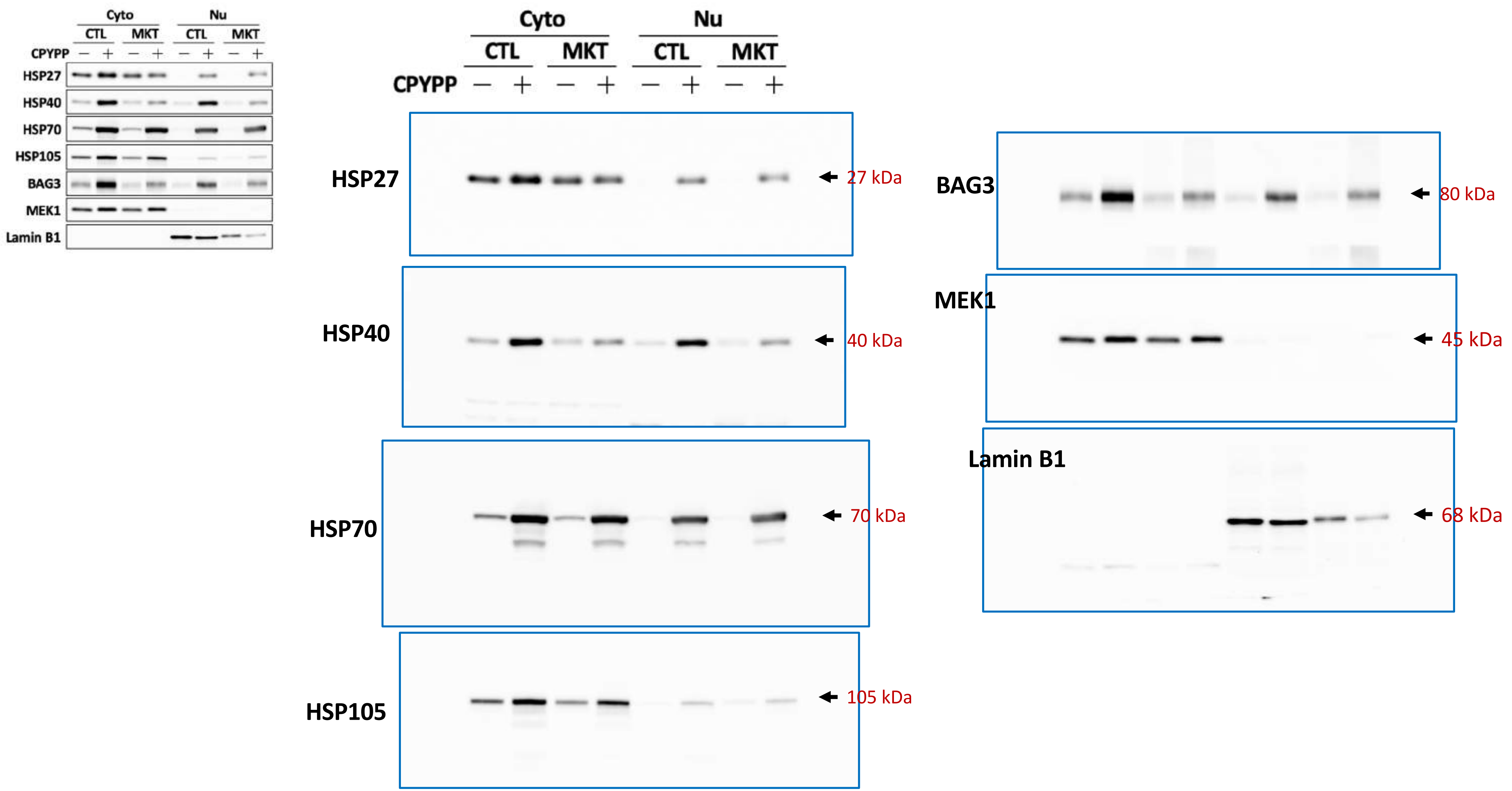

Cyto
Nu
CTL
MKT
CTL
MKT
CPYPP – + – + – + – +
HSP27
HSP40
HSP70
HSP105
BAG3
MEK1
Lamin B1
27 kDa
40 kDa
70 kDa
105 kDa
80 kDa
45 kDa
68 kDa

Fig. S8 (left panel)

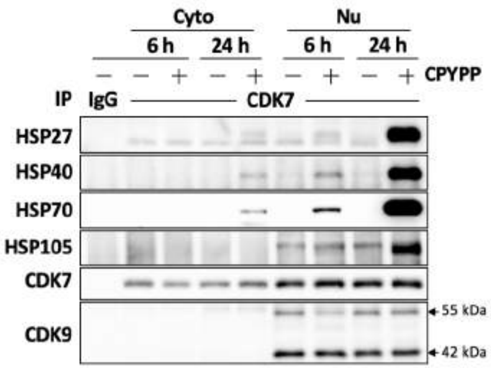

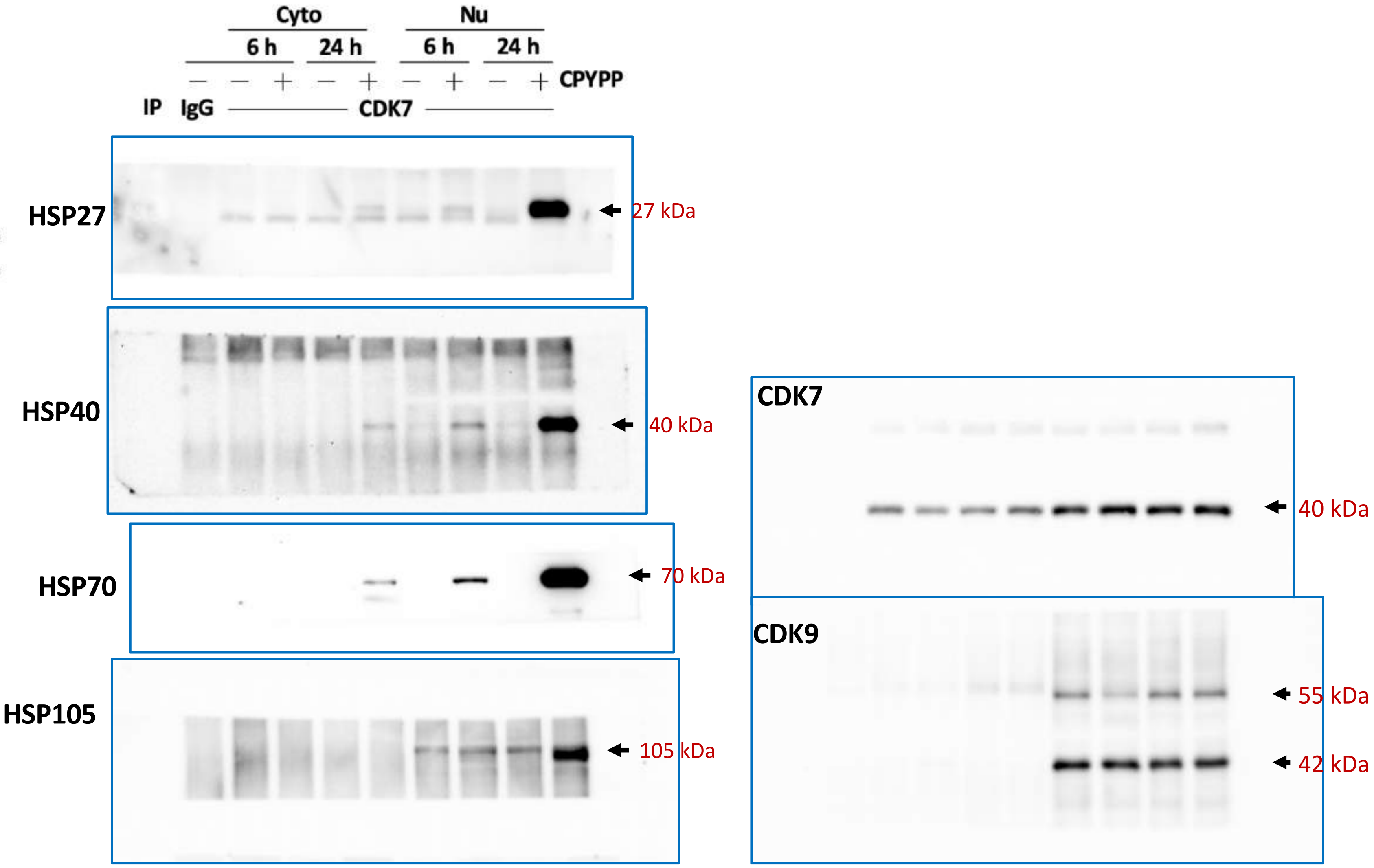

Fig. S8 (right panel)

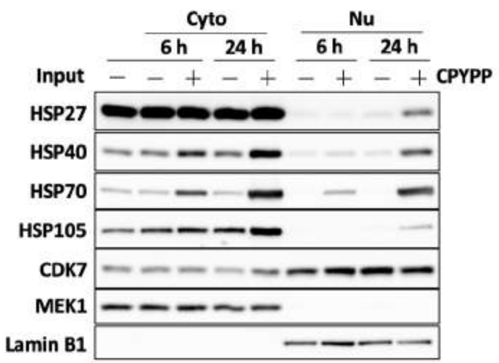

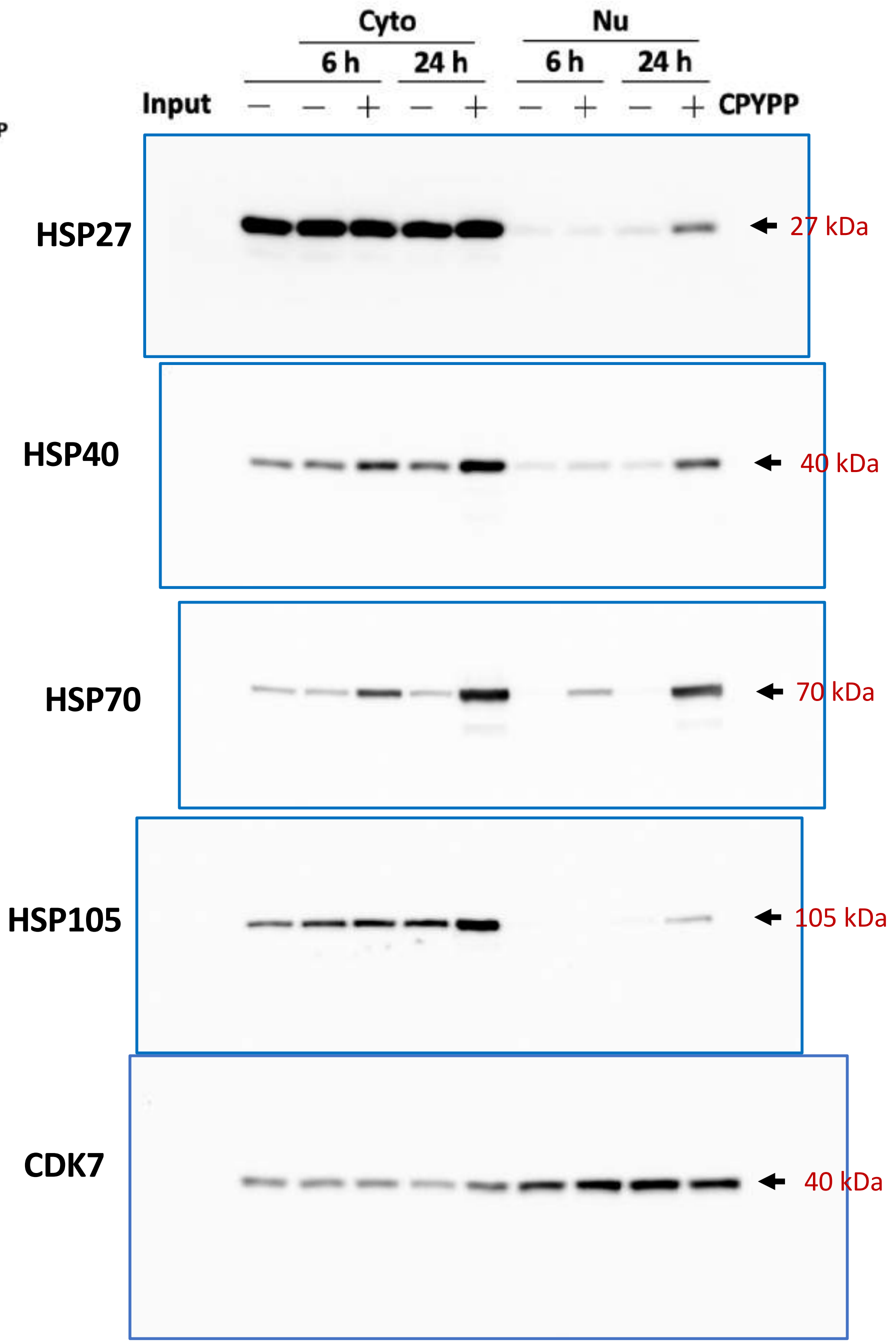

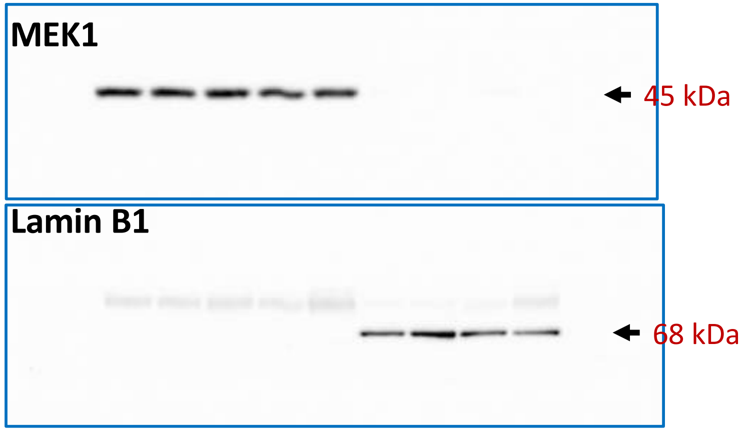

Fig. S9A

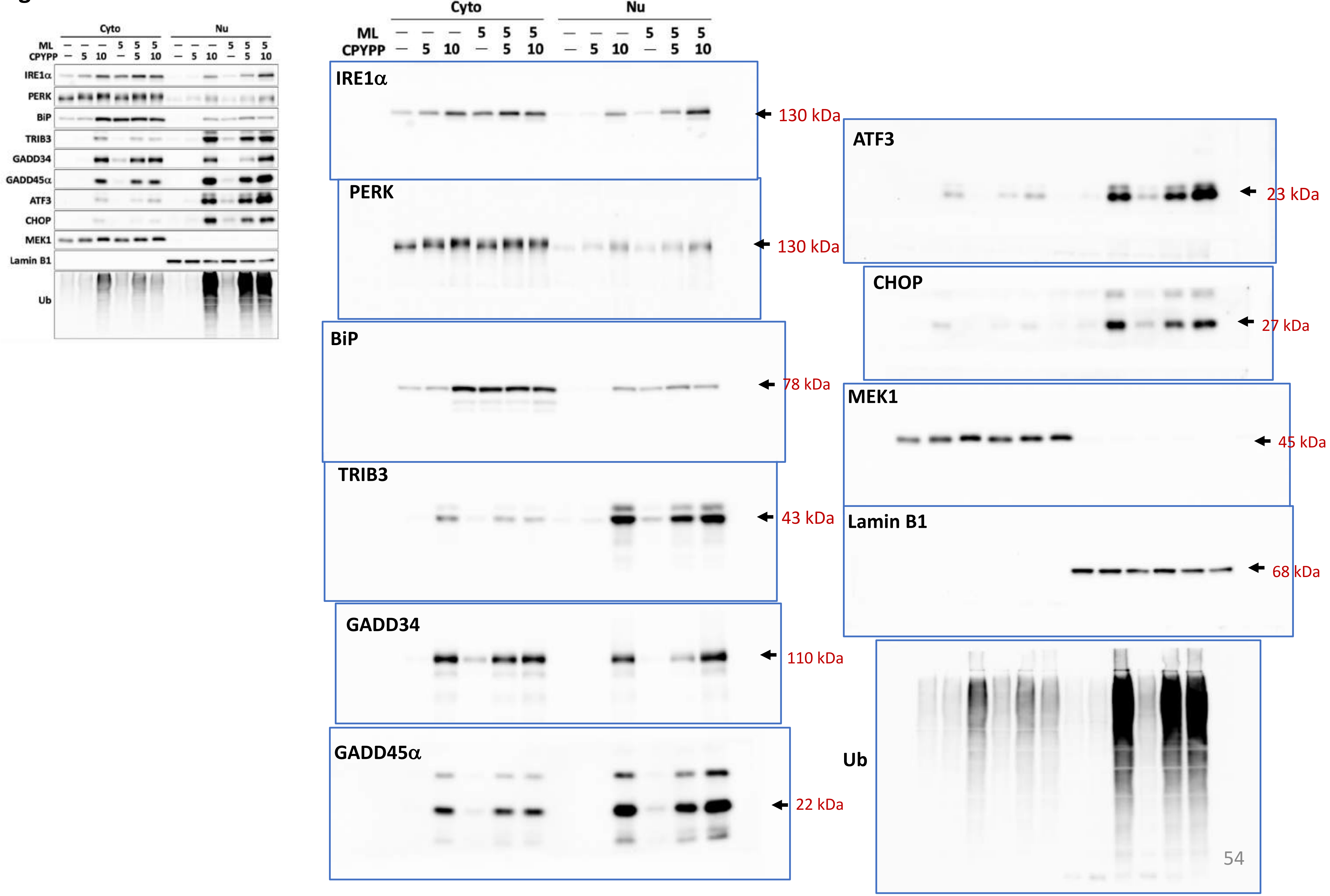

Cyto
Nu
ML
CPYPP
IRE1α
PERK
BiP
TRIB3
GADD34
GADD45α
ATF3
CHOP
MEK1
Lamin B1
Ub
130 kDa
130 kDa
78 kDa
43 kDa
110 kDa
22 kDa
23 kDa
27 kDa
45 kDa
68 kDa

Fig. S10A

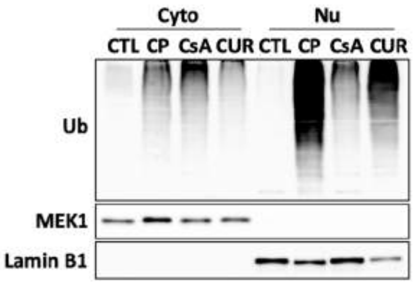

Cyto
Nu
CTL CP CsA CUR CTL CP CsA CUR
Ub
MEK1
Lamin B1

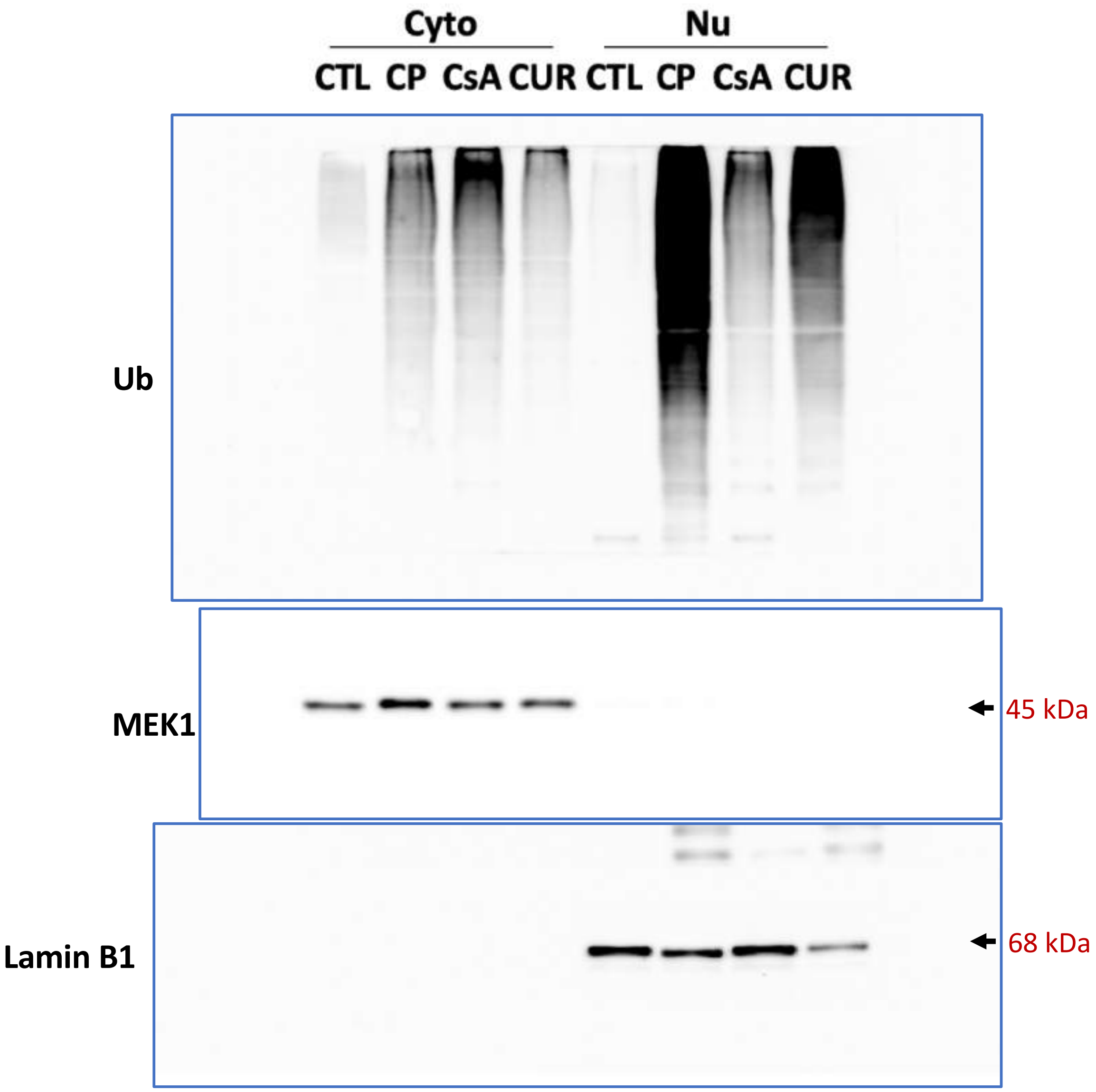

Cyto
Nu
CTL CP CsA CUR CTL CP CsA CUR
Ub
MEK1
45 kDa
Lamin B1
68 kDa

Fig. S10B

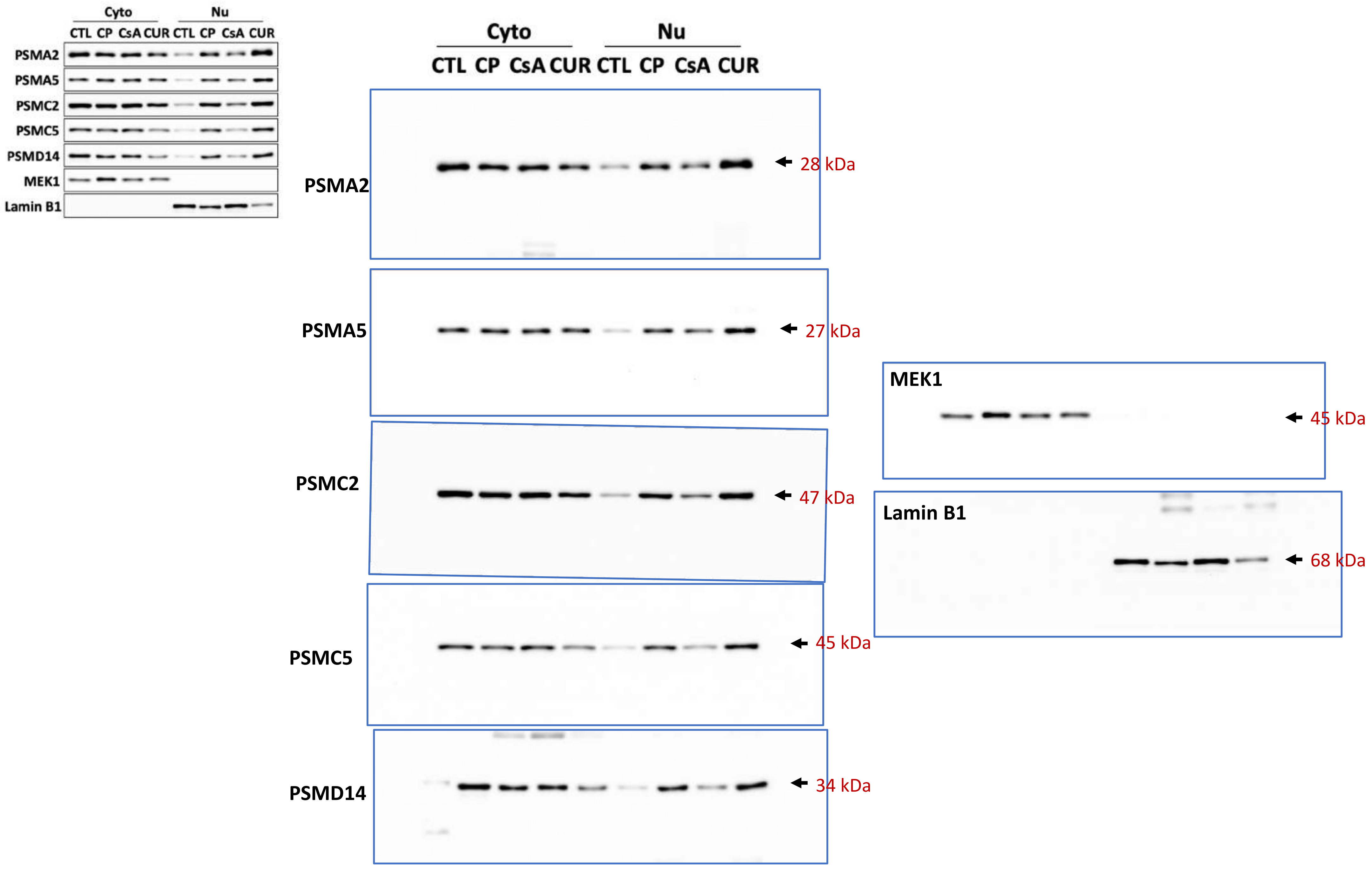
Cyto
Nu
CTL CP CsA CUR CTL CP CsA CUR
PSMA2
PSMA5
PSMC2
PSMC5
PSMD14
MEK1
Lamin B1
28 kDa
27 kDa
47 kDa
45 kDa
34 kDa
45 kDa
68 kDa

Fig. S10D

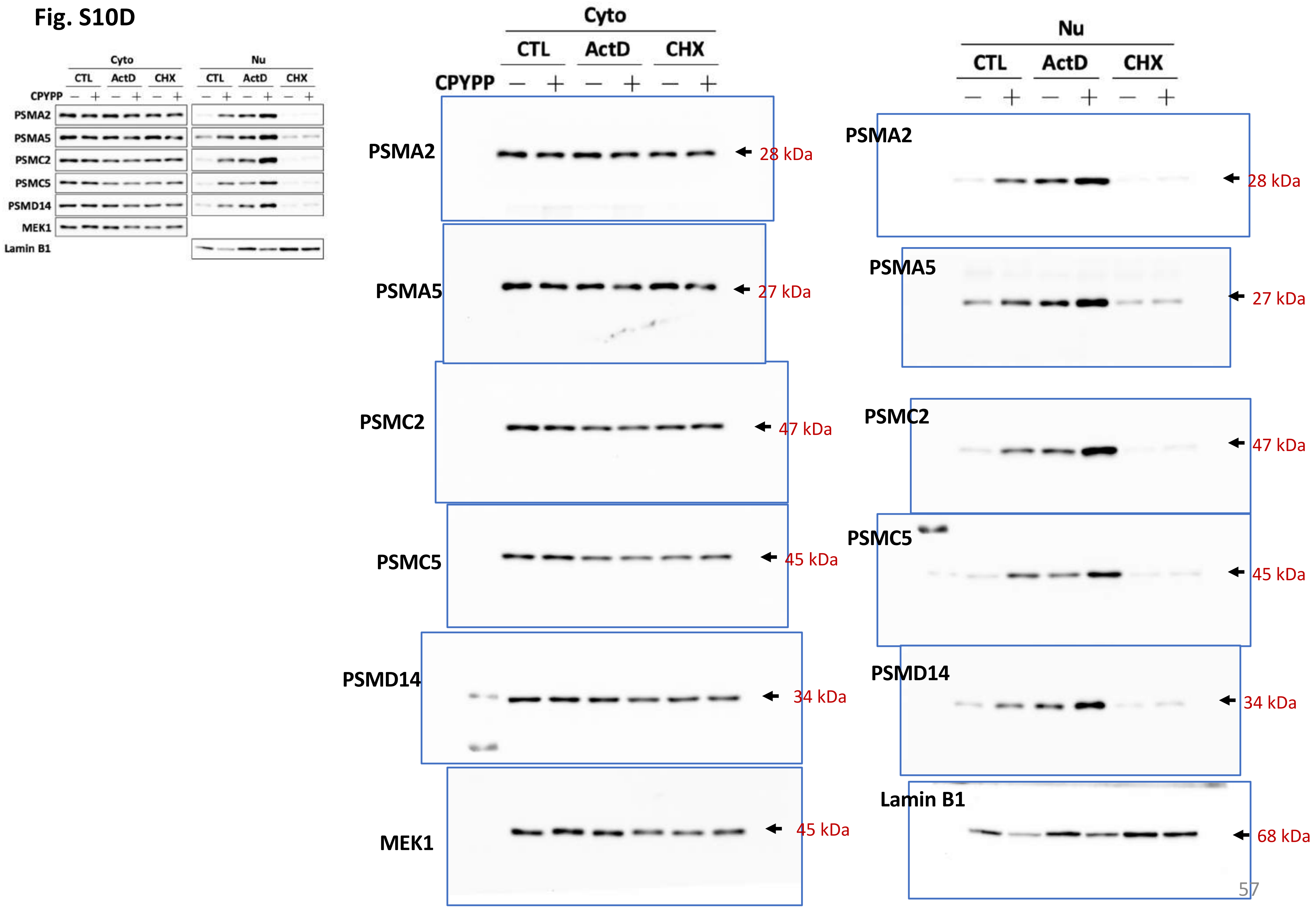

Cyto
Nu
CTL
ActD
CHX
CPYPP
PSMA2
PSMA5
PSMC2
PSMC5
PSMD14
MEK1
Lamin B1
28 kDa
27 kDa
47 kDa
45 kDa
34 kDa
45 kDa
68 kDa

Fig. S10G

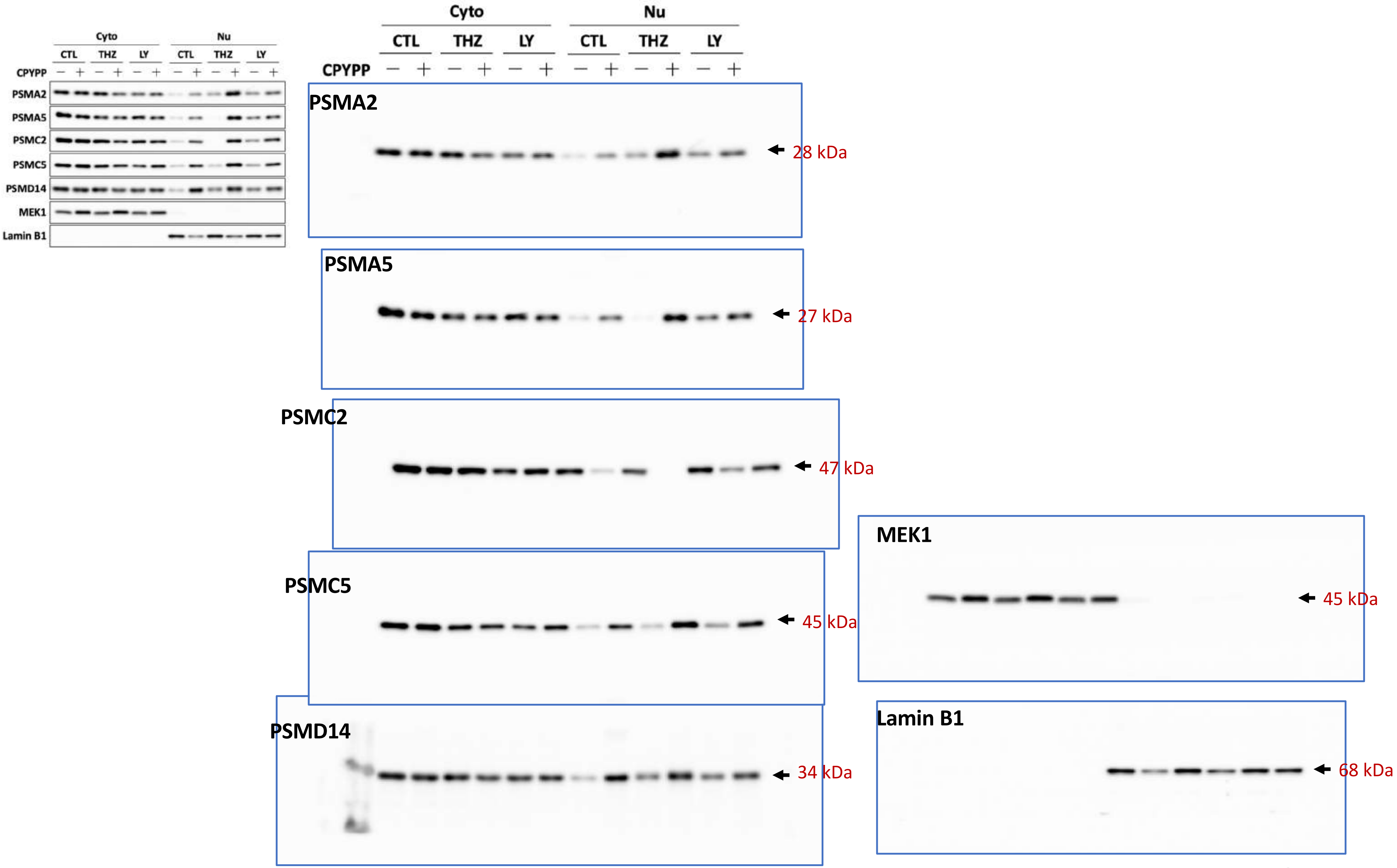

Cyto
Nu
CTL
THZ
LY
CTL
THZ
LY
CPYPP
− + − + − + − + − + − +
PSMA2
PSMA5
PSMC2
PSMC5
PSMD14
MEK1
Lamin B1
28 kDa
27 kDa
47 kDa
45 kDa
34 kDa
45 kDa
68 kDa

Fig. S10I

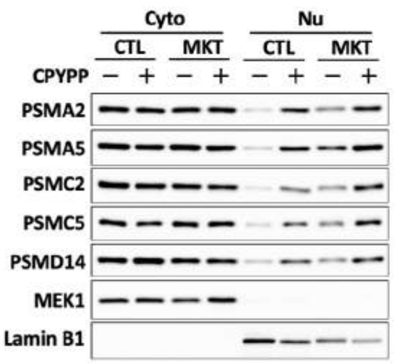

Cyto
Nu
CTL
MKT
CTL
MKT
CPYPP – + – + – + – +
PSMA2
PSMA5
PSMC2
PSMC5
PSMD14
MEK1
Lamin B1

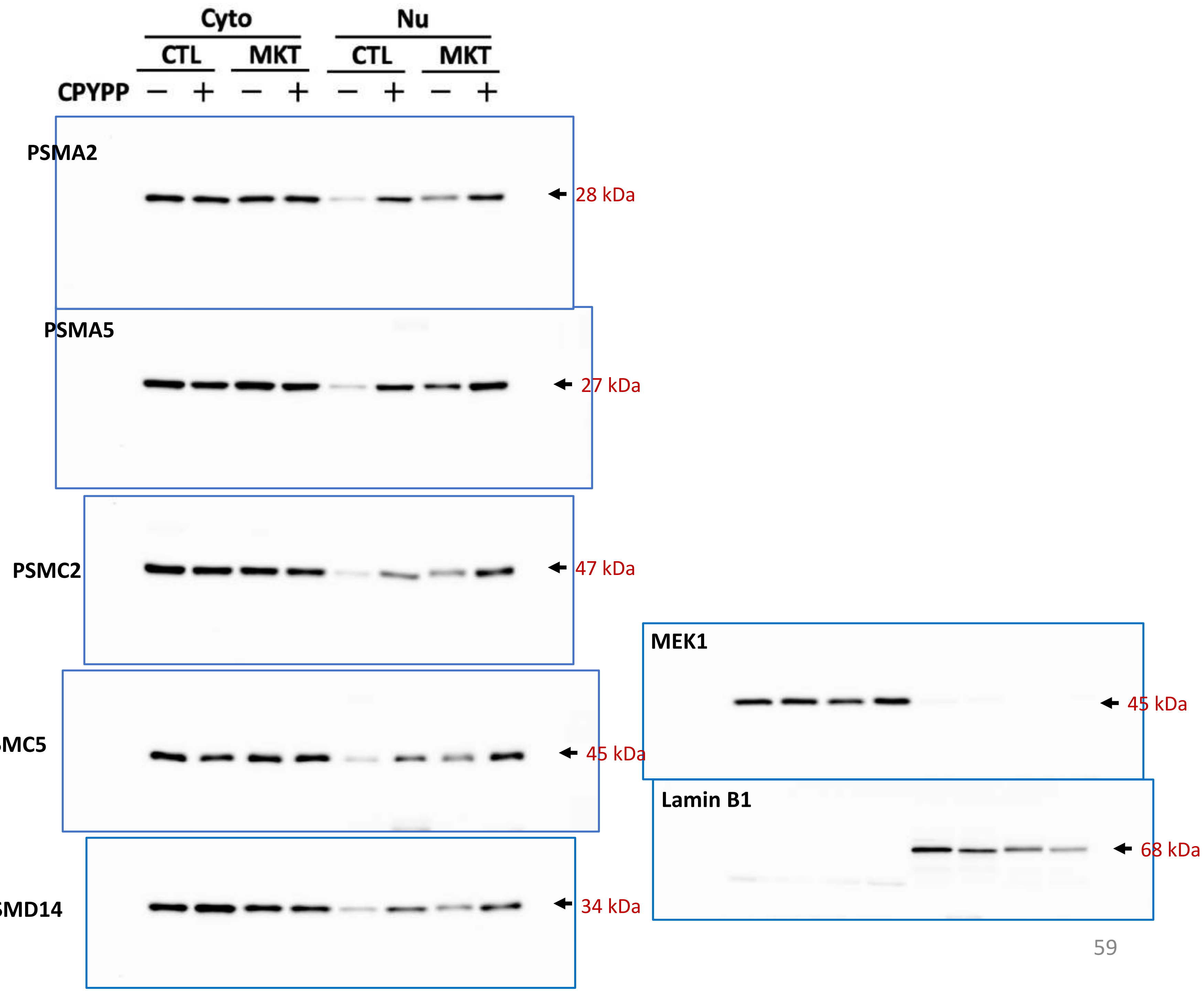

Cyto
Nu
CTL
MKT
CTL
MKT
CPYPP – + – + – + – +
PSMA2
28 kDa
PSMA5
27 kDa
PSMC2
47 kDa
PSMC5
45 kDa
PSMD14
34 kDa
MEK1
45 kDa
Lamin B1
68 kDa

Fig. S10J

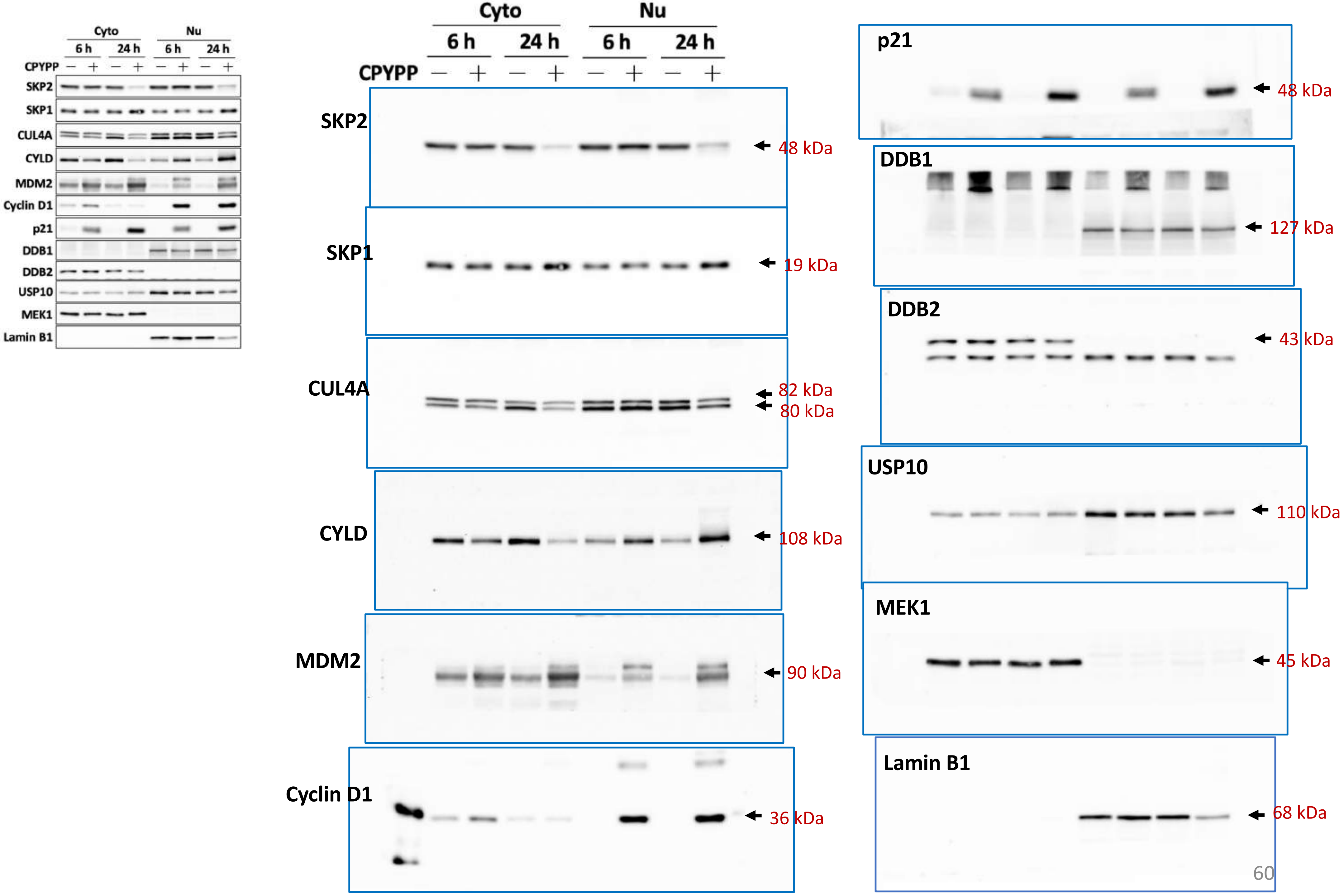
Cyto
Nu
6 h
24 h
6 h
24 h
CPYPP
SKP2
SKP1
CUL4A
CYLD
MDM2
Cyclin D1
p21
DDB1
DDB2
USP10
MEK1
Lamin B1
48 kDa
19 kDa
82 kDa
80 kDa
108 kDa
90 kDa
36 kDa
48 kDa
127 kDa
43 kDa
110 kDa
45 kDa
68 kDa

Fig. S11A

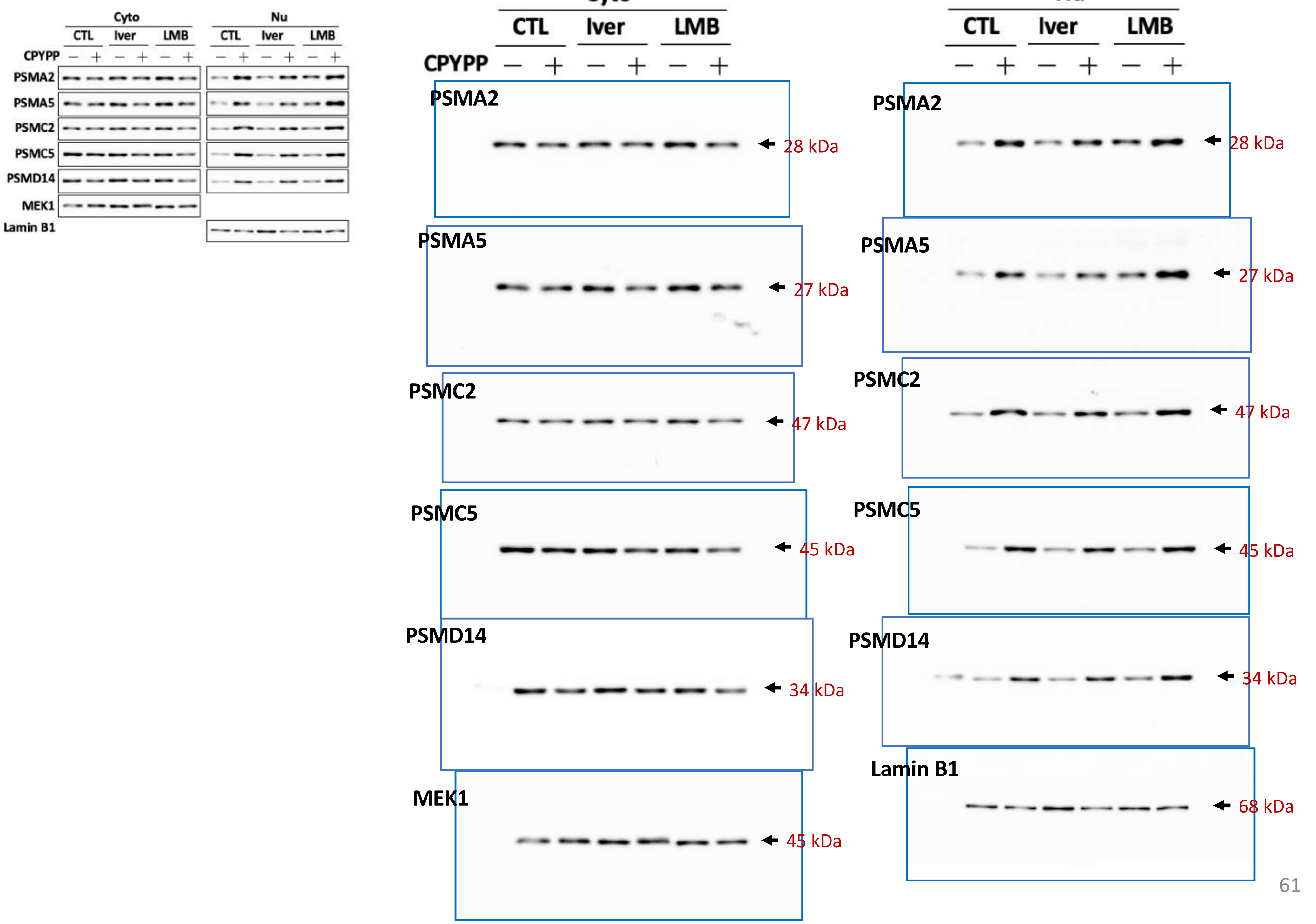

Cyto
Nu
CTL
Iver
LMB
CPYPP
PSMA2
PSMA5
PSMC2
PSMC5
PSMD14
MEK1
Lamin B1
28 kDa
27 kDa
47 kDa
45 kDa
34 kDa
45 kDa
68 kDa

Fig. S12B (left panel)

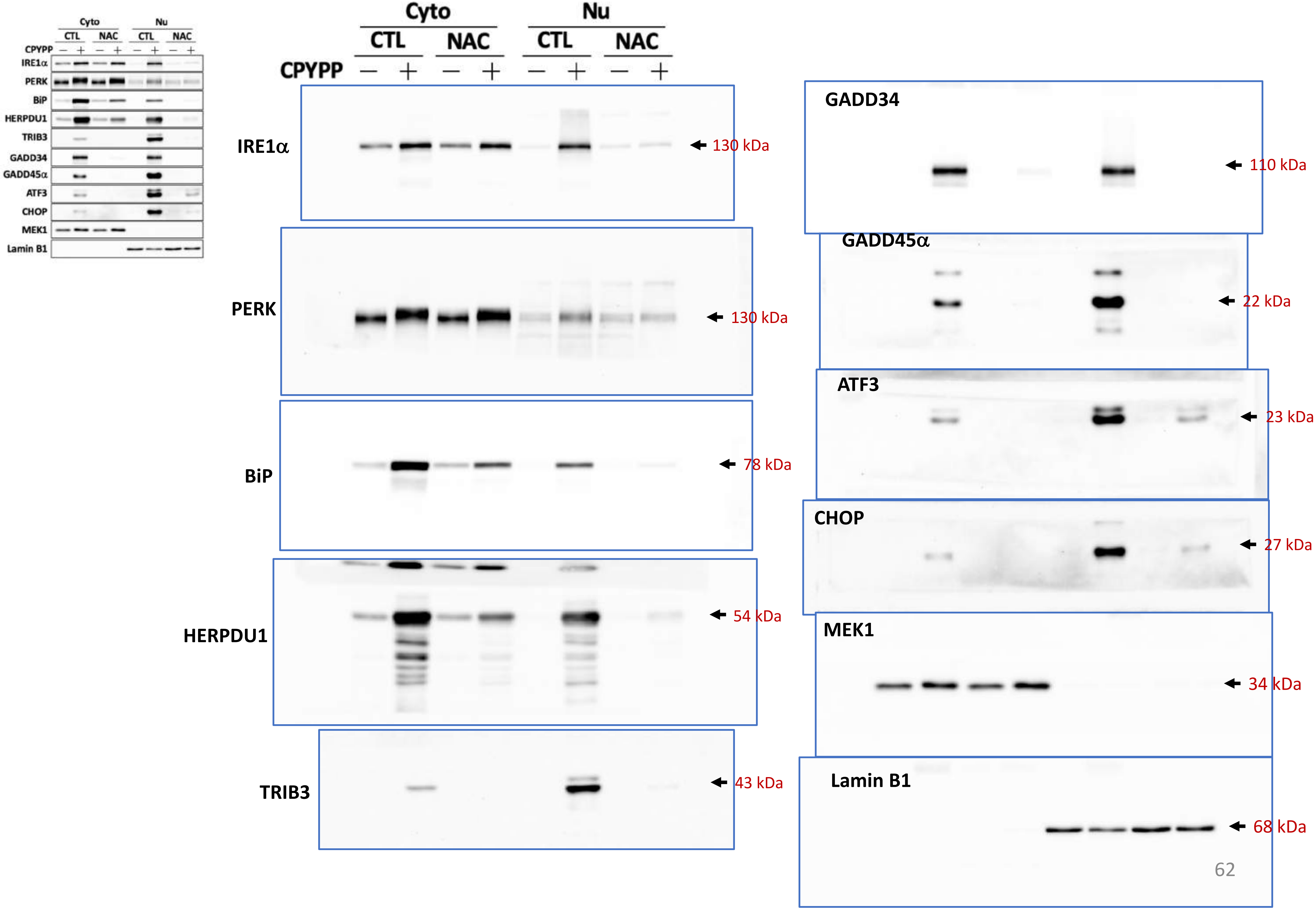

Fig. S12B (right panel)

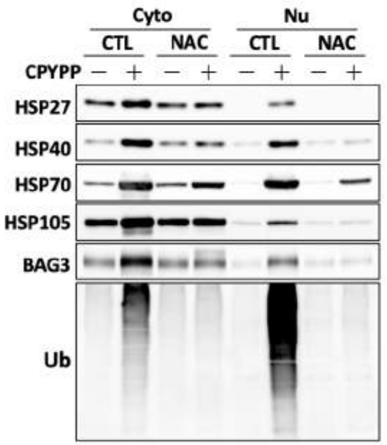

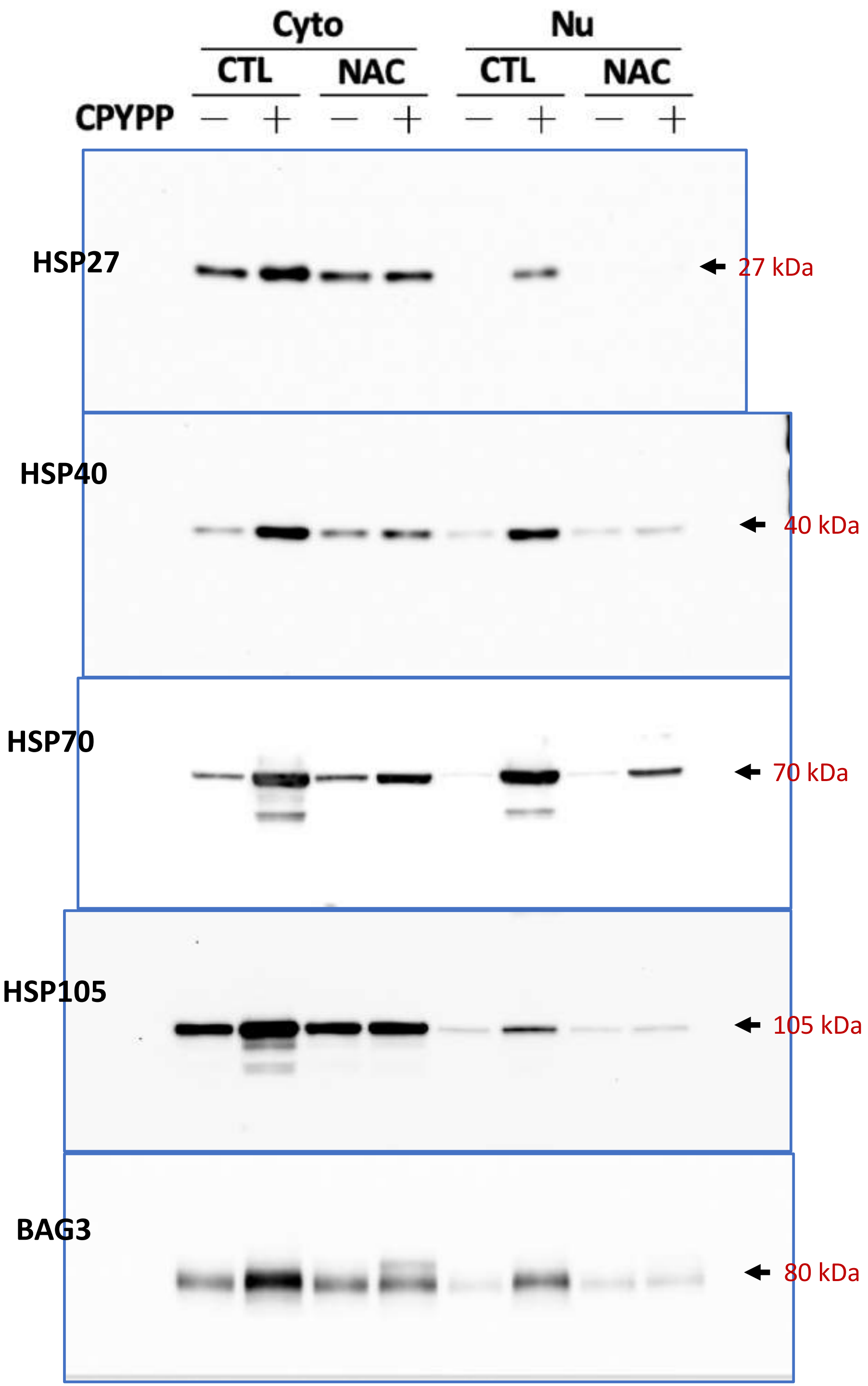

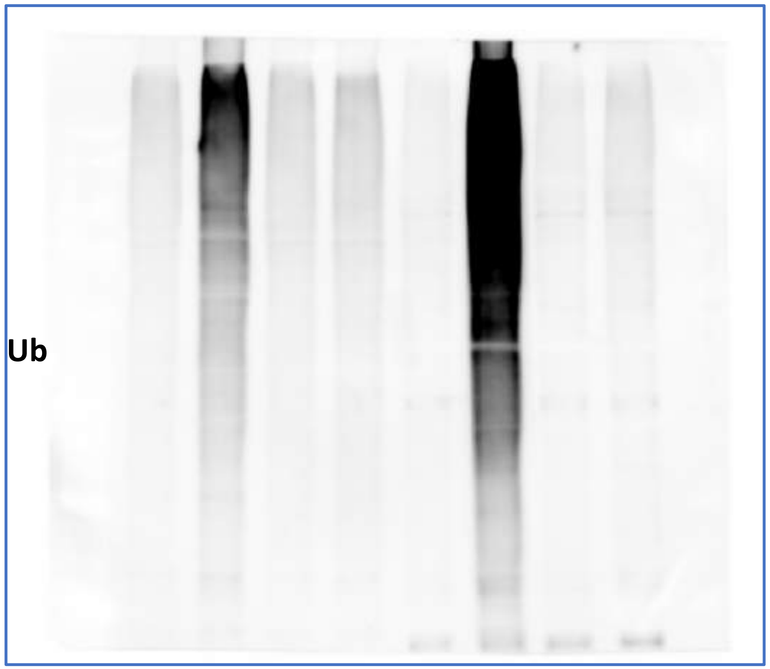

Fig. S12C

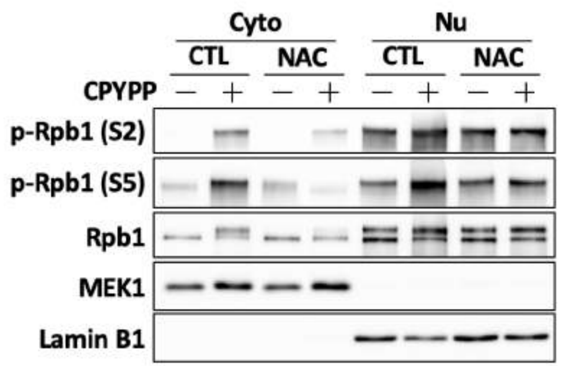
Cyto
Nu
CTL
NAC
CTL
NAC
CPYPP – + – + – + – +
p-Rpb1 (S2)
p-Rpb1 (S5)
Rpb1
MEK1
Lamin B1

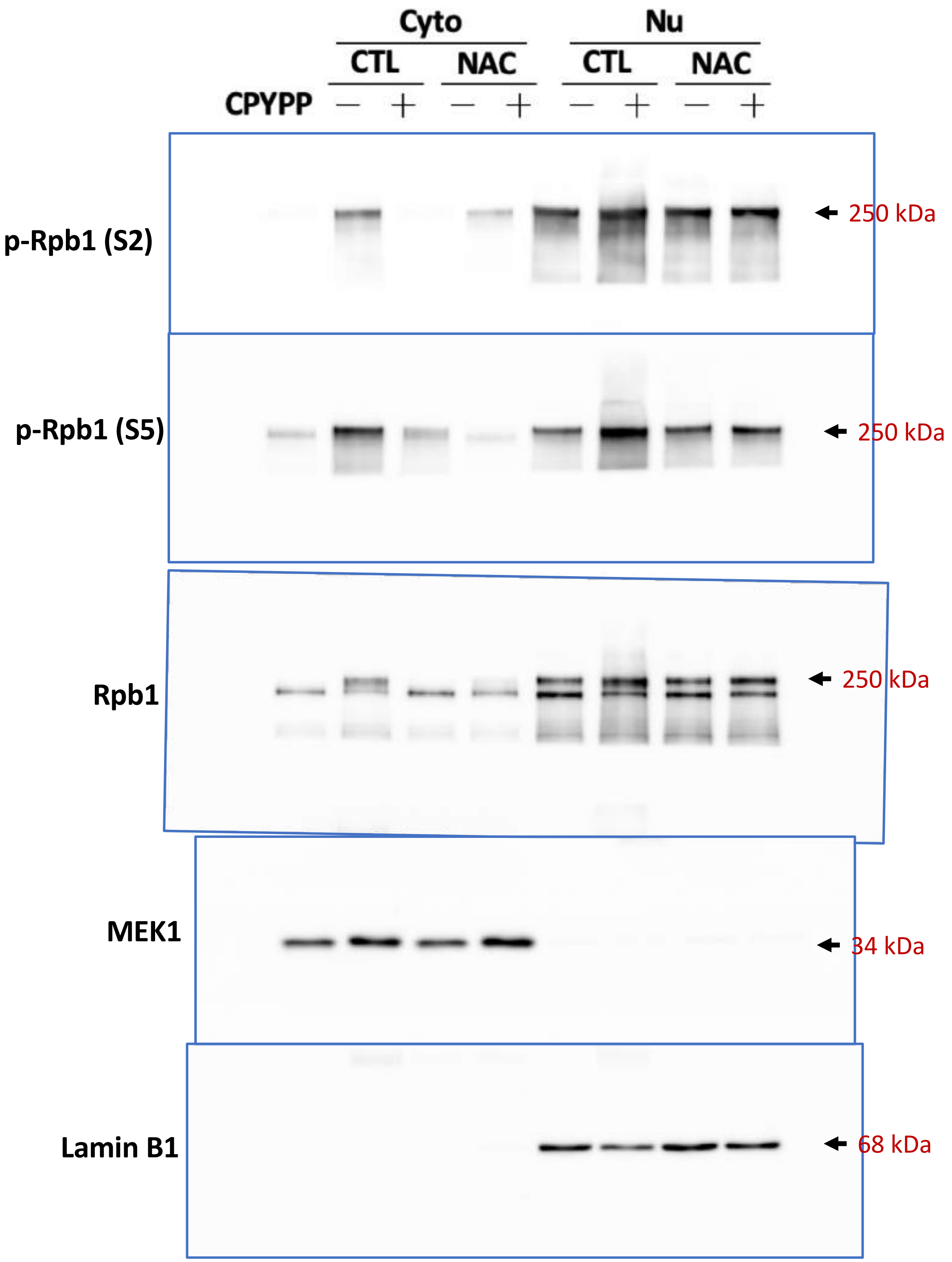
Cyto
Nu
CTL
NAC
CTL
NAC
CPYPP – + – + – + – +
p-Rpb1 (S2)
250 kDa
p-Rpb1 (S5)
250 kDa
Rpb1
250 kDa
MEK1
34 kDa
Lamin B1
68 kDa

Fig. S12D

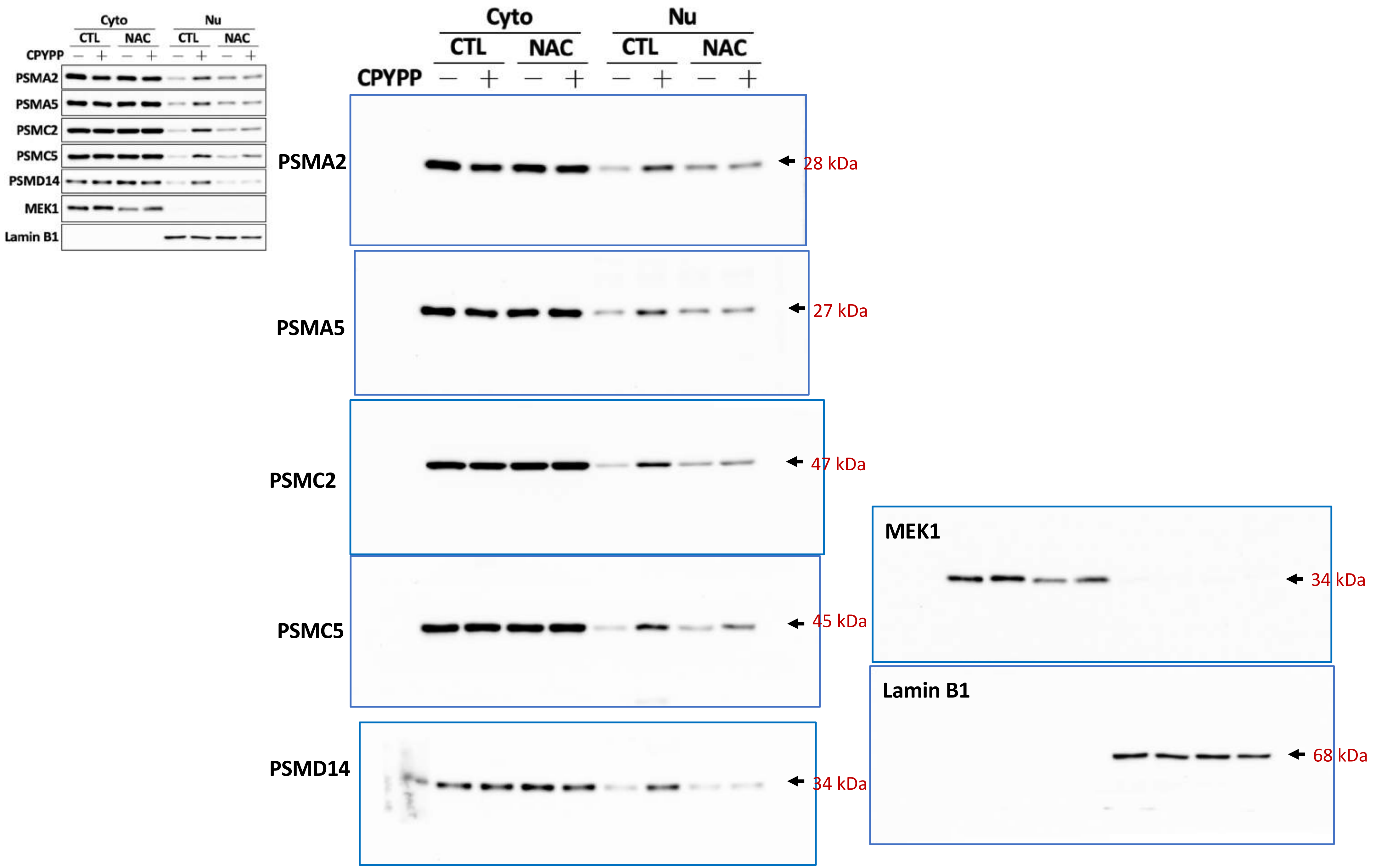

Cyto
Nu
CTL
NAC
CTL
NAC
CPYPP
− + − + − + − +
PSMA2
PSMA5
PSMC2
PSMC5
PSMD14
MEK1
Lamin B1
28 kDa
27 kDa
47 kDa
45 kDa
34 kDa
34 kDa
68 kDa

Fig. S13

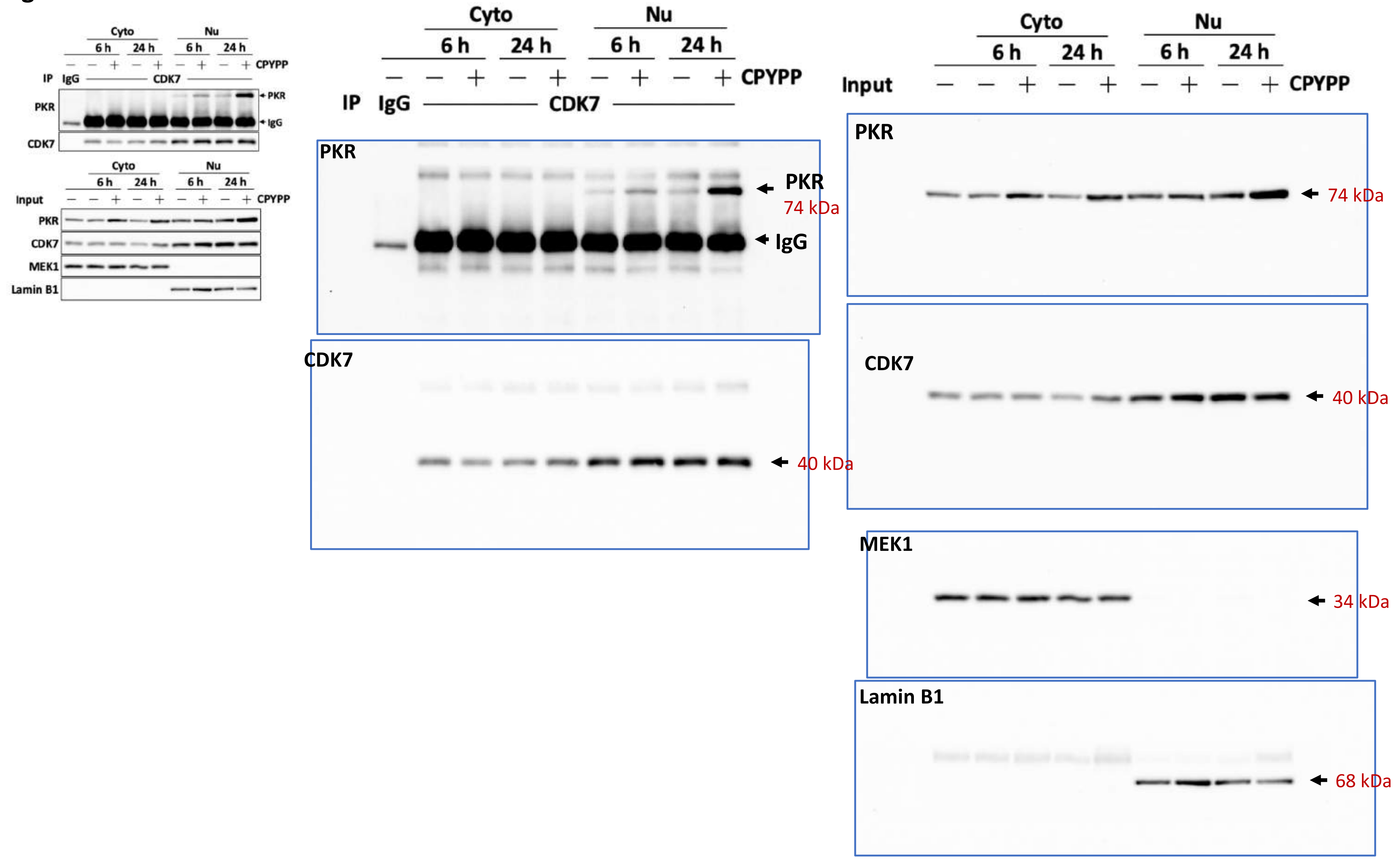
Cyto
Nu
6 h
24 h
IP IgG CDK7
CPYPP
PKR
IgG
CDK7
Input
MEK1
Lamin B1
74 kDa
40 kDa
34 kDa
68 kDa

Fig. S14A

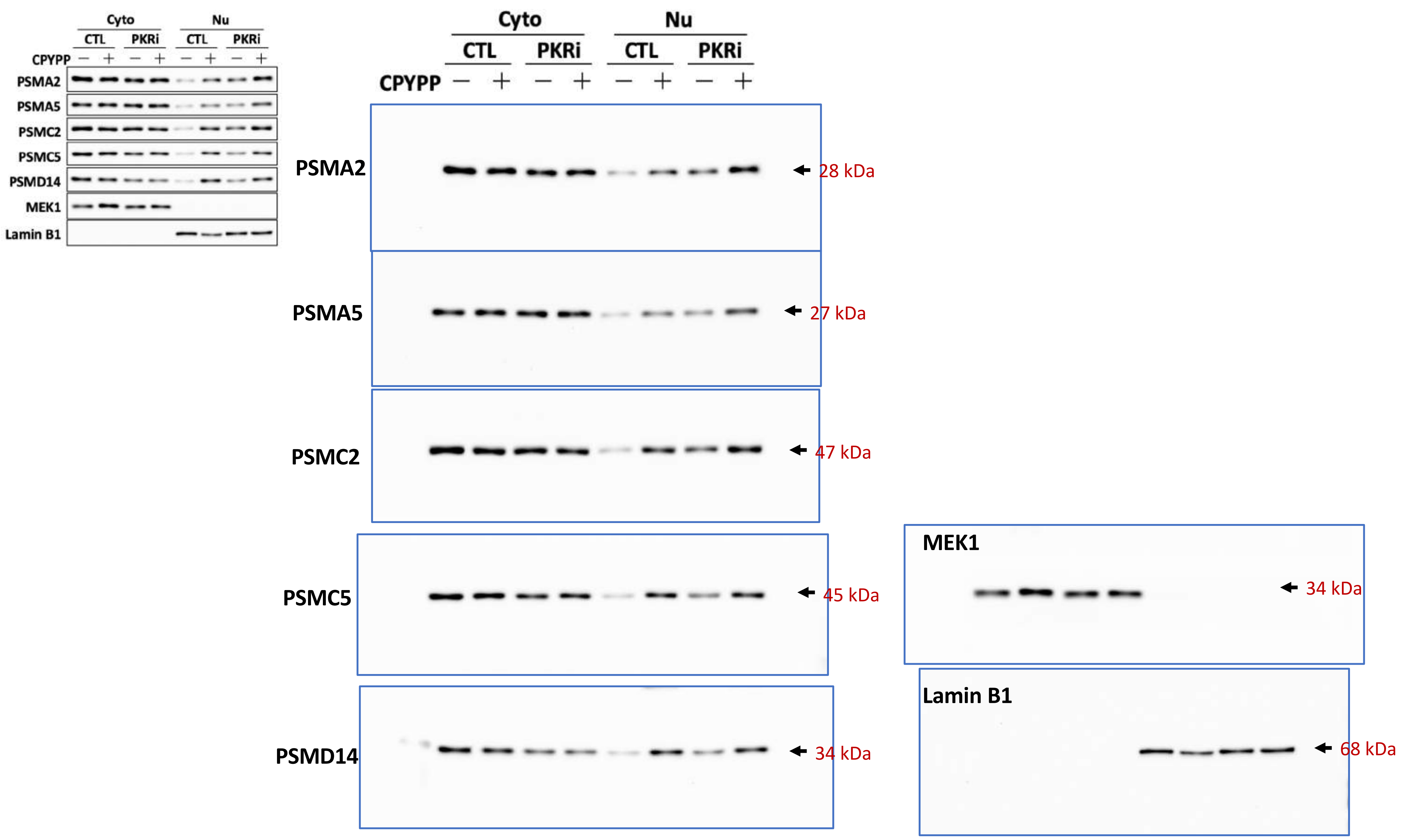
Cyto
Nu
CTL
PKRi
CTL
PKRi
CPYPP
PSMA2
PSMA5
PSMC2
PSMC5
PSMD14
MEK1
Lamin B1
28 kDa
27 kDa
47 kDa
45 kDa
34 kDa
34 kDa
68 kDa
